# Probabilistic modelling of chromatin code landscape reveals functional diversity of enhancer-like chromatin states

**DOI:** 10.1038/ncomms10528

**Published:** 2016-02-04

**Authors:** Jian Zhou, Olga G. Troyanskaya

**Affiliations:** 1 Lewis-Sigler Institute for Integrative Genomics, Princeton University, Princeton, New Jersey 08540, USA; 2 Graduate Program in Quantitative and Computational Biology, Princeton University, Princeton, New Jersey 08540, USA; 3 242 Carl Icahn Laboratory, Department of Computer Science, Princeton University, Princeton, New Jersey 08540, USA; 4 Simons Center for Data Analysis, Simons Foundation, New York, New York 10010, USA

## Abstract

Interpreting the functional state of chromatin from the combinatorial binding patterns of chromatin factors, that is, the chromatin codes, is crucial for decoding the epigenetic state of the cell. Here we present a systematic map of *Drosophila* chromatin states derived from data-driven probabilistic modelling of dependencies between chromatin factors. Our model not only recapitulates enhancer-like chromatin states as indicated by widely used enhancer marks but also divides these states into three functionally distinct groups, of which only one specific group possesses active enhancer activity. Moreover, we discover a strong association between one specific enhancer state and RNA Polymerase II pausing, linking transcription regulatory potential and chromatin organization. We also observe that with the exception of long-intron genes, chromatin state transition positions in transcriptionally active genes align with an absolute distance to their corresponding transcription start site, regardless of gene length. Using our method, we provide a resource that helps elucidate the functional and spatial organization of the chromatin code landscape.

## Abstract (web summary)

The chromatin functional state can be derived from the binding patterns of chromatin factors. Here, Zhou and Troyanskaya report a data-driven probabilistic modelling of dependencies between chromatin factors that can divide enhancer-like chromatin states into three functionally distinct groups.

## Graphical abstract

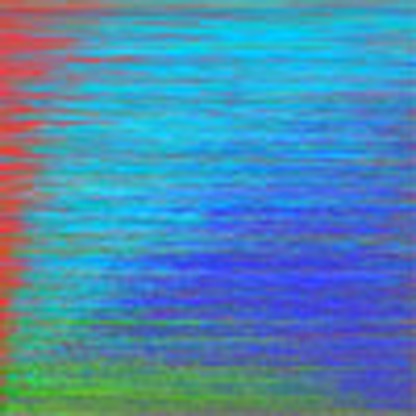

Large-scale chromatin profiling efforts in modENCODE, ENCODE and Roadmap Epigenomics projects^1^,^2^,^3^,^4^ are providing an increasingly complete picture of chromatin organization by generating genome-wide binding profile data of diverse chromatin factors, including histones with posttranslational modifications, transcription factors (TFs) and non-TF chromatin-associated proteins. Chromatin protein-binding patterns observed across diverse genomic regions, cell types, developmental stages and environmental conditions provide rich information on the spatial-temporal functional status of chromatin. Each distinct chromatin factor pattern can be considered a chromatin code that needs to be decoded to understand the transcriptional and regulatory status of chromatin. To achieve this goal, chromatin state discovery algorithms aim to identify functionally coherent groups of chromatin codes underlying specific chromatin states.

Previous methods for probabilistic modelling of chromatin states have yielded significant new insights into chromatin states^5^,^6^,^7^,^8^, but they did not consider dependencies between different chromatin factors at the same genomic location. We previously showed that given the existence of strong and widespread interactions between chromatin factors, including physical and catalytic interactions between histone marks and chromatin proteins, accounting for such interactions leads to greatly improved accuracy in the probability estimation of chromatin codes^9^. Furthermore, we expected the improved estimation of the chromatin code probability landscape to lead to improved chromatin state detection. To this end, we have developed a novel approach for identification of chromatin states that models the chromatin code organization while considering statistical dependencies and interactions between chromatin factors at the same genomic location.

Using our probabilistic, dependency aware approach, we systematically identified and characterized 30 chromatin states from genome-wide profiles of 73 histone marks and non-histone chromatin proteins from the *Drosophila melanogaster* S2-DRSC cell^1^. Among these states, we identified three distinct functional groups of enhancer-like states carrying the widely used active enhancer marks H3K4me1 and H3K27ac, where only one of these groups was strongly associated with active enhancers and explained the majority of STARR-seq^10^ detected strong enhancers. In addition, we discovered a specific chromatin state with a previously uncharacterized association with Pol II pausing. We also observed that the spatial organization of chromatin states in actively transcribed genes were mostly determined by an absolute distance to transcription start site (TSS) and appeared to be invariant to gene length. In particular, a specific group of chromatin states only appeared in active genes with long introns and exhibited remarkably different spatial organization. Identification of these novel properties of chromatin states provides new insights into the regulatory information carried by the chromatin factors.

## Results

### Systematic identification of chromatin states

**Figure 1:**
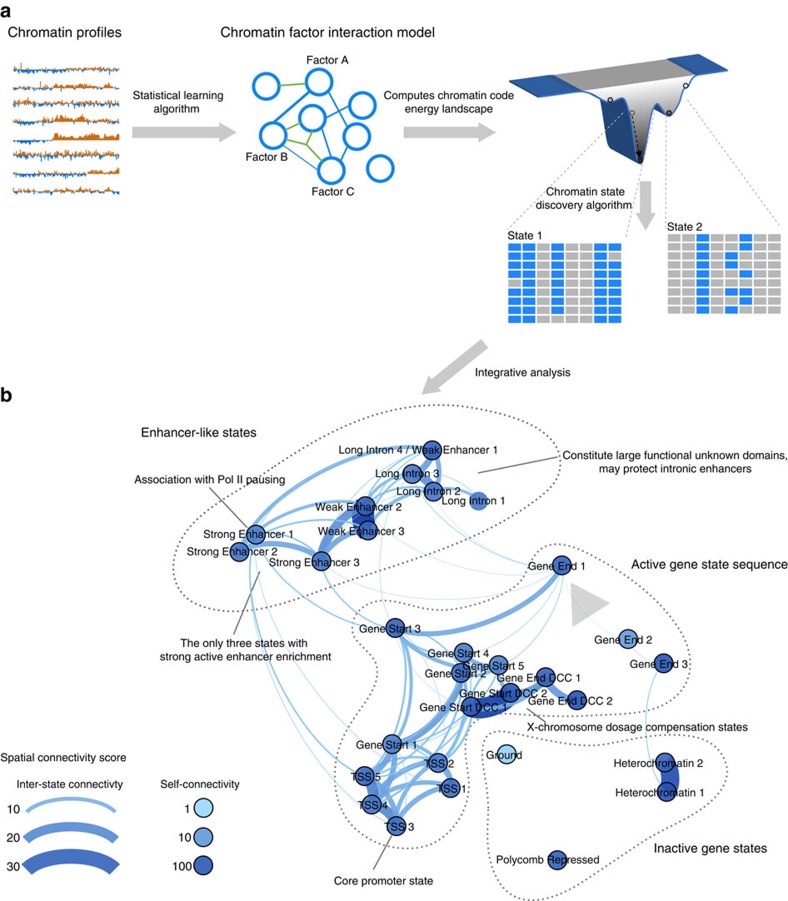
Discovery of chromatin states from the chromatin code energy landscape. (**a**) Schematic overview of the chromatin state identification algorithm. Chromatin factor pattern energy landscape is given by the chromatin factor interaction model learned from large-scale chromatin profiling data (blue and green edges indicate positive and negative interactions, respectively; a positive interaction indicates that the presence of one factor has a positive effect on the presence probability of the other factor, whereas a negative interaction indicates a negative effect. See Methods section for formal mathematical definitions). Our chromatin state discovery algorithm uses optimization-based approach to identify the stable energy minima in the energy landscape starting from any chromatin factor pattern. Chromatin factor patterns within the same energy valley are recognized by the algorithm as arriving to the same local minimum and are grouped as a chromatin state. (**b**) Overview of the spatial and functional organization of the chromatin states discovered by our approach. The major groups of chromatin states include the following: (1) the enhancer-like states that include distinct functional groups of states carrying the widely used active enhancer marks H3K27ac and H3K4me1, (2) the active gene state sequence, for which we indicate the 5′→3′ sequence of states with a grey arrow, and (3) the inactive gene states. Significant findings about specific chromatin states are remarked. Edge width and colour indicates spatial connectivity score between chromatin states; a score of 30 indicates that the frequency of observing the two states in question being adjacent to each other in the genome is 30 times higher than expected by chance. Node colour darkness indicates the level of spatial connectivity score of the state to itself; most states appear very stable as indicated by the high self-connectivity score.

To identify chromatin states from genome-wide *D. melanogaster* chromatin profiling data, we first inferred chromatin factor interactions through a maximum entropy model of all chromatin factor profiles^9^. The chromatin state identification algorithm can be interpreted as discovering chromatin states as valleys in the energy landscape of the maximum entropy model of chromatin code (Fig. 1a and Methods), where lower energy corresponds to higher probability. The energy of each set of chromatin codes was directly computed from the model based on estimated interactions. For example, a chromatin code containing mostly positively interacting and few negatively interacting chromatin factors would have low energy and equivalently higher probability.

More specifically, the algorithm finds the local minima in the energy landscape by simulating a Hopfield-network-type dynamical system naturally derived from the maximum entropy model. Initialized from any chromatin code, the dynamic system monotonically decreases the energy by walking down the energy landscape until reaching a local minimum. This procedure identifies all observed chromatin codes residing in the same valley of the chromatin factor energy landscape by detecting that they reach the same local minimum. On an intuitive level, our algorithm essentially groups chromatin codes that are very similar to each other given the interactions of chromatin factors.

**Figure 2:**
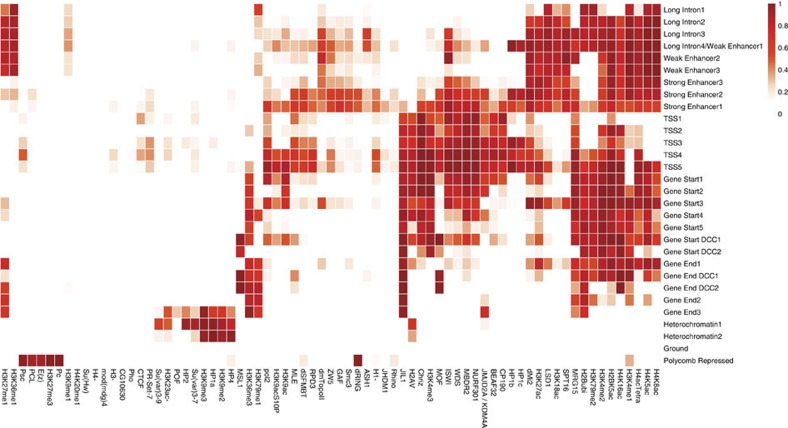
Distinct functional properties of chromatin states reflected in state-specific chromatin factor compositions. Chromatin factor compositions of each chromatin state, showing the average frequency of observing each of the 73 chromatin factors within each state. Each chromatin state displays distinctive marks. The *x* axis shows the 73 chromatin factors, whereas the *y* axis shows the 30 chromatin states. It is noteworthy that for chromatin factors H3K23ac-, H1-, H3- and H4-, the average frequency of these protein/marks being absent rather than present is shown, in consistency with the model. Only chromatin factors enriched with *P*-value<0.01 by permutation test are shown. The null distribution of the permutation test is computed by permuting chromatin state annotations by 50-kbp-length blocks.

**Figure 3:**
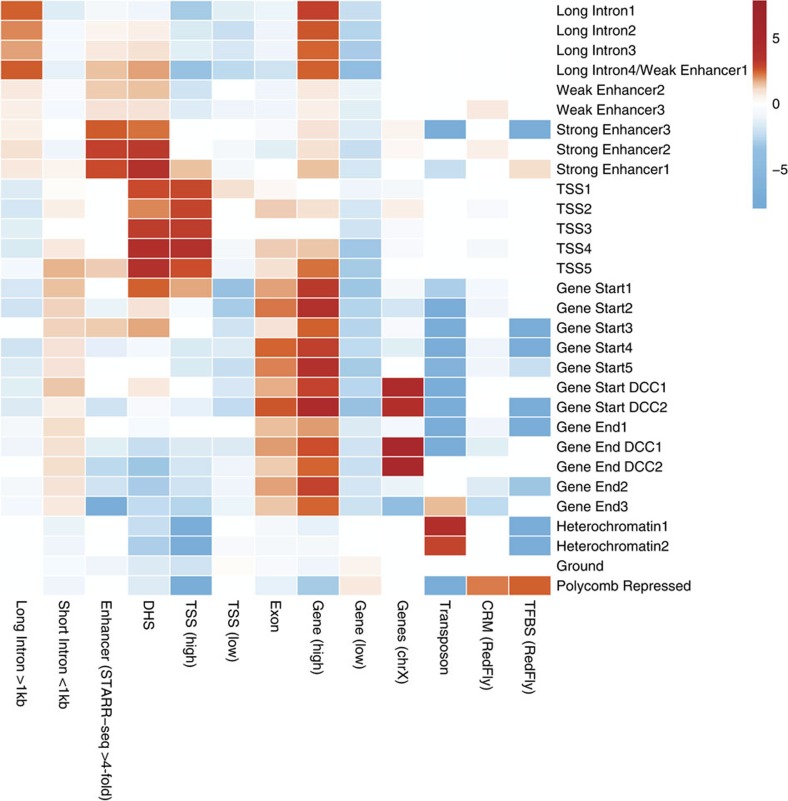
Integrative functional characterization of chromatin states. Chromatin states show distinct functional genomic elements enrichment patterns. Enrichment for various genomic elements (for example, the presence of long introns or TSS) or external experimental observations (for example, sequences capable of driving reporter expression in STARR-seq assays or DNase I hypersensitivity sites) were measured as the difference between log odds of observed overlapping region proportion and the log odds expected, assuming independence in spatial positioning. Only functional element enrichment or depletion with *P*-value<0.01 by permutation test is shown. The null distribution of the permutation test is computed by permuting chromatin state annotations by 50-kbp-length blocks. In the *x*-axis: CRM, *cis*-regulatory modules from REDfly database^11^; DHS, DNase I hypersensitive sites^10^; Enhancer (STARR-seq), active enhancer elements in S2-DRSC cell identified by STARR-seq with over fourfold expression change^10^; Exon: Genes (chrX), genes located in chromosome X, which is dosage compensated; Gene (Active), actively transcribed genes according to RNA-seq; Gene (Inactive), inactive or low expression genes according to RNA-seq; TFBS, transcriptional factor binding site from REDfly database^11^; Transposon, annotated transposons from FlyBase annotation^12^; TSS (Active), actively transcribed TSS according to RNA-seq (see Methods for details); TSS (Inactive), lowly expressed or repressed TSS according to RNA-seq.

When applied to the 73 chromatin factor profiles of *D. melanogaster* S2-DRSC cells, the algorithm identified 30 chromatin states with specific functional properties and spatial characteristics (Figs 1b, 2 and 3, and Supplementary Data 1). The connections between chromatin states were naturally revealed by the spatial connectivity between each pair of states (Fig. 1b). The spatial connectivity organization of chromatin states is highly consistent with functional similarities of chromatin states shown by chromatin factor composition and enrichment patterns of diverse functional genomic elements (Figs 2 and 3, and Supplementary Data 2 and 3).

### Architecture of the chromatin state landscape

We discovered several groups of chromatin states exhibiting unexpected properties based on the enrichment of chromatin states in diverse functional genomic elements. The chromatin states we identified can be summarized into three groups: (1) the enhancer-like states, (2) the canonical active gene state sequence and (3) the inactive gene states (Fig. 1b).

Among the enhancer-like states (those carrying widely used active enhancer marks H3K27ac and H3K4me1), we found distinct groups with striking functional differences and their differentiating marks are valuable in identifying more functionally specific chromatin states. With regards to the canonical active gene state sequence, the majority of active genes have a canonical 5′→3′ chromatin state sequence that spans the TSS states, Gene Start states and ends at the Gene End states. We discovered that transition positions between chromatin states in the whole sequence are dependent on the distance to TSS, but invariant to gene length (see Canonical spatial sequence of active gene chromatin states section). The inactive gene states include the well-known Polycomb-Repressed state (marked by H3K27me3 and Polycomb group proteins), the Heterochromatin states (marked by H3K9me3 and HP1a) and the Ground state. The Ground state covers the majority of inactive genes and intergenic regions, and shows no enrichment of any chromatin factor; thus, it may be considered as a default chromatin state.

We summarized the nomenclature and distinctive features of each chromatin state in Supplementary Table 1 and showed that previous studies did not distinguish the key functional states that we discuss below (Supplementary Note 1 and Supplementary Figs 1–5). Furthermore, we systematically analysed the dependency of chromatin state identification on each single chromatin factor through perturbation analyses. We demonstrated that the identification of the vast majority of the chromatin states were not affected by removing any single chromatin factor, whereas flipping the on–off state of single chromatin factors had a larger effect and, as such, revealed key identity chromatin factors for each chromatin state (Supplementary Note 2, Supplementary Figs 6–8 and Supplementary Data 4).

### Discovery of functional diversity among enhancer-like states

We identified a meta-group of states, which we call enhancer-like states, carrying the widely used enhancer marks, H3K27ac and H3K4me1 (Figs 1b and 2). However, unexpectedly we also found that the enhancer-like states were each associated with distinct genomic elements and possessed different chromatin factor compositions. Based on their genomic element enrichment, we grouped enhancer-like states into Strong Enhancer (SE), Weak Enhancer and Long Intron states.

Strong Enhancer states were compositionally distinct from other enhancer-like states for chromatin factor marks, for example, exhibiting low levels of H3K36me1 and H3K27me1 (91.4% of Weak Enhancer and Long Intron states possess H3K36me1, whereas this is only true for 22.4% of SE states; similar patterns were observed for H3K27me1). They were also the only group of enhancer-like states that showed significant active enhancer activity as identified by genome-wide screening of regulatory sequences with STARR-seq^10^ (Fig. 3). Even though SE states only cover 3.7% of the genome (Supplementary Table 2), over 75% of STARR-seq enhancers with over fourfold reporter expression change and over 87% of STARR-seq enhancers with over eightfold reporter expression change were detected within 100 bp of a SE state (Supplementary Fig. 9).

Compared with the widely adopted approach of using H3K27me1 and H3K4me1 marks to define active enhancers, SE states had 2.7-fold higher specificity for the enrichment of STARR-seq enhancers. The higher specificity is explained by that in contrast to the SE states, only weak or no enrichment for STARR-seq enhancer elements or DNase I hypersensitive sites sites were observed for Weak Enhancer and Long Intron states (Fig. 3), even though they also carried H3K27ac and H3K4me1. Compared with the Weak Enhancer states and Long Intron states, SE states had respectively 4.6 and 9.6-fold higher specificity for the enrichment of STARR-seq enhancers responsible for over 4-fold expression change. Thus, we identified chromatin states that are much more specifically enriched in active enhancers and, consequently, we suggest that additional marks such as the absence of H3K36me1 or H3K27me1 should be used to distinguish SE states from other enhancer-like states (Fig. 2). These marks have not been previously identified as predictive of enhancer activity.

### SE1 state is highly predictive for Pol II pausing

Chromatin states in the TSS region have been shown to be predictive of the expression level of genes^1^,^13^,^14^,^15^, but the connection between chromatin in the transcription initiation region and the regulatory potential of gene transcription was not understood. We found that in terms of chromatin states, most active gene promoters fall into one of two groups, revealed by visualizing the space of promoter chromatin states with multidimensional scaling (Supplementary Fig. 10). Although as expected the majority of genes have promoters whose chromatin is largely in the TSS and Gene Start states surprisingly, the other group of genes have a high proportion of their promoters in the SE1 chromatin state (we will denote these SE1+ promoters).

**Figure 4:**
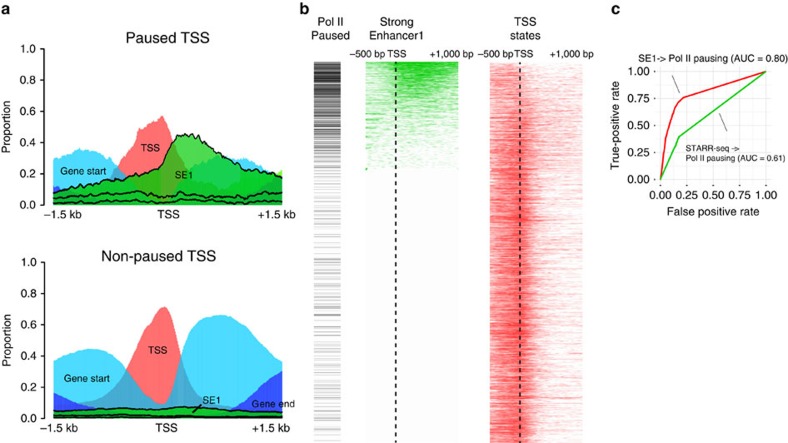
SE1 state is strongly predictive for Pol II pausing. (**a**) Pol II-paused active genes show distinct chromatin state spatial patterns with high proportions of SE1 state. The distributions of each chromatin state group that is enriched near the TSS (SE, Weak Enhancer, Gene End, Gene Start and TSS states) within −1.5 to 1.5 kb region are shown for both paused (top) and non-paused (bottom) active genes. The colour legend are as follows: SE states: green; Weak Enhancer states/Long Intron states: light green; TSS states: red; Gene Start states: light blue; Gene End states: blue. (**b**) Pol II-paused genes are highly enriched in genes with SE1+ promoter. Genes are ordered by proportion of SE1 state. Left panel shows paused genes by a black tick mark. Mid panel shows the presence of the SE1 state along the transcription initiation region of each gene (green). Right panel shows TSS states for the same genes (red). (**c**) SE1 proportion within −500 bp to +1 kb relative to TSS region is a strong predictor of Pol II pausing. TPR (true positive rate) shows the proportion of pausing genes above a given threshold of SE1 proportion within all pausing active genes. FPR (false positive rate) shows the proportion of non-pausing genes above a given threshold of SE1 proportion within all non-pausing active genes. Area under receiver operating curve (AUC) is also shown.

We found these SE1+ promoters to be strongly associated with Pol II pausing (Fig. 4a,b). Pol II pausing is a widespread phenomenon in both fly and mammals that has been discovered to take part in the rapid response to signals that initiate transcription in many processes, such as development and immediate early gene response in neurons^16^,^17^,^18^,^19^,^20^. Strikingly, 72% (532 out of 744) of active genes with paused promoters in S2 cells^17^ had SE1+ promoters, even though only 23% of all active genes in S2 cell were SE1+ (*P*-value<2.2e−16, Fisher's exact test). In fact, the proportion of the transcription initiation region of a gene that was in the SE1 state was predictive of Pol II pausing, with higher SE1 state proportions correlating to higher probability of Pol II pausing (area under curve (AUC)=0.801; Fig. 4c). Furthermore, in paused genes, the distance between TSS and the nearest SE1 element was significantly shorter than that of TSS and nearest SE1 elements of non-paused genes (*P*-value<2.2e−16, Wilcoxon's signed-rank test; Supplementary Fig. 11). Consistent with the important role Pol II pausing plays in development^16^,^18^,^20^, we found that many developmental process genes were strongly enriched among genes with SE1+ promoters (Supplementary Table 3).

The association between the SE chromatin state and Pol II pausing did not appear to be explained simply by enhancer activity, as STARR-seq enhancer elements alone were not sufficient to accurately predict pausing (Fig. 4c and Supplementary Fig. 11). In addition, to ensure that our results were independent of the Pol II binding data that was one of the inputs to our chromatin state identification model, we performed chromatin state identification without these data. We found that removing Pol II binding data had a negligible effect on predicting Pol II pausing promoters (71.2% active pausing promoters in S2 cells are SE1+; AUC=0.798), demonstrating that histone marks and chromatin protein alone provided sufficient information for achieving the pausing prediction performance.

We next examined the DNA sequence basis of SE1 by motif enrichment analysis. Interestingly, the GAGA motif is both very strongly and specifically enriched within the SE1 chromatin state (Supplementary Fig. 12). The GAGA motif was previously found to be overrepresented in promoters with Pol II pausing in the *Drosophila* embryo^21^,^22^ and is required for establishing Pol II pausing at the *hsp70* gene^23^,^24^. GAGA factor protein, which binds the GAGA motif and has been previously shown to bind paused genes and promote pausing by recruiting NELF^25^,^26^, was also present in SE1 (Fig. 2). GAGA factor protein is thus a potential factor in connecting the SE1 chromatin state with pausing. We also found several other motifs enriched in SE1, including the Pause Button motif, which has been previously associated with pausing^21^; however, no other enrichment was as specific to SE1 as the GAGA motif (Supplementary Fig. 12).

The discovery of the SE1 state challenges the conventional view that enhancer chromatin states are mutually exclusive with the active TSS state, and that the absence of the active TSS mark H3K4me3 acts as an enhancer mark. The SE1 chromatin state shows molecular signatures of both enhancers (such as H3K4me1 and H3K27ac) and active TSS (such as H3K4me3), and is located near or immediately downstream of the TSS. Our finding thus suggests that, in contrast to prior belief, enhancer states and TSS state marks do not antagonize each other. Instead, enhancer state can be an integrated component of the promoter and may play a role in determining its regulatory properties.

### Canonical spatial sequence of active gene chromatin states

**Figure 5:**
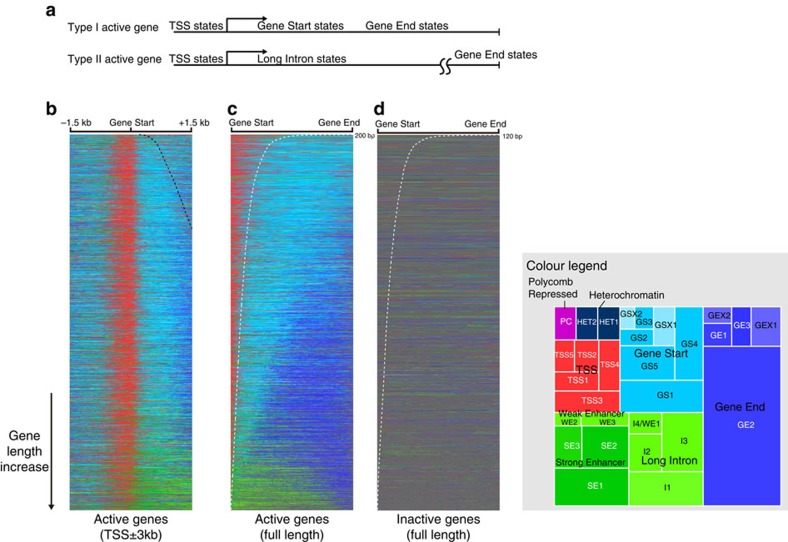
Spatial organization analysis reveals active gene chromatin state sequences. (**a**) Actively transcribed genes show a canonical chromatin state sequence anchored by distance to TSS and an alternative sequence is observed in a group of larger genes with high proportion of Long Intron states. (**b**,**c**) Canonical and alternative gene state sequences. Genes are sorted by length with shorter genes shown at the top. The −1.5 to +1.5 kb region relative to TSS for each gene is shown in **b** and each gene is scaled by its length to show the whole gene in **c**. Black dashed line in **b** shows gene length when it is within the 0–+1.5 kb range. White dashed line in **c** shows the same genomic length scale represented in different genes. Chromatin state groups are colour coded as in colour legend provided on the right. Size of a block in the colour legend is proportional to the total coverage of the state. For chromatin states: GS, Gene Start; GSX, Gene Start DCC; GE, Gene End; GEX, Gene End DCC; HET, Heterochromatin; I, Long Intron;PC, Polycomb Repressed; SE, Strong Enhancer; TSS, transcription start site; WE, Weak Enhancer. (**d**) Inactive genes are generally covered by the Ground state (grey) and show no dominant spatial organization pattern. As in **c**, each gene is scaled by its length, and the white dashed line indicates the fixed length scale represented in different gene.

Our analysis revealed that the spatial organization of chromatin states for the majority of active genes is uniformly anchored by an absolute distance to the TSS (Fig. 5a–c). Given this relatively stable structure, we used a hierarchical nomenclature to represent chromatin state groups by spatial order. From 5′ to 3′, we formed three groups of chromatin states: TSS, Gene Start and Gene End (Figs 1b, 2 and 3). Chromatin states within each group were also indexed following the 5′–3′ order, for example, within TSS states, TSS1 was the farthest 5′-state and TSS5 was the farthest 3′-one. We note that the Gene Start and Gene End state nomenclature do not imply that they necessarily appear at gene start or gene end, but that they are chromatin states whose positioning appear to be determined by an absolute distance to TSS regardless of gene length.

This canonical active gene chromatin state sequence, spanning from the TSS states to the Gene End states, had each state enriched at a typical distance relative to the TSS (Supplementary Fig. 13). This chromatin state sequence was also observable at the single gene level (Supplementary Fig. 14). Moreover, the chromatin state transition points, for example, TSS → Gene Start states and Gene Start → Gene End states, appeared at specific distances relative to the TSS and these distances are invariant to gene length (Fig. 5b,c). Although it is well known that the TSS and the 3′-region of active transcribed genes have distinct chromatin features, to our knowledge, this gene-length invariant property of active gene chromatin state spatial organization has not been previously reported. This observation supports the pivotal role of TSS in organizing the chromatin state of active genes.

The five most upstream TSS chromatin states spanning approximately −300 to approximately +300 bp of active TSS (Supplementary Fig. 13) were all marked by the previously known active TSS mark H3K4me3, as well as the absence of the H3K36me3 elongation mark and the H3K4me1 enhancer mark (Fig. 2). TSS states were highly enriched in DNase I hypersensitive sites, in line with their function involving TF binding (Fig. 3). Despite the apparent similarities of these TSS states, they did appear to possess distinct characteristics. One example is TSS3, which peaked at 200∼0 bp upstream of the TSS and showed exclusive and strong enrichment of many core promoter and TF motifs (Supplementary Fig. 12). TSS3 demonstrates our chromatin state discovery method's ability to finely dissect highly similar states with distinct properties.

Progressive change in chromatin factor compositions was observed moving down the sequence towards the 3′-end. Seven Gene Start states located 200–1,000 bp downstream of TSS (Supplementary Fig. 13) were enriched in both the active TSS mark H3K4me3/2 and the elongation mark H3K36me3 (Fig. 2), whereas five Gene End states that appear at over 1–1.5 kb downstream of the TSS (Supplementary Fig. 13) could be identified by the absence of H3K4me3 and the presence of elongation marks H3K79me1 and H3K36me3 (Fig. 2).

A special subset of the active gene states, Gene Start DCC1/2 and Gene End DCC1/2, was exclusively observed on the X-chromosome (Fig. 3). These states were uniquely marked by components of the dosage compensation complex (DCC) including MSL1 and the MSL1-catalysed histone mark H4K16ac (Fig. 2). The dosage-compensated X-chromosome active genes were upregulated in *Drosophila* males to compensate for differences in copy number between females and males^27^.

### Long Intron state marks alternative chromatin state sequence

In most active genes with large introns, we discovered an alternative type of spatial organization that is distinct from the canonical TSS→Gene Start→Gene End path (Fig. 5a–c). This alternative chromatin state sequence featured large domains of Long Intron states, which is a group of enhancer-like states with no or little enrichment of active enhancers. Long Intron states were unique for their enrichment of long (>1 kb), but not short, introns in active genes (Fig. 3) and they were distinct from other active gene states in that they formed large continuous chromatin state domains on the chromosome (Supplementary Data 1). Long Intron states differed from other enhancer-like states in many transcription elongation-associated chromatin marks, such as enrichment of H3K79me2 and H2Bubi marks (89% of Long Intron states regions had H3K79me2 and only 21.8% of non-Long Intron enhancer-like states regions had H3K79me2; similar patterns for H2Bubi were observed). In general, Long Intron states shared many chromatin marks with enhancer states (all Long Intron states possessed both of the commonly used enhancer marks H3K4me1 and H3K27ac, except Long Intron 1, which was only enriched in H3K4me1, but not H3K27ac) but have not been clearly distinguished before.

*D. melanogaster* has a compact genome with far shorter intergenic regions than the human genome^28^. However, some genes harbour very large introns spanning tens of kilobases. Evolutionary evidence suggests selective pressure in preserving these introns^28^ and this may be explained by *cis*-regulatory elements within long introns. Indeed, 55.6% of STARR-seq enhancers were located within introns^10^. We propose that the Long Intron chromatin states may protect the activity of regulatory elements such as enhancers that are located within long introns. In line with this hypothesis, we observed that both the presence and the adjusted proportion of SE states (the proportion of SE states in regions excluding Long Intron states) were positively correlated with the proportion of Long Intron states in a gene (Supplementary Fig. 15). In contrast, Gene Start and Gene End states, which would appear in the position of Long Intron states in the canonical active gene chromatin state sequence, negatively correlated with SE states (Supplementary Fig. 15), suggesting that the Long Intron state may antagonize the canonical active gene chromatin state sequence and protect intronic enhancers.

## Discussion

The large-scale chromatin profiling efforts present both exciting opportunities and challenges in understanding the system of chromatin and transcriptional regulation. Here we introduced effective methods for extracting the chromatin state information from chromatin profiles, characterized the structure of the identified chromatin state landscape and performed systematic analyses pursuing the function of the discovered chromatin states. Our chromatin state discovery method based on chromatin factor dependencies allowed comprehensive chromatin state identification leveraging associations among chromatin factors identified from the data, without making any assumptions of spatial dependencies of chromatin states. Our work provides a new perspective on the regulatory roles and mechanisms of chromatin organization, including revealing the functional and compositional differences of enhancer-like chromatin states with a more specific detection of active enhancers, a novel association between a specific chromatin state (SE1 state) and Pol II pausing, and the characterization of the active gene chromatin state sequence with both a canonical sequence organized by distance to the TSS and an alternative chromatin state sequence featuring Long Intron states.

Our finding of the diversity of enhancer-like states enabled us to identify new marks for more specific detection of active enhancers. Histone marks H3K27ac, H3K4me1, depletion of H3K4me3 and occasionally H3K9ac have been widely used as enhancer marks in different organisms for identifying active enhancers^29^,^30^,^31^,^32^, but our results suggest that these marks are insufficient for pinning down states with active enhancer activity. Additional marks, such as the absence of H3K36me1 or H3K27me1, are required to distinguish SE states from other enhancer-like states. Furthermore, the H3K9ac mark can only be used for specifically detecting the SE1 state, as this mark is not present in other SE states. In addition, unlike other enhancer-like states, the SE1 state is not depleted of H3K4me3, which was previously considered an enhancer mark.

The chromatin states we characterized also contribute to identifying novel chromatin functions. To our knowledge, the strong positive association between chromatin state and Pol II pausing has not been previously reported and our finding that SE1 marks a group of genes highly enriched in the Pol II paused genes suggests a novel link between chromatin state, enhancers and transcription regulation by Pol II pausing. This finding may contribute to further elucidation of the mechanism of Pol II pausing.

Despite significant progress, further work is necessary to understand the mechanism of the discovered associations between chromatin factors and chromatin states. For example, H3K36me1 and H3K27me1, which distinguish SE states from other enhancer-like states, have not been associated with enhancer activity before and it is of significant interest whether they are directly involved in suppression of enhancer activity. Furthermore, the current availability of chromatin profiling data limits our analysis to a single cell type. We expect that as the experimental barriers for scaling up chromatin profiling experiments get resolved, our analytical framework will be useful for analysing and understanding cell-type specificity and the dynamics of chromatin states.

## Methods

### Chromatin state identification

Chromatin states were identified by an optimization-based algorithm based on the chromatin code energy landscape given by a probabilistic model. Our probabilistic model of chromatin codes is a maximum entropy model with group *L*_1_ regularized pairwise and third-order interactions estimated from modENCODE chromatin profiling data for S2-DRSC cell^9^. Specifically:


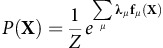


The energy of a chromatin code is calculated as 
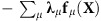
. Thus, the energy of a chromatin code is always equal to the negative log probability plus a constant. **X** is a chromatin factor pattern encoded as a binary vector of length 73, with each entry indicating the presence of one chromatin factor (0 indicates the absence and 1 indicates the presence, with the exceptions that for H3K23ac-, H1-, H3- and H4-, 0 indicates the presence and 1 indicates the absence). Each **f**_*μ*_(**X**) is a binary feature representing the presence of single chromatin factor or co-occurrence of a pair or triplet of chromatin factors, and ***λ***_*μ*_ is the corresponding self, pairwise and triplet interaction scores that will be learned from chromatin profile data. *μ* is the index for the features of **X**. The 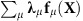 term in our model specifically are decomposed to self, pairwise and triplet interaction terms, which can be written as


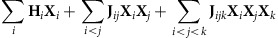


where *i*, *j*, *k*∈{1, 2 … 73} are indices of chromatin factors. **H**_*i*_, **J**_*ij*_, **J**_*ijk*_ corresponds to ***λ***_*μ*_ and **X**_*i*_, **X**_*ij*_, **X**_*ijk*_ corresponds to **f**_*μ*_(**X**). *Z* is the normalization constant that ensures that the sum of probabilities of all possible **X** equals to 1.

The model was trained with modENCODE chromatin data for S2-DRSC cell as described previously^9^ and the model can be downloaded with the code at https://bitbucket.org/jzthree/chromatin-state.

To classify patterns to states, we used a Hopfield network-like dynamical system, which can also be interpreted as a steepest descent optimization algorithm to navigate the landscape by finding the local minima of the valley a chromatin code resides in. The algorithm iteratively selects the lowest energy pattern in the neighbourhood of the selected pattern until reaching the local minimum (Supplementary Note 3). The neighbourhood of a chromatin code is defined to be all codes that differ from that code at only one chromatin factor. The local minimum that this algorithm finally reaches identifies the valley that a pattern resides in. This algorithm for finding the local minimum of the energy landscape can be viewed as a variant of the algorithm for recalling the attractors in a Hopfield network^33^.

When applied to the *D. melanogaster* modENCODE chromatin profiling data, the algorithm described above identified 30 major, most frequent chromatin states that covered 94% of the genomic regions covered by the chromatin profiles data. We then further integrated the remaining mini states with the major states that they were highly connected to by iteratively combining the least frequent mini state with another state, which had the highest spatial connectivity score to that mini state, until all mini states were combined with the top 30 states (Supplementary Note 3). The final chromatin state compositions were highly similar to the top 30 chromatin states before combination (Supplementary Fig. 16). The chromatin state annotations generated in this study are provided in Supplementary Data 1.

### Data processing

Chromatin ChIP tilling array data were normalized to probe *t*-values as described in Zhou *et al.*^9^ (raw CEL file downloaded from ftp://data.modencode.org/ in July 2012, see Supplementary Data 5 for complete list). Probes that mapped more than once within any 1-kb region were removed before the analysis. After computing probe *t*-values, we applied a moving window of 200 bp with step size 50 bp. Genomic bins with lower than 100 bp probe sequence coverage were not used for further analysis throughout the manuscript. Probe *t*-values within a bin were averaged and then binarized as described in Zhou *et al.*^9^ The resulting data matrix was of size 73 chromatin factors by 2,141,361 bins. The processed data are available for download at https://bitbucket.org/jzthree/chromatin-state. *D. melanogaster* Apr. 2006 (BDGP R5/dm3) genome assembly is used throughout this study.

### Spatial organization

The spatial connectivity score of chromatin states measures the tendency of two chromatin states being adjacent with each other in the genome. Specifically, spatial connectivity score was computed as the log ratio of the frequency of observed chromatin state pairs in adjacent non-overlapping bins and the expected frequency by chance computed from chromatin state proportions.


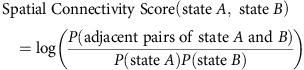


The *P*(statement) notation stands for proportion of genomic regions satisfying the condition described by the statement.

### Experimentally annotated genomic element enrichment analysis

To identify which genomic elements were overrepresented in each chromatin state, we performed systematic enrichment analysis. We calculated the enrichment score as the log odds of the proportion of a chromatin state overlapping with a genomic element type, normalized by subtracting the expected log odds assuming independence between the chromatin state and the genomic element type, or 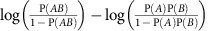. *P*(*AB*) indicates the proportion of overlapping region between the chromatin state and the genomic element type. *P*(*A*) and *P*(*B*) indicate the proportion of individual state and genomic element type, respectively. Genomic elements used were obtained from several sources. Gene, intron, exon, 5′-untranslated region, 3′-untranslated regionand transposon coordinates were obtained from Flybase r5.46 annotations^12^. Curated *cis*-regulatory modules and TF-binding sites were exported from the REDfly database (downloaded on 12 February 2012)^11^. Active enhancer elements in S2 cell were experimentally determined by STARR-seq^10^. Active and repressed genes were classified based on the RNA-seq data of S2-DRSC cell^10^. As the distribution of log reads per kilobase of transcript per million mapped reads (RPKM) is bimodal and we refer to the higher mode as active and the lower mode as inactive genes^34^. We thus fit a mixture of two univariate Gaussians model to set the threshold for classifying active and inactive genes based on the log RPKM, with a gene classified as active if the probability that the signal coming from the high expression component is >0.9 (RPKM>2.101) or inactive if the probability that the signal coming from the low expression component is >0.9 (RPKM<0.435).

### Association between SE1 and Pol II pausing

To study the association between the SE1 state and Pol II pausing, we annotate the Pol II paused promoter based on Muse *et al.*^17^. SE1+ promoters were defined based on the proportion of the −500 to +1,000 bp relative to TSS region that is in the SE1. The threshold, 4.4%, was determined by maximizing 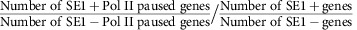. It is notewothy that only the active genes determined from RNA-seq data as described above were classified as SE1+ or SE1−. The majority (92%) of the Pol II-paused genes are active genes. Receiver-operating characteristic curves were computed for evaluating the prediction performance of using the percentages of SE1 state, as well as STARR-seq enhancers, in the −500 to +1,000 bp region to predict paused genes among all active genes. AUC was calculated for quantifying receiver-operating characteristic-based prediction performance. Gene Ontology term enrichment was calculated for all SE1+ genes across all biological process terms^35^.

To evaluate the effect of removing the Pol II data for predicting paused genes, we replaced the Pol II binding data with the imputed Pol II binding profile. The imputed Pol II binding profile was based on conditional probabilities of Pol II conditioned on other chromatin factor profiles given by the maximum entropy chromatin model as described in ref. 9. Genomic bins with conditional probability of Pol II presence >0.5 were imputed as Pol II positive. Then the same chromatin state identification procedures and analyses were performed on the imputed data and compared with original results.

### Code Availability

The code is available at https://bitbucket.org/jzthree/chromatin-state.

## Additional information

**How to cite this article:** Zhou, J. & Troyanskaya, O. G. Probabilistic modelling of chromatin code landscape reveals functional diversity of enhancer-like chromatin states. *Nat. Commun.* 7:10528 doi: 10.1038/ncomms10528 (2016).

## Supplementary Material

Supplementary Information Supplementary Figures 1-16, Supplementary Tables 1-3, Supplementary Notes 1-3, Supplementary Methods and Supplementary References

Supplementary Data 1 The 30-chromatin state annotation tracks for Drosophila melanogaster DRSC-S2 cell.

Supplementary Data 2 Chromatin factor compositions of each chromatin state.

Supplementary Data 3 Enrichment of functional genomic elements in each chromatin state.

Supplementary Data 4 Effects of positive and negative perturbations of single chromatin factors on chromatin state identity.

Supplementary Data 5 List of raw ChIP-chip data files from modENCODE used in this study
