# Supplementary material for: Probabilistic modelling of chromatin code landscape reveals functional diversity of enhancer-like chromatin states
Source: Nat Commun. 2016 Feb 4;7:10528. doi: 10.1038/ncomms10528 (PMC4742914; doi:10.1038/ncomms10528)
Supplement: Supplementary Information — Supplementary Figures 1-16, Supplementary Tables 1-3, Supplementary Notes 1-3, Supplementary Methods and Supplementary References [file ncomms10528-s1.pdf]

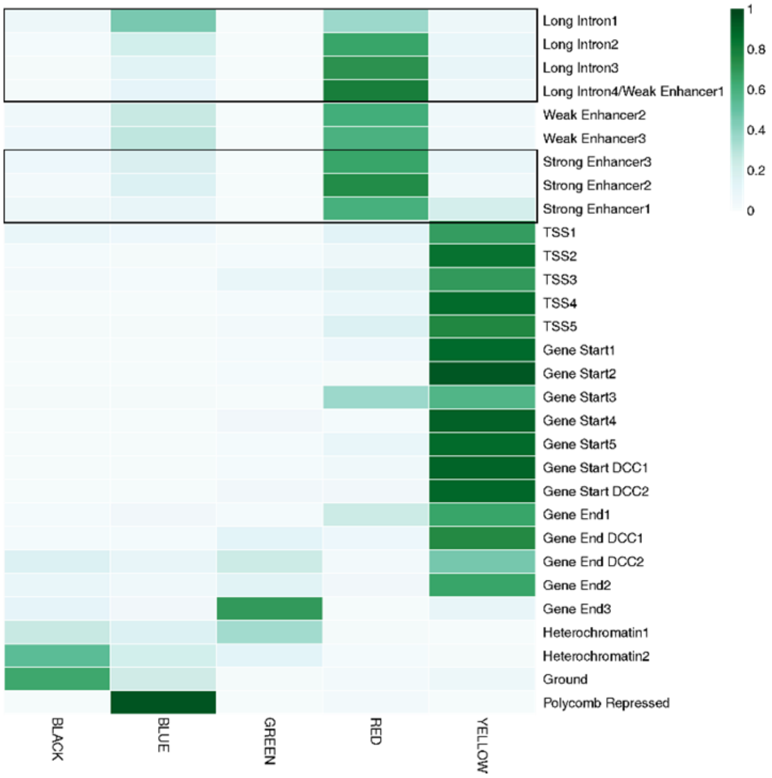


**Supplementary Figure 1. Comparison with the 5 chromatin states from Fillion et al. for Kc167 cell.**

For each of the 30 states we identified, we showed the proportion of genomic regions in that state overlapping with each state in the Fillion et al. 5 chromatin state system for Kc167 cell[1]. Notable differences include that Strong Enhancer, Weak Enhancer, and Long Intron states were not distinguished in the 5 states in Fillion et al., and Strong Enhancer 1 is not distinguished from other enhancer-like states (See **Supplementary Note 1** for details).

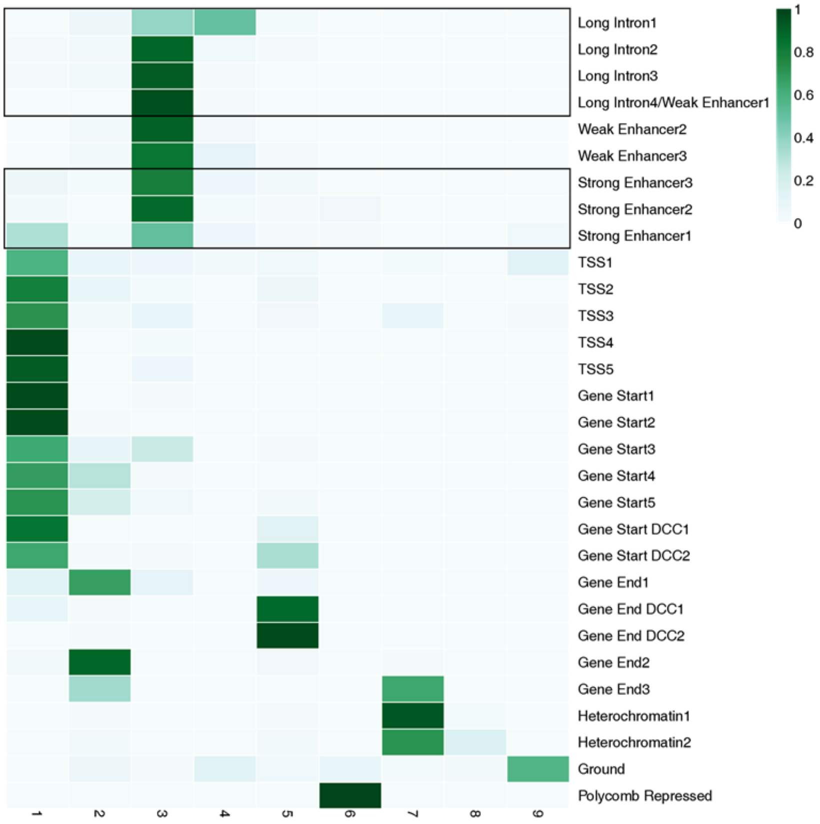


**Supplementary Figure 2. Comparison with the 9 chromatin states from Kharchenko et al. for S2 cell.**

For each of the 30 states we identified, we showed the proportion of genomic regions in that state overlapping with each state in the Kharchenko et al. 9 chromatin state system[2]. Notable differences include that Strong Enhancer, Weak Enhancer, and Long Intron states were not distinguished in the 9 states annotation in Kharchenko et al., and Strong Enhancer 1 is not distinguished from other enhancer-like states (See **Supplementary Note 1** for details).

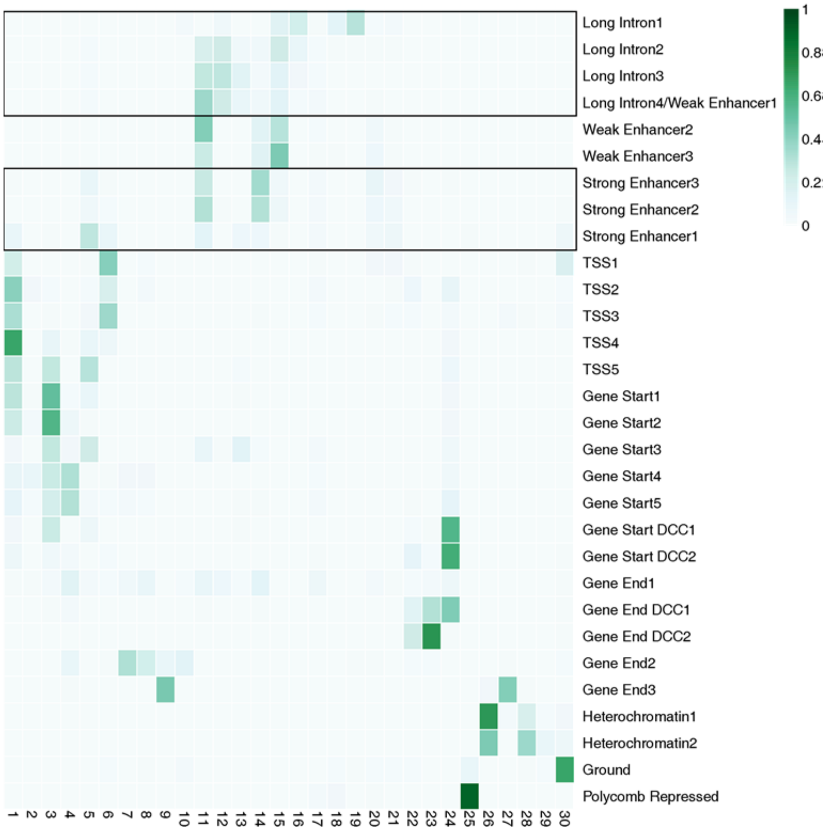


**Supplementary Figure 3. Comparison with the 30 chromatin states from Kharchenko et al. for S2 cell.**

For each of the 30 states we identified, we showed the proportion of genomic regions in that state overlapping with each state in the Kharchenko et al. 30 chromatin state system[2]. Notable differences include that Strong Enhancer, Weak Enhancer, and Long Intron states were not distinguished in the 30 states annotation in Kharchenko et al., and Strong Enhancer 1 is not distinguished from other canonical active gene sequence states and enhancer-like states (See **Supplementary Note 1** for details).

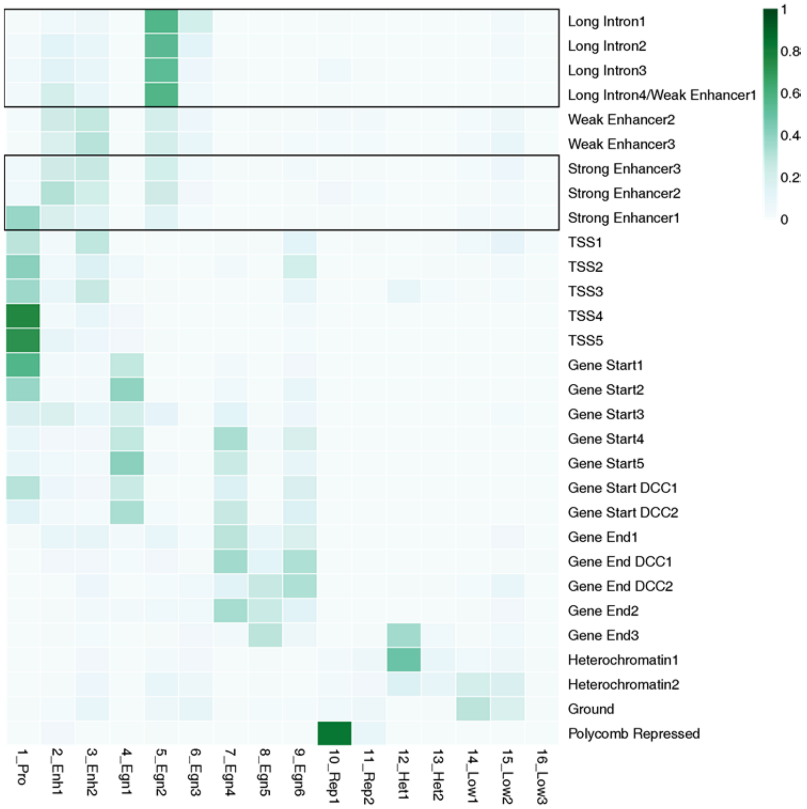


**Supplementary Figure 4. Comparison with the 16 chromatin states from Ho et al. for late embryo.**

For each of the 30 states we identified, we showed the proportion of genomic regions in that state overlapping with each state in the Ho et al. chromatin state system for late embryo[3]. Notable differences include that Strong Enhancer and Weak Enhancer states were not distinguished in the 16 states in Ho et al. for late embryo, and Strong Enhancer 1 is not distinguished from other canonical active gene sequence states and enhancer-like states. Ho et al. identified a chromatin state that corresponds to our Long Intron states, but it was described by Ho et al. as a “transcription 5’ 2” state and the specific enrichment in Long Intron was not discussed (See **Supplementary Note 1** for details).

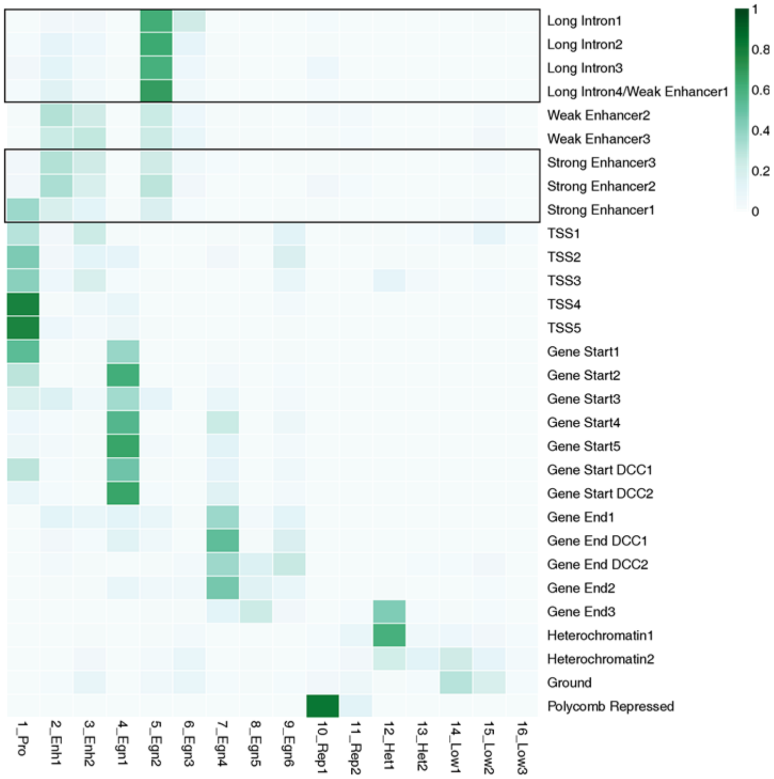


**Supplementary Figure 5. Comparison with the 16 chromatin states from Ho et al. for third instar larvae.**

For each of the 30 states we identified, we showed the proportion of genomic regions in that state overlapping with each state in the Ho et al. chromatin state system for third instar larvae[3]. Notable differences include that Strong Enhancer and Weak Enhancer states were not distinguished in the 16 states in Ho et al. for third instar larvae, and Strong Enhancer 1 is not distinguished from other canonical active gene sequence states and enhancer-like states. Ho et al. indeed identified a chromatin state that corresponds to Long Intron states, but it was described by Ho et al. as a "transcription 5' 2" state and the specific enrichment in Long Intron was not discovered (See **Supplementary Note 1** for details).

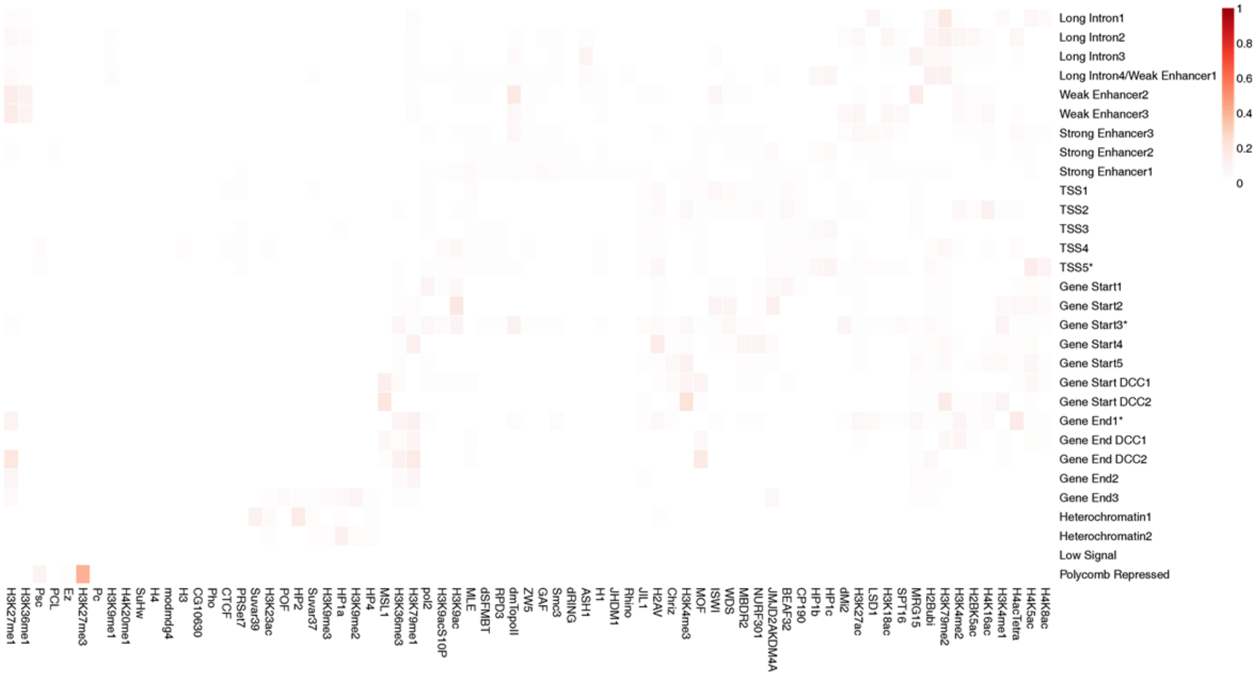


**Supplementary Figure 6. Chromatin state identification is robust to single chromatin factor removal.**

For each chromatin state, the proportion of genomic regions altered to a different chromatin state by removing each chromatin factor data is shown (See **Supplementary Note 2** for details).

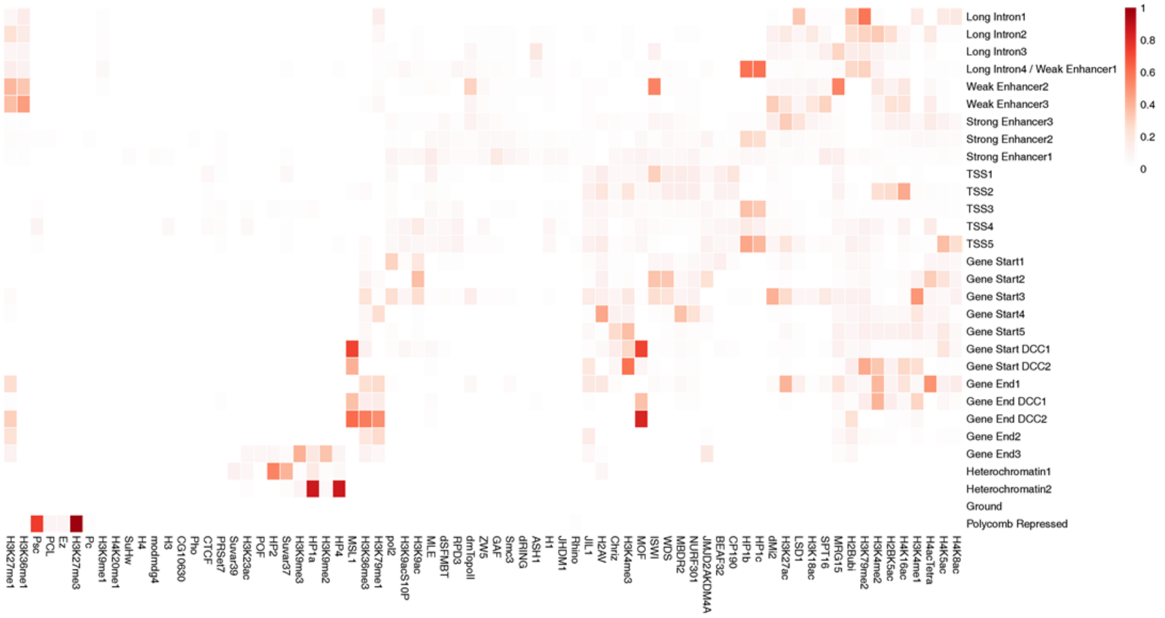


**Supplementary Figure 7. Negative perturbation effects of chromatin factors on each chromatin state.**

The heatmap shows proportion of regions of each chromatin state changed to another chromatin state when a chromatin factor is perturbed (from present to absent). Only genomic regions in which the chromatin factor is present are considered in the computation (See **Supplementary Note 2** for details).

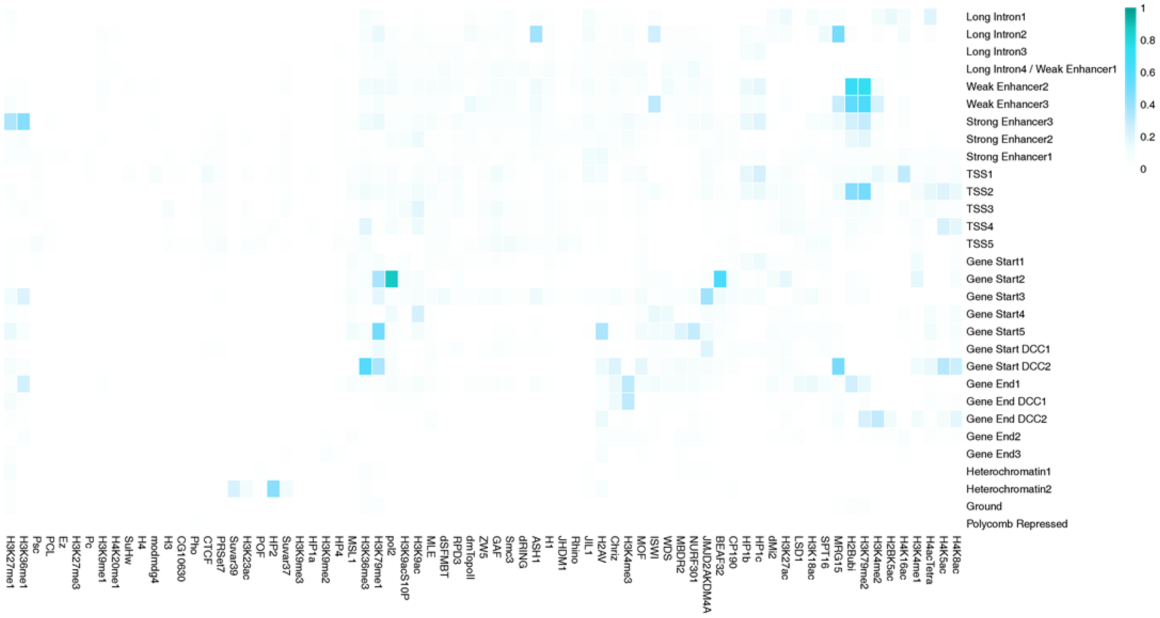


**Supplementary Figure 8. Positive perturbation effects of chromatin factors on each chromatin state.**

The heatmap shows proportion of regions of each chromatin state changed to another chromatin state when a chromatin factor is perturbed (from absent to present). Only genomic regions in which the chromatin factor is absent are considered in the computation (See **Supplementary Note 2** for details).

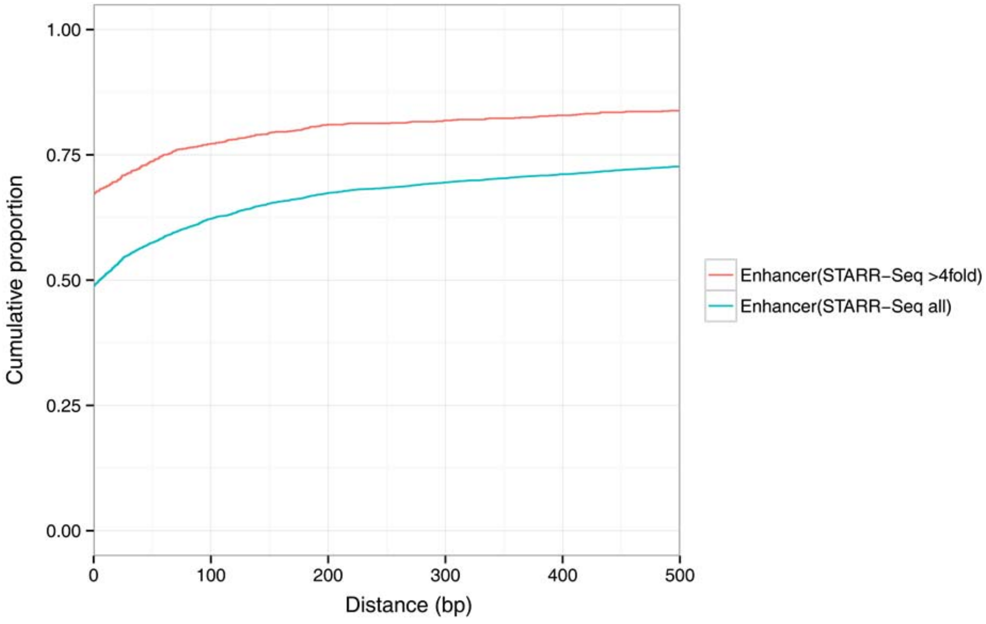


**Supplementary Figure 9. Strong Enhancer states co-localize with the majority of active strong enhancers detected by STARR-seq.**

Cumulative proportions (y-axis) located within certain distances (x-axis) from Strong Enhancer states for strong STARR-seq enhancers with >4 fold reporter expression change (red) and all STARR-seq enhancers (blue) are shown.

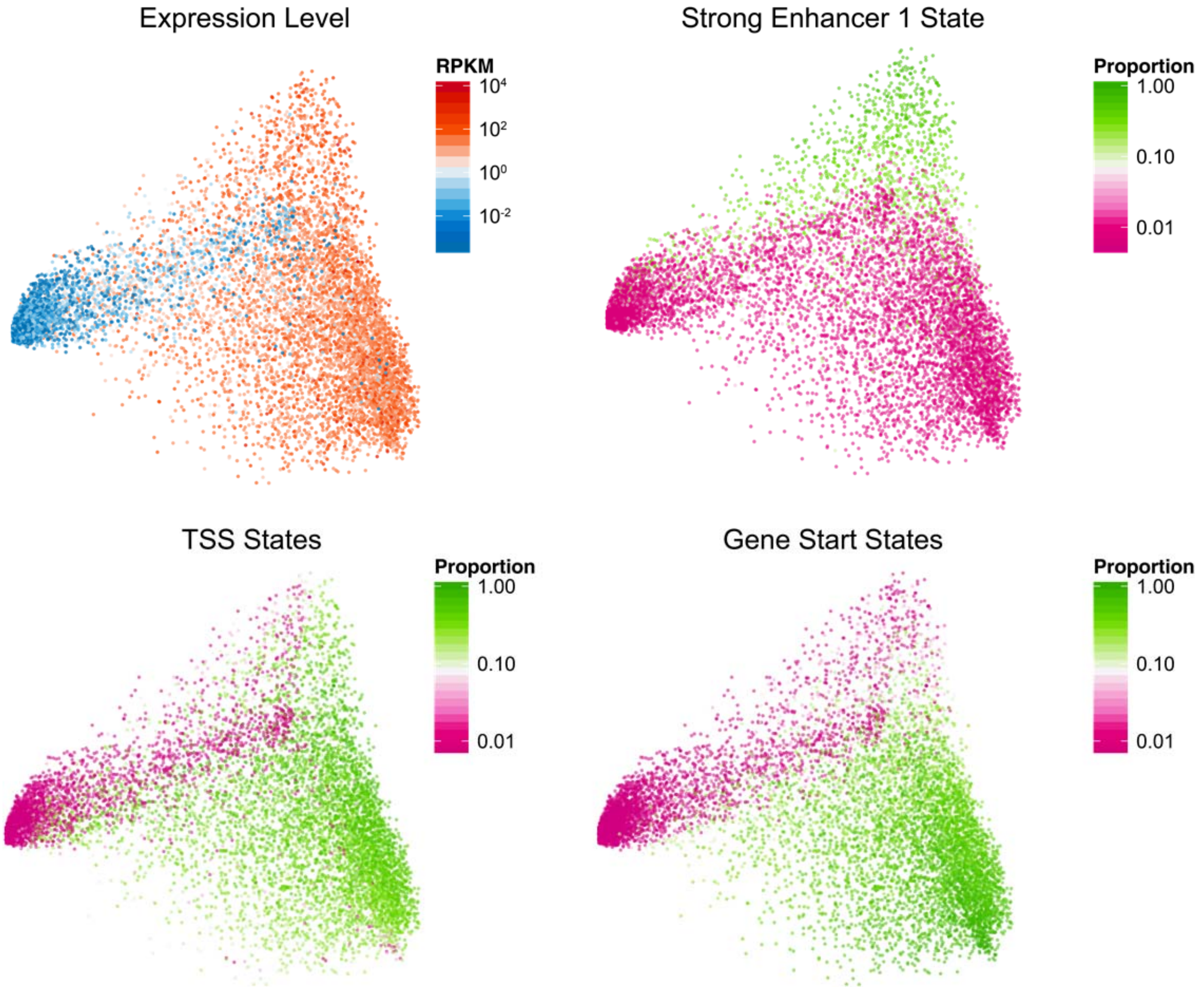


**Supplementary Figure 10. A distinct category of active gene chromatin state is marked by high proportion of SE1.**

Each gene is represented as a dot in the map. Projection of the chromatin state sequences within -500bp to +1000bp region relative TSS to 2-dimensional space is computed by multi-dimensional scaling (MDS), using dissimilarities between chromatin state sequences as input. RNA expression quantified by RPKM (reads per kbp per million) and chromatin state proportions of each gene's transcription initiation region are shown by color of dots in separate panels.

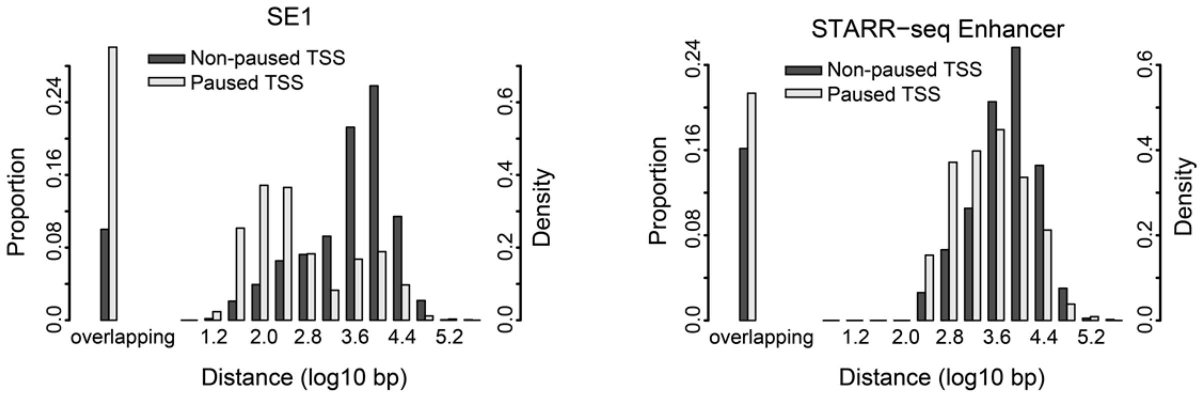


**Supplementary Figure 11. Pol II paused transcription start sites show strong preferential localization within or near SE1 state.**

The plots show distributions of distances between paused (black) or non-paused (white) active TSS and nearest SE1 (left panel) or STARR-seq enhancers (right panel). The proportions of paused or non-paused TSS overlapping with SE1 or STARR-seq enhancer are shown in the left of each panel. Paused TSS overall locate significantly closer to SE1 than non-paused TSS, while the difference in distance distribution is much smaller for STARR-seq enhancers.

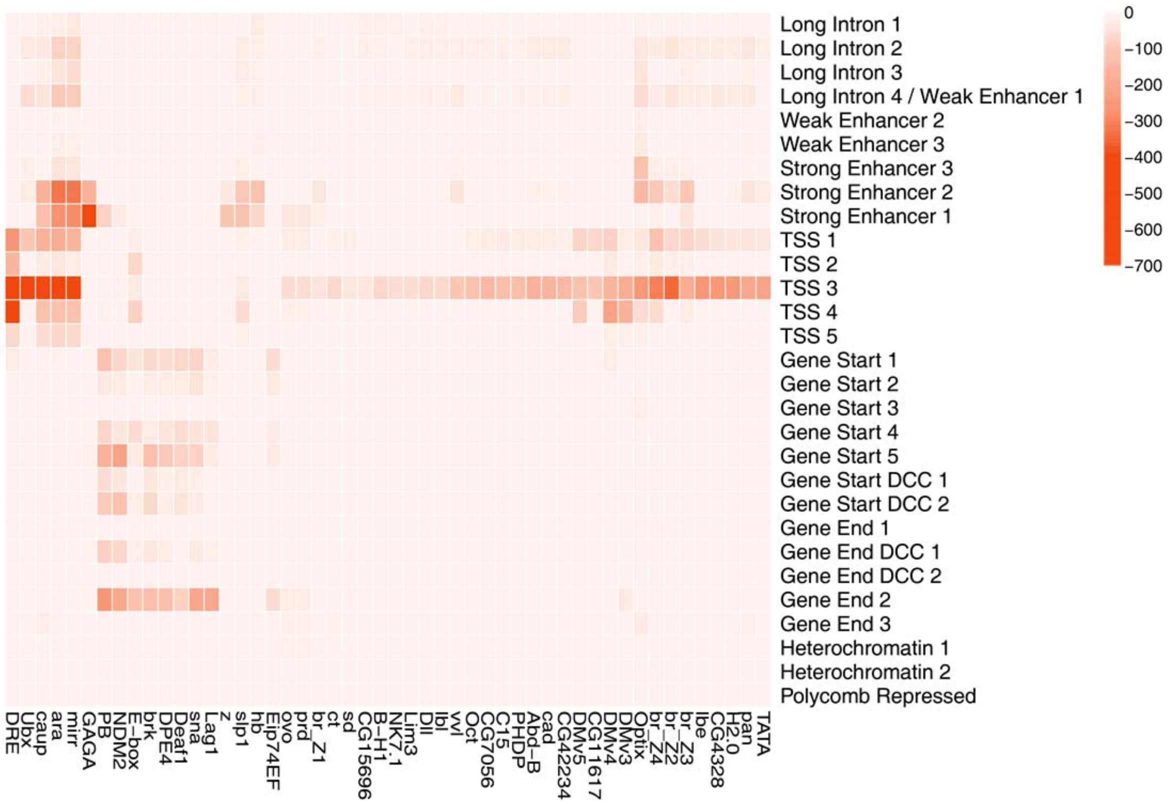


**Supplementary Figure 12. Enrichment of TF binding motifs and core promoter motifs.**

Log of p-values for enrichment are shown for each motif (x-axis) and each chromatin state (y-axis).

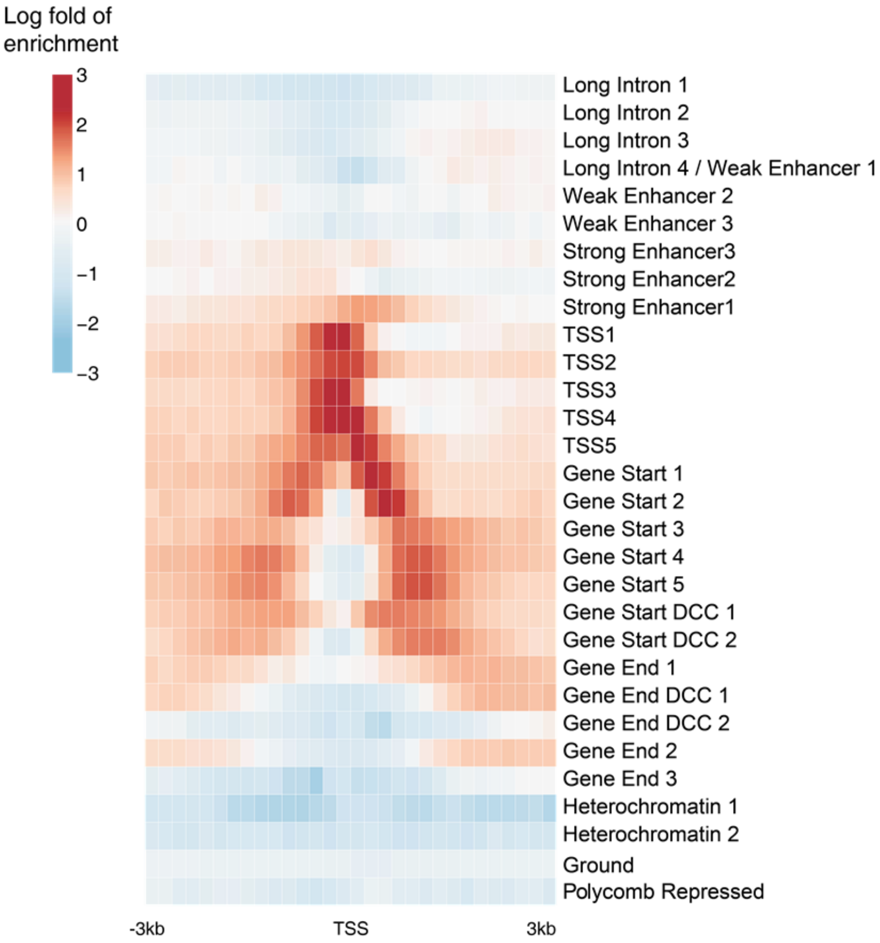


**Supplementary Figure 13. Chromatin states show position-specific localization relative to TSS.**

Log fold of enrichment scores are computed as the log odds of the percentage coverage by each chromatin state at a specific region relative to TSS subtracted by the log odds expected if chromatin states are randomly positioned.

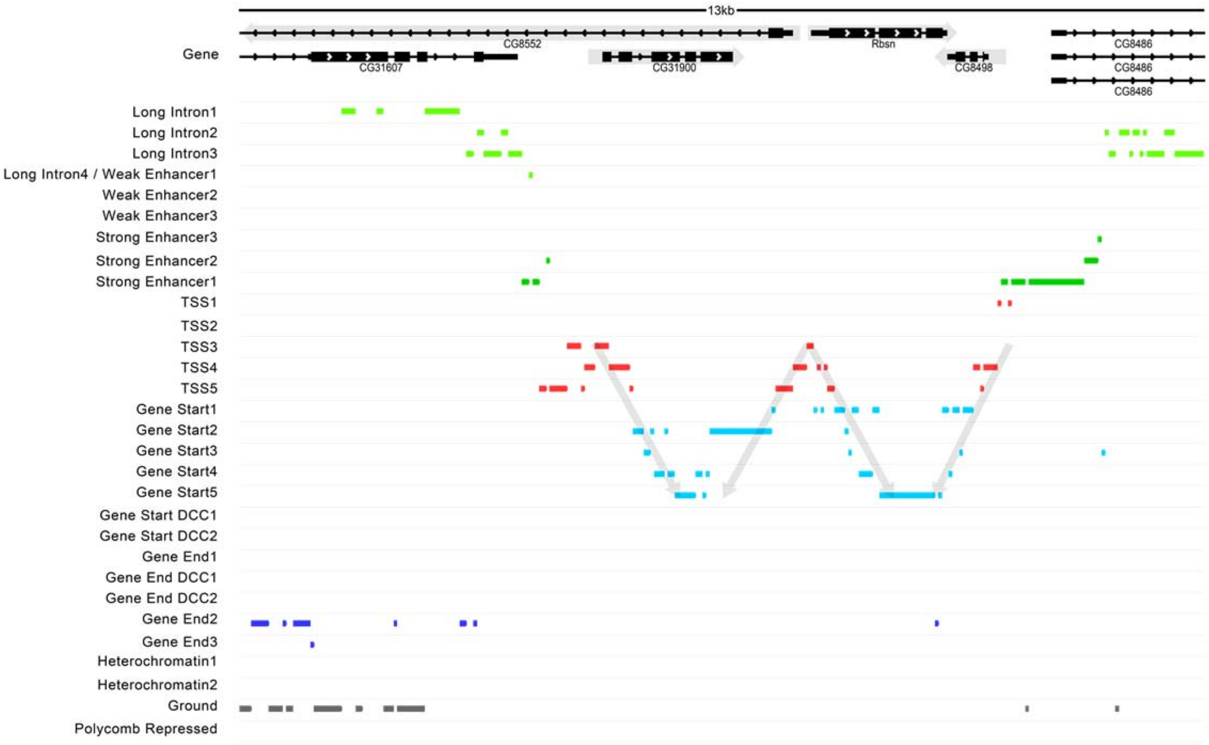


**Supplementary Figure 14. Chromatin state sequence of active genes at single gene level.**

Genome browser style view shows chromatin state annotation of a 13kb genomic region as a demonstration. The gray arrows show that the directions of transcription align with the chromatin state sequences for four genes.

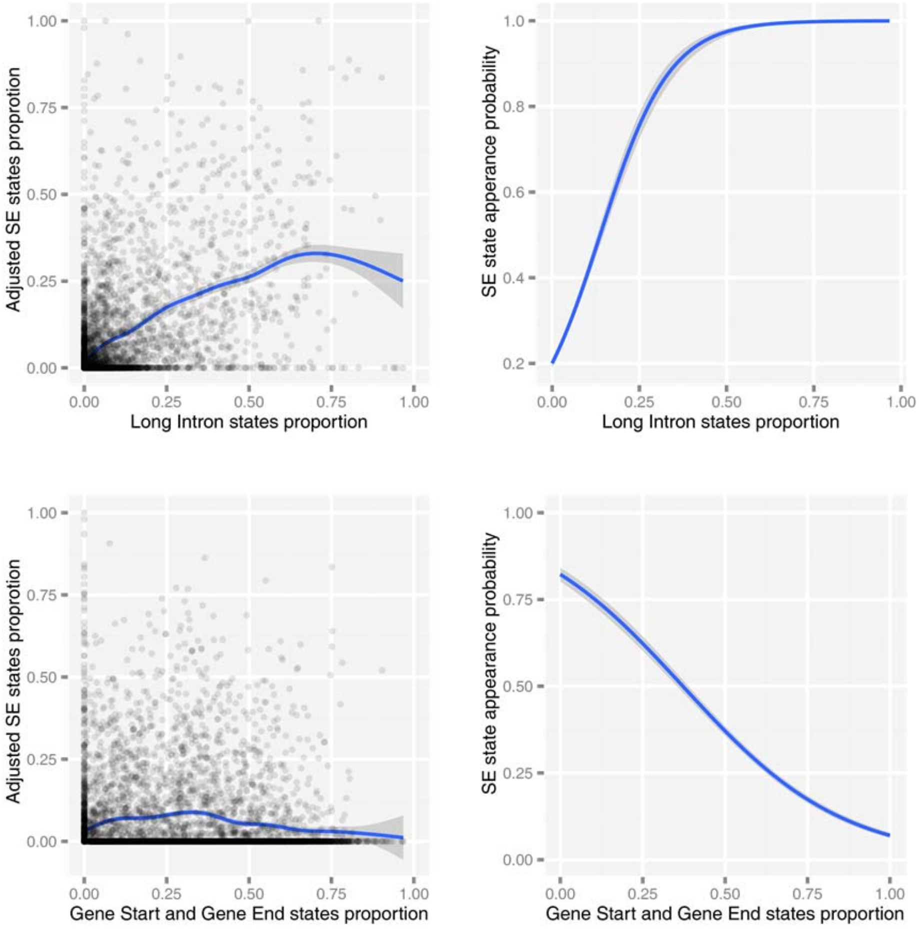


**Supplementary Figure 15. Proportion of Long Intron states but not Gene Start and Gene Ends states positively correlates with appearance and proportion of Strong Enhancer states.**

Proportion of Long Intron states in a gene positively correlates with both adjusted proportion (top left) and appearance probability (top right) of Strong Enhancer (SE) states, while proportion of Gene Start and Gene End states, which would appear in the position of Long Intron states in the canonical gene path, negatively correlate with both (bottom left; bottom right). Adjusted proportion of SE states is computed as the SE states proportion divided by one minus the proportion of states on x-axis. The fitted curves and 95% confidence intervals showing the trends are estimated with generalized additive models for Gaussian (for SE states proportion) and binomial (for SE states appearance probability) families.

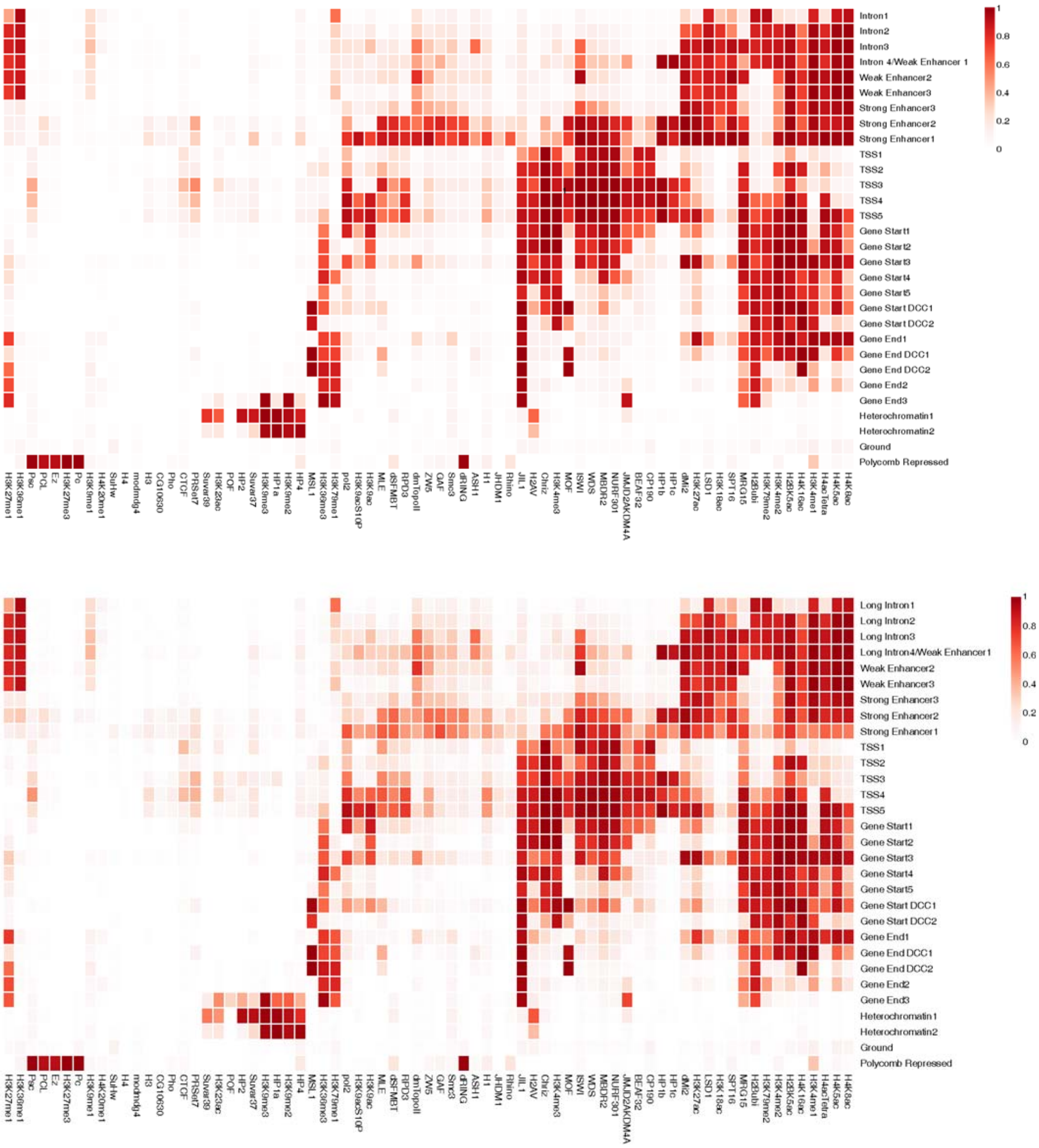


**Supplementary Figure 16. Comparison of the chromatin factor compositions of the top 30 states prior to iterative combination and the final chromatin states.**

The chromatin factor compositions of the top 30 most frequent states prior (top panel) and after (bottom panel) iterative combination to 30 states are shown. The final chromatin states are very close to the top 30 chromatin states prior to iterative combination.

**Supplementary Table 1. Nomenclature of the 30 chromatin states**

| | Chromatin state name | Acronym | Functional evidences |
|---|---|---|---|
| ‘Enhancer-like’ states | Long Intron1 | I1 | Strong enrichment in Long Introns |
| | Long Intron2 | I2 | |
| | Long Intron3 | I3 | |
| | Long Intron4 / Weak Enhancer1 | I4/WE1 | Strong enrichment in Long Introns / Weak enrichment for STARR-seq enhancers |
| | Weak Enhancer2 | WE2 | Weak enrichment for STARR-seq enhancers |
| | Weak Enhancer3 | WE3 | |
| | Strong Enhancer3 | SE3 | Strong enrichment for STARR-seq enhancers<br>(SE1 is located near TSS and strongly associated with Pol II pausing.) |
| | Strong Enhancer2 | SE2 | |
| | Strong Enhancer1 | SE1 | |
| Canonical active gene sequence<br>(indexed in 5’ to 3’ order) | TSS1 | TSS1 | Strong enrichment for active TSS<br>(TSS3 is specifically enriched in core promotor motifs.) |
| | TSS2 | TSS2 | |
| | TSS3 | TSS3 | |
| | TSS4 | TSS4 | |
| | TSS5 | TSS5 | |
| | Gene Start1 | GS1 | Strong enrichment for active genes and located closely downstream (+200 to +1000bp) of TSS |
| | Gene Start2 | GS2 | |
| | Gene Start3 | GS3 | |
| | Gene Start4 | GS4 | |
| | Gene Start5 | GS5 | |
| | Gene Start DCC1 | GSX1 | Strong enrichment for X-chromosome active gene and located closely downstream (+200 to +1000bp) of TSS |
| | Gene Start DCC2 | GSX2 | |
| | Gene End DCC1 | GEX1 | Strong enrichment for X-chromosome active gene and located distantly downstream (+1500bp to 3’ end) of |
| | Gene End DCC2 | GEX2 | |

| | | | |
|---|---|---|---|
| | | | TSS |
| | Gene End1 | GE1 | Strong enrichment for active gene and located distantly downstream (+1500bp to 3' end) of TSS.<br><br>(GE3 is H3K9me3 positive and associated actively transcribed genes in near heterochromatin region.) |
| | Gene End2 | GE2 | |
| | Gene End3 | GE3 | |
| Inactive gene states | Heterochromatin1 | HET1 | Known heterochromatin marks.<br><br>Strong enrichment for transposons. |
| | Heterochromatin2 | HET2 | |
| | Ground | G | No enrichment for any chromatin factor |
| | Polycomb Repressed | PC | Known polycomb repressive complex component and associated marks. Strong enrichment for regulatory elements (mostly developmental). |

**Supplementary Table 2. Genome coverage of each chromatin state.**

| Chromatin state | Coverage (bp) |
|---|---|
| Long Intron1 | 1681400 |
| Long Intron2 | 818100 |
| Long Intron3 | 1616050 |
| Long Intron4/Weak Enhancer1 | 466850 |
| Weak Enhancer2 | 235400 |
| Weak Enhancer3 | 420550 |
| Strong Enhancer3 | 762400 |
| Strong Enhancer2 | 1309650 |
| Strong Enhancer1 | 1851750 |
| TSS1 | 559900 |
| TSS2 | 511350 |
| TSS3 | 926850 |
| TSS4 | 712750 |
| TSS5 | 421550 |
| Gene Start1 | 1770600 |
| Gene Start2 | 368900 |
| Gene Start3 | 280150 |
| Gene Start4 | 1377050 |
| Gene Start5 | 1248650 |
| Gene Start DCC1 | 546600 |
| Gene Start DCC2 | 216400 |
| Gene End1 | 430850 |
| Gene End DCC1 | 777200 |
| Gene End DCC2 | 311900 |
| Gene End2 | 8177900 |
| Gene End3 | 508050 |
| Heterochromatin1 | 475000 |
| Heterochromatin2 | 485550 |
| Ground | 77325150 |
| Polycomb Repressed | 473550 |

**Supplementary Table 3. Top enriched gene ontology biological process terms for SE1+ genes.**

| GO term name | P-value | Benjamini–Hochberg FDR |
|---|---|---|
| system development | 3.12E-37 | 7.47E-34 |
| multicellular organismal development | 7.40E-36 | 8.85E-33 |
| anatomical structure development | 5.61E-35 | 4.48E-32 |
| organ development | 3.80E-34 | 2.27E-31 |
| developmental process | 2.59E-32 | 1.24E-29 |
| anatomical structure morphogenesis | 8.79E-32 | 3.51E-29 |
| biological regulation | 8.18E-30 | 2.80E-27 |
| tissue development | 4.25E-28 | 1.27E-25 |
| nervous system development | 6.24E-28 | 1.66E-25 |
| cellular developmental process | 1.84E-27 | 4.41E-25 |
| cell differentiation | 2.33E-27 | 5.07E-25 |
| regulation of biological process | 3.88E-26 | 7.74E-24 |
| generation of neurons | 3.81E-25 | 7.02E-23 |
| neurogenesis | 5.36E-25 | 9.17E-23 |
| epithelium development | 4.80E-24 | 7.67E-22 |

## Supplementary Note 1

## Comparison with chromatin state systems in previous studies

We performed a systematic comparison with five previous chromatin state systems for Drosophila melanogaster[1-3]. Specifically, for each of our chromatin states, we computed the proportions of genomic regions in that chromatin state overlapping with each chromatin state in other chromatin state systems. In summary, for coarse grained chromatin state groups such as enhancer-like states, canonical active gene sequence states, heterochromatin states, and polycomb repressed states, in most cases we found corresponding chromatin state(s) in previous chromatin state systems; however, none of the previous chromatin states distinguish the Strong Enhancer, Weak Enhancer and Long Intron states (**Supplementary Figures 1-5**). Moreover, we have found no previous chromatin state to be strongly predictive to Pol II pausing ($AUC \leq 0.68$; $AUC = 0.5$ is the expected performance of random predictions).

## Supplementary Note 2

### Analysis for single chromatin factor removal and alteration effects on chromatin state identification

We evaluated the effect of two types of single chromatin factor perturbations on chromatin state identification. For the first perturbation type (**Supplementary Figure 6**), we removed data for each chromatin factor in turn. The removed chromatin factor was then imputed from the other chromatin factor profiles by conditional probability given by our model as described in our previous work[4]. Genomic bins with conditional probability larger than 0.5 were imputed as 1 and otherwise imputed as 0. The chromatin state identification algorithm was then applied to the imputed data. The output chromatin state annotations were compared with chromatin states annotations identified with full data. For each chromatin state, the proportions of regions altered by removing each chromatin factor are shown below. For the second perturbation type (**Supplementary Figure 7, 8**), we altered a chromatin factor from present to absent or from absent to present, and then assess proportion of regions in each chromatin state changed.

Overall, for almost all chromatin states removing a single chromatin factor does not alter the chromatin state for the majority of the regions, suggesting that chromatin state identification is generally not dictated by single chromatin factor but rather integrate information from multiple chromatin factors. Alteration of chromatin factor often has larger effect and identify the chromatin factors important for the identity of the chromatin state.

## Supplementary Note 3

**Pseudocode 1. Finding local minima of chromatin code energy landscape**

**Input:** all observed chromatin codes, chromatin model

```
for chromatin code in observed chromatin codes:
    current code ← chromatin code
    list of neighbor codes with distance 1 ← flip current code at each chromatin feature
    while energy of any neighbor chromatin code is larger than the current
        current code ← lowest energy code in the list of neighbor codes
    end
    local minimum associated with the chromatin code ← current code
end
chromatin states ← sets of chromatin codes associated with the same local minimum
```

**Output**: chromatin states

**Pseudocode 2. Iterative combination of mini-states**

**Input**: list of all chromatin states, target number of chromatin states *k*, spatial connectivity scores between all pairs of chromatin states

```
anchored states ← top k largest chromatin states according to the number of observed chromatin codes
while current number of chromatin states > target number of chromatin states :
    source state ← the smallest non-anchored state
    target state ← the state that has the highest spatial connectivity score with the source state
    combine the source state with the target state.
    update spatial connectivity scores between all pairs of chromatin states after combination.
end
```

**Output:** chromatin states after combination

## Supplementary Methods

### Visualization of the transcription initiation region chromatin state sequence space

Dimensionality reduction by multidimensional scaling (MDS) was used to visualize transcription initiation region (-500bp to 1000bp relative to TSS) chromatin state sequence in a two-dimensional plane with each point representing the chromatin state sequence of a gene. We first computed dissimilarities for each pair of chromatin state sequence. We defined the dissimilarity metric as the proportion of regions with non-identical chromatin states between two sequences. MDS algorithm was applied to the distance matrix and the first two principle coordinates were plotted for visualization.

### Motif enrichment analysis

Enrichment p-values of the JASPAR Drosophila motifs[5] and promoter motifs [6,7] were calculated using the PWMEnrich R Package with the “affinity” algorithm[8]. To estimate the background distribution only genomic sequences with available processed ChIP-chip data were used. P-values were corrected for multi-hypothesis testing using the Bonferroni method.

## Supplementary References


1. Filion, G.J. *et al.* Systematic protein location mapping reveals five principal chromatin types in Drosophila cells. *Cell* **143**, 212-24 (2010).
2. Kharchenko, P.V. *et al.* Comprehensive analysis of the chromatin landscape in Drosophila melanogaster. *Nature* **471**, 480-5 (2011).
3. Ho, J.W. *et al.* Comparative analysis of metazoan chromatin organization. *Nature* **512**, 449-52 (2014).
4. Zhou, J. & Troyanskaya, O.G. Global Quantitative Modeling of Chromatin Factor Interactions. *PLoS Comput Biol* **10**, e1003525 (2014).
5. Bryne, J.C. *et al.* JASPAR, the open access database of transcription factor-binding profiles: new content and tools in the 2008 update. *Nucleic Acids Res* **36**, D102-6 (2008).
6. FitzGerald, P.C., Sturgill, D., Shyakhtenko, A., Oliver, B. & Vinson, C. Comparative genomics of Drosophila and human core promoters. *Genome Biology* **7**(2006).
7. Ohler, U., Liao, G.C., Niemann, H. & Rubin, G.M. Computational analysis of core promoters in the Drosophila genome. *Genome Biology* **3**, RESEARCH0087 (2002).
8. Stojnic, R. PWMEnrich: PWM enrichment analysis. (2012).
