# Supplementary figures and images for: Probabilistic modelling of chromatin code landscape reveals functional diversity of enhancer-like chromatin states (part 2 of 2)
Source: Nat Commun. 2016 Feb 4;7:10528. doi: 10.1038/ncomms10528 (PMC4742914; doi:10.1038/ncomms10528)

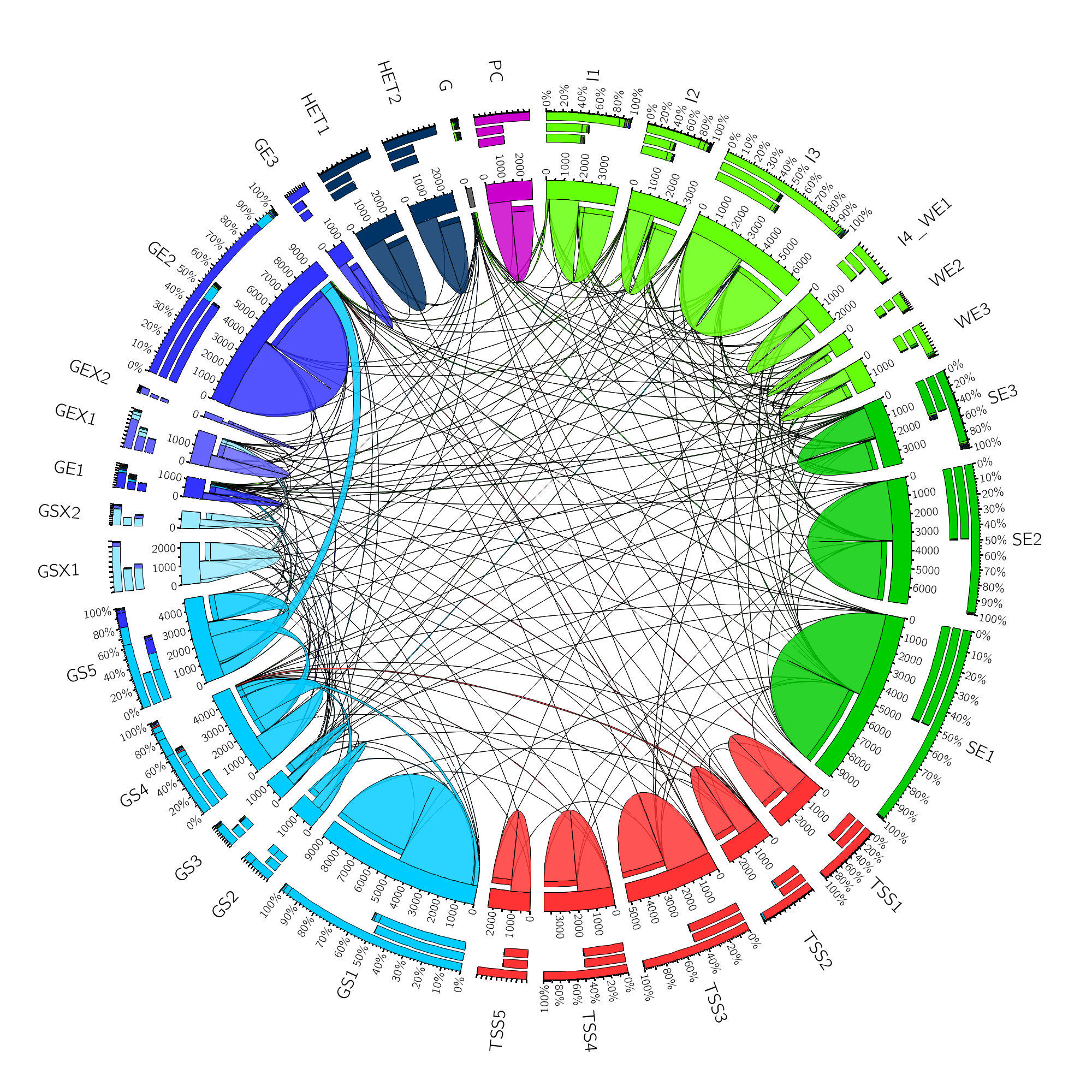

Supplement: Supplementary Data 4 — Effects of positive and negative perturbations of single chromatin factors on chromatin state identity. [file ncomms10528-s5.zip › Supplementary Data 4/PositivePerturbation/H3K79me1.png]

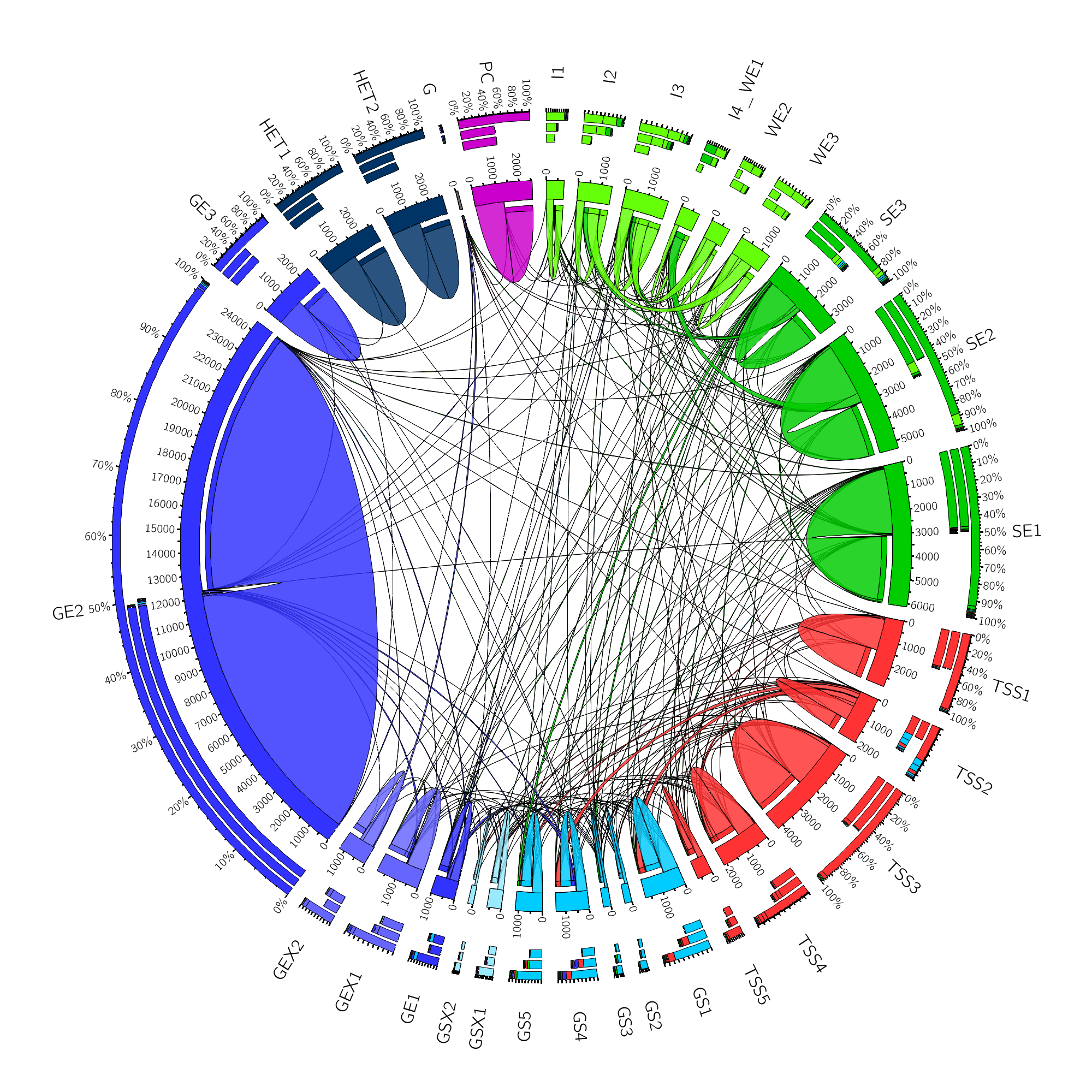

Supplement: Supplementary Data 4 — Effects of positive and negative perturbations of single chromatin factors on chromatin state identity. [file ncomms10528-s5.zip › Supplementary Data 4/PositivePerturbation/H3K79me2.png]

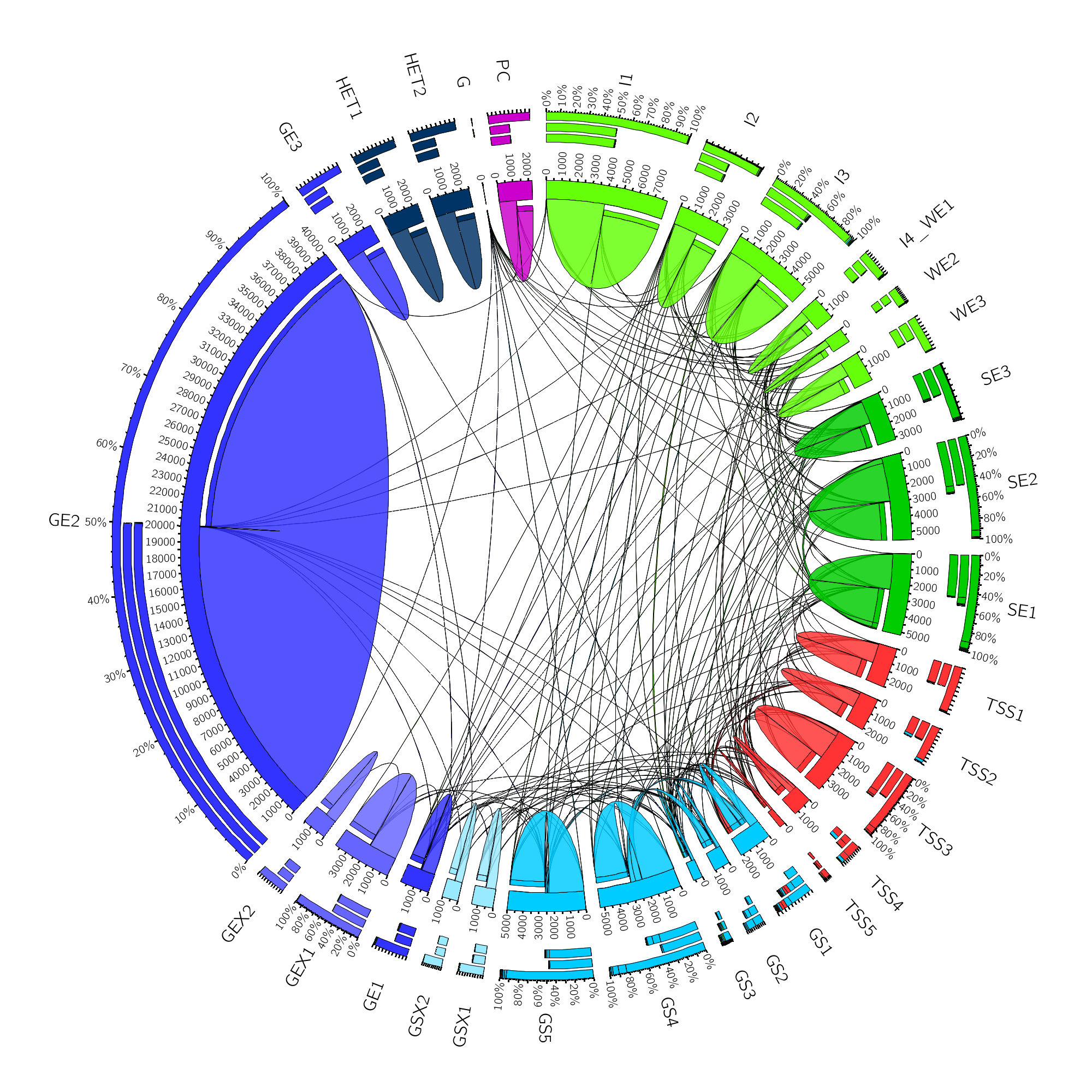

Supplement: Supplementary Data 4 — Effects of positive and negative perturbations of single chromatin factors on chromatin state identity. [file ncomms10528-s5.zip › Supplementary Data 4/PositivePerturbation/H3K9ac.png]

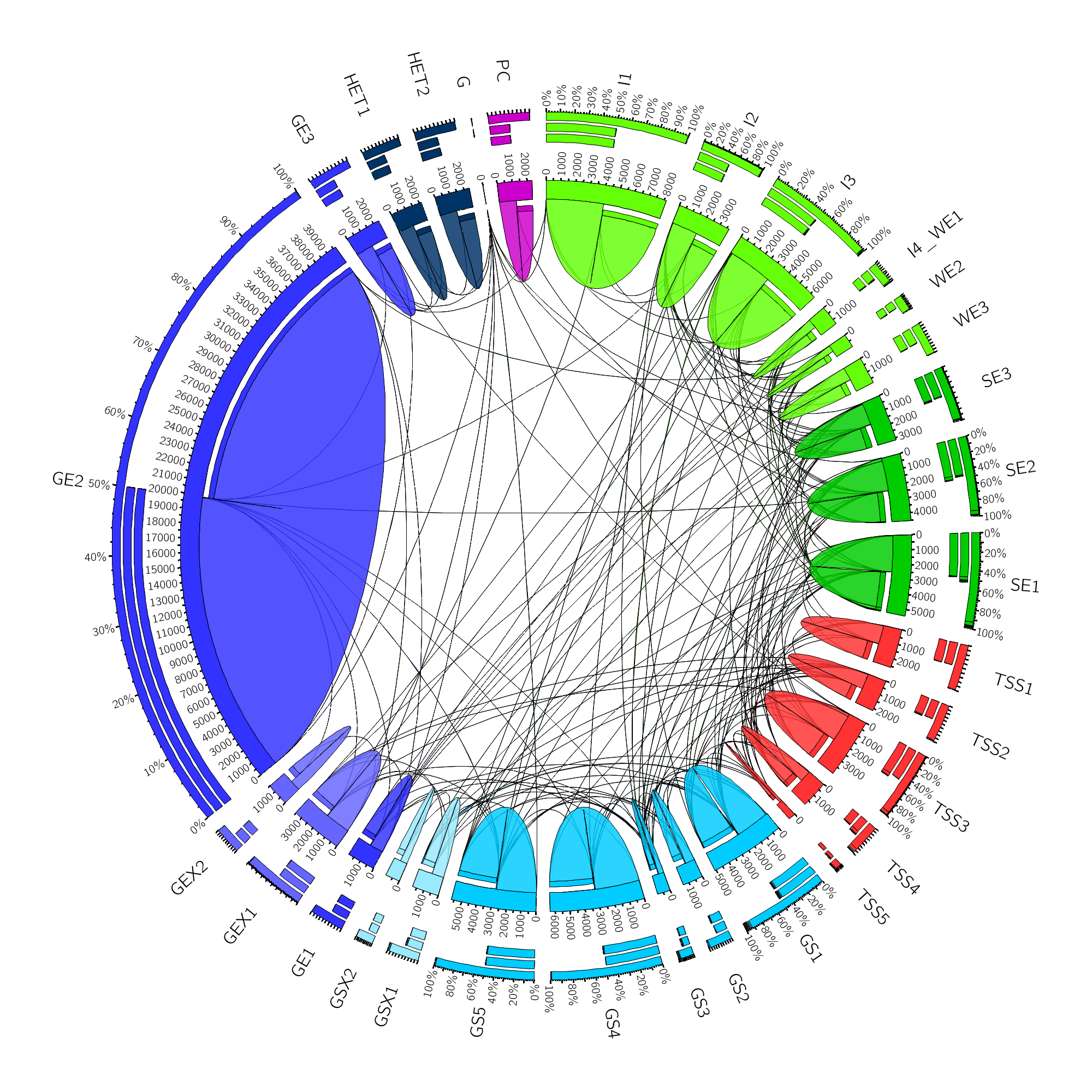

Supplement: Supplementary Data 4 — Effects of positive and negative perturbations of single chromatin factors on chromatin state identity. [file ncomms10528-s5.zip › Supplementary Data 4/PositivePerturbation/H3K9acS10P.png]

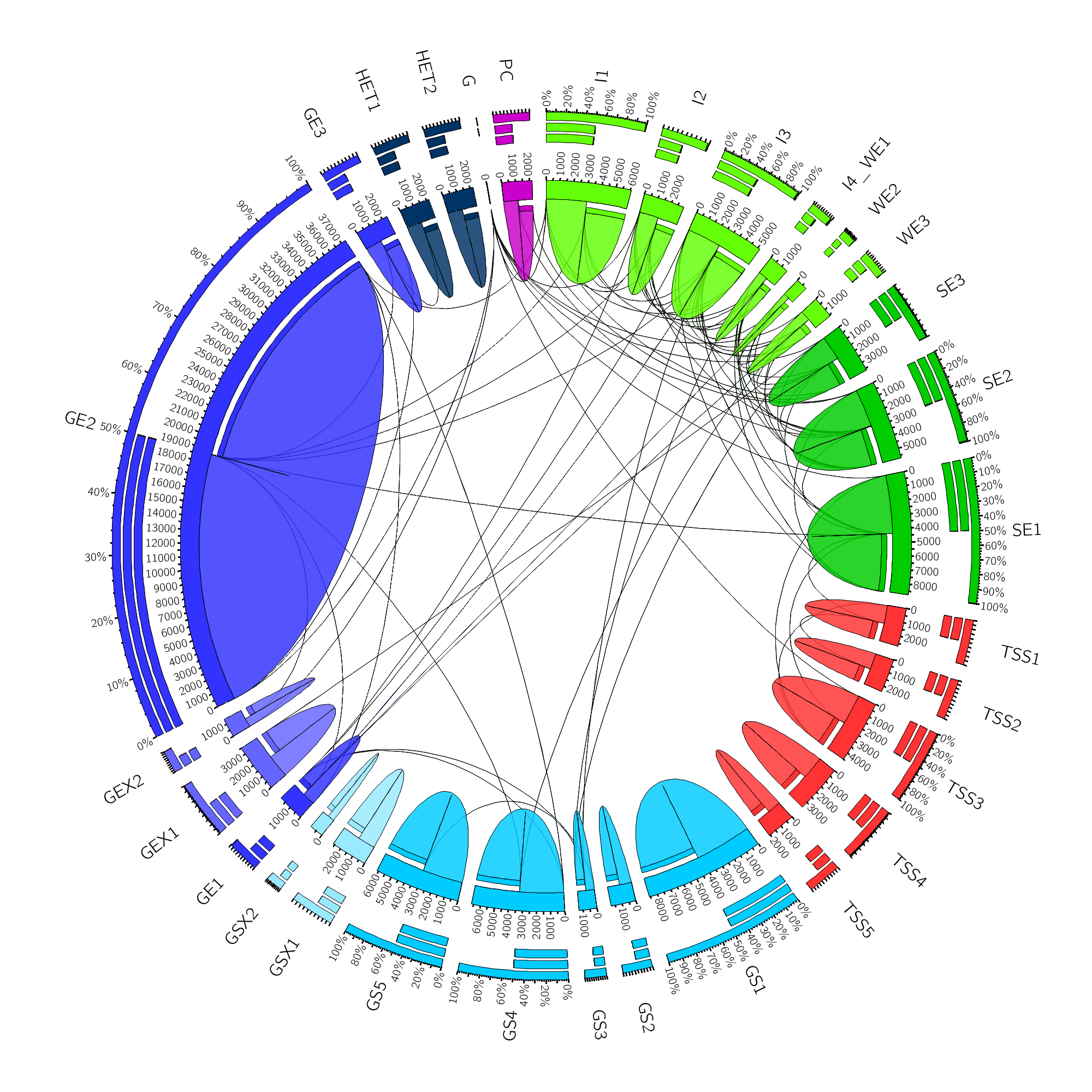

Supplement: Supplementary Data 4 — Effects of positive and negative perturbations of single chromatin factors on chromatin state identity. [file ncomms10528-s5.zip › Supplementary Data 4/PositivePerturbation/H3K9me1.png]

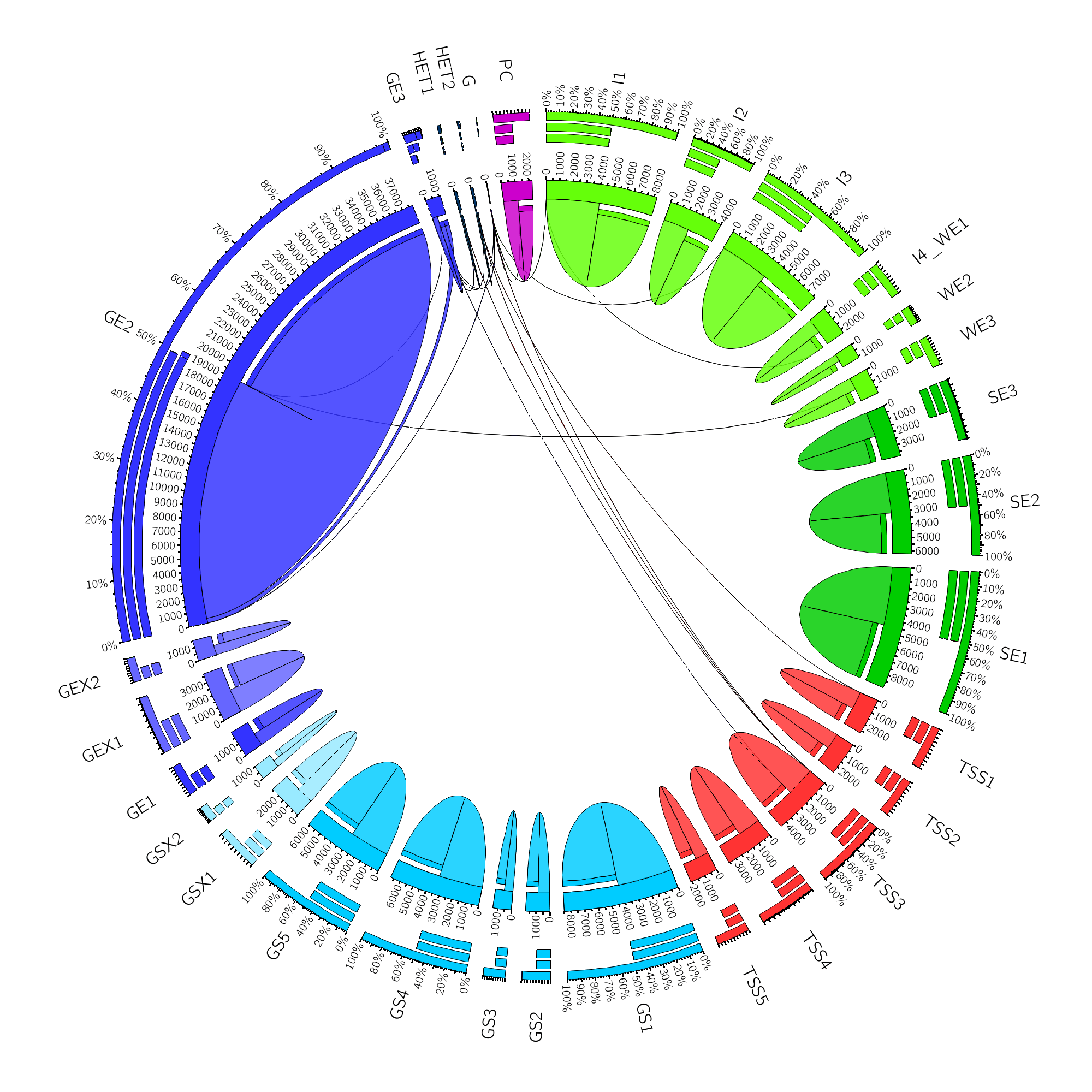

Supplement: Supplementary Data 4 — Effects of positive and negative perturbations of single chromatin factors on chromatin state identity. [file ncomms10528-s5.zip › Supplementary Data 4/PositivePerturbation/H3K9me2.png]

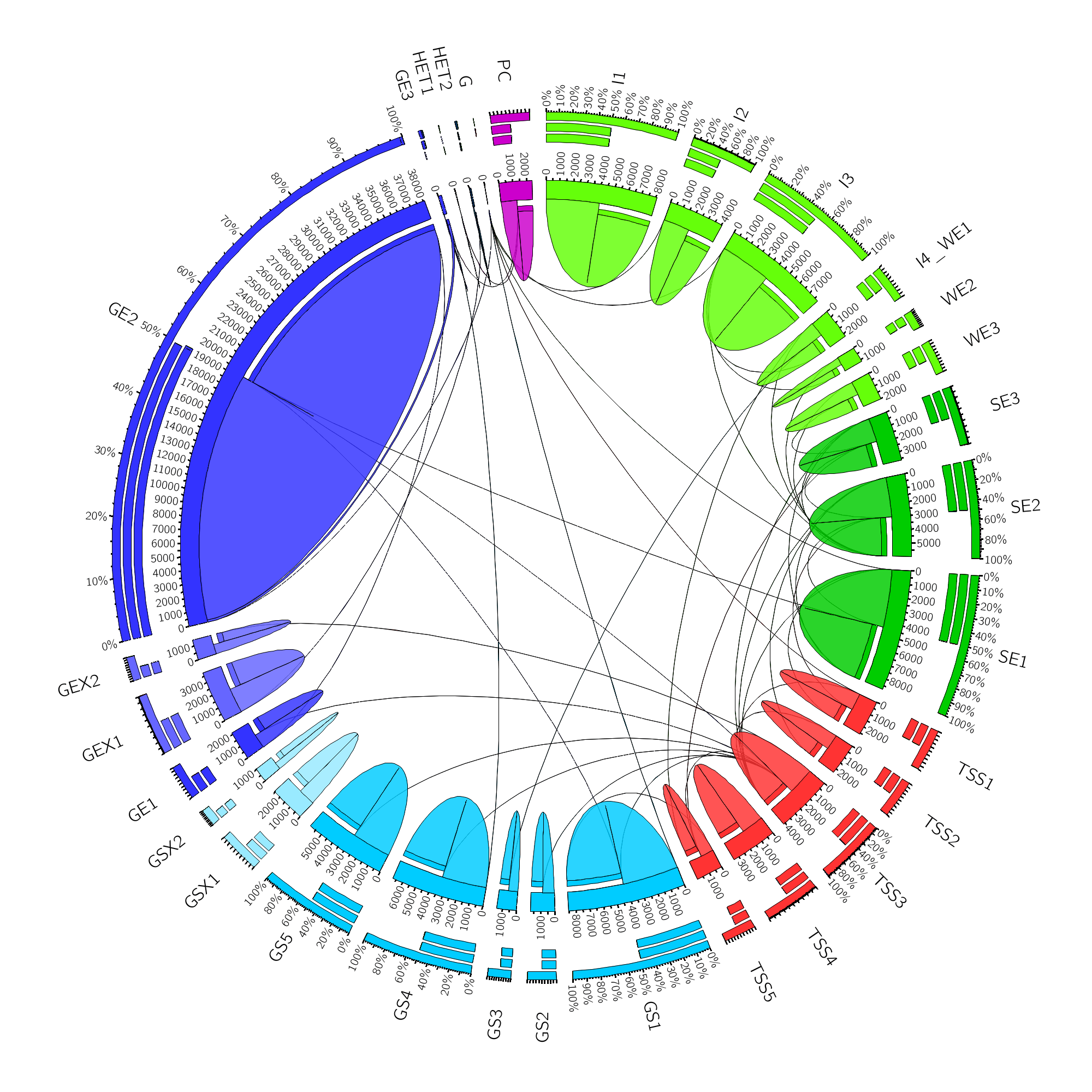

Supplement: Supplementary Data 4 — Effects of positive and negative perturbations of single chromatin factors on chromatin state identity. [file ncomms10528-s5.zip › Supplementary Data 4/PositivePerturbation/H3K9me3.png]

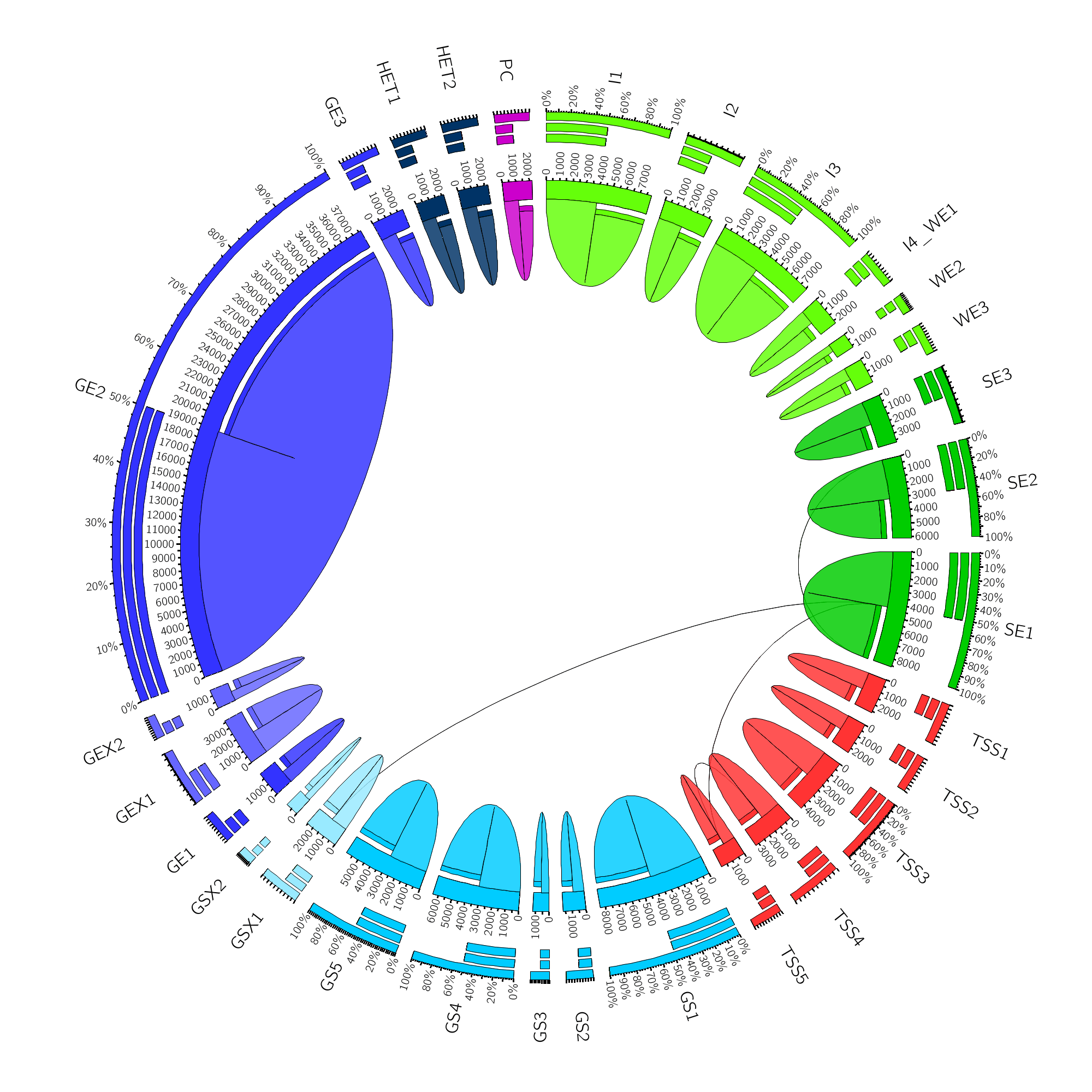

Supplement: Supplementary Data 4 — Effects of positive and negative perturbations of single chromatin factors on chromatin state identity. [file ncomms10528-s5.zip › Supplementary Data 4/PositivePerturbation/H4.png]

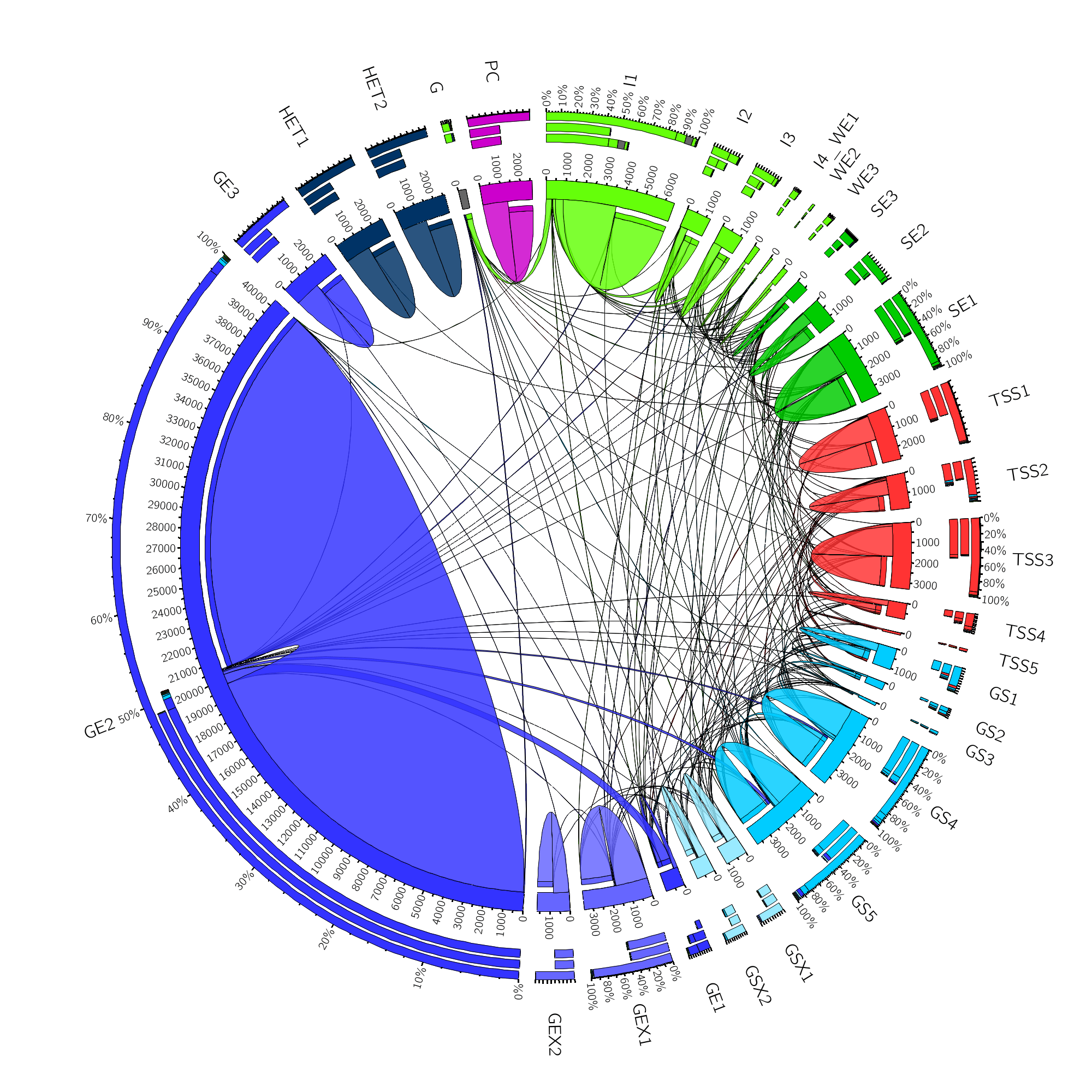

Supplement: Supplementary Data 4 — Effects of positive and negative perturbations of single chromatin factors on chromatin state identity. [file ncomms10528-s5.zip › Supplementary Data 4/PositivePerturbation/H4acTetra.png]

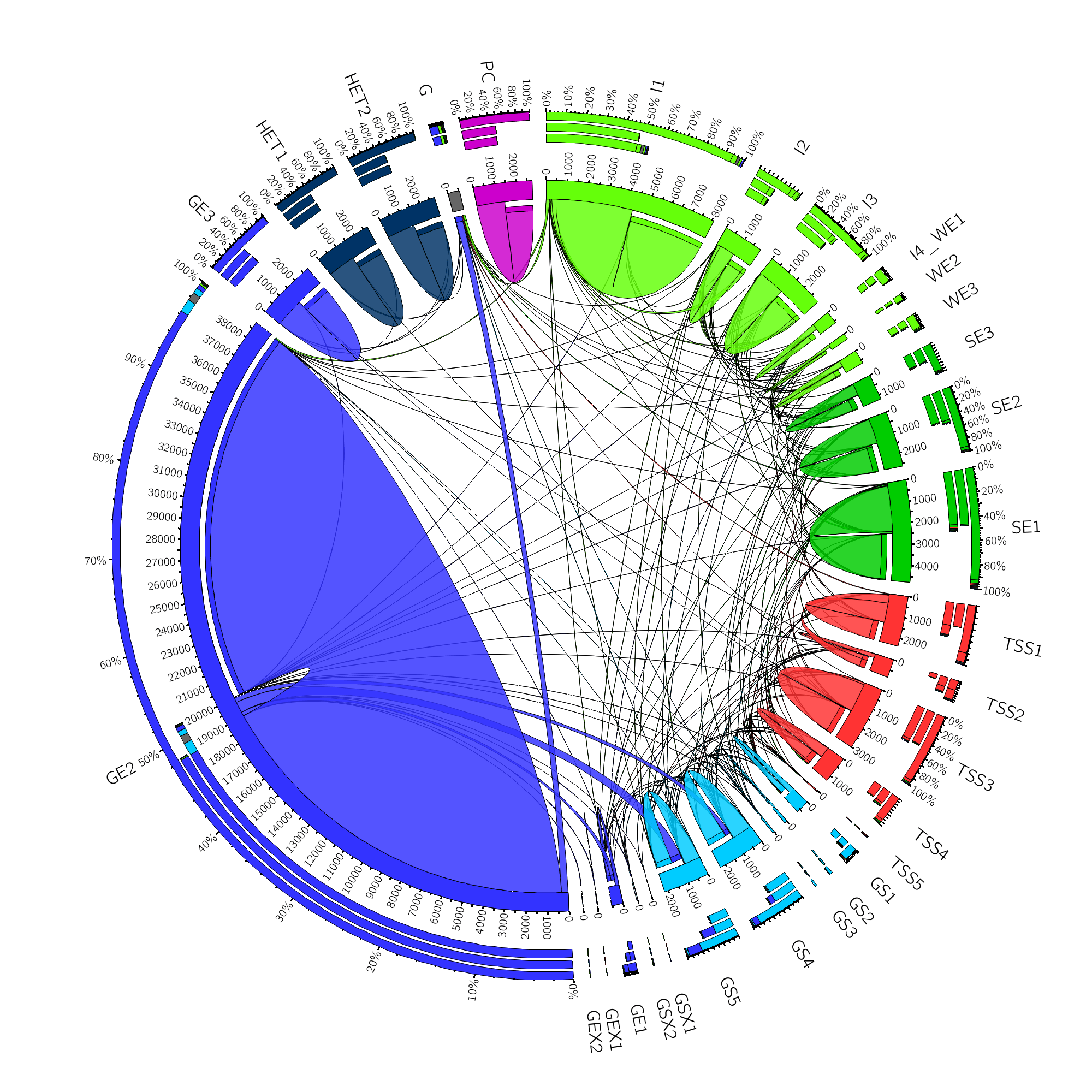

Supplement: Supplementary Data 4 — Effects of positive and negative perturbations of single chromatin factors on chromatin state identity. [file ncomms10528-s5.zip › Supplementary Data 4/PositivePerturbation/H4K16ac.png]

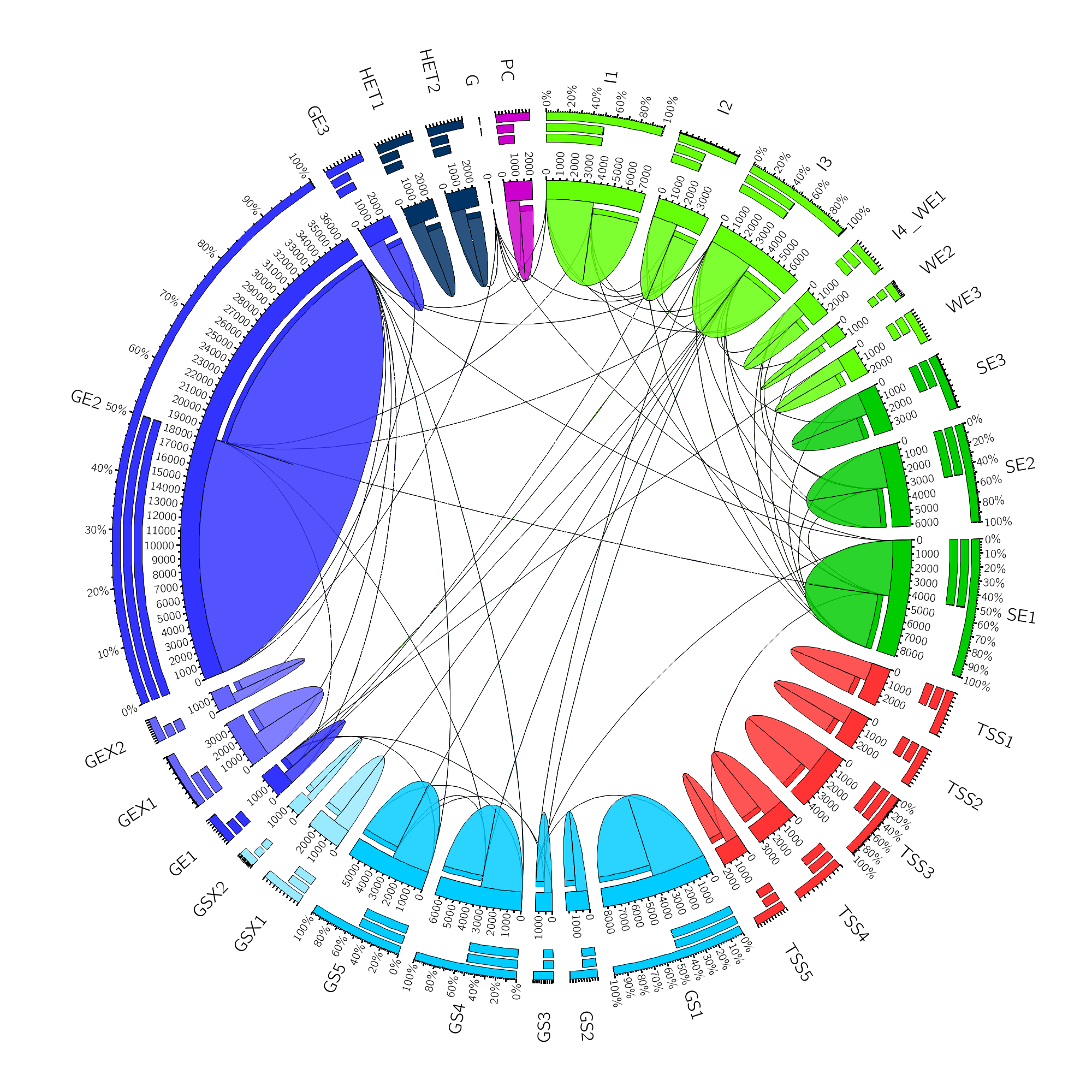

Supplement: Supplementary Data 4 — Effects of positive and negative perturbations of single chromatin factors on chromatin state identity. [file ncomms10528-s5.zip › Supplementary Data 4/PositivePerturbation/H4K20me1.png]

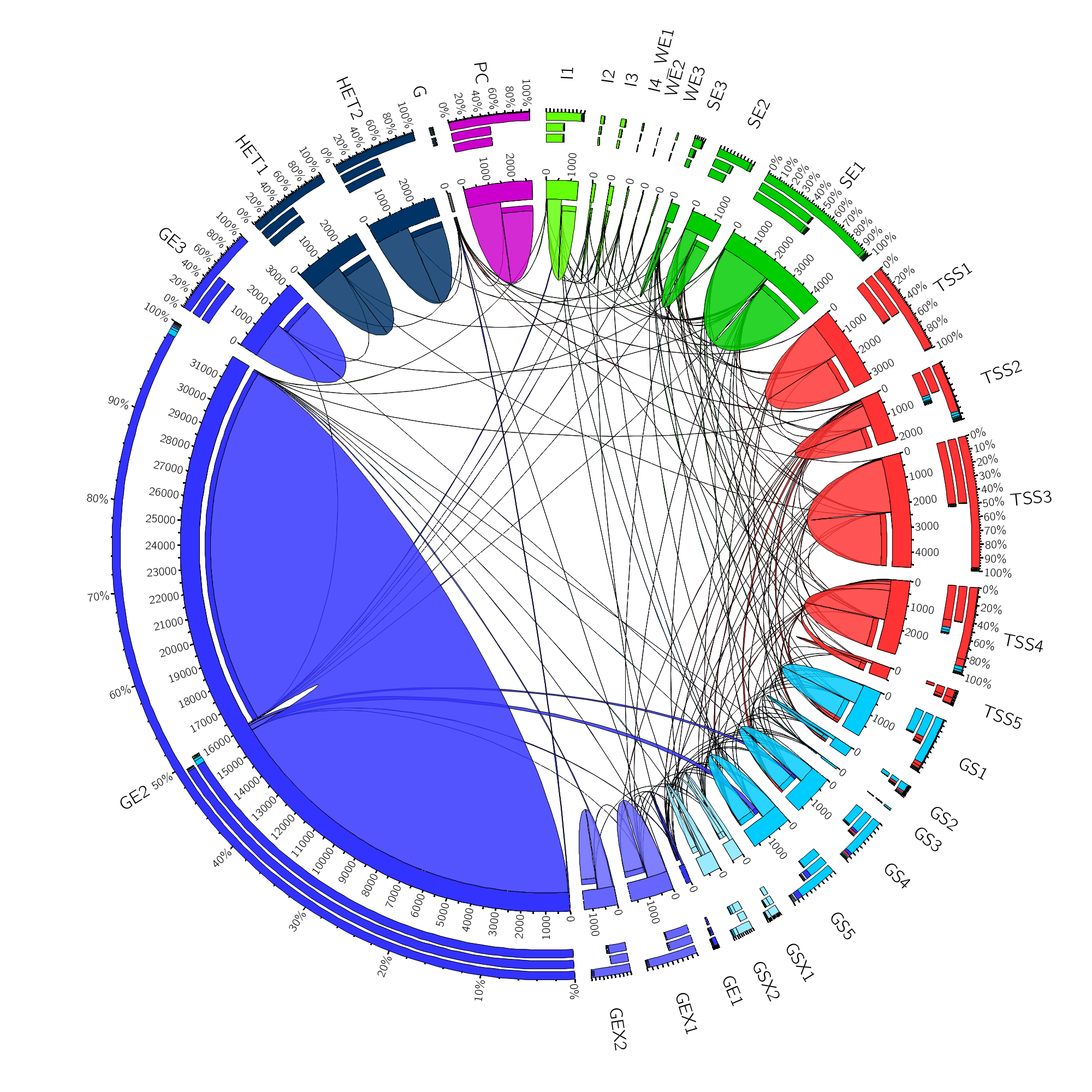

Supplement: Supplementary Data 4 — Effects of positive and negative perturbations of single chromatin factors on chromatin state identity. [file ncomms10528-s5.zip › Supplementary Data 4/PositivePerturbation/H4K5ac.png]

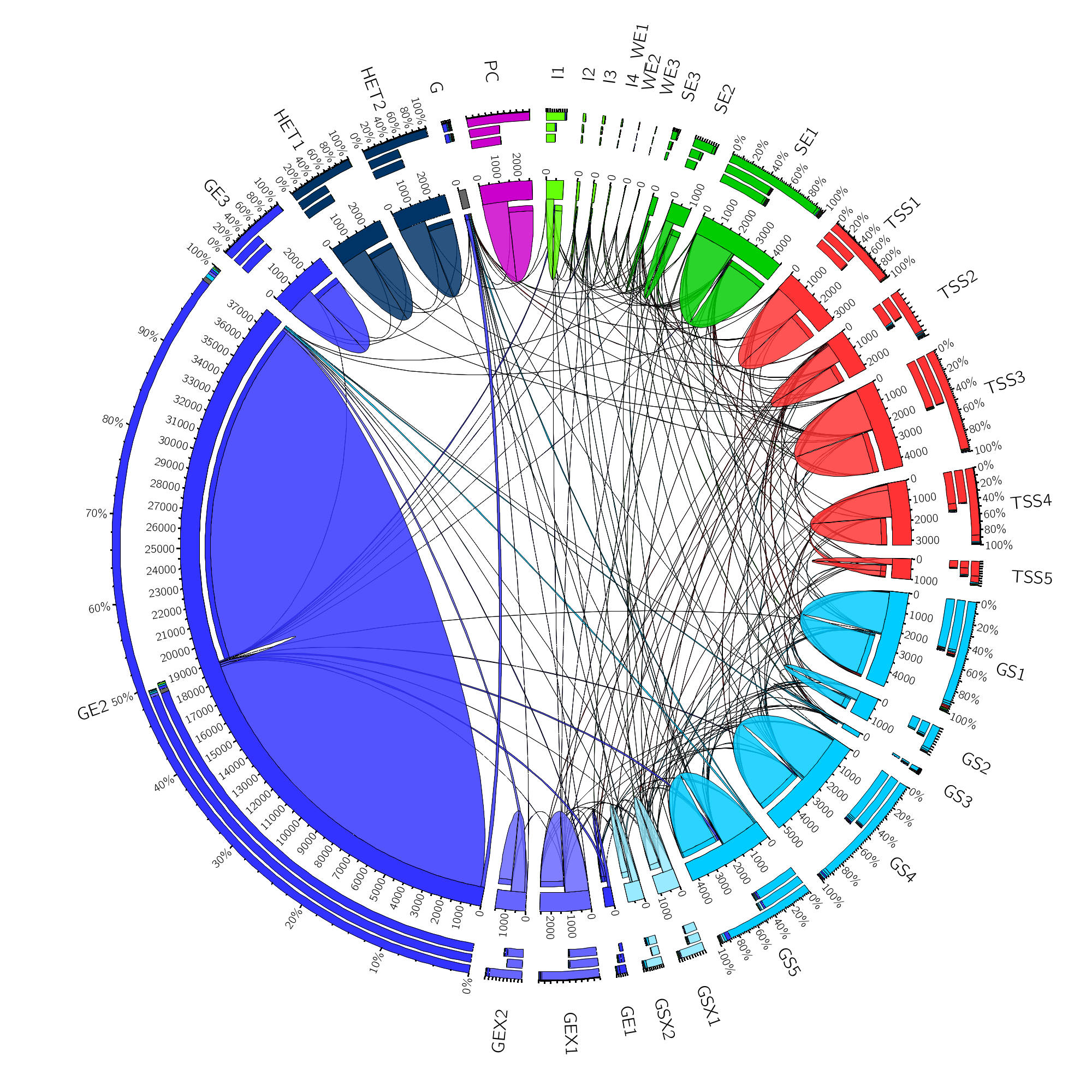

Supplement: Supplementary Data 4 — Effects of positive and negative perturbations of single chromatin factors on chromatin state identity. [file ncomms10528-s5.zip › Supplementary Data 4/PositivePerturbation/H4K8ac.png]

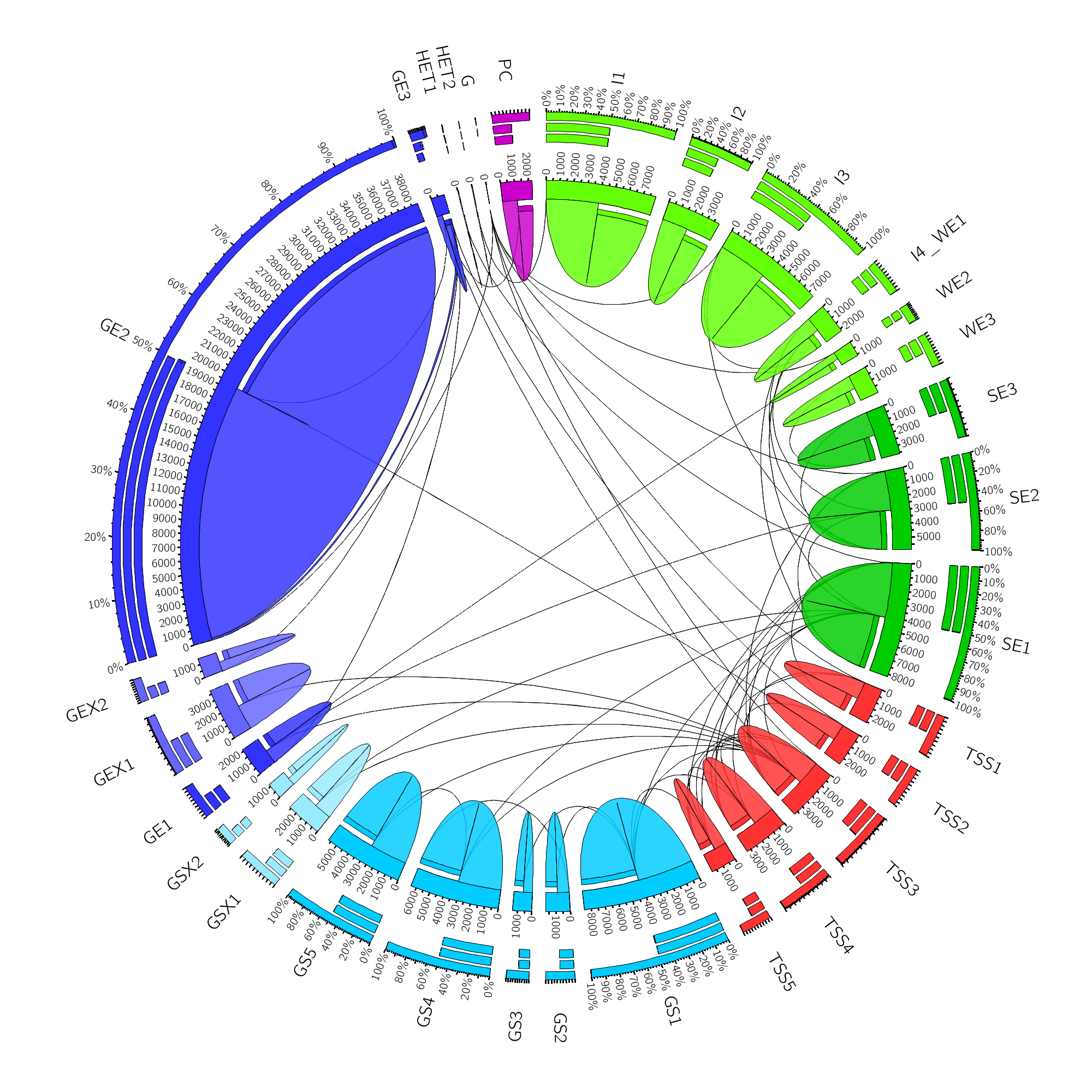

Supplement: Supplementary Data 4 — Effects of positive and negative perturbations of single chromatin factors on chromatin state identity. [file ncomms10528-s5.zip › Supplementary Data 4/PositivePerturbation/HP1a.png]

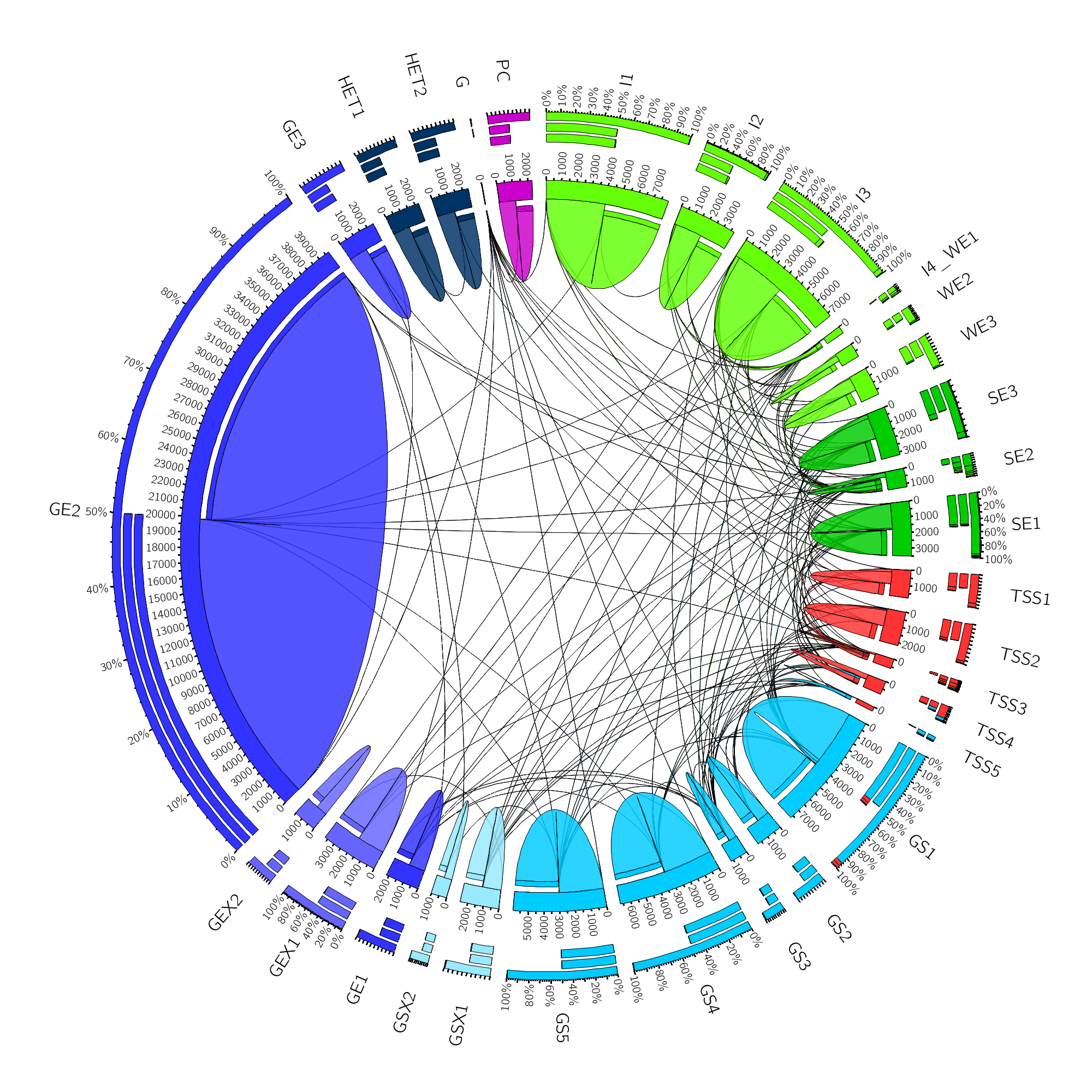

Supplement: Supplementary Data 4 — Effects of positive and negative perturbations of single chromatin factors on chromatin state identity. [file ncomms10528-s5.zip › Supplementary Data 4/PositivePerturbation/HP1b.png]

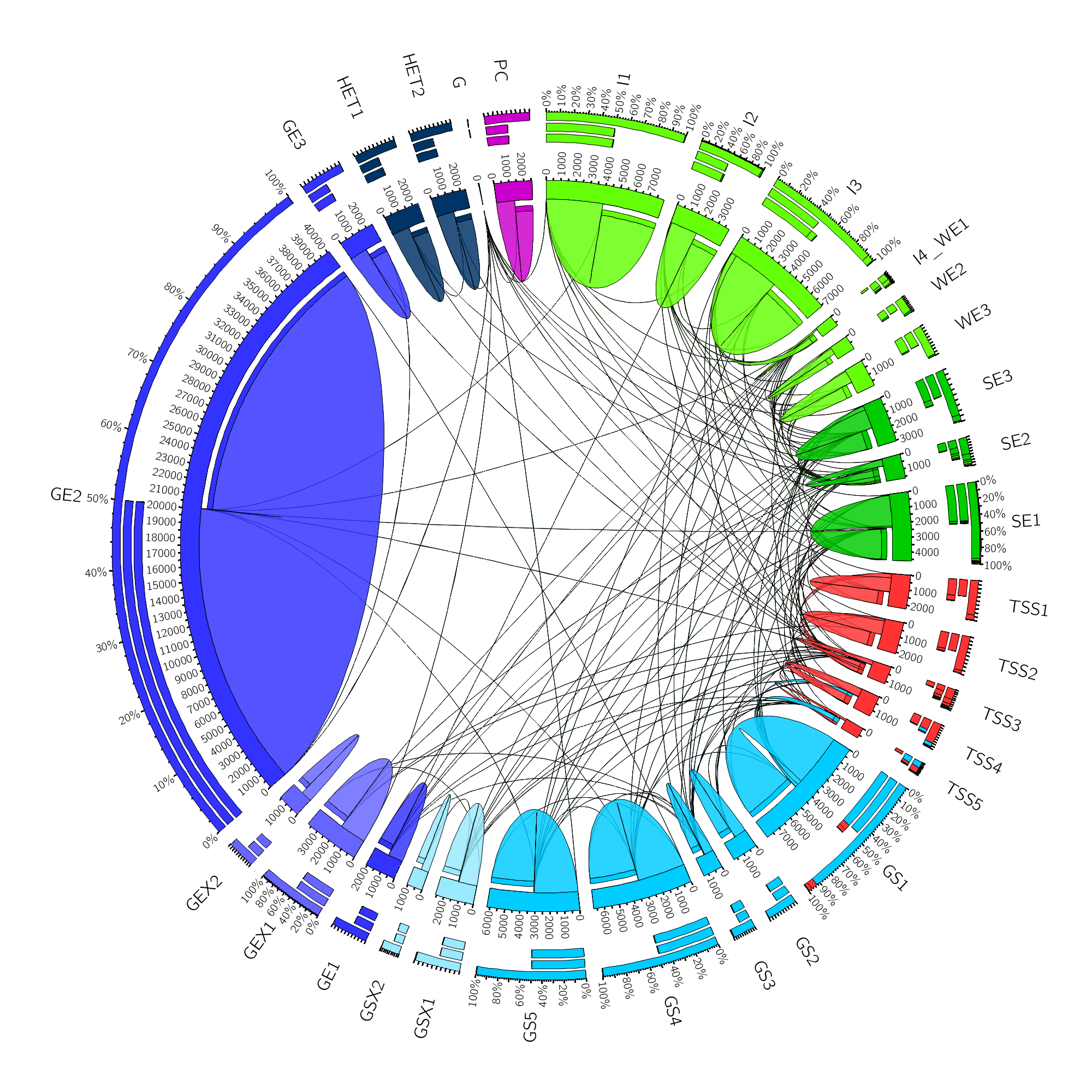

Supplement: Supplementary Data 4 — Effects of positive and negative perturbations of single chromatin factors on chromatin state identity. [file ncomms10528-s5.zip › Supplementary Data 4/PositivePerturbation/HP1c.png]

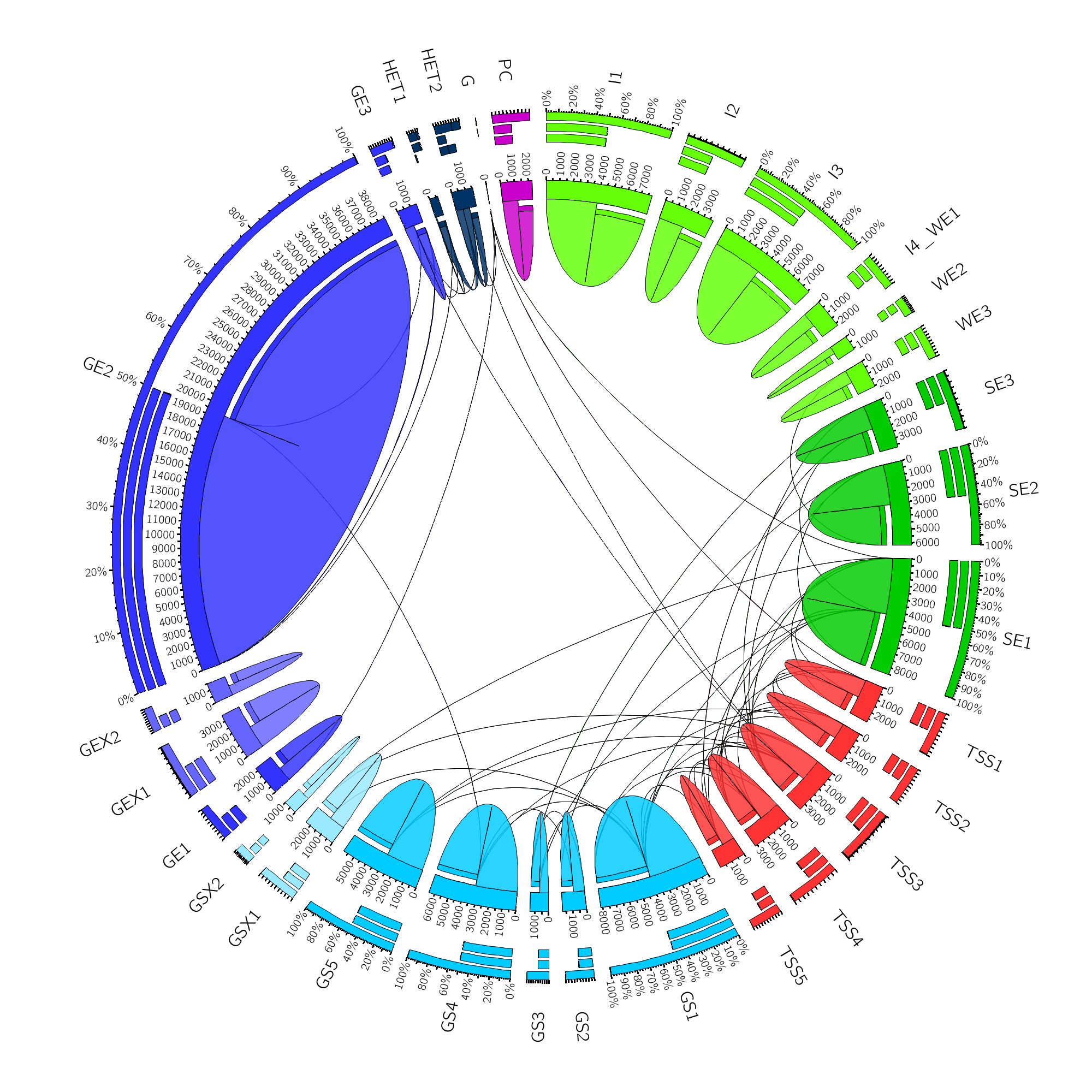

Supplement: Supplementary Data 4 — Effects of positive and negative perturbations of single chromatin factors on chromatin state identity. [file ncomms10528-s5.zip › Supplementary Data 4/PositivePerturbation/HP2.png]

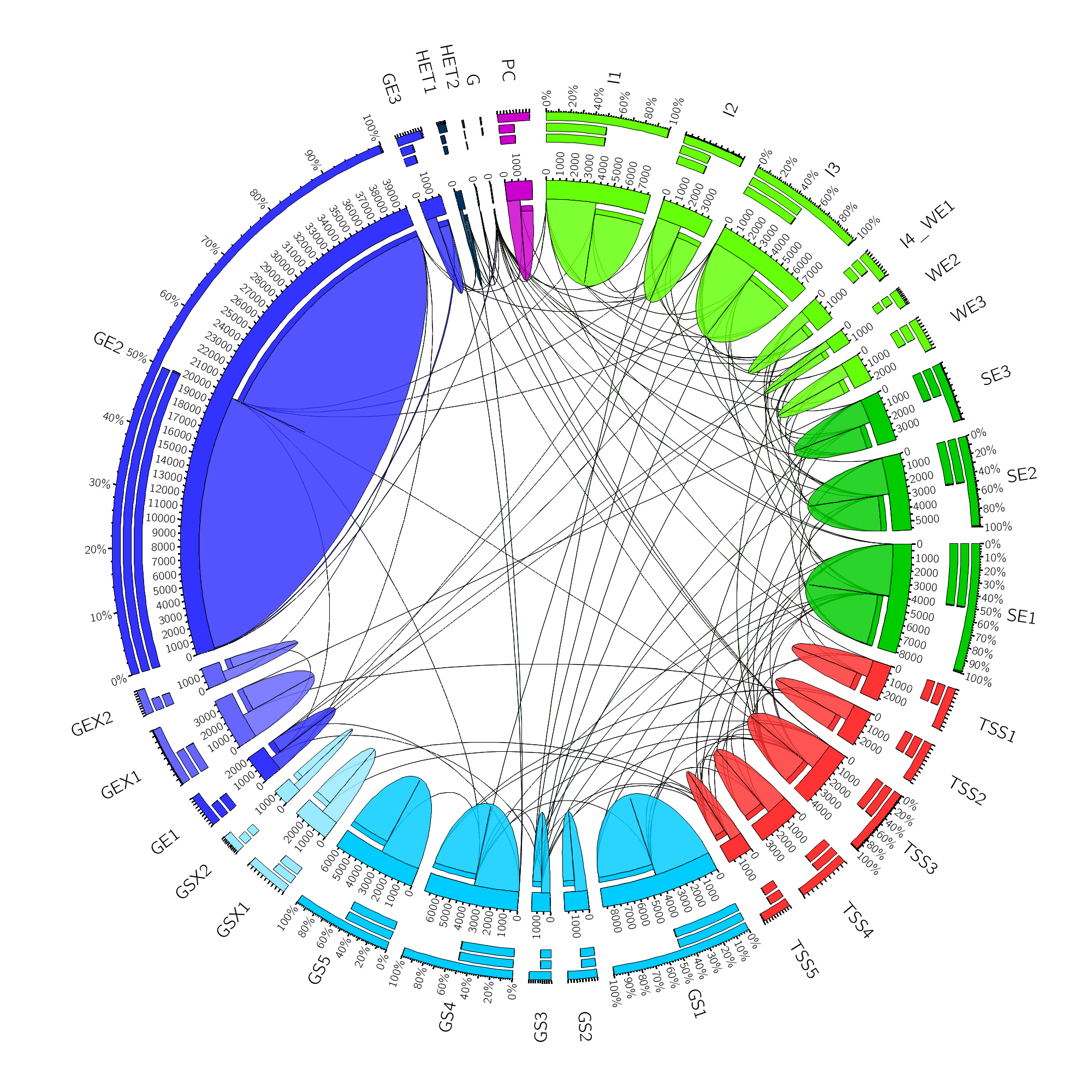

Supplement: Supplementary Data 4 — Effects of positive and negative perturbations of single chromatin factors on chromatin state identity. [file ncomms10528-s5.zip › Supplementary Data 4/PositivePerturbation/HP4.png]

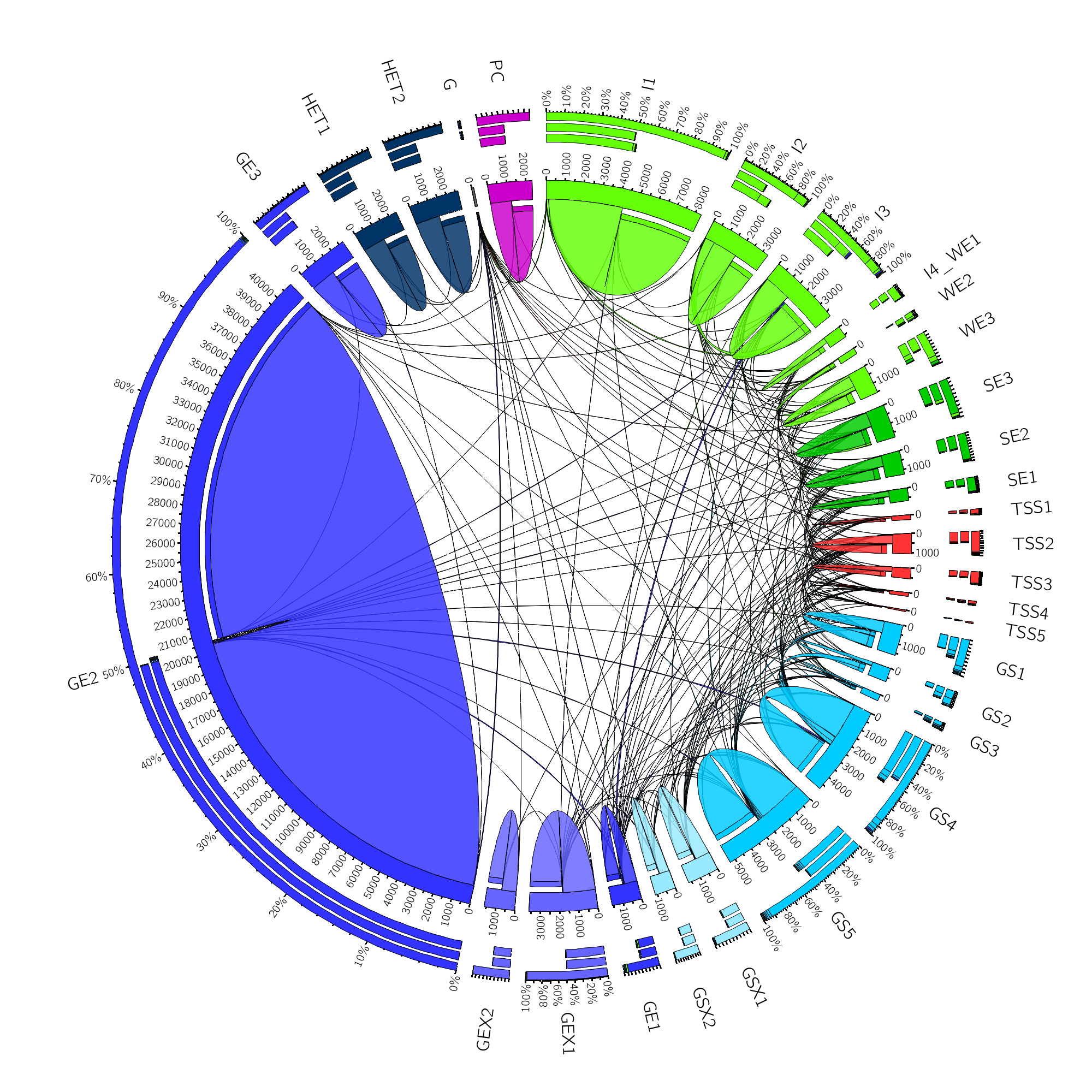

Supplement: Supplementary Data 4 — Effects of positive and negative perturbations of single chromatin factors on chromatin state identity. [file ncomms10528-s5.zip › Supplementary Data 4/PositivePerturbation/ISWI.png]

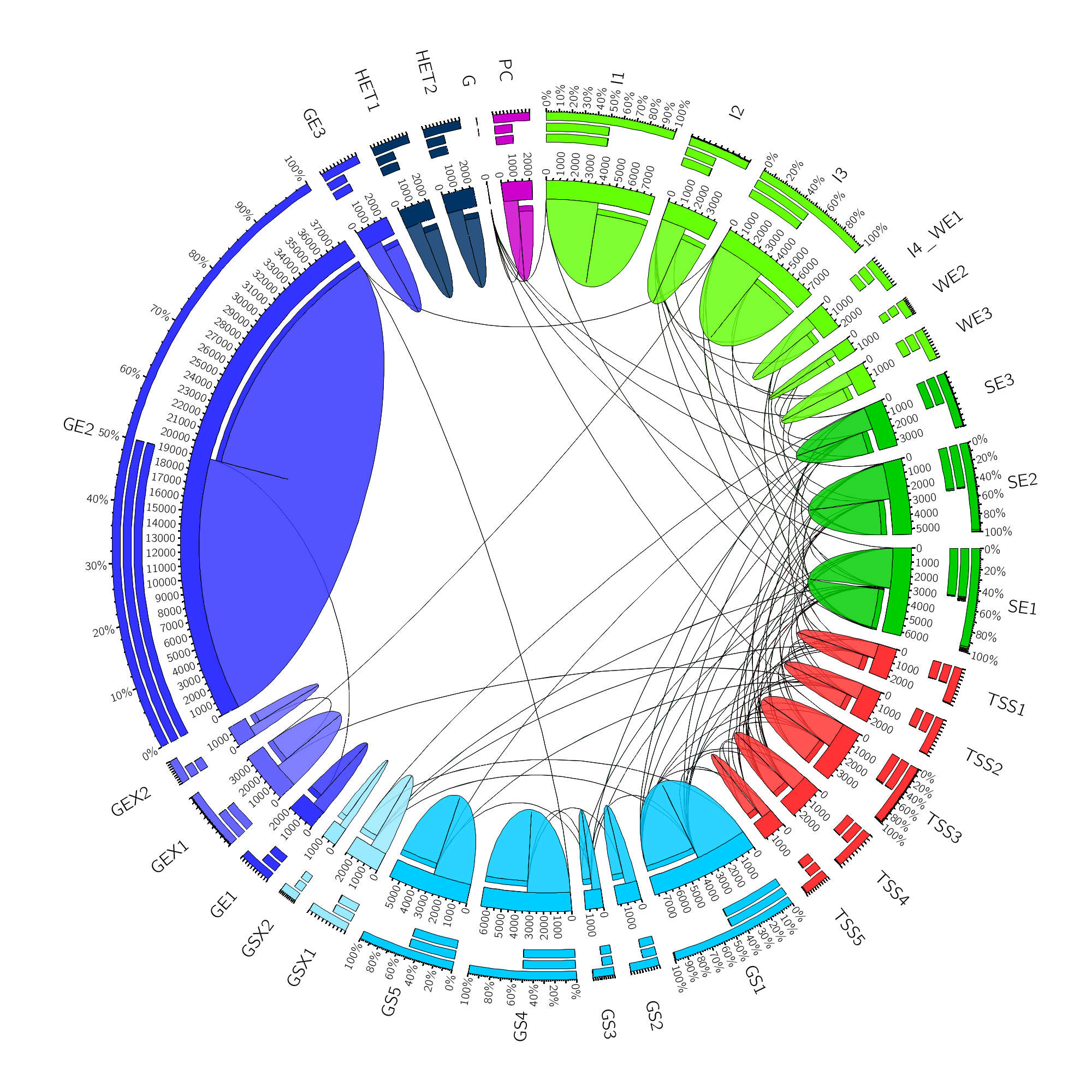

Supplement: Supplementary Data 4 — Effects of positive and negative perturbations of single chromatin factors on chromatin state identity. [file ncomms10528-s5.zip › Supplementary Data 4/PositivePerturbation/JHDM1.png]

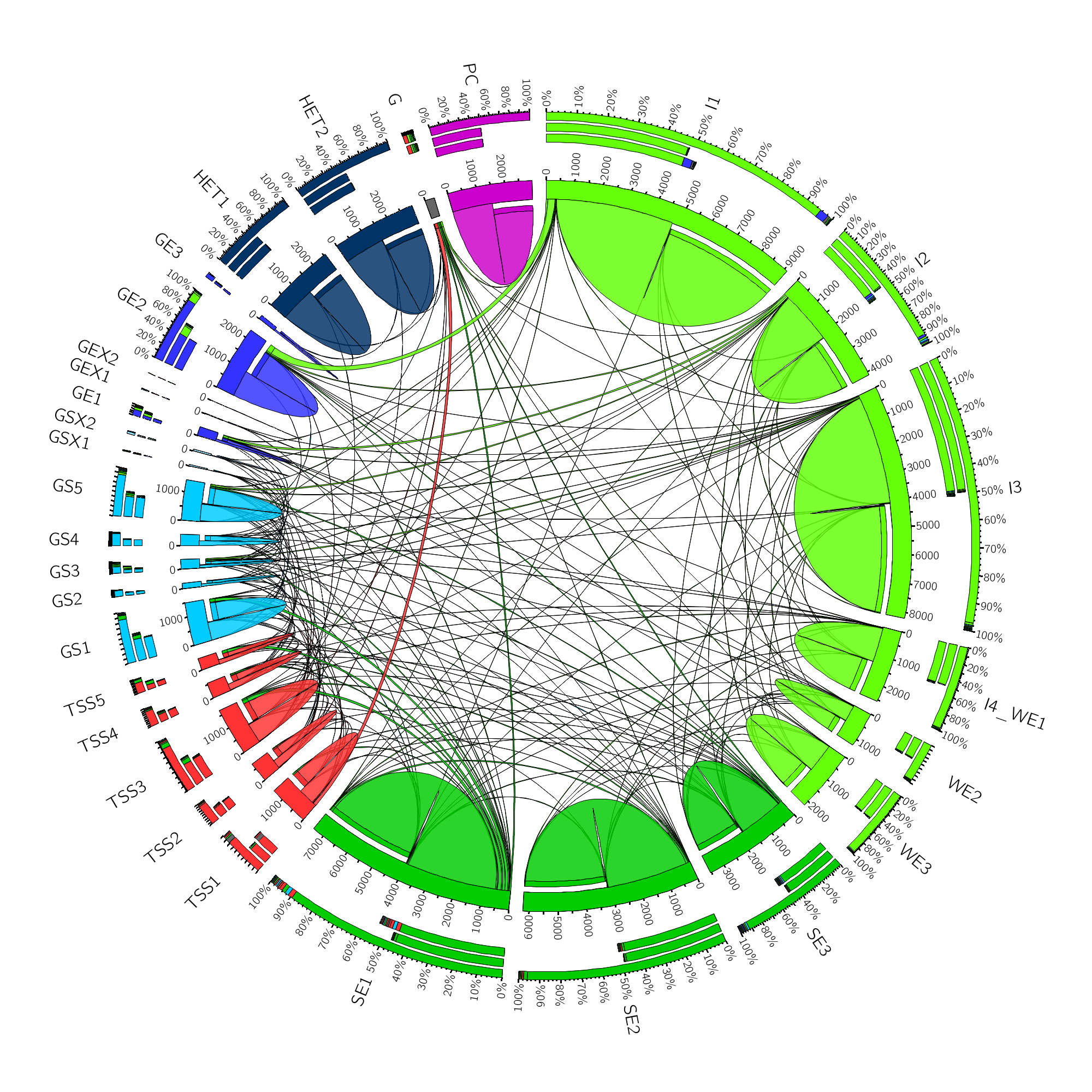

Supplement: Supplementary Data 4 — Effects of positive and negative perturbations of single chromatin factors on chromatin state identity. [file ncomms10528-s5.zip › Supplementary Data 4/PositivePerturbation/JIL1.png]

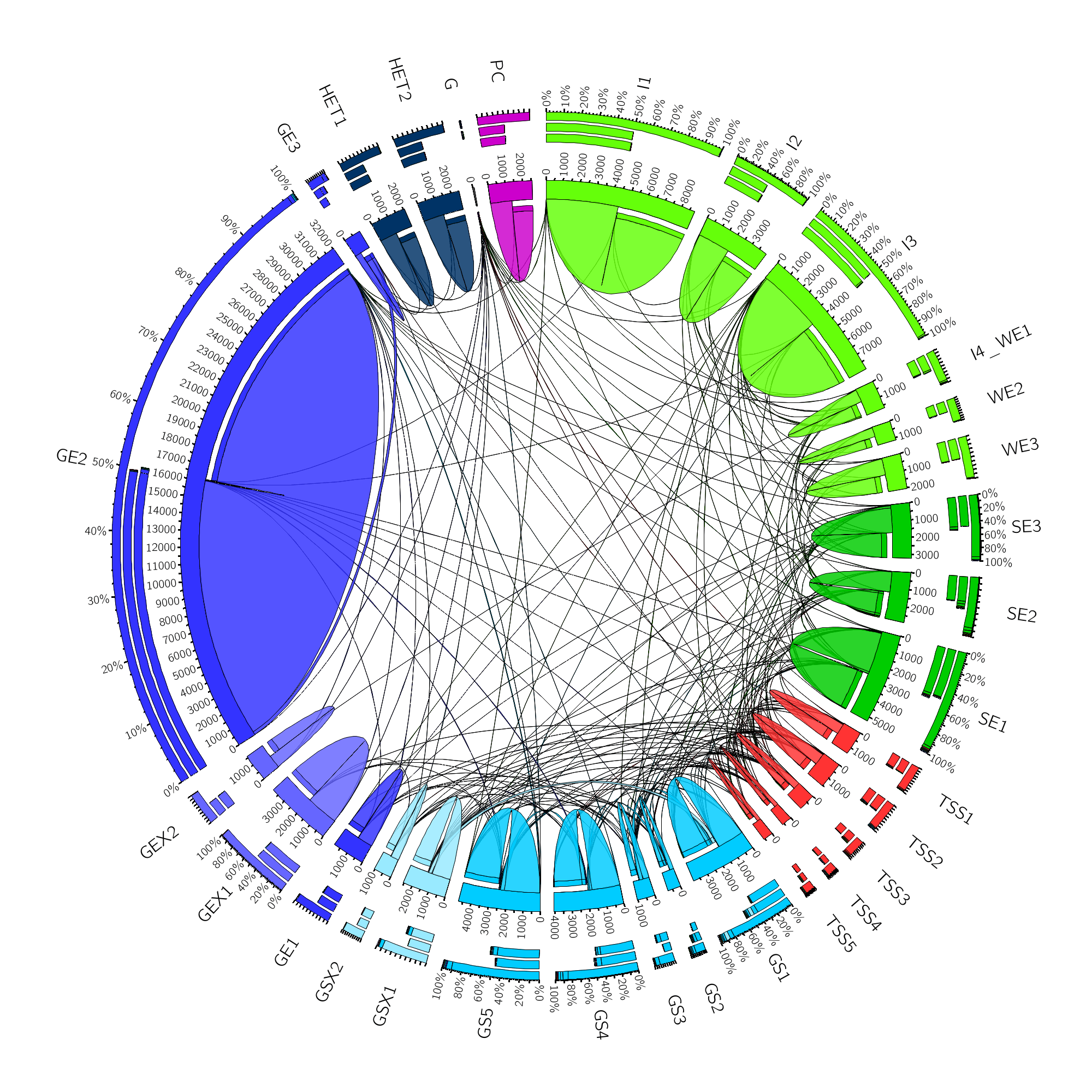

Supplement: Supplementary Data 4 — Effects of positive and negative perturbations of single chromatin factors on chromatin state identity. [file ncomms10528-s5.zip › Supplementary Data 4/PositivePerturbation/JMJD2AKDM4A.png]

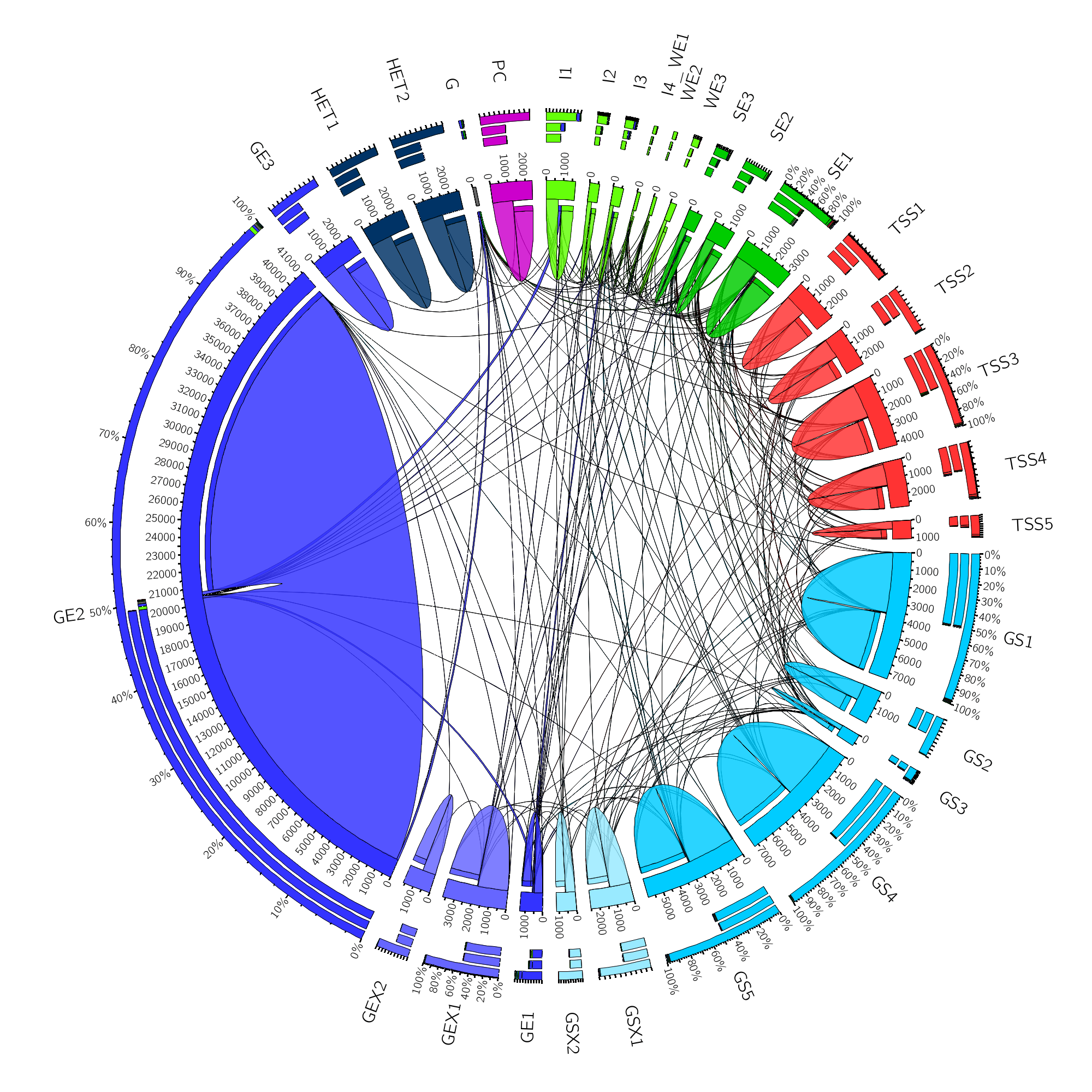

Supplement: Supplementary Data 4 — Effects of positive and negative perturbations of single chromatin factors on chromatin state identity. [file ncomms10528-s5.zip › Supplementary Data 4/PositivePerturbation/LSD1.png]

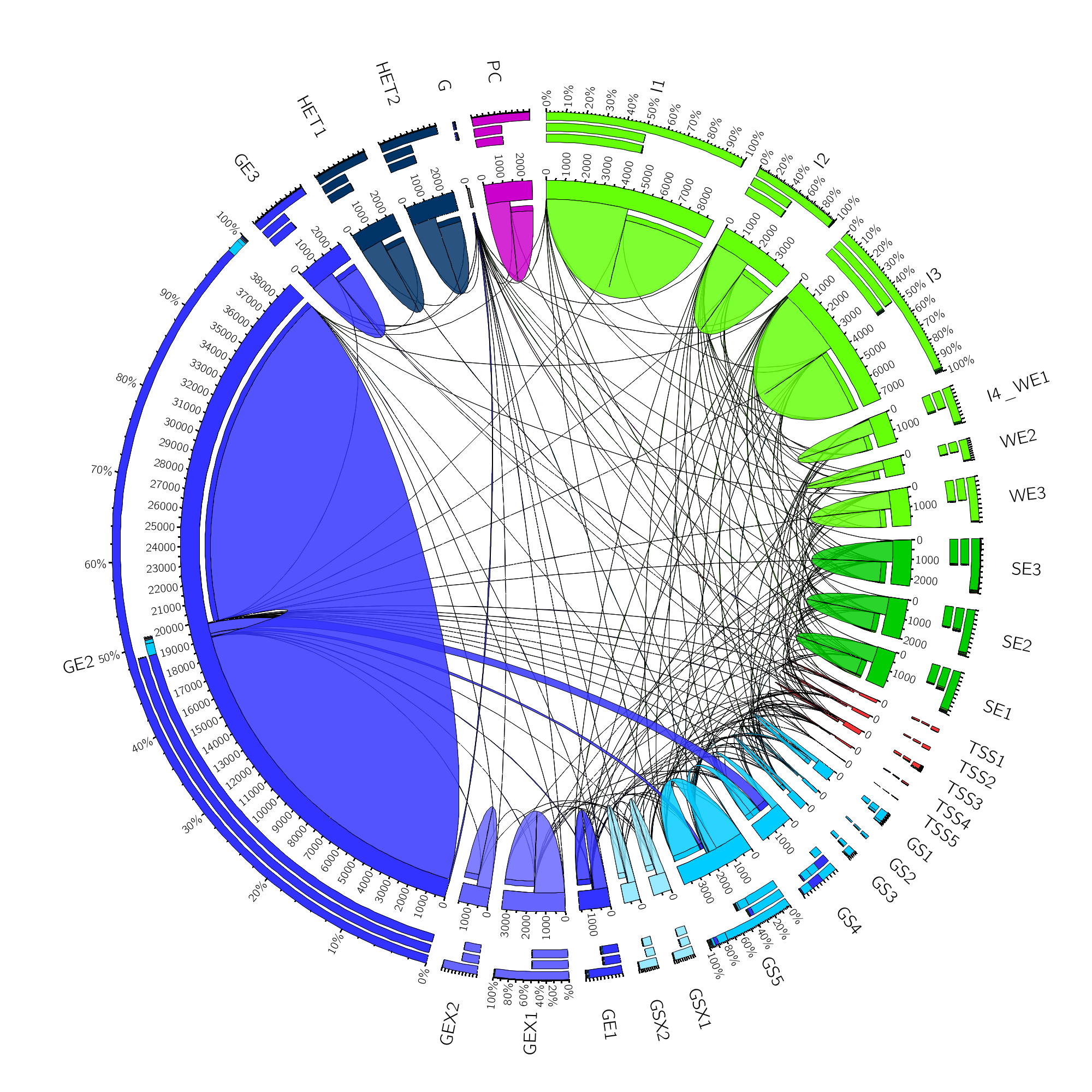

Supplement: Supplementary Data 4 — Effects of positive and negative perturbations of single chromatin factors on chromatin state identity. [file ncomms10528-s5.zip › Supplementary Data 4/PositivePerturbation/MBDR2.png]

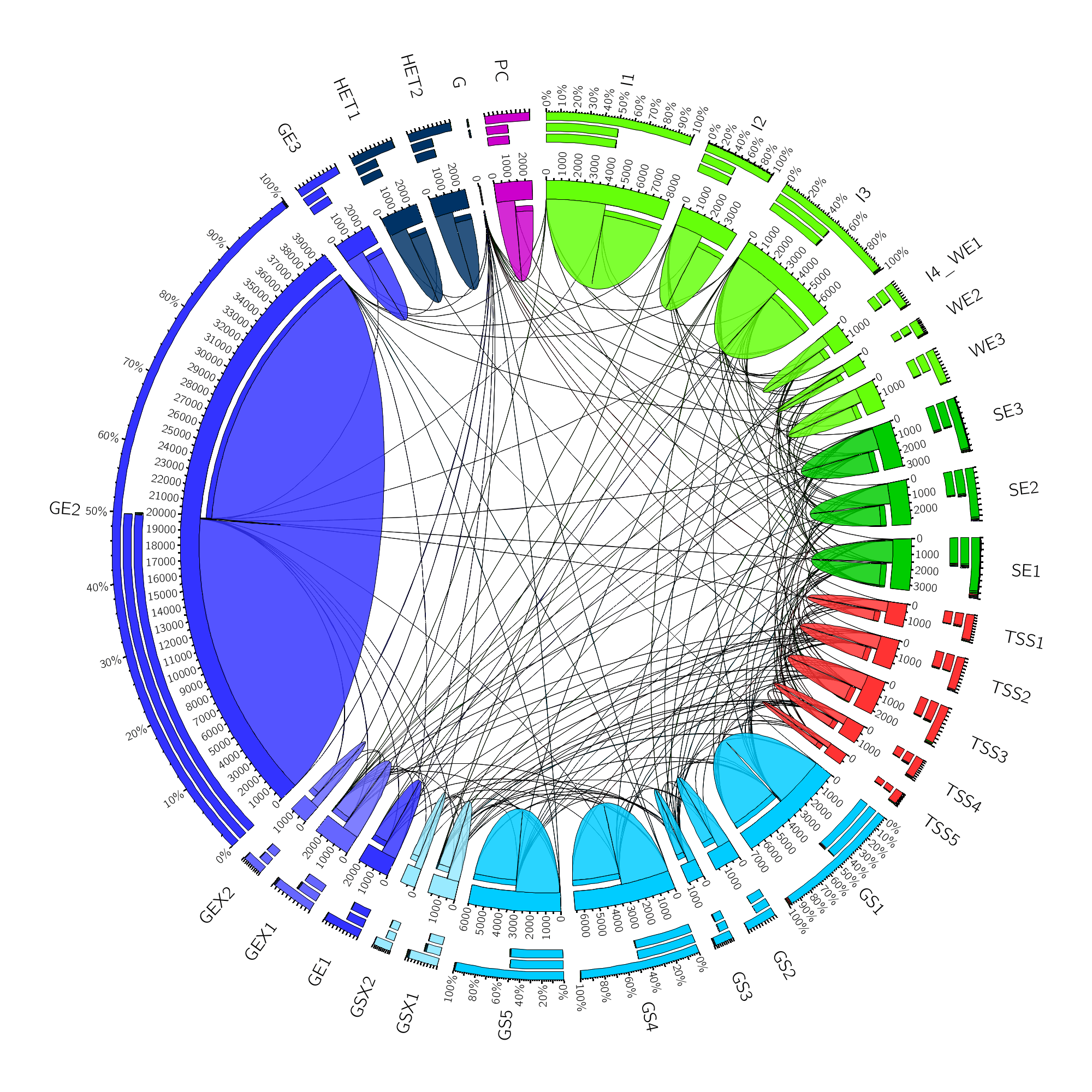

Supplement: Supplementary Data 4 — Effects of positive and negative perturbations of single chromatin factors on chromatin state identity. [file ncomms10528-s5.zip › Supplementary Data 4/PositivePerturbation/MLE.png]

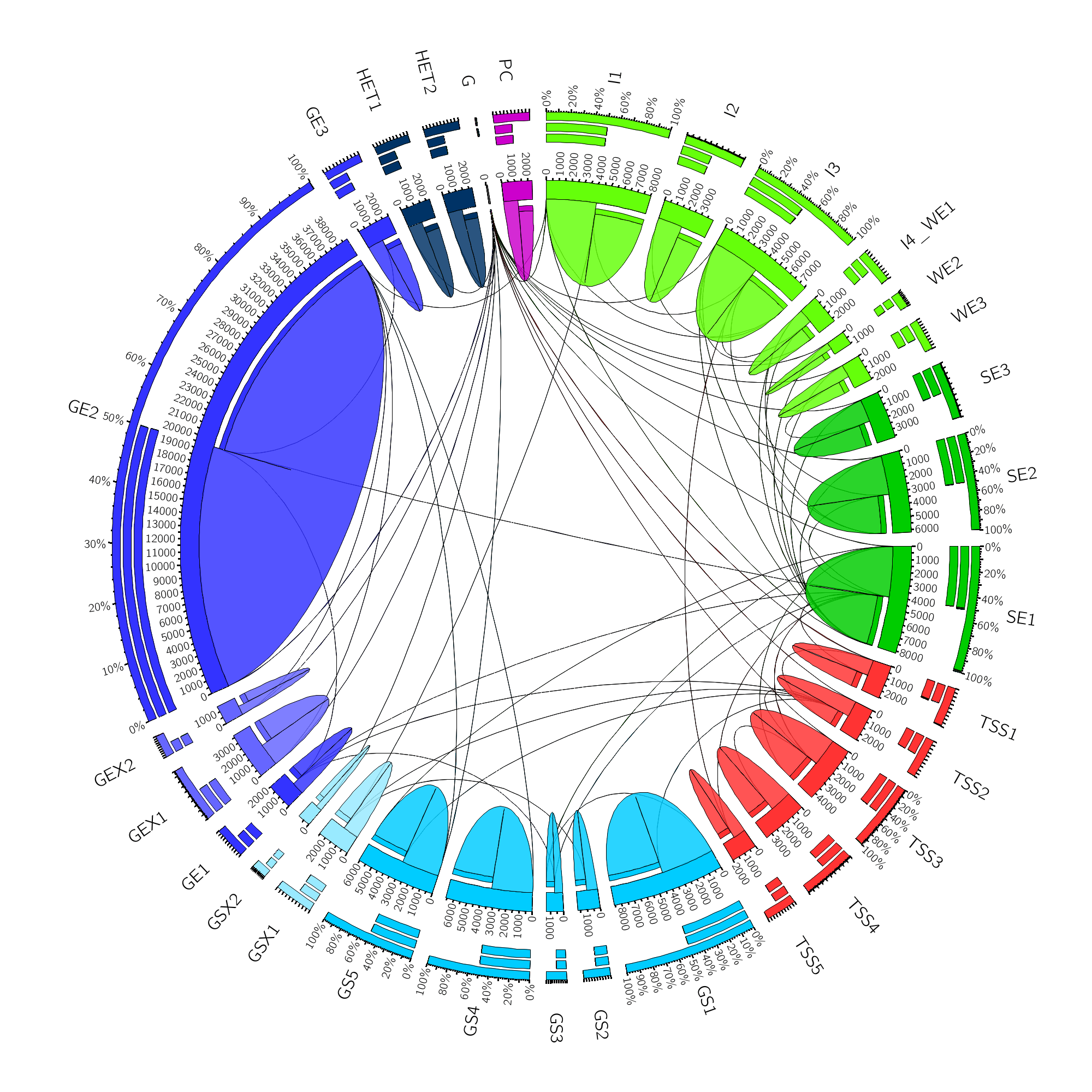

Supplement: Supplementary Data 4 — Effects of positive and negative perturbations of single chromatin factors on chromatin state identity. [file ncomms10528-s5.zip › Supplementary Data 4/PositivePerturbation/modmdg4.png]

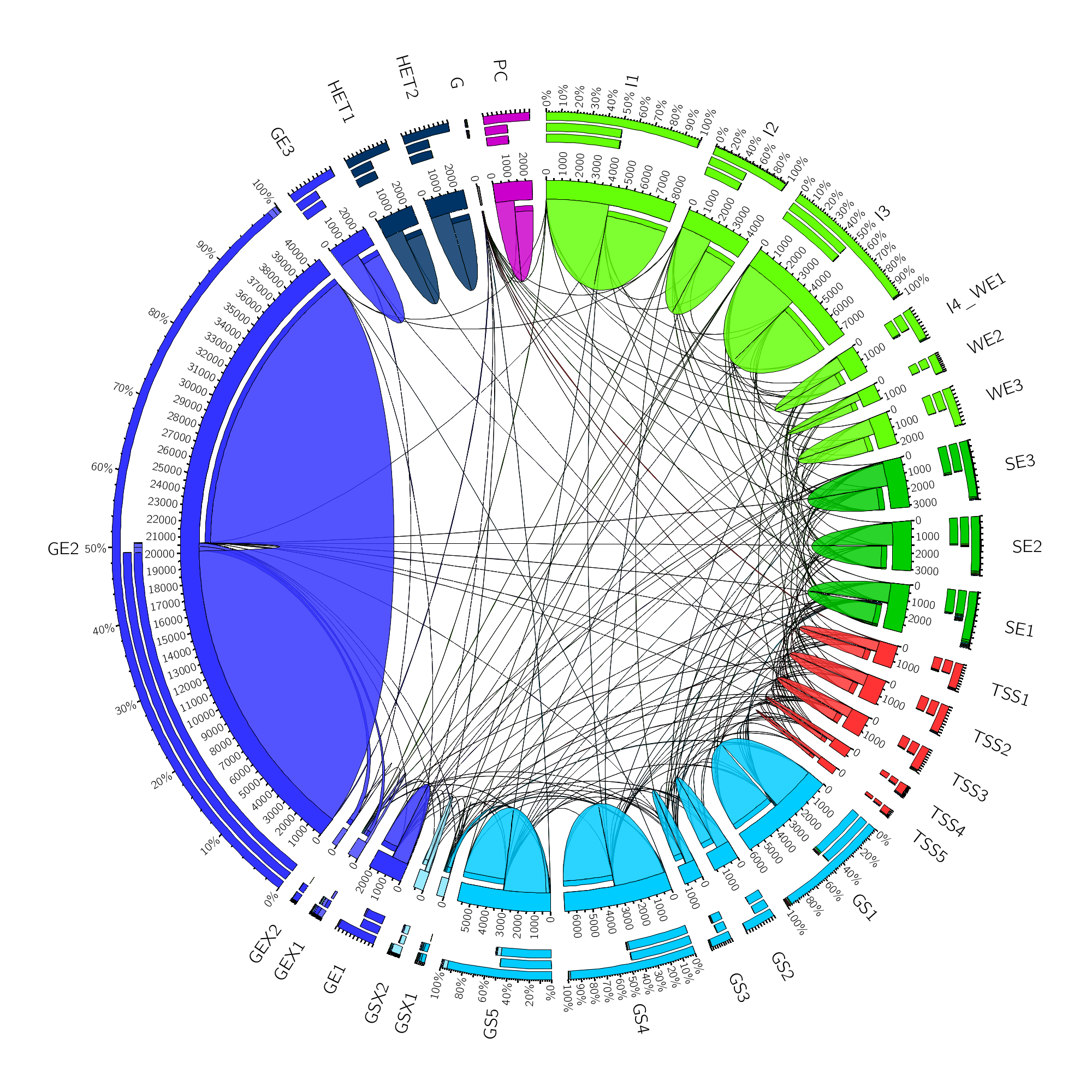

Supplement: Supplementary Data 4 — Effects of positive and negative perturbations of single chromatin factors on chromatin state identity. [file ncomms10528-s5.zip › Supplementary Data 4/PositivePerturbation/MOF.png]

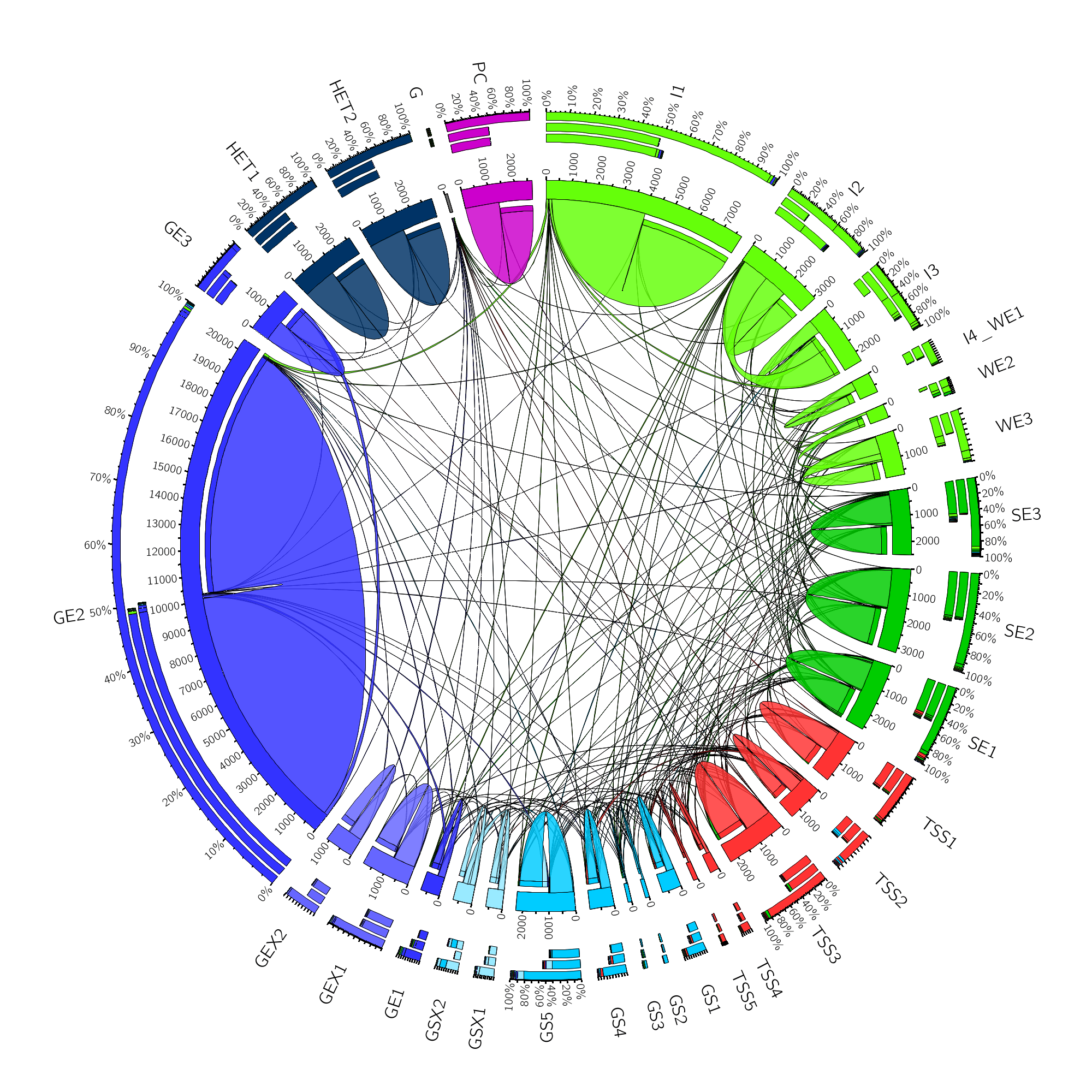

Supplement: Supplementary Data 4 — Effects of positive and negative perturbations of single chromatin factors on chromatin state identity. [file ncomms10528-s5.zip › Supplementary Data 4/PositivePerturbation/MRG15.png]

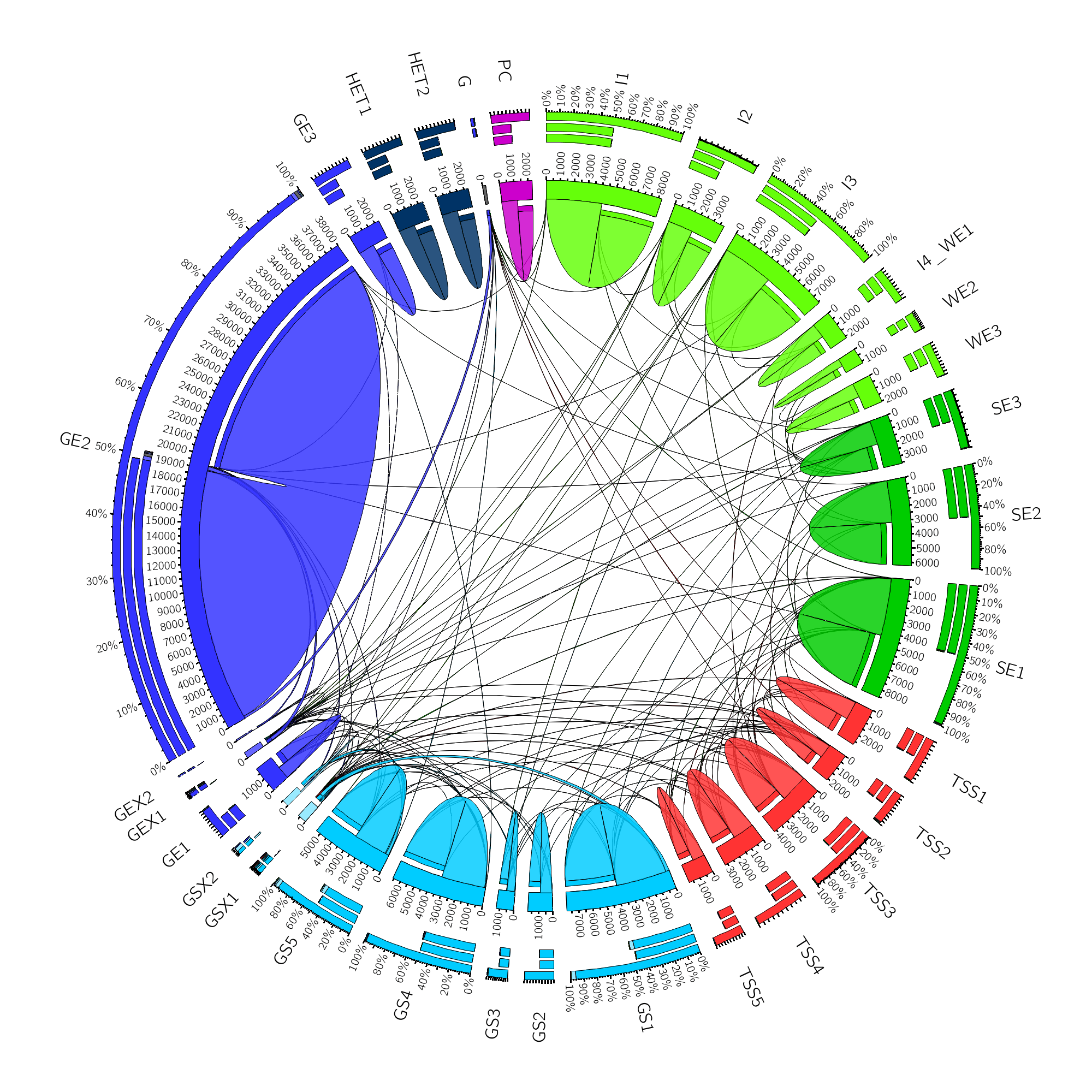

Supplement: Supplementary Data 4 — Effects of positive and negative perturbations of single chromatin factors on chromatin state identity. [file ncomms10528-s5.zip › Supplementary Data 4/PositivePerturbation/MSL1.png]

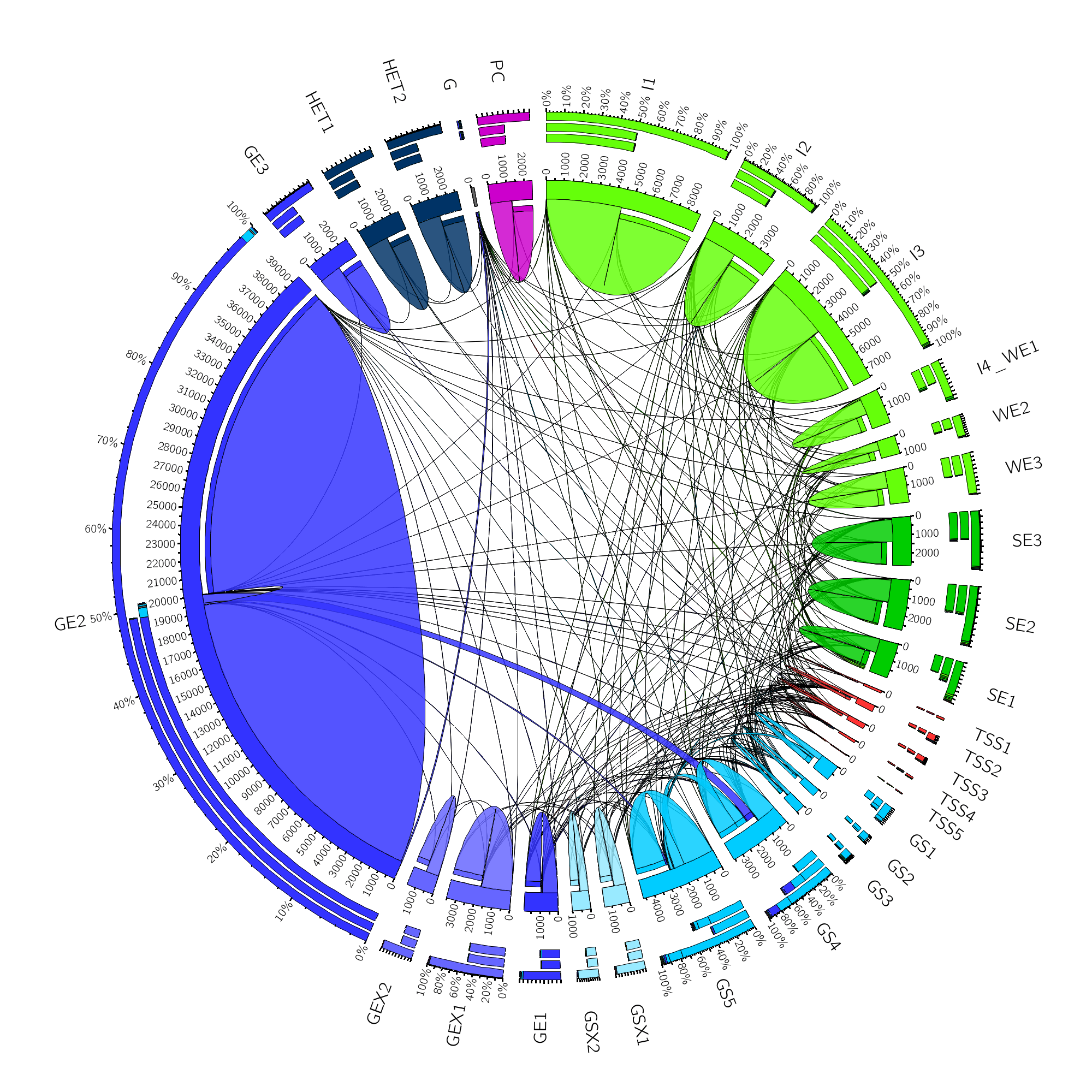

Supplement: Supplementary Data 4 — Effects of positive and negative perturbations of single chromatin factors on chromatin state identity. [file ncomms10528-s5.zip › Supplementary Data 4/PositivePerturbation/NURF301.png]

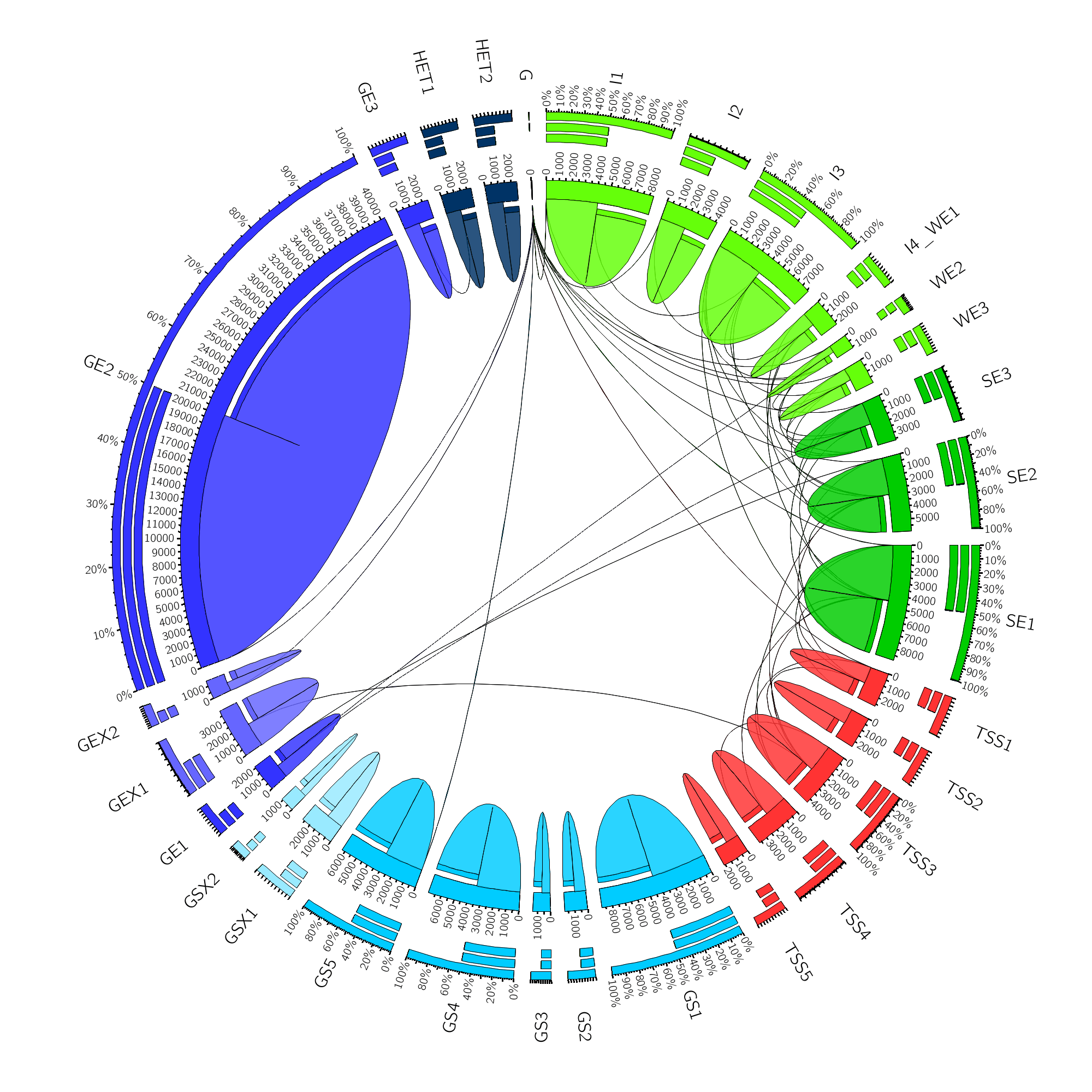

Supplement: Supplementary Data 4 — Effects of positive and negative perturbations of single chromatin factors on chromatin state identity. [file ncomms10528-s5.zip › Supplementary Data 4/PositivePerturbation/Pc.png]

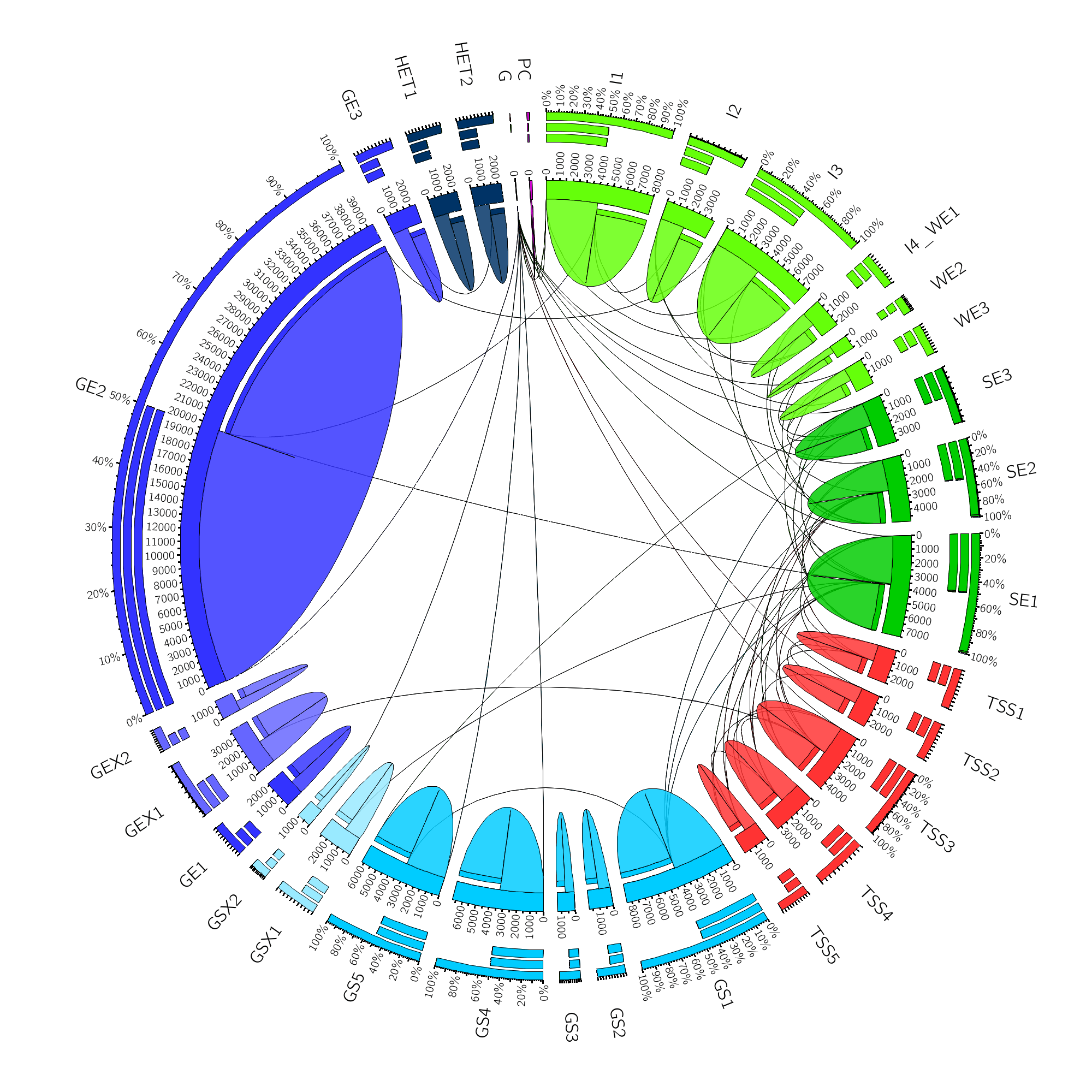

Supplement: Supplementary Data 4 — Effects of positive and negative perturbations of single chromatin factors on chromatin state identity. [file ncomms10528-s5.zip › Supplementary Data 4/PositivePerturbation/PCL.png]

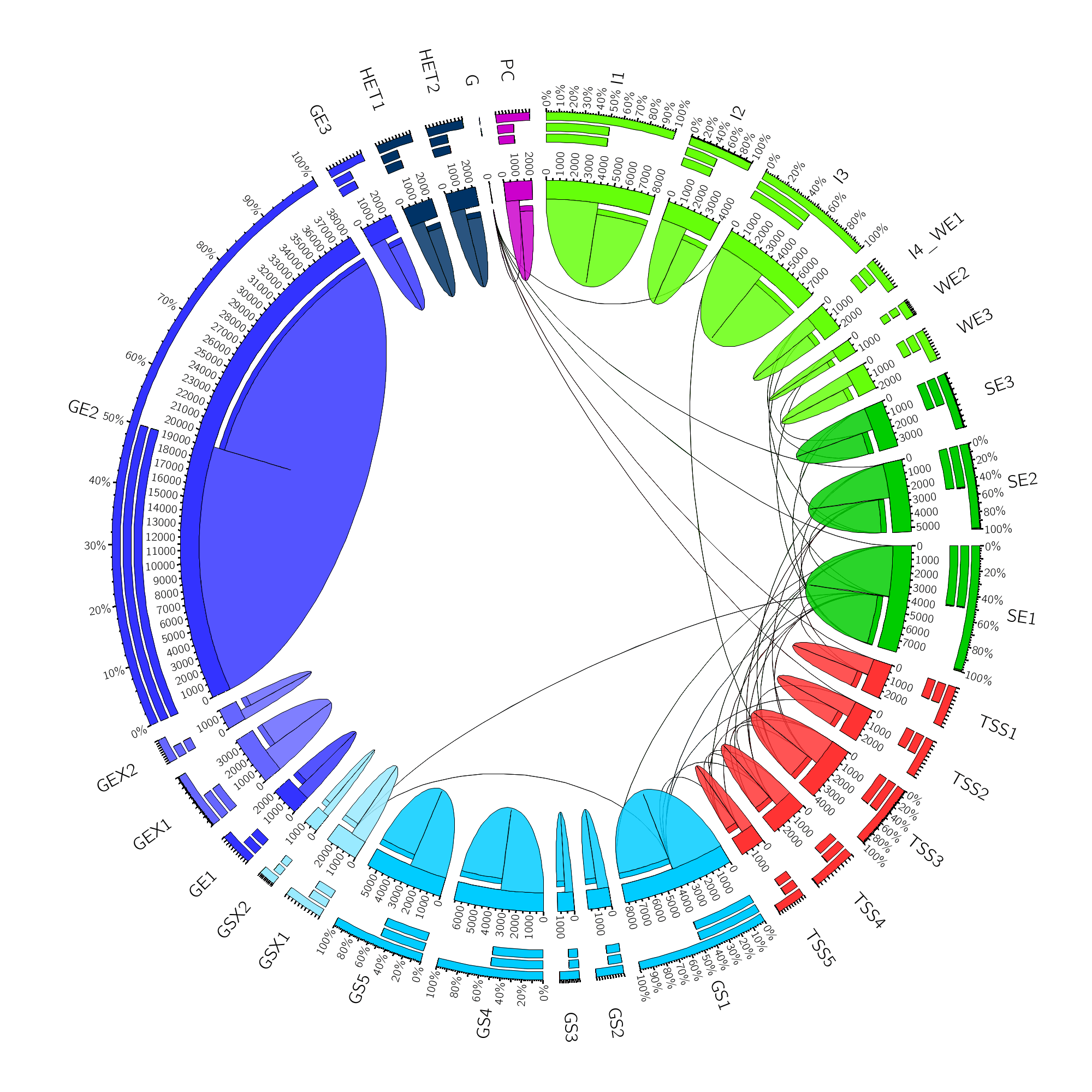

Supplement: Supplementary Data 4 — Effects of positive and negative perturbations of single chromatin factors on chromatin state identity. [file ncomms10528-s5.zip › Supplementary Data 4/PositivePerturbation/Pho.png]

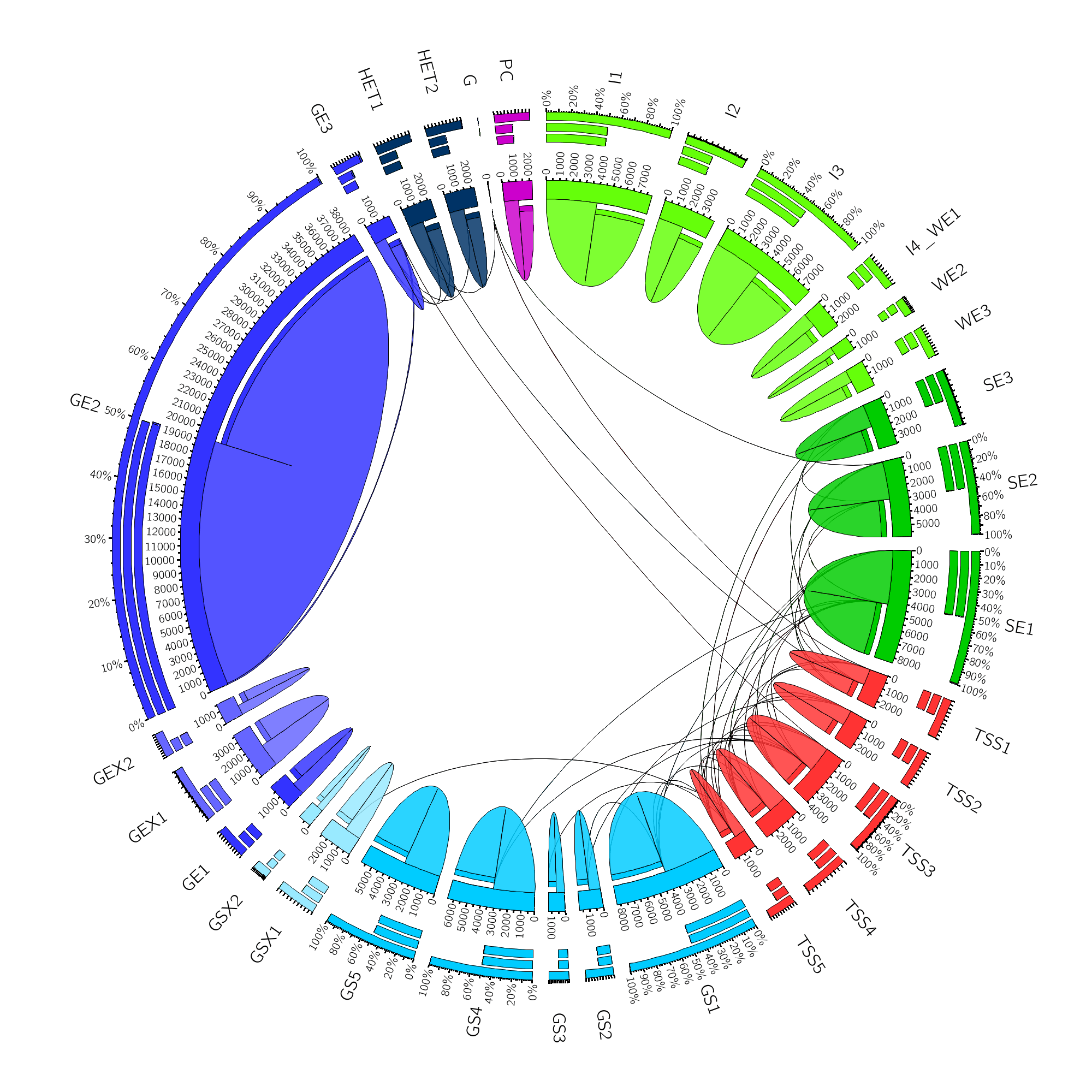

Supplement: Supplementary Data 4 — Effects of positive and negative perturbations of single chromatin factors on chromatin state identity. [file ncomms10528-s5.zip › Supplementary Data 4/PositivePerturbation/POF.png]

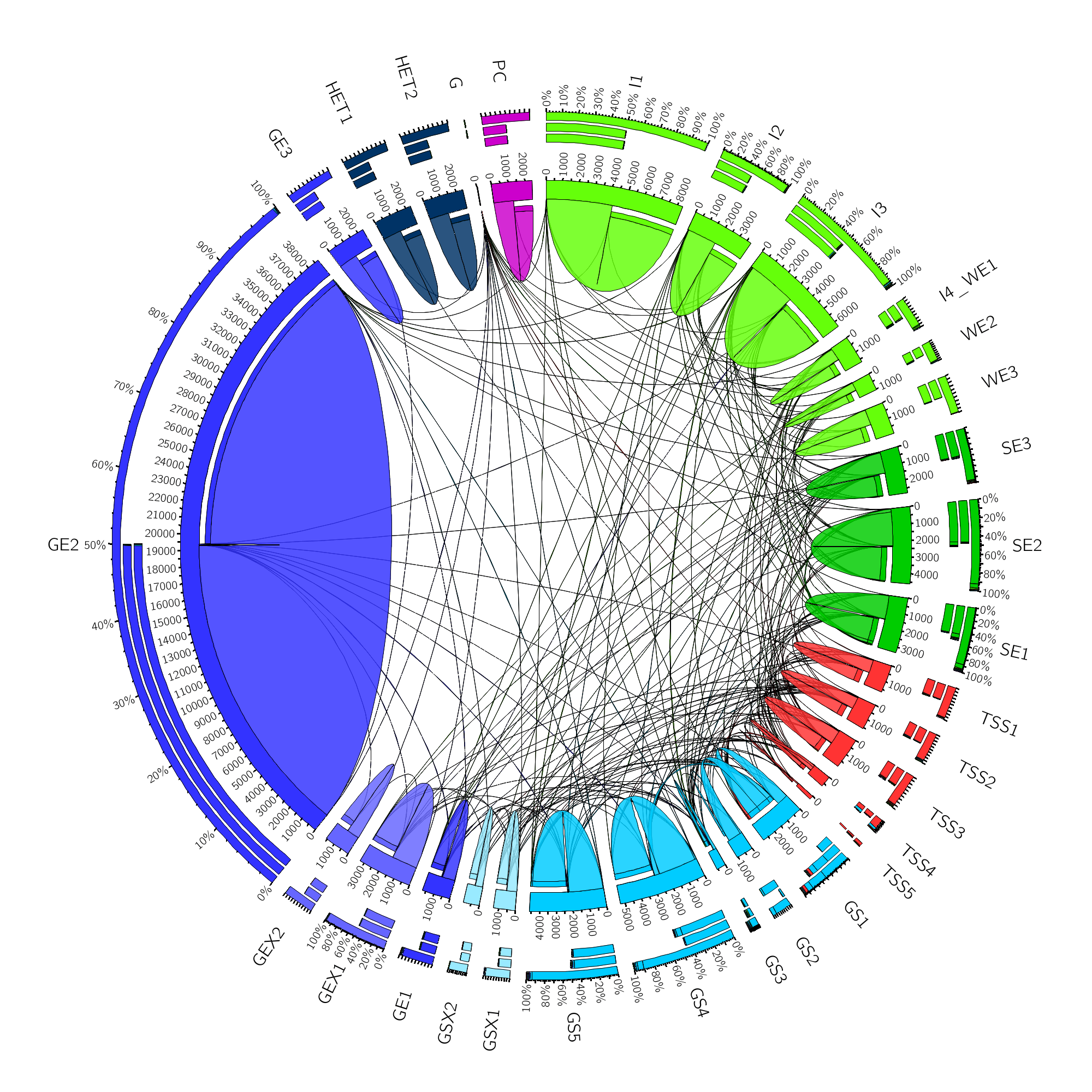

Supplement: Supplementary Data 4 — Effects of positive and negative perturbations of single chromatin factors on chromatin state identity. [file ncomms10528-s5.zip › Supplementary Data 4/PositivePerturbation/pol2.png]

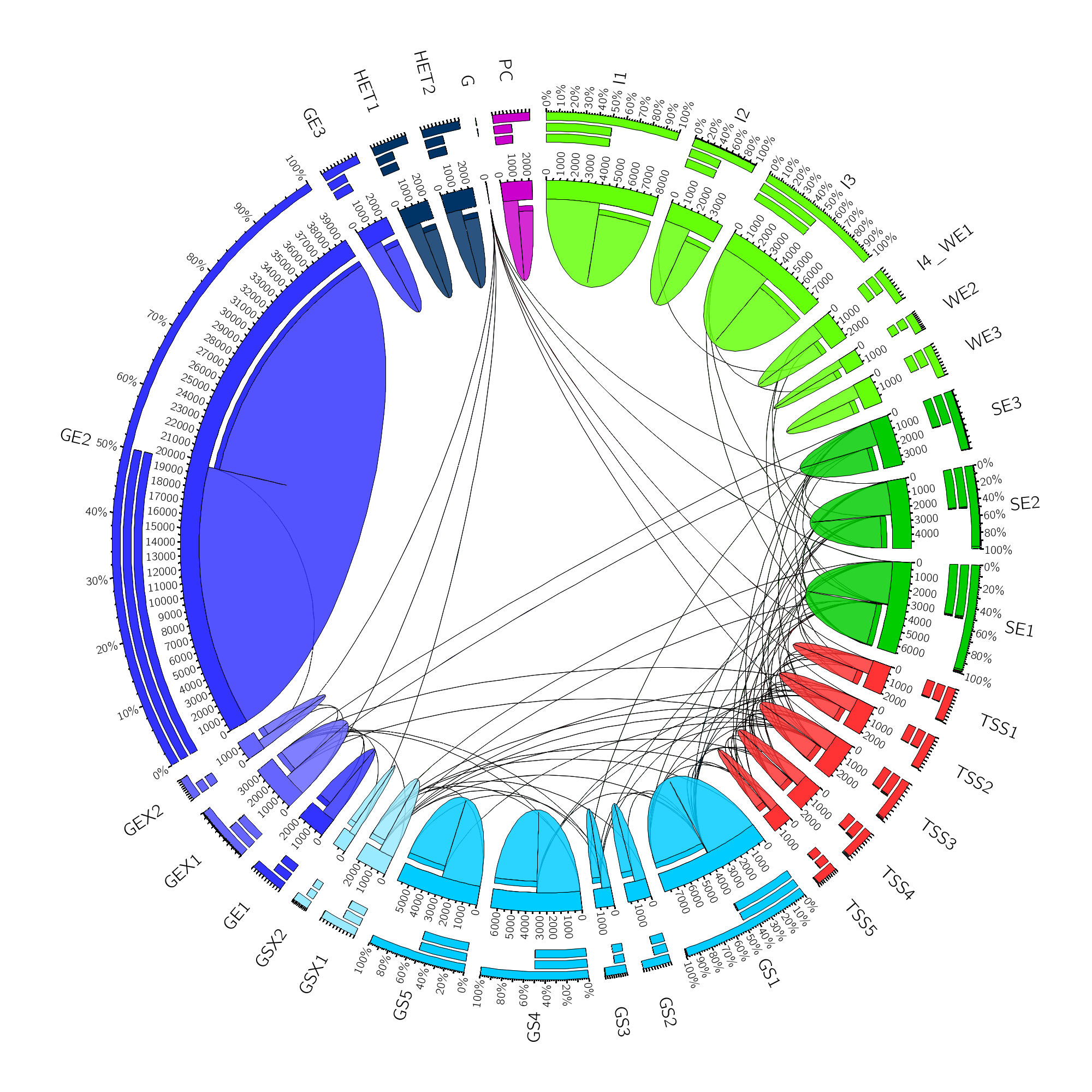

Supplement: Supplementary Data 4 — Effects of positive and negative perturbations of single chromatin factors on chromatin state identity. [file ncomms10528-s5.zip › Supplementary Data 4/PositivePerturbation/PRSet7.png]

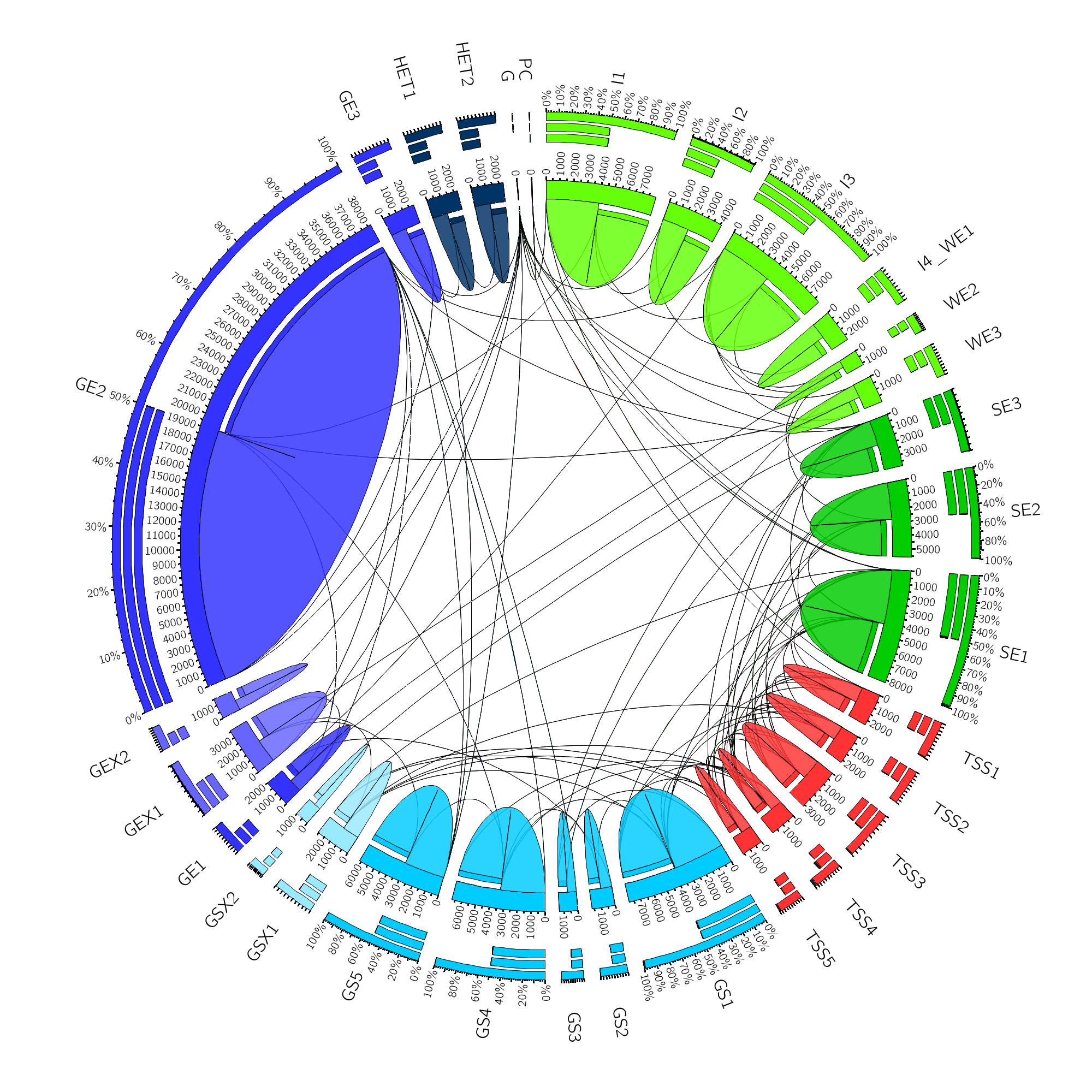

Supplement: Supplementary Data 4 — Effects of positive and negative perturbations of single chromatin factors on chromatin state identity. [file ncomms10528-s5.zip › Supplementary Data 4/PositivePerturbation/Psc.png]

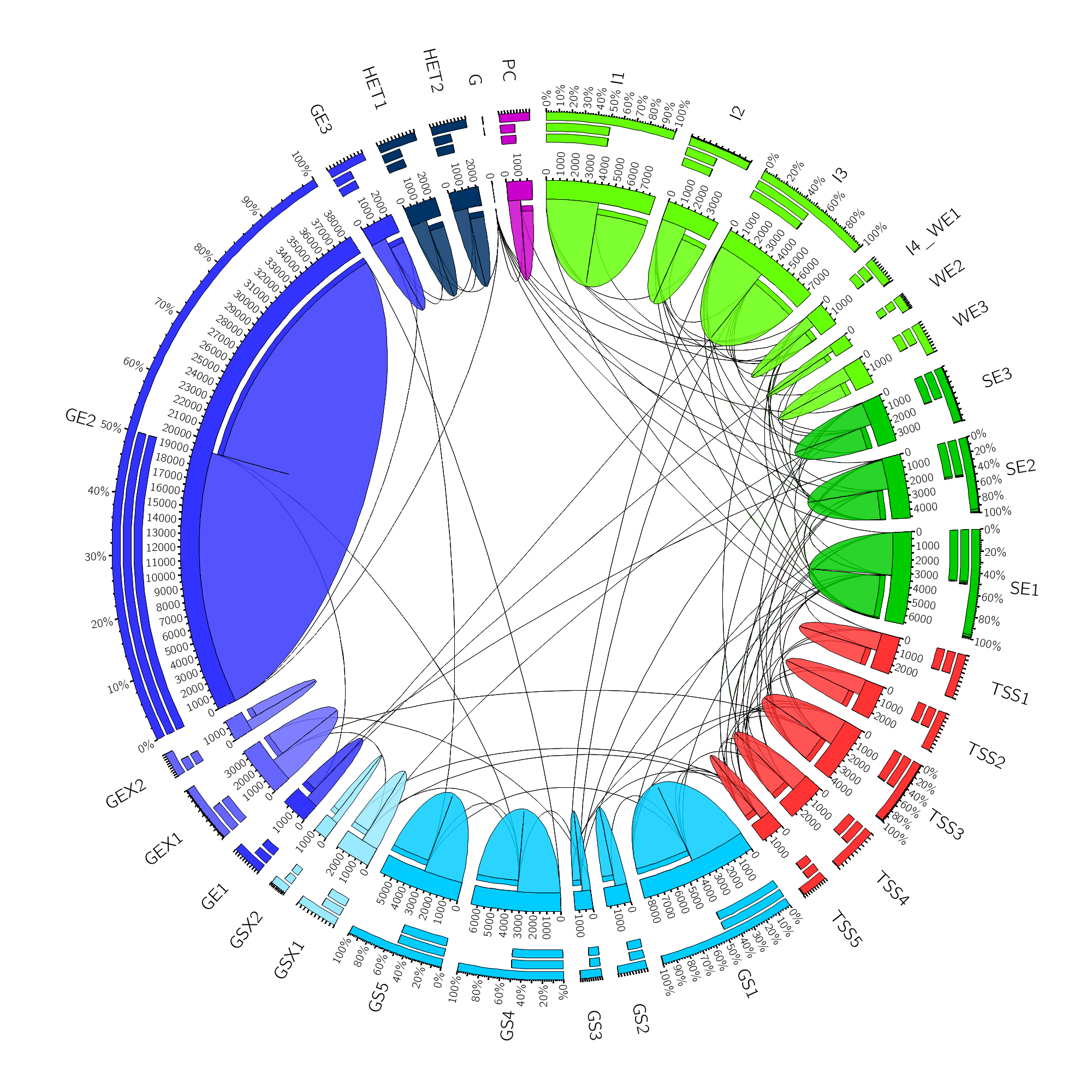

Supplement: Supplementary Data 4 — Effects of positive and negative perturbations of single chromatin factors on chromatin state identity. [file ncomms10528-s5.zip › Supplementary Data 4/PositivePerturbation/Rhino.png]

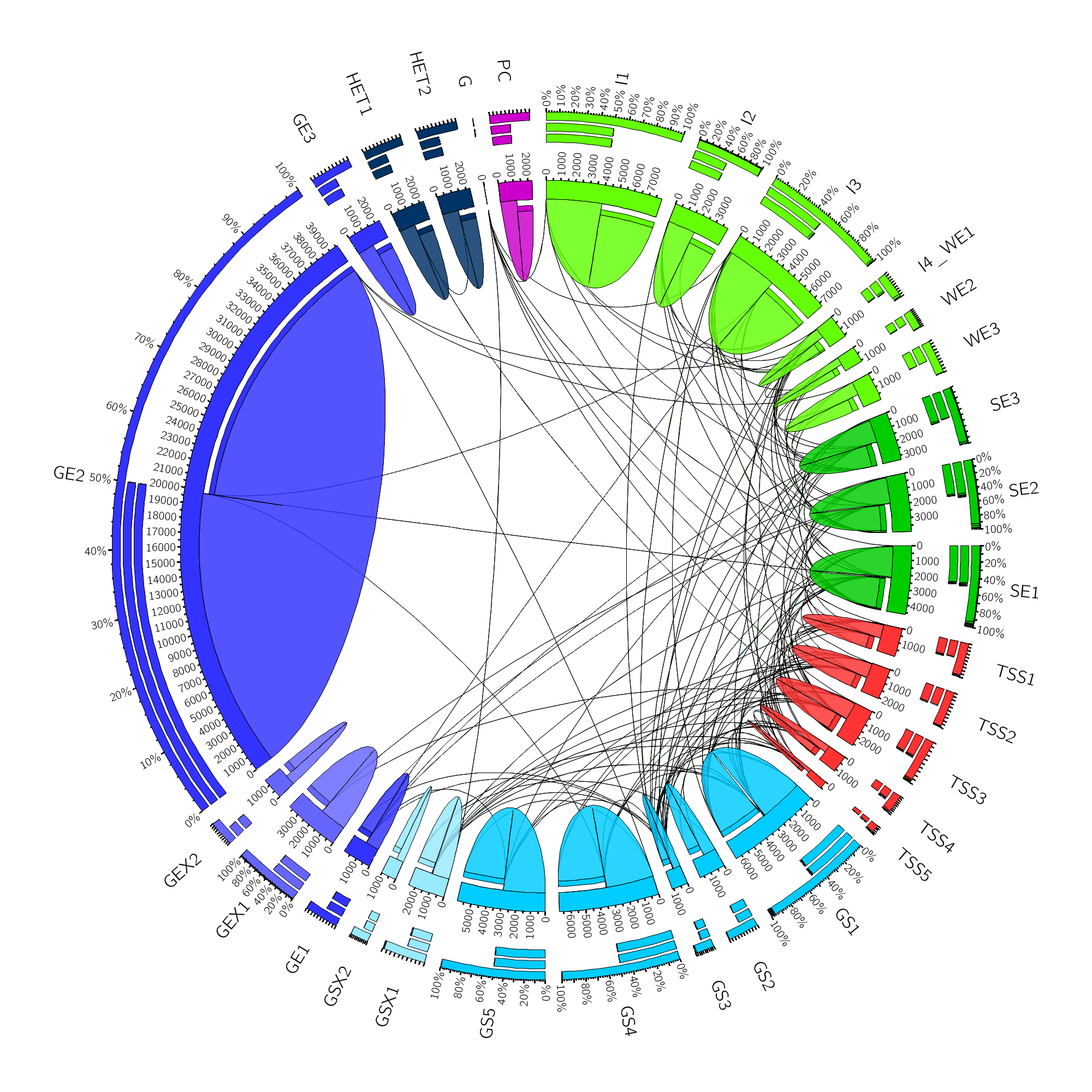

Supplement: Supplementary Data 4 — Effects of positive and negative perturbations of single chromatin factors on chromatin state identity. [file ncomms10528-s5.zip › Supplementary Data 4/PositivePerturbation/RPD3.png]

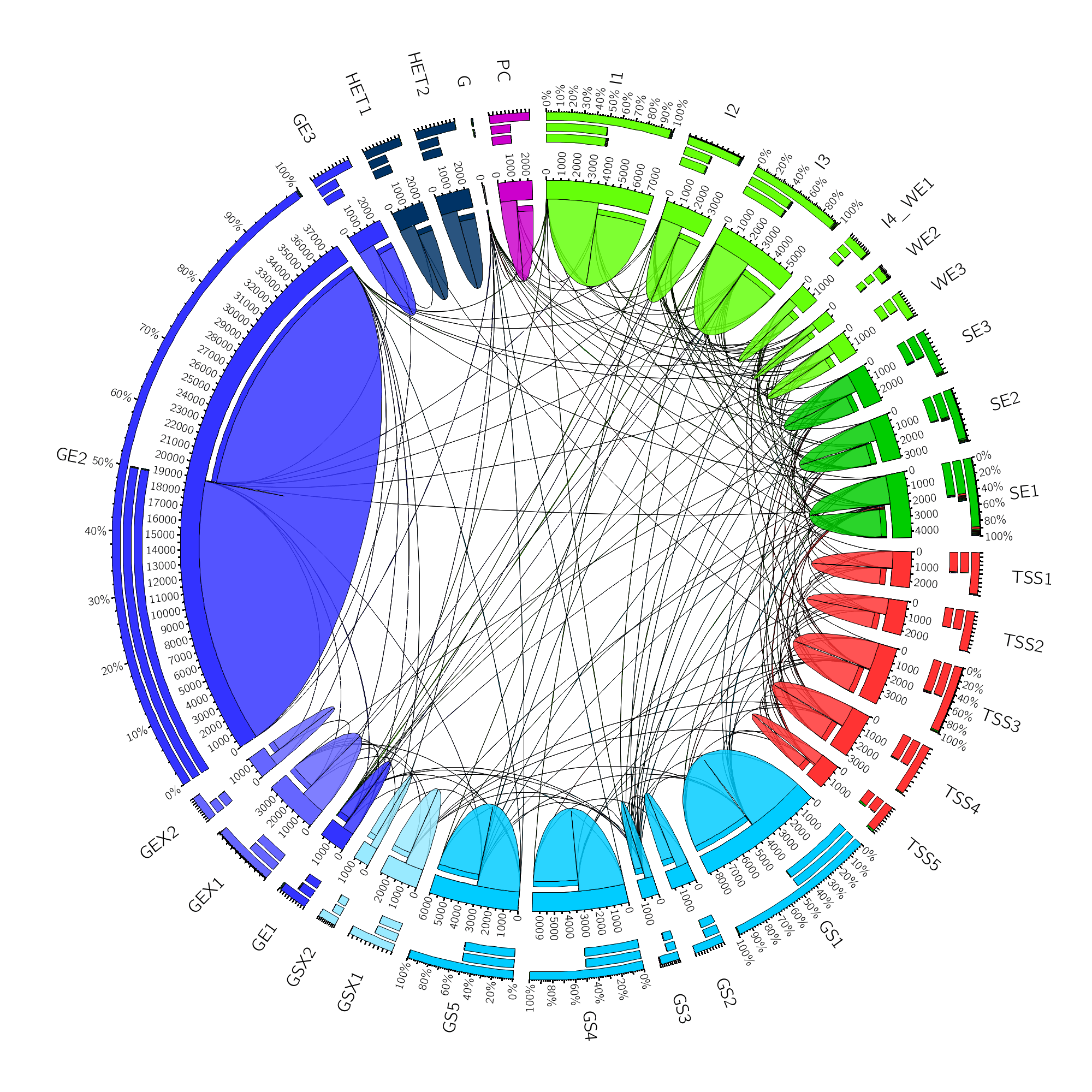

Supplement: Supplementary Data 4 — Effects of positive and negative perturbations of single chromatin factors on chromatin state identity. [file ncomms10528-s5.zip › Supplementary Data 4/PositivePerturbation/Smc3.png]

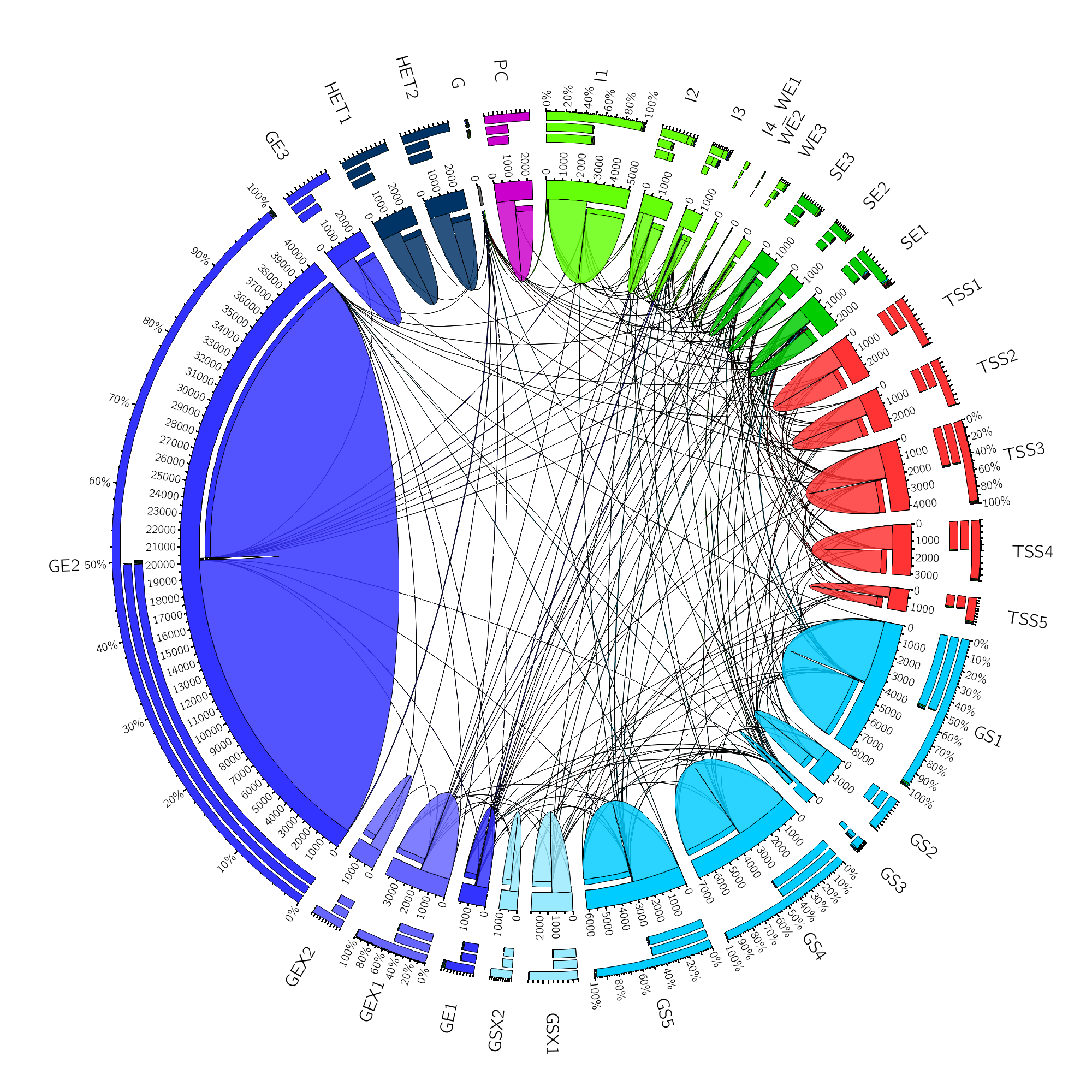

Supplement: Supplementary Data 4 — Effects of positive and negative perturbations of single chromatin factors on chromatin state identity. [file ncomms10528-s5.zip › Supplementary Data 4/PositivePerturbation/SPT16.png]

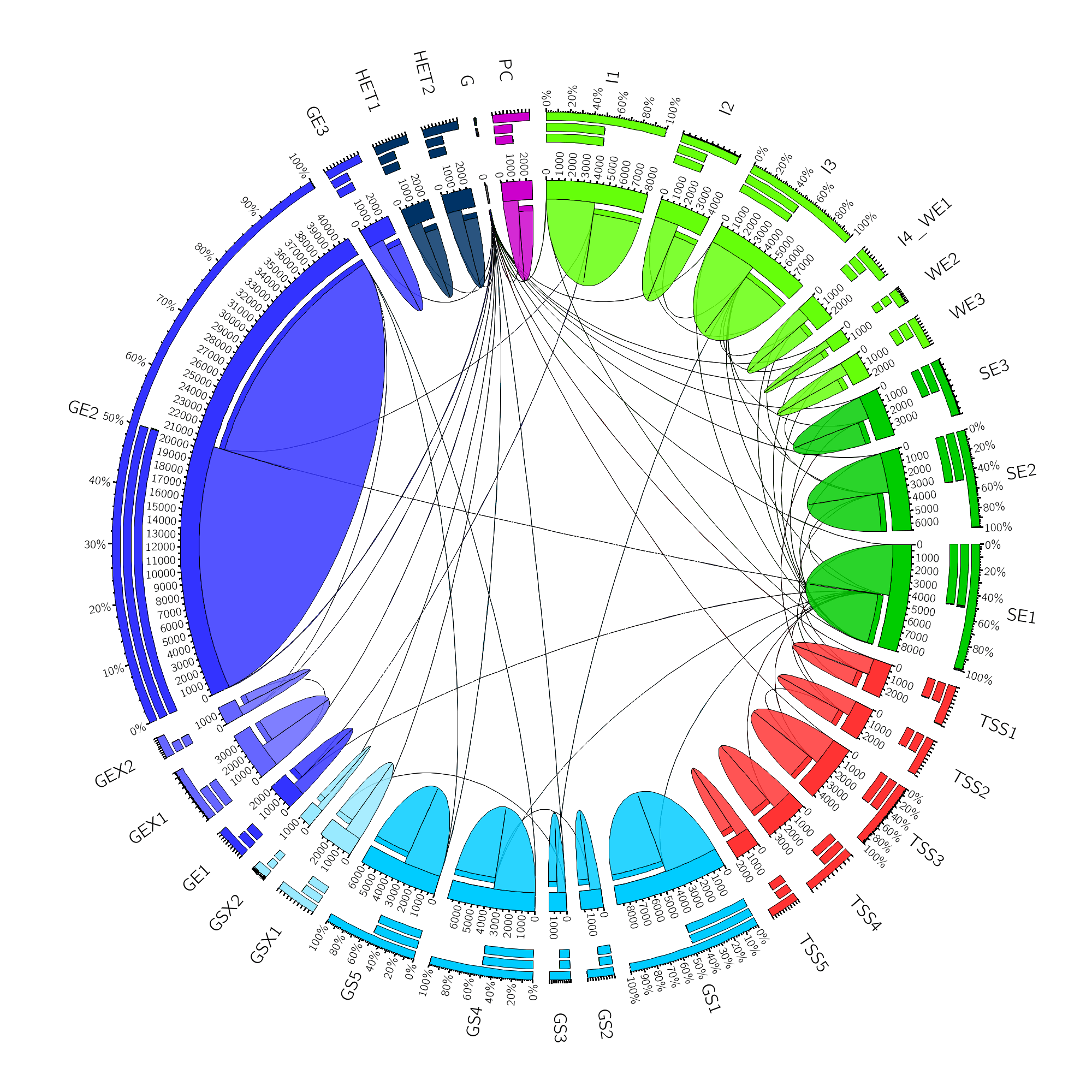

Supplement: Supplementary Data 4 — Effects of positive and negative perturbations of single chromatin factors on chromatin state identity. [file ncomms10528-s5.zip › Supplementary Data 4/PositivePerturbation/SuHw.png]

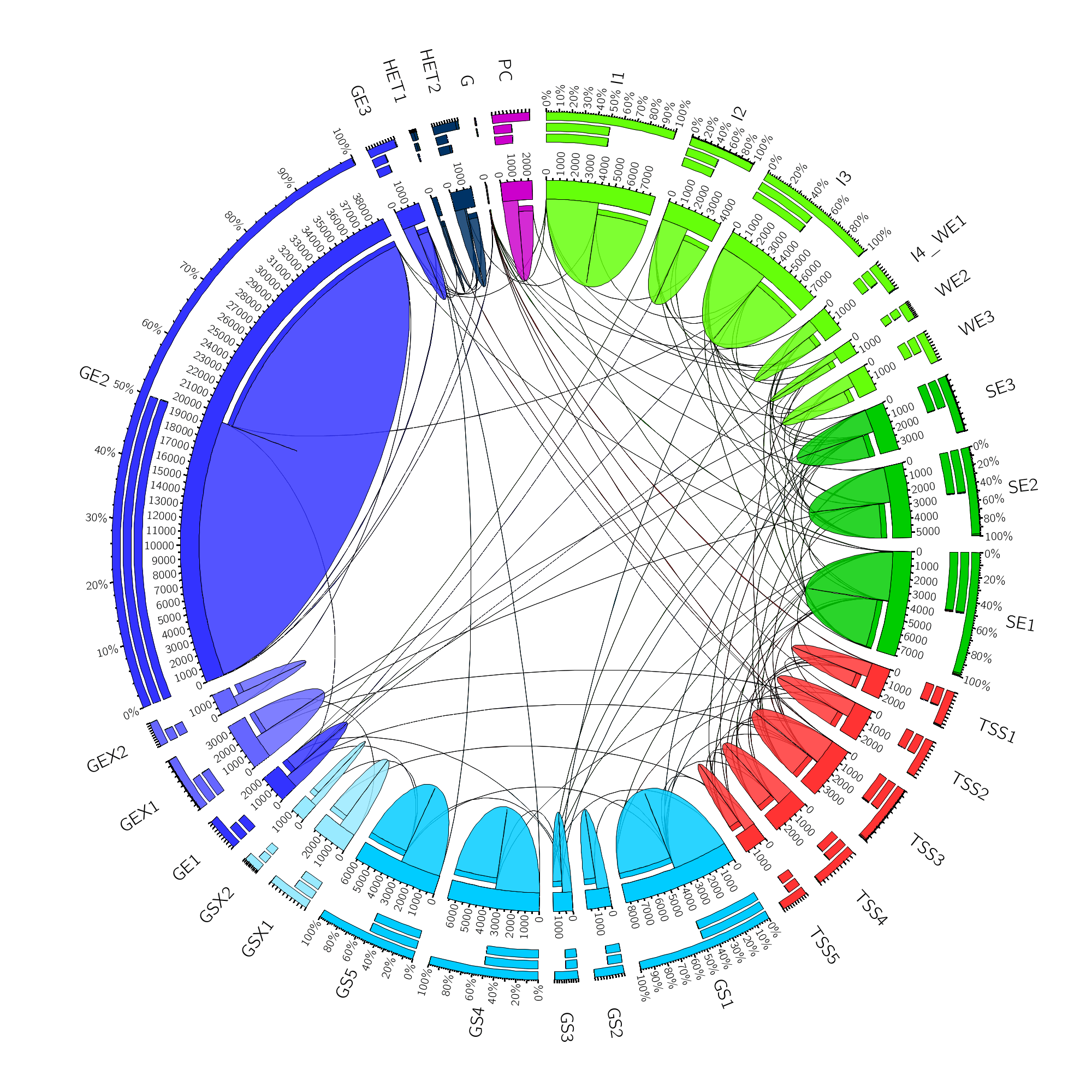

Supplement: Supplementary Data 4 — Effects of positive and negative perturbations of single chromatin factors on chromatin state identity. [file ncomms10528-s5.zip › Supplementary Data 4/PositivePerturbation/Suvar37.png]

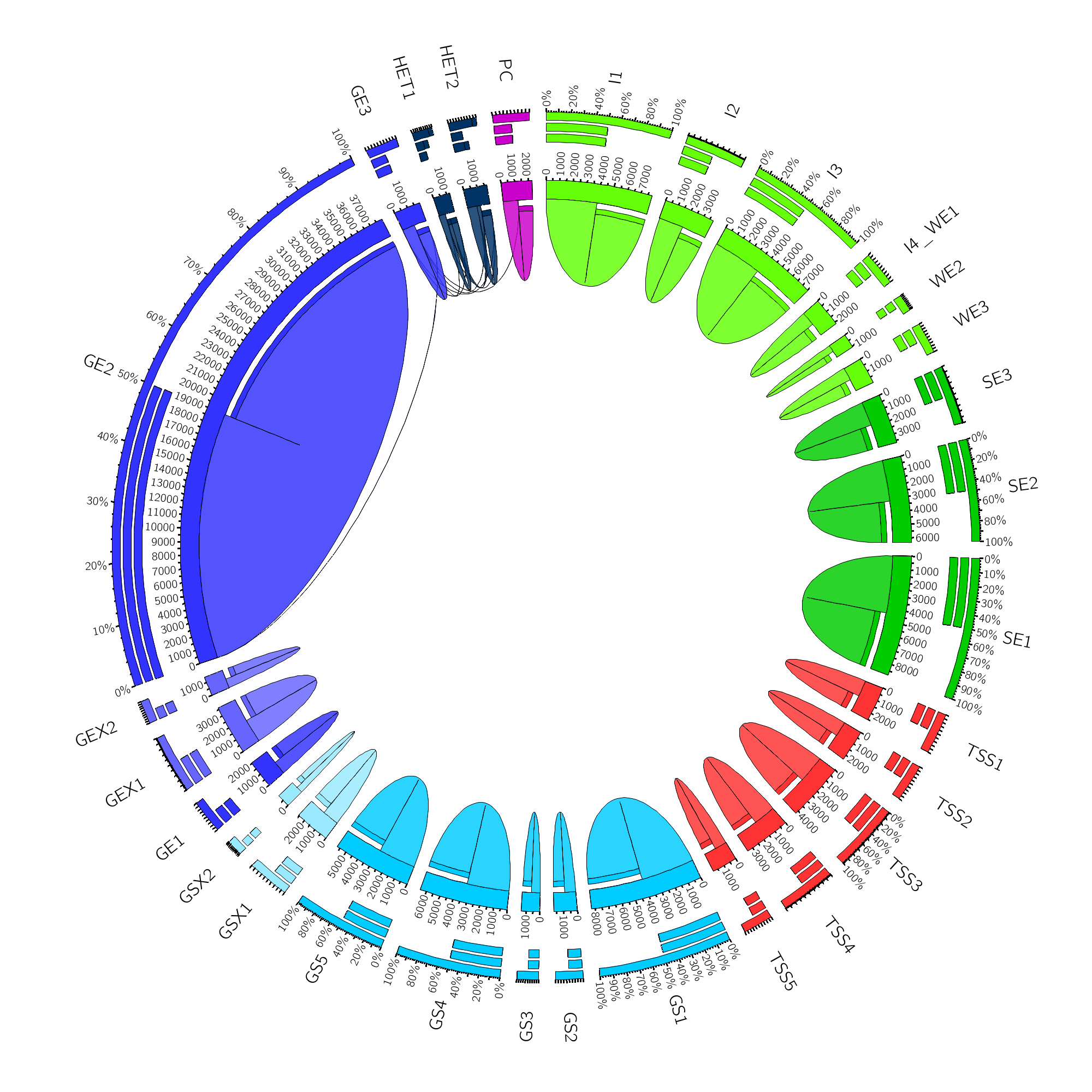

Supplement: Supplementary Data 4 — Effects of positive and negative perturbations of single chromatin factors on chromatin state identity. [file ncomms10528-s5.zip › Supplementary Data 4/PositivePerturbation/Suvar39.png]

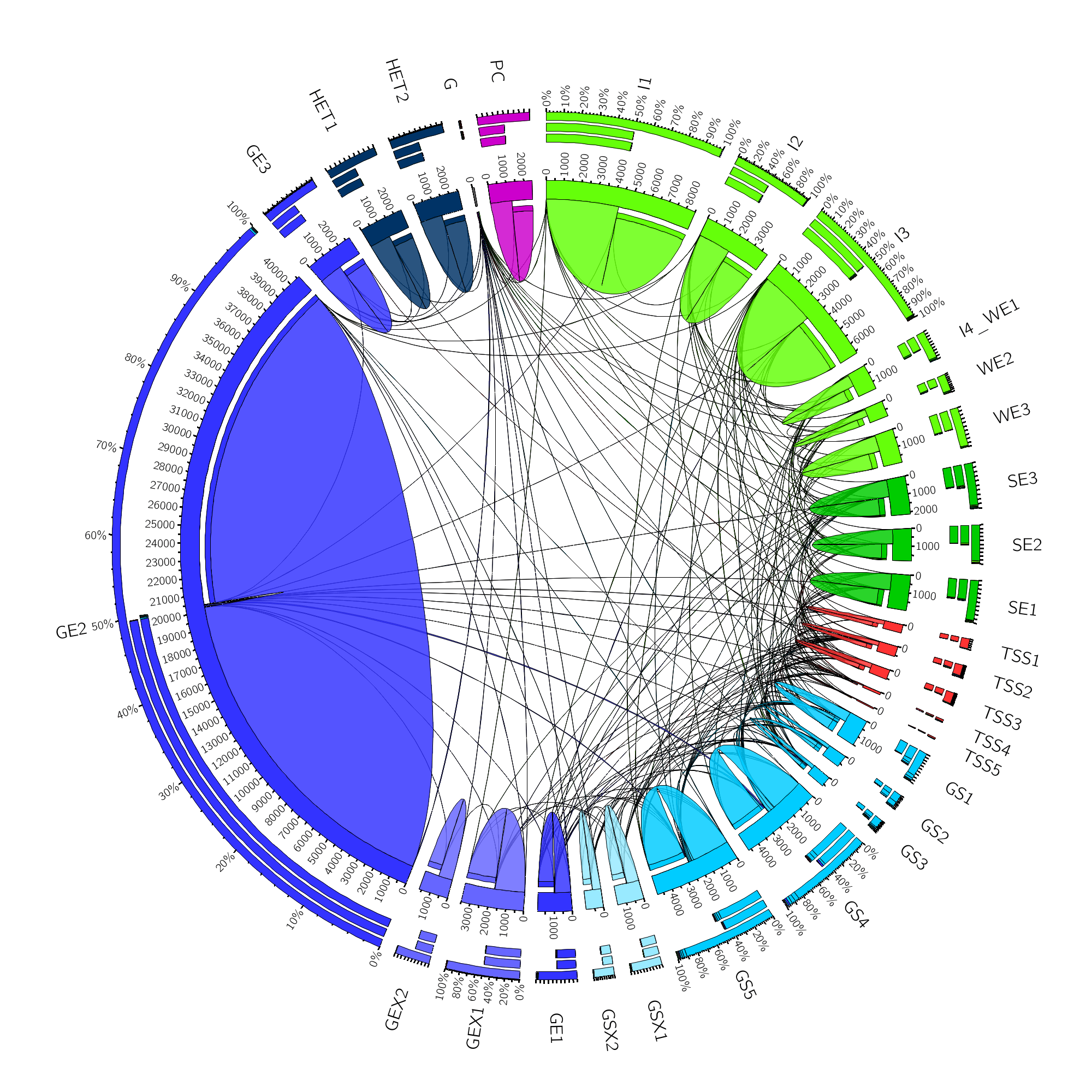

Supplement: Supplementary Data 4 — Effects of positive and negative perturbations of single chromatin factors on chromatin state identity. [file ncomms10528-s5.zip › Supplementary Data 4/PositivePerturbation/WDS.png]

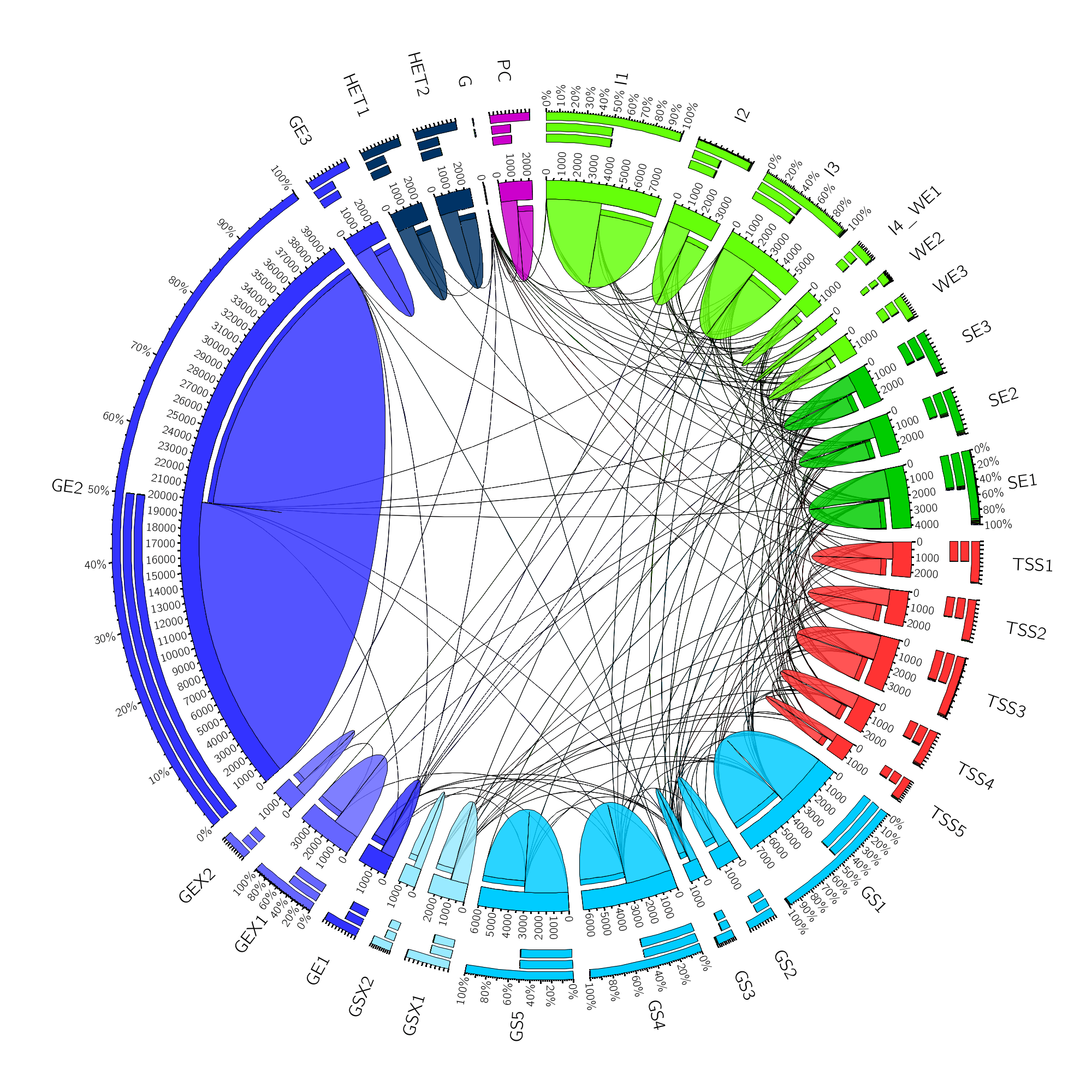

Supplement: Supplementary Data 4 — Effects of positive and negative perturbations of single chromatin factors on chromatin state identity. [file ncomms10528-s5.zip › Supplementary Data 4/PositivePerturbation/ZW5.png]
