# Supplementary figures and images for: Probabilistic modelling of chromatin code landscape reveals functional diversity of enhancer-like chromatin states (part 1 of 2)
Source: Nat Commun. 2016 Feb 4;7:10528. doi: 10.1038/ncomms10528 (PMC4742914; doi:10.1038/ncomms10528)

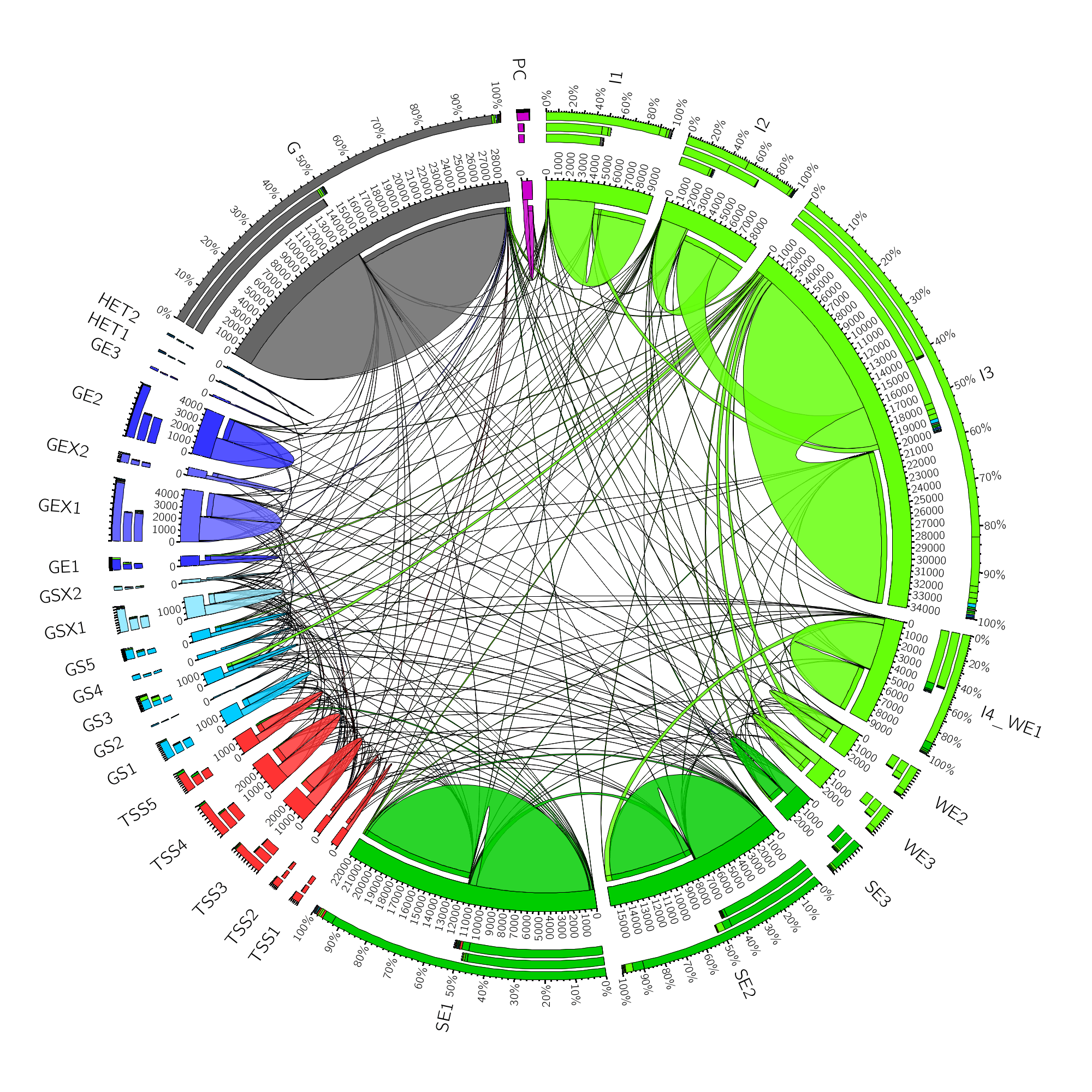

Supplement: Supplementary Data 4 — Effects of positive and negative perturbations of single chromatin factors on chromatin state identity. [file ncomms10528-s5.zip › Supplementary Data 4/NegativePerturbation/ASH1.png]

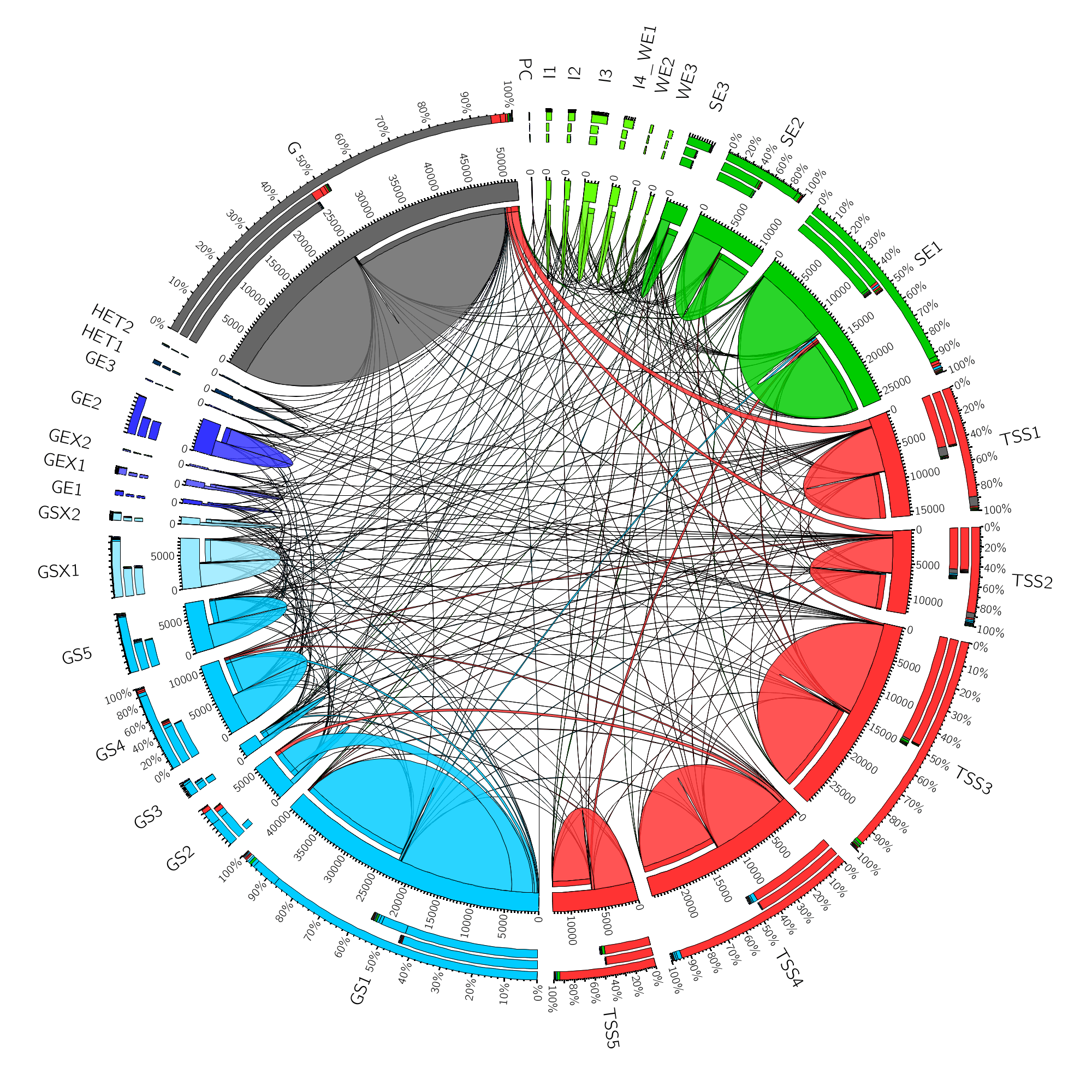

Supplement: Supplementary Data 4 — Effects of positive and negative perturbations of single chromatin factors on chromatin state identity. [file ncomms10528-s5.zip › Supplementary Data 4/NegativePerturbation/BEAF32.png]

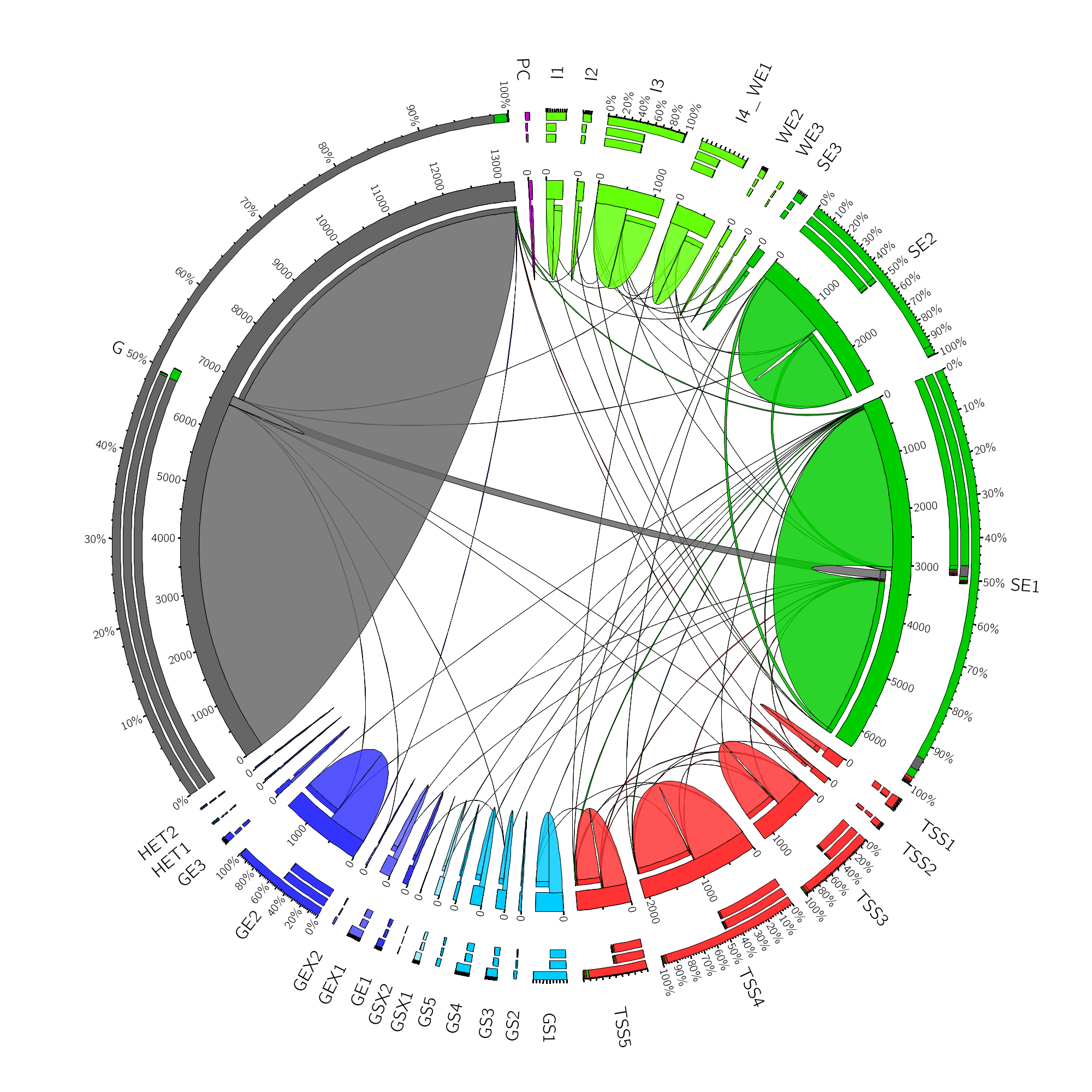

Supplement: Supplementary Data 4 — Effects of positive and negative perturbations of single chromatin factors on chromatin state identity. [file ncomms10528-s5.zip › Supplementary Data 4/NegativePerturbation/CG10630.png]

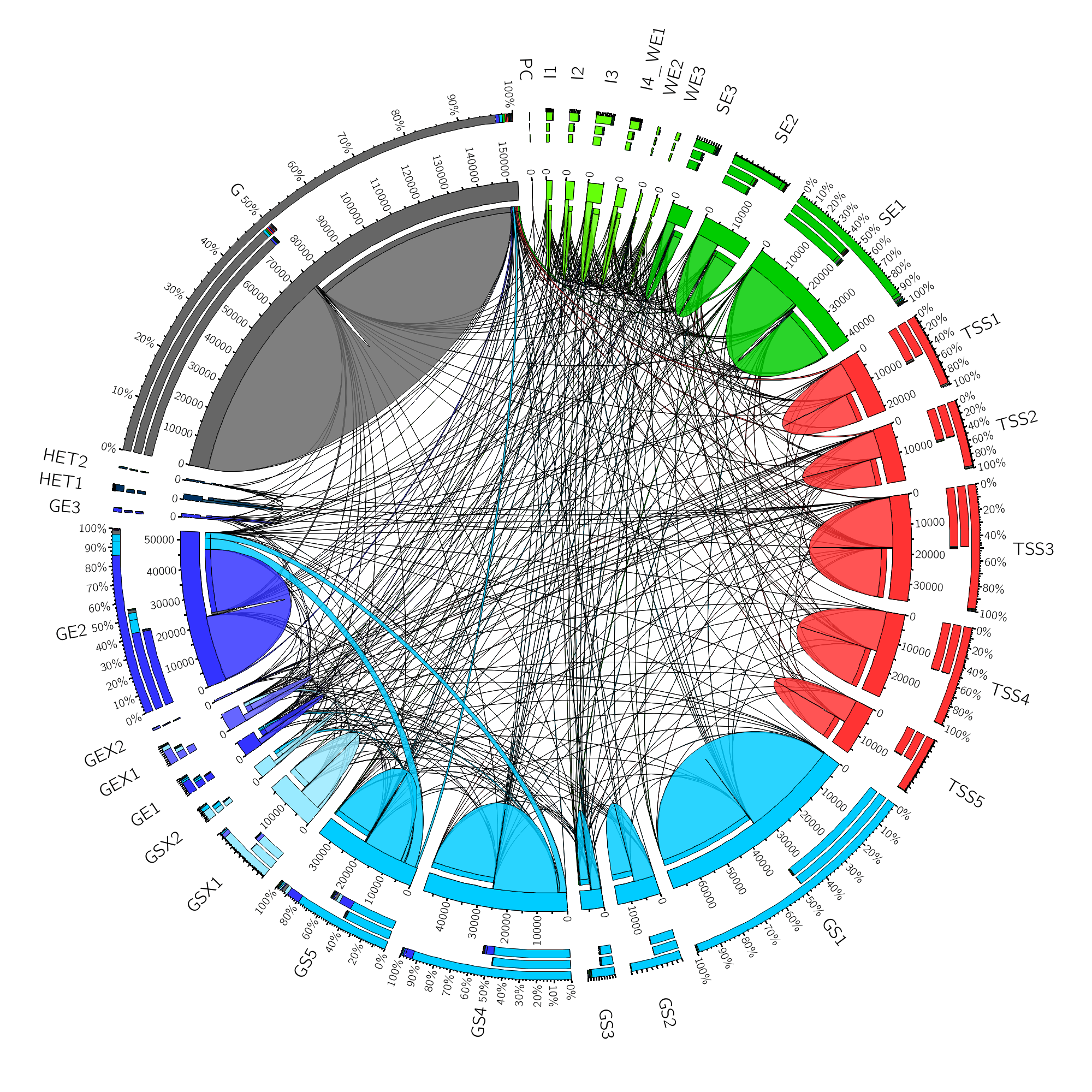

Supplement: Supplementary Data 4 — Effects of positive and negative perturbations of single chromatin factors on chromatin state identity. [file ncomms10528-s5.zip › Supplementary Data 4/NegativePerturbation/Chriz.png]

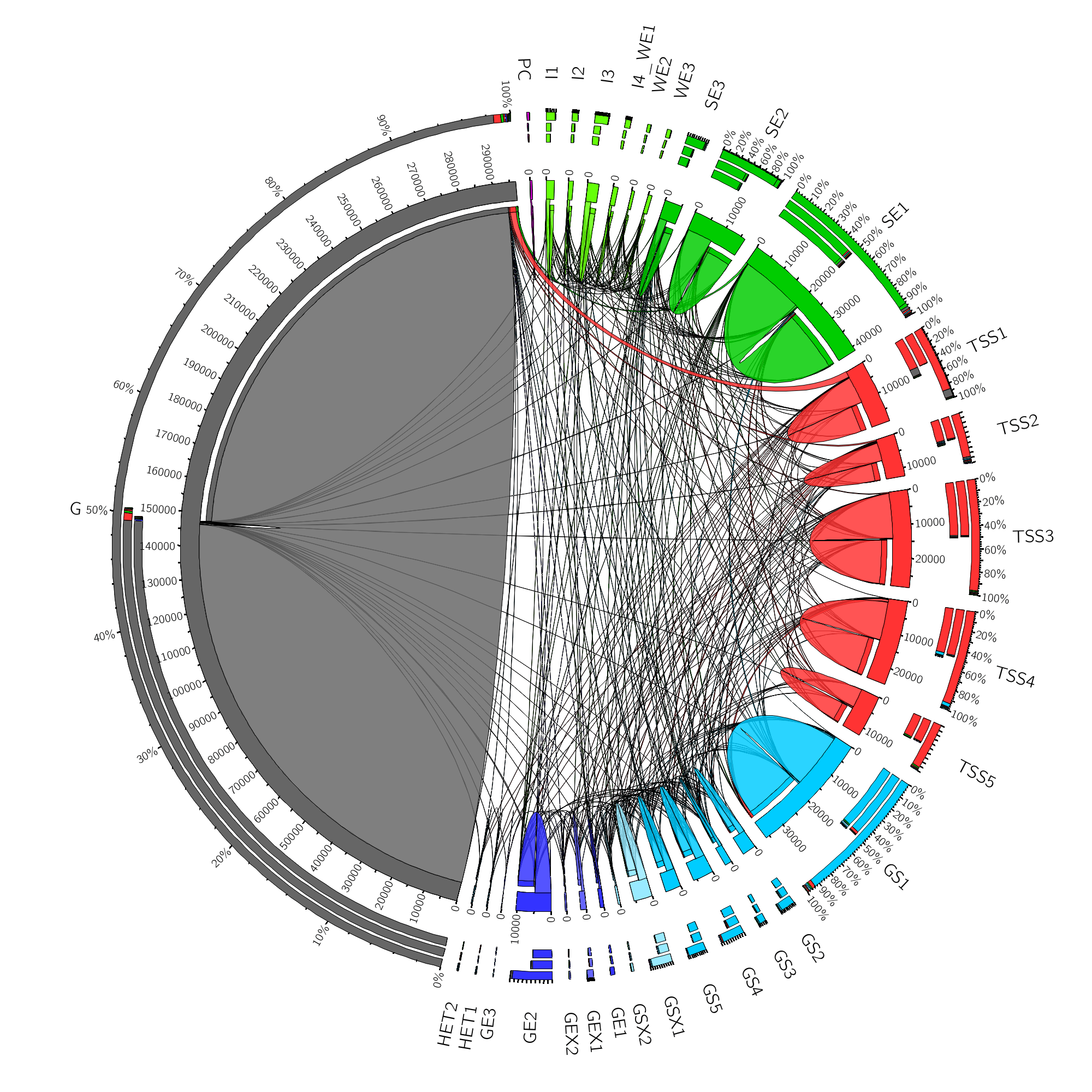

Supplement: Supplementary Data 4 — Effects of positive and negative perturbations of single chromatin factors on chromatin state identity. [file ncomms10528-s5.zip › Supplementary Data 4/NegativePerturbation/CP190.png]

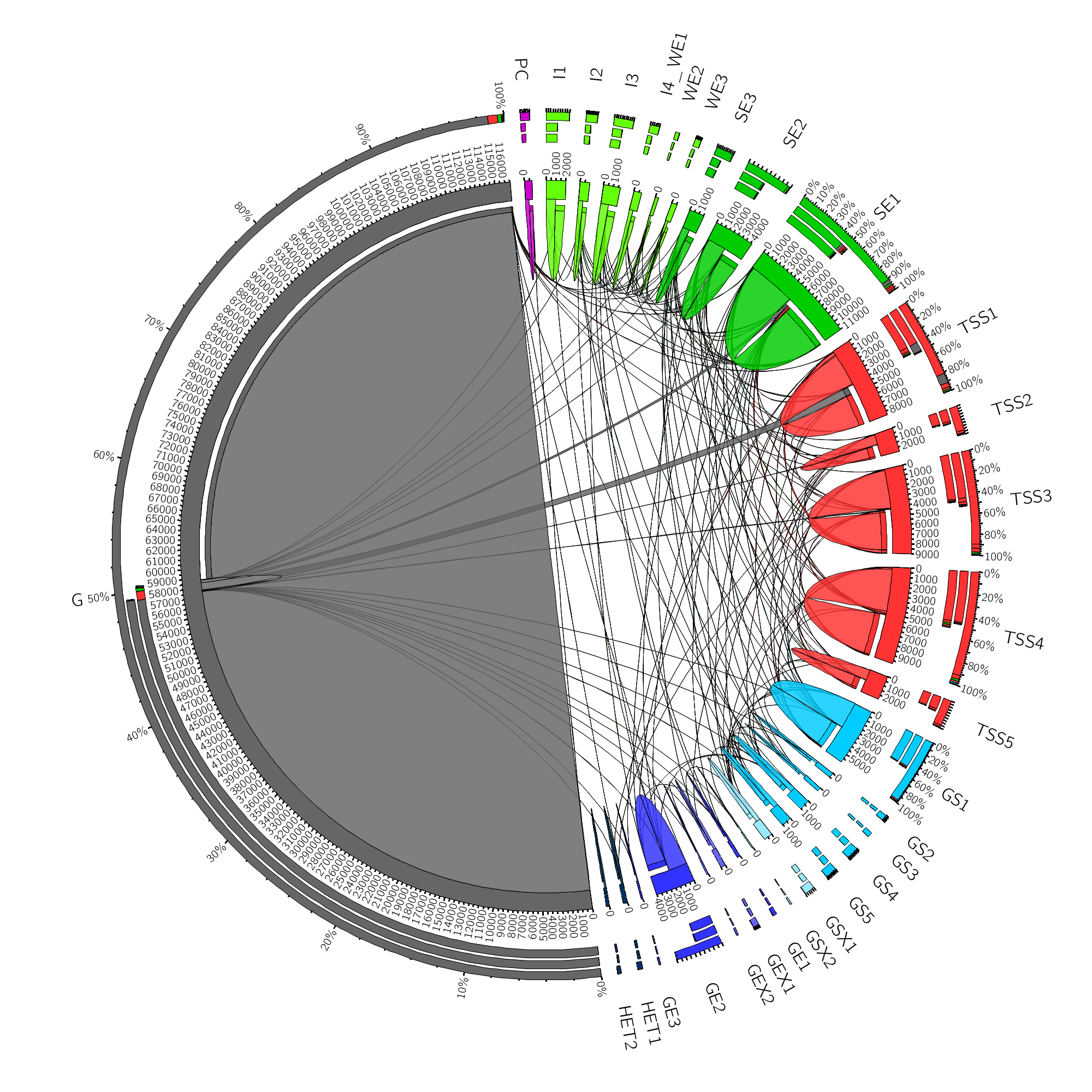

Supplement: Supplementary Data 4 — Effects of positive and negative perturbations of single chromatin factors on chromatin state identity. [file ncomms10528-s5.zip › Supplementary Data 4/NegativePerturbation/CTCF.png]

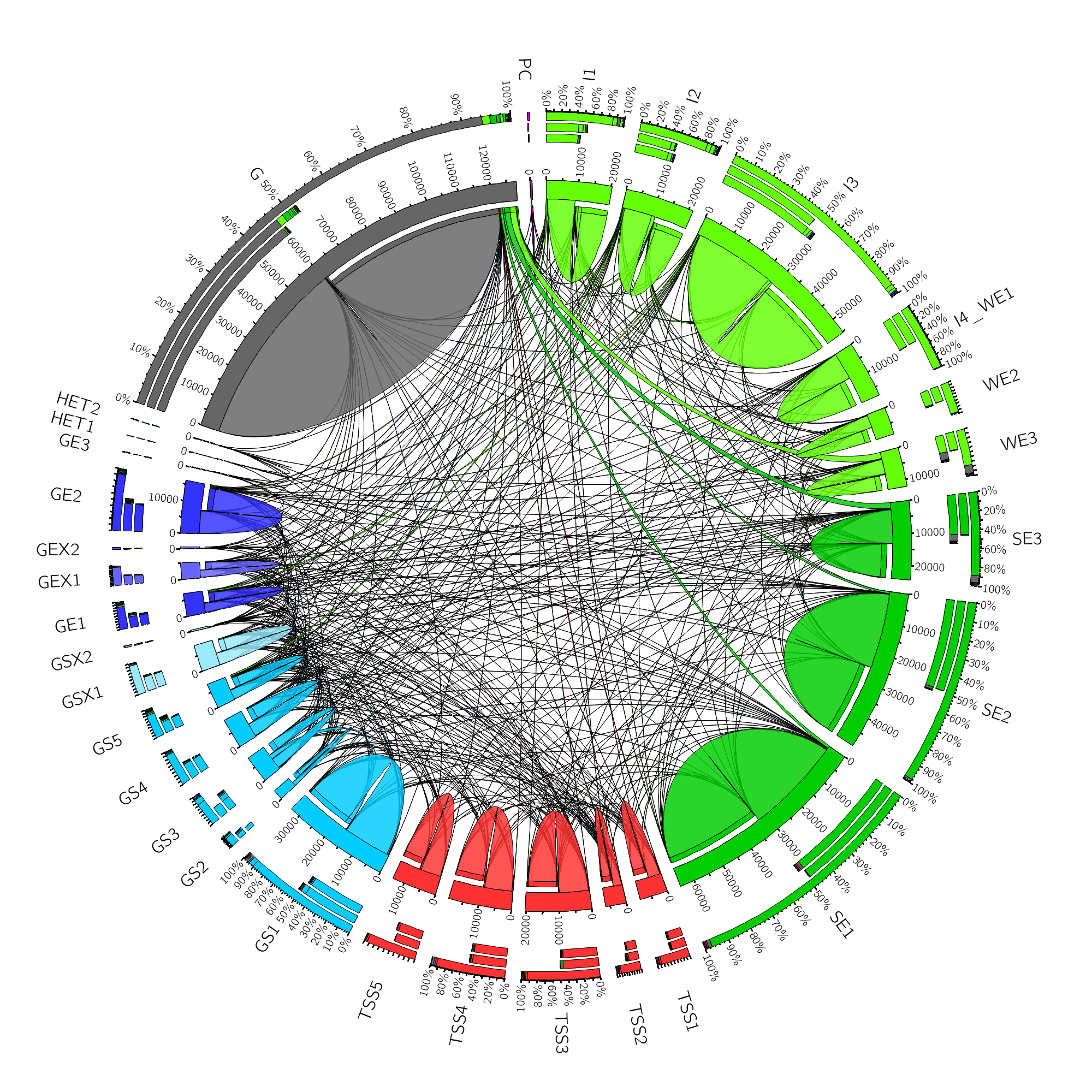

Supplement: Supplementary Data 4 — Effects of positive and negative perturbations of single chromatin factors on chromatin state identity. [file ncomms10528-s5.zip › Supplementary Data 4/NegativePerturbation/dMi2.png]

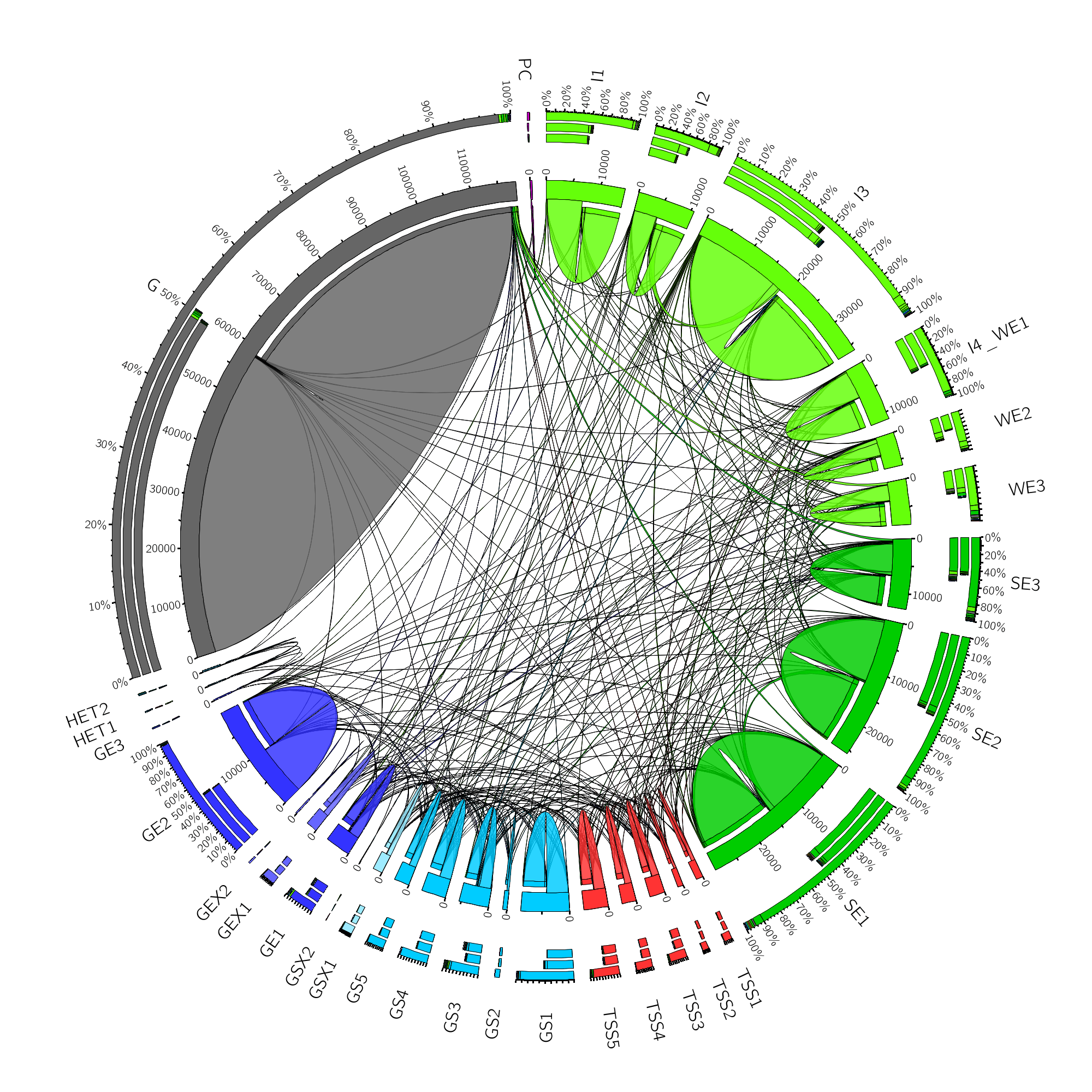

Supplement: Supplementary Data 4 — Effects of positive and negative perturbations of single chromatin factors on chromatin state identity. [file ncomms10528-s5.zip › Supplementary Data 4/NegativePerturbation/dmTopoII.png]

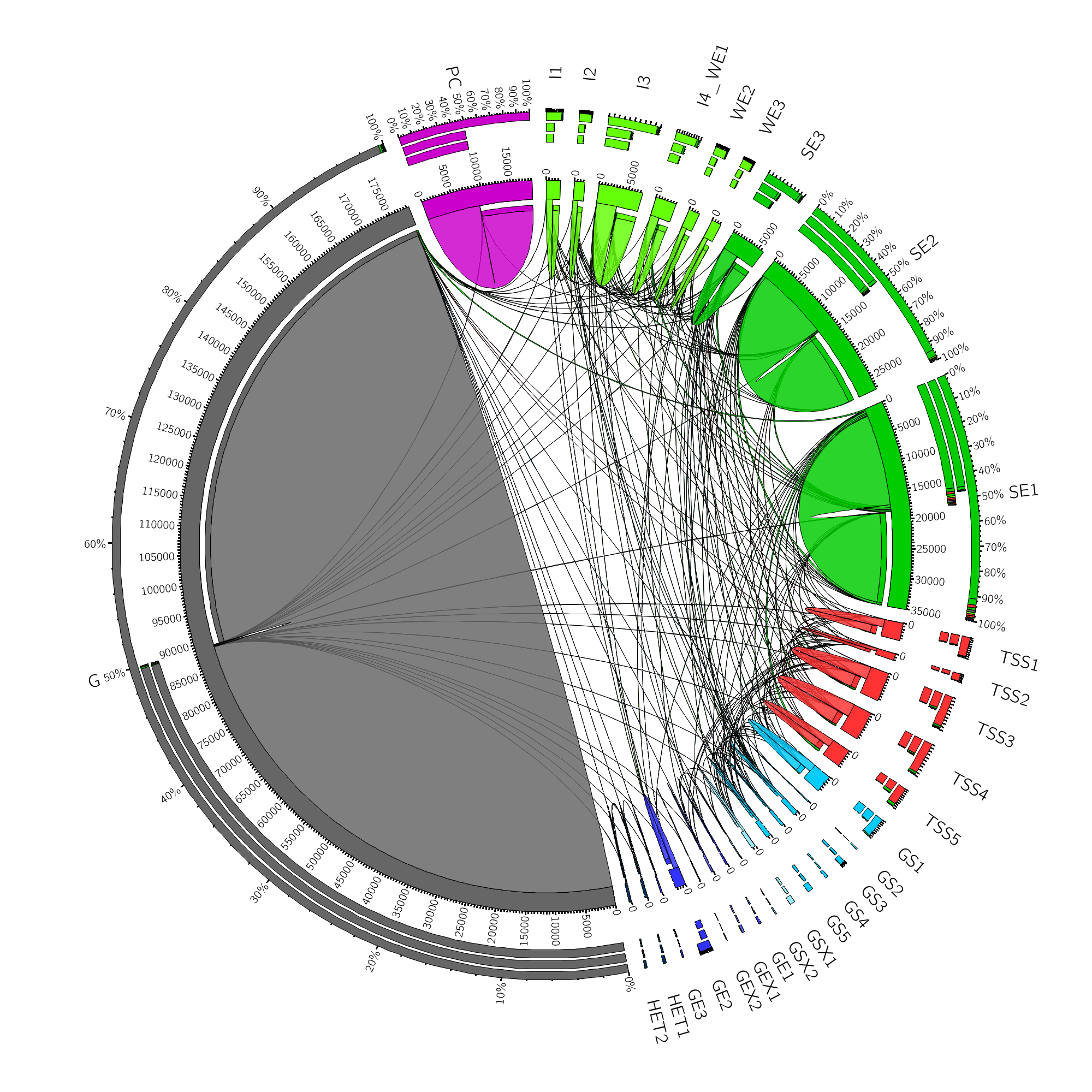

Supplement: Supplementary Data 4 — Effects of positive and negative perturbations of single chromatin factors on chromatin state identity. [file ncomms10528-s5.zip › Supplementary Data 4/NegativePerturbation/dRING.png]

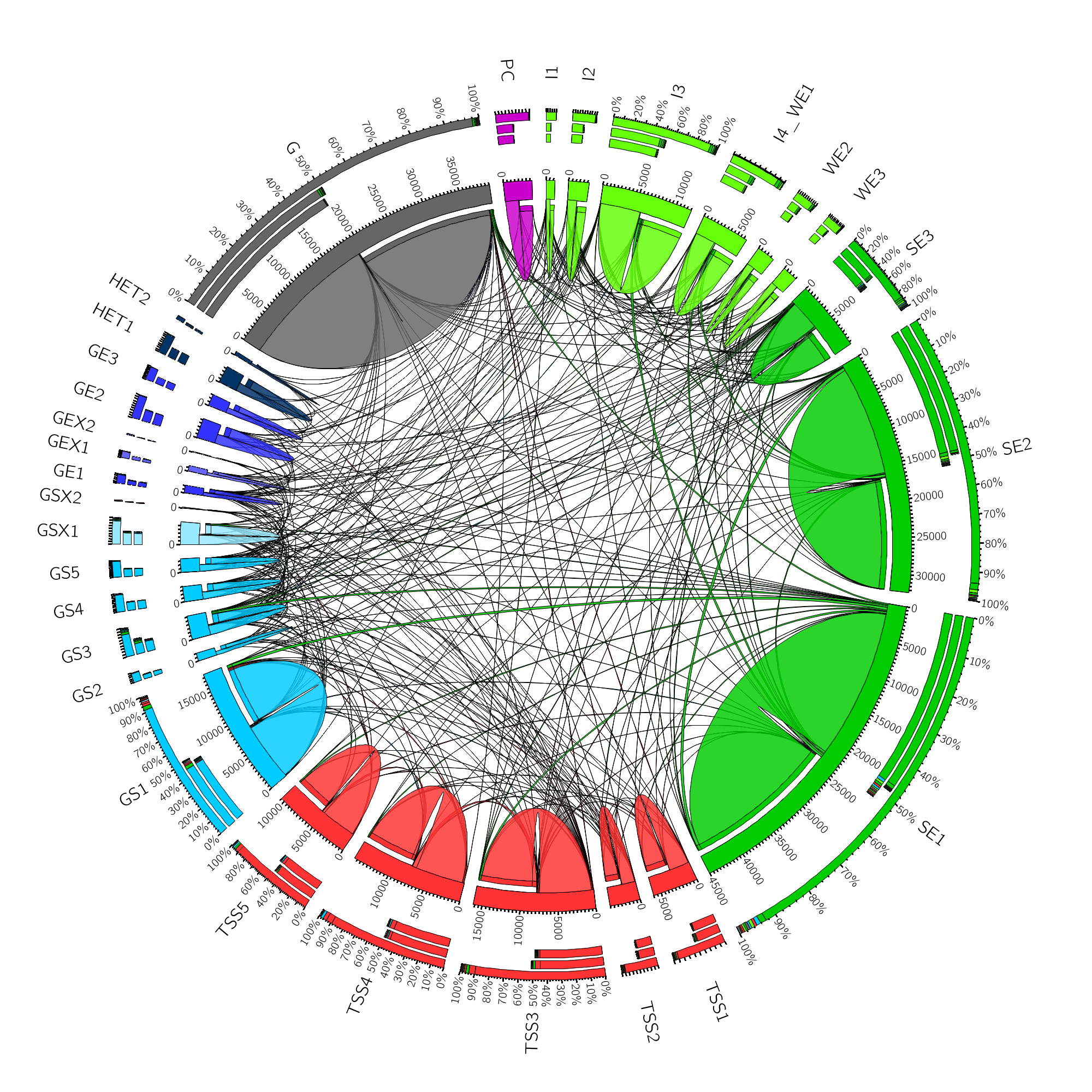

Supplement: Supplementary Data 4 — Effects of positive and negative perturbations of single chromatin factors on chromatin state identity. [file ncomms10528-s5.zip › Supplementary Data 4/NegativePerturbation/dSFMBT.png]

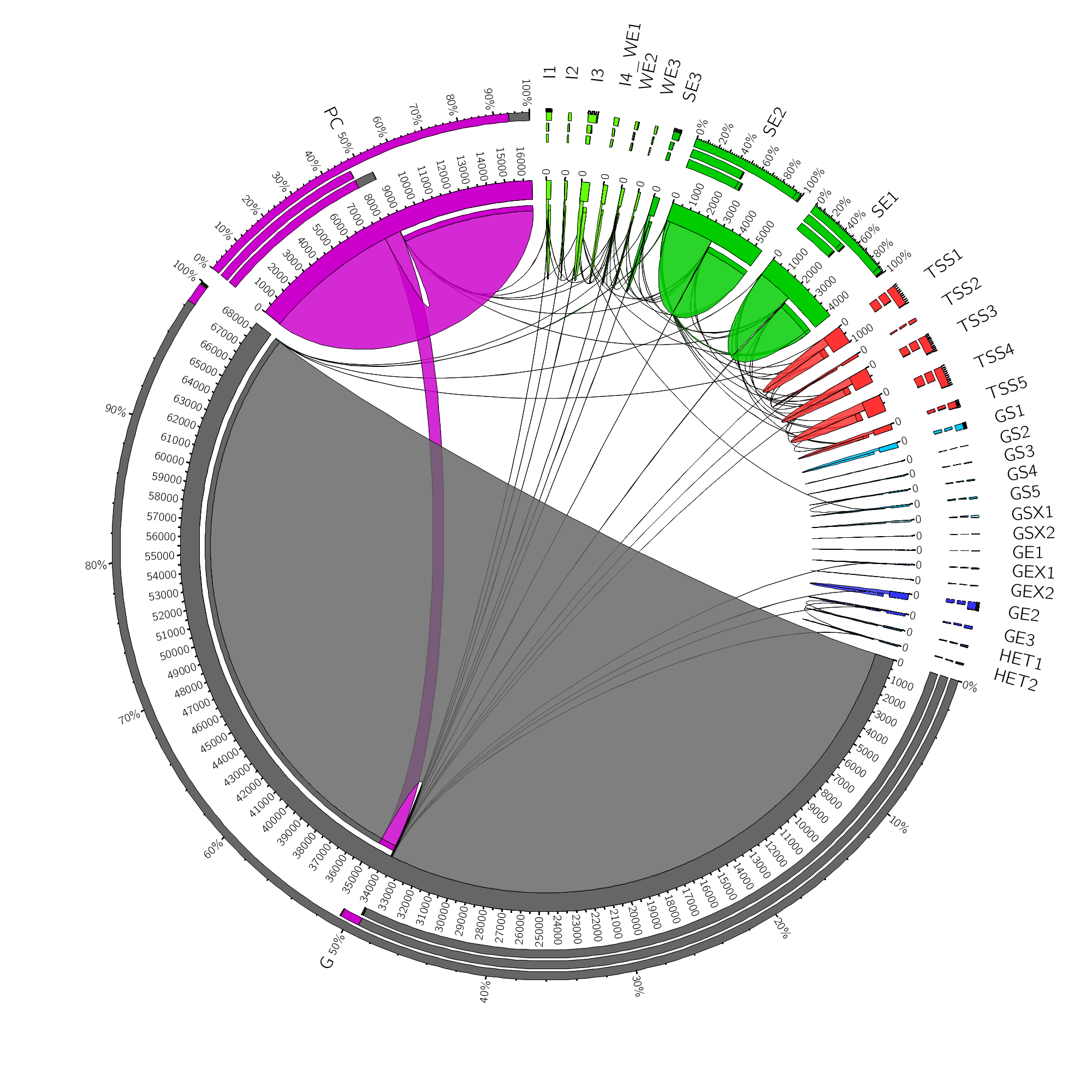

Supplement: Supplementary Data 4 — Effects of positive and negative perturbations of single chromatin factors on chromatin state identity. [file ncomms10528-s5.zip › Supplementary Data 4/NegativePerturbation/Ez.png]

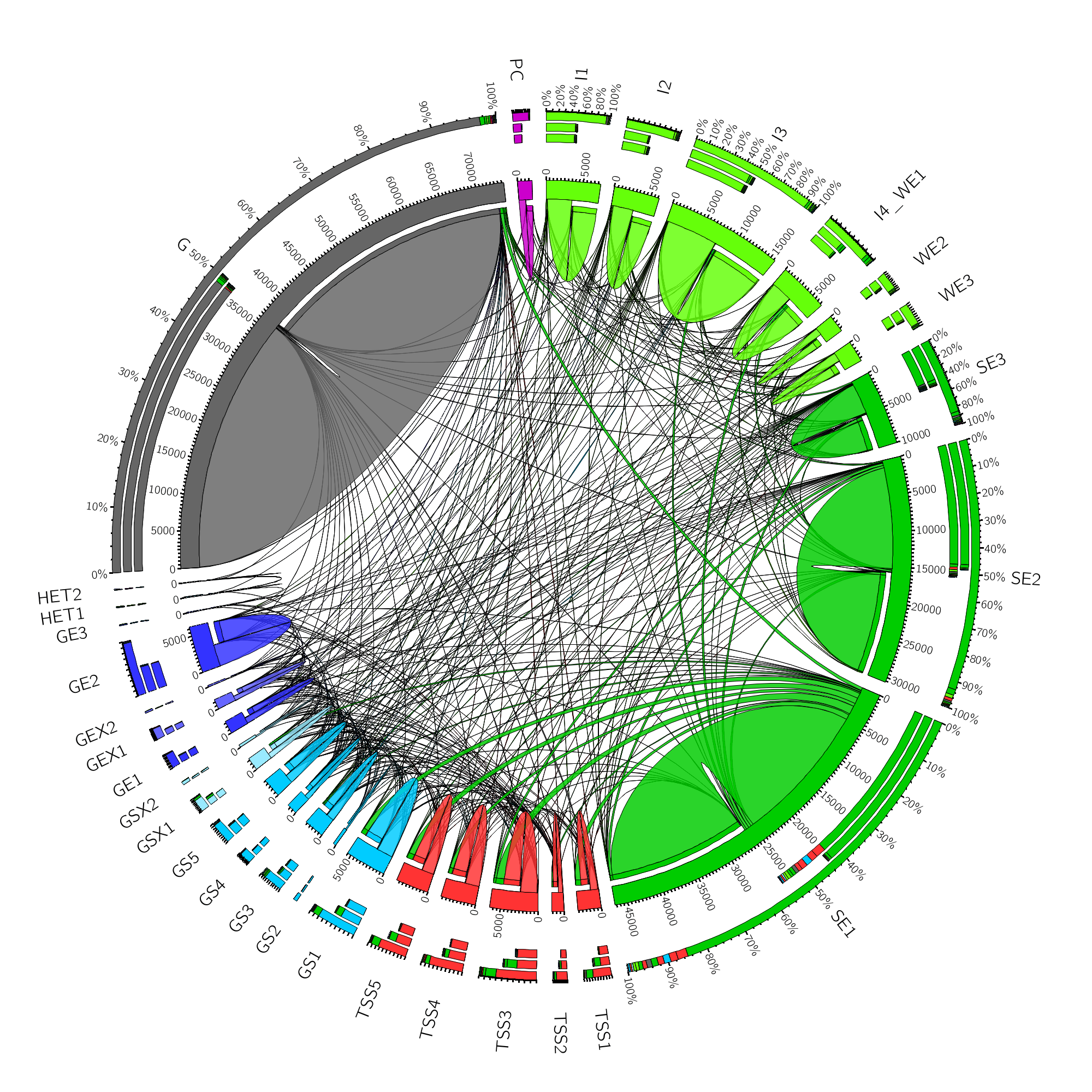

Supplement: Supplementary Data 4 — Effects of positive and negative perturbations of single chromatin factors on chromatin state identity. [file ncomms10528-s5.zip › Supplementary Data 4/NegativePerturbation/GAF.png]

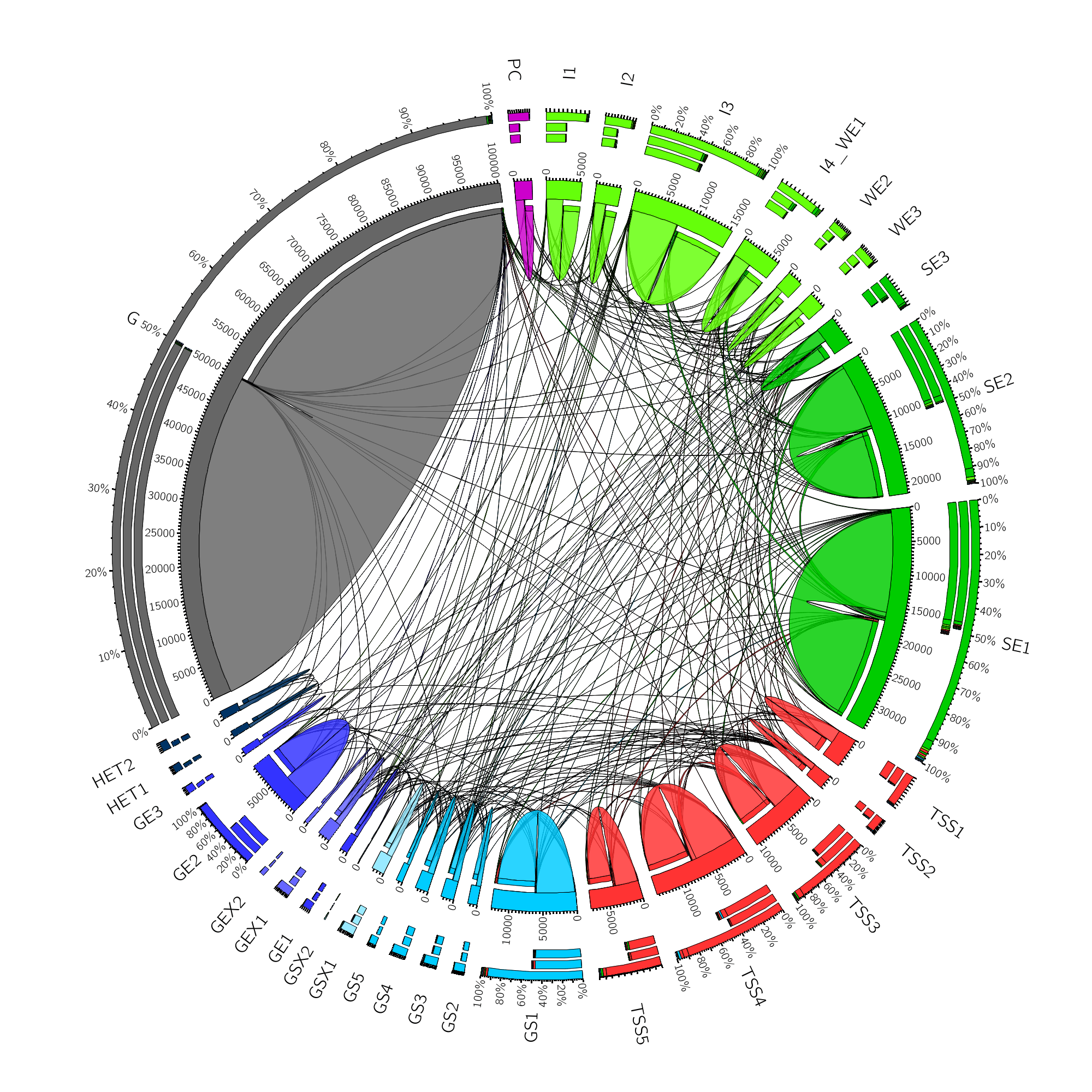

Supplement: Supplementary Data 4 — Effects of positive and negative perturbations of single chromatin factors on chromatin state identity. [file ncomms10528-s5.zip › Supplementary Data 4/NegativePerturbation/H1.png]

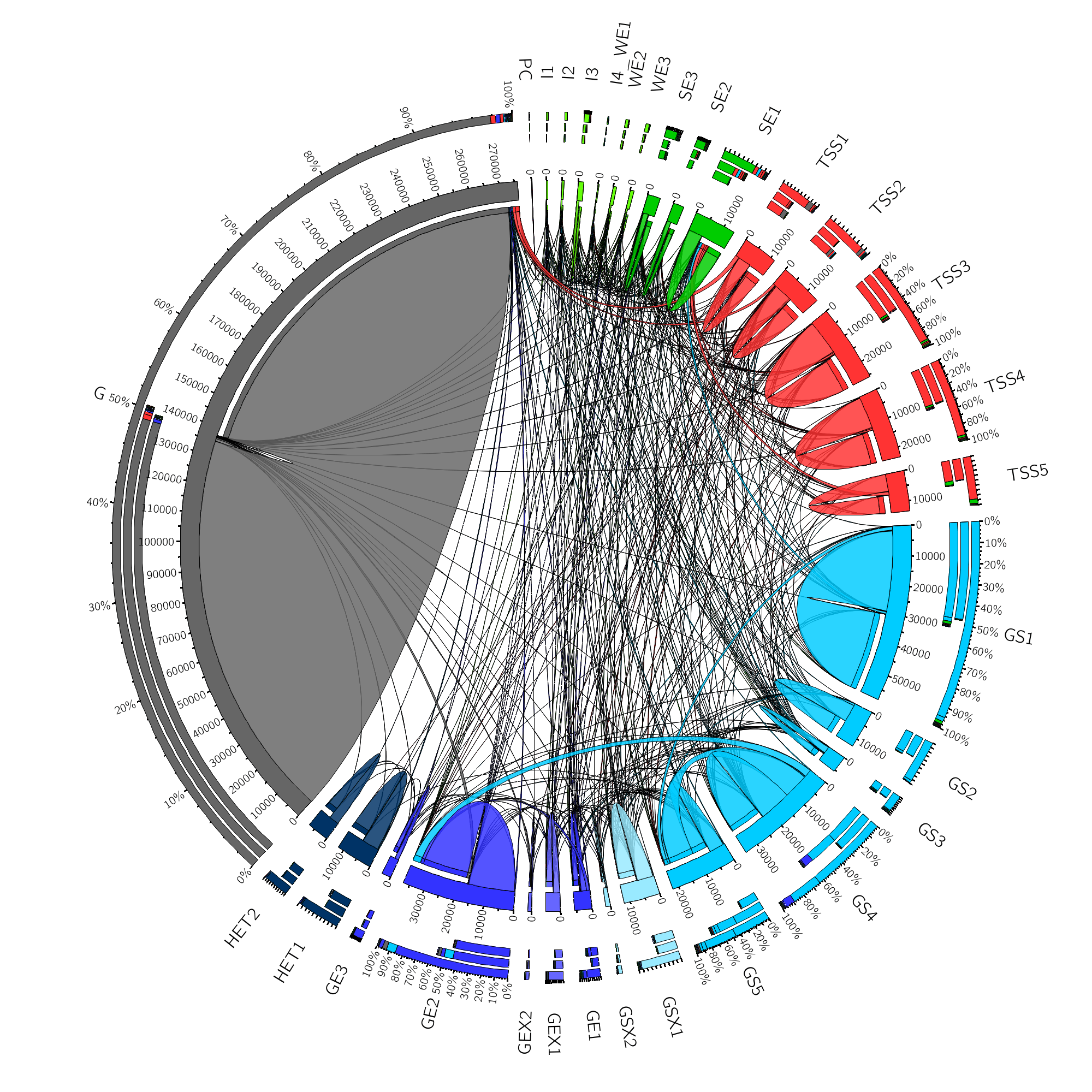

Supplement: Supplementary Data 4 — Effects of positive and negative perturbations of single chromatin factors on chromatin state identity. [file ncomms10528-s5.zip › Supplementary Data 4/NegativePerturbation/H2AV.png]

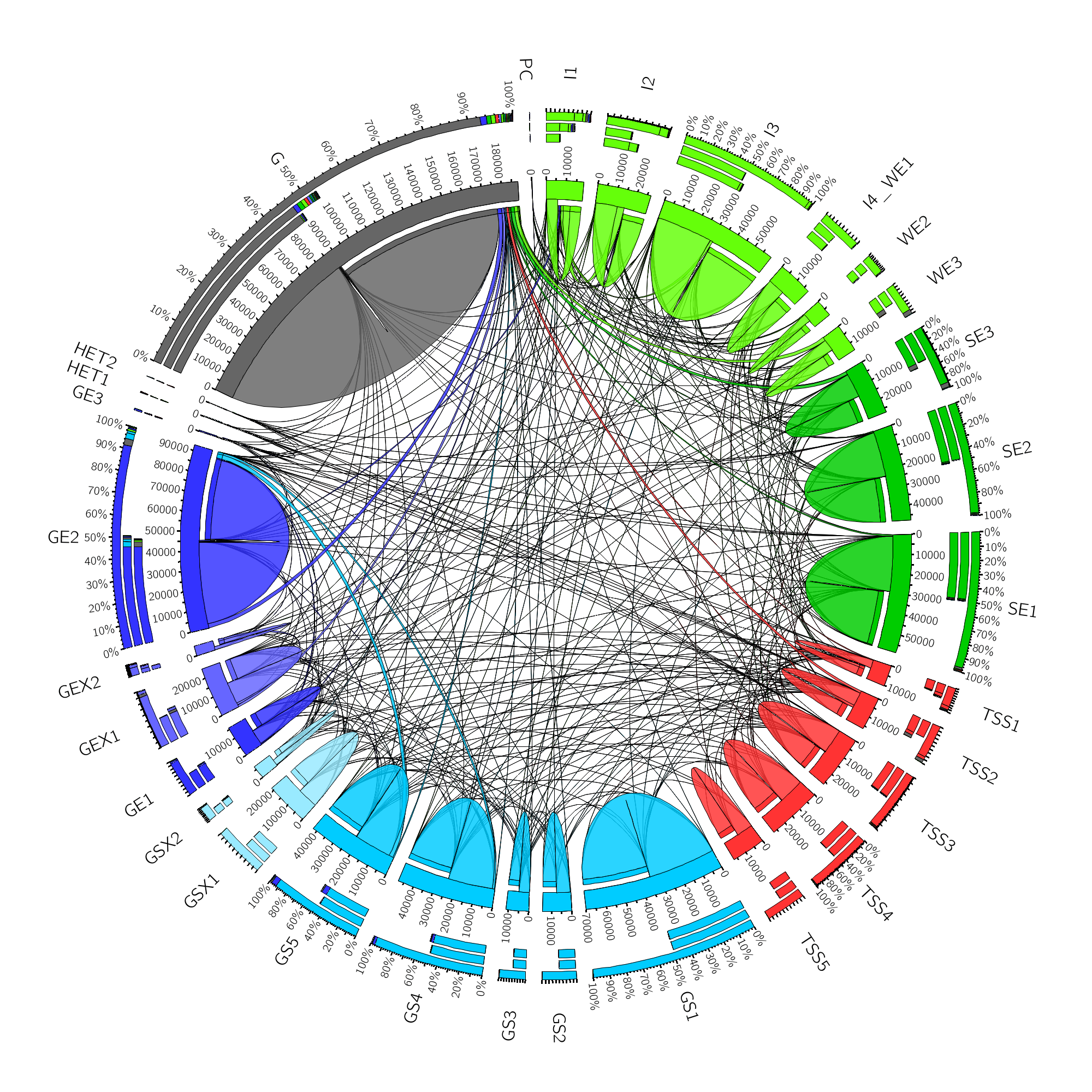

Supplement: Supplementary Data 4 — Effects of positive and negative perturbations of single chromatin factors on chromatin state identity. [file ncomms10528-s5.zip › Supplementary Data 4/NegativePerturbation/H2BK5ac.png]

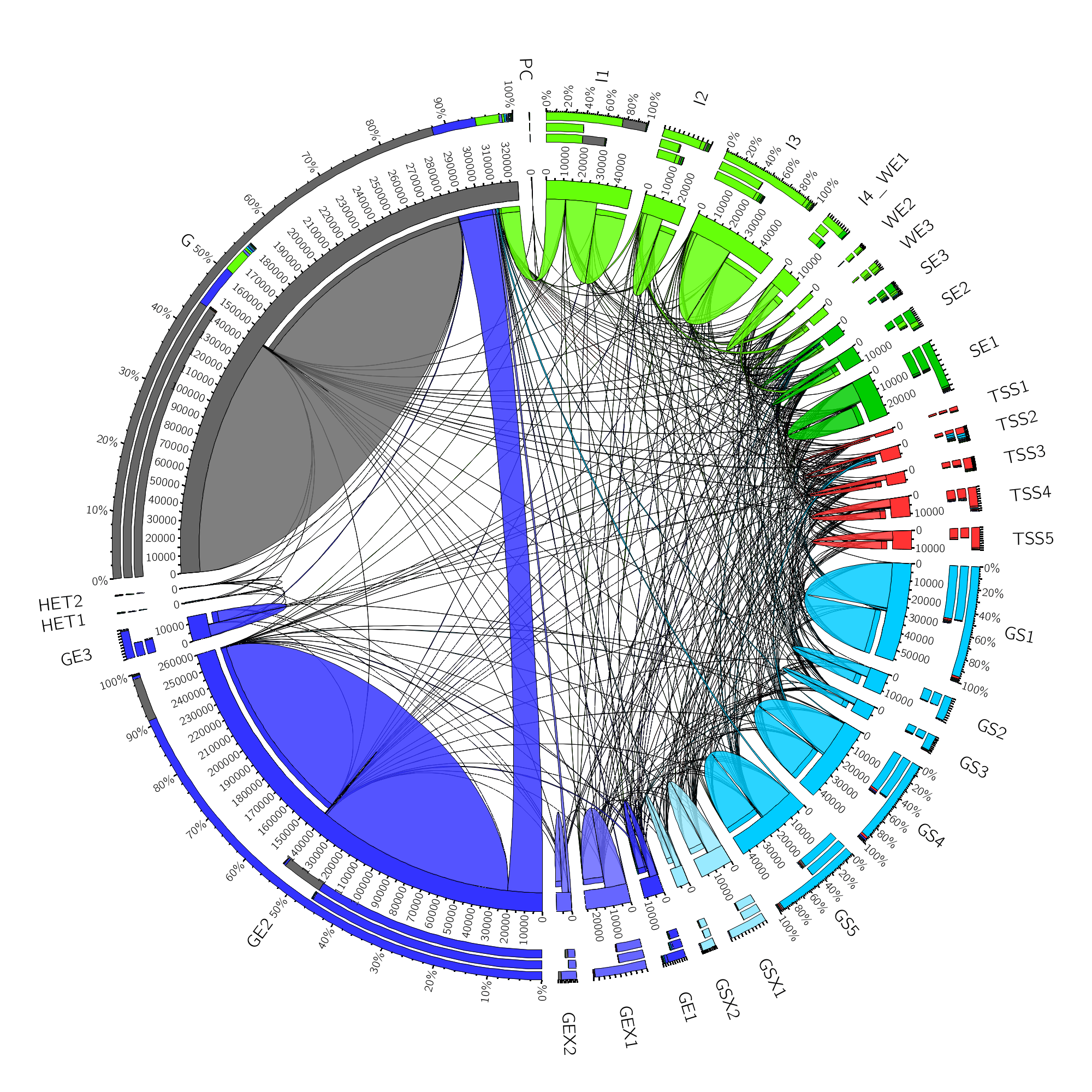

Supplement: Supplementary Data 4 — Effects of positive and negative perturbations of single chromatin factors on chromatin state identity. [file ncomms10528-s5.zip › Supplementary Data 4/NegativePerturbation/H2Bubi.png]

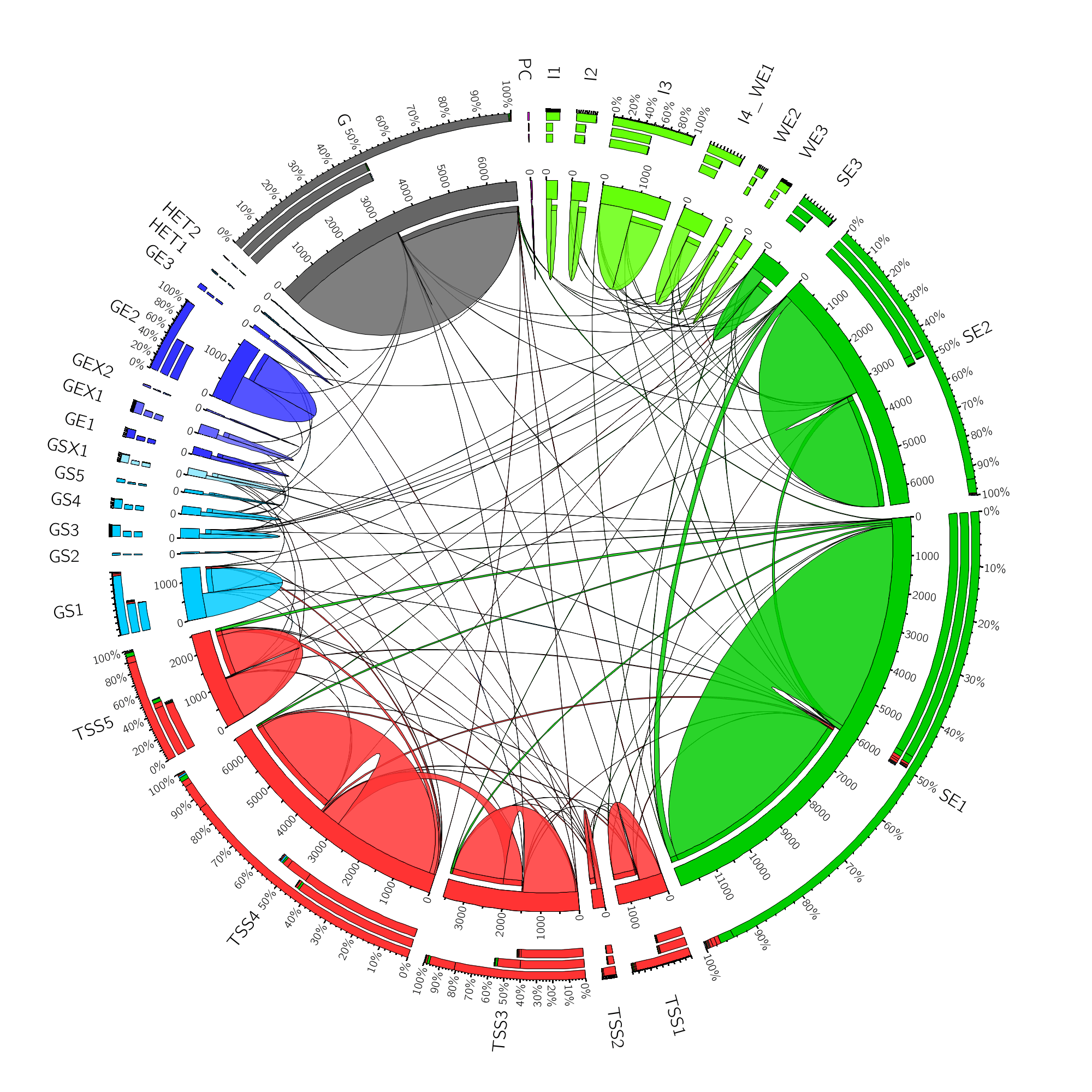

Supplement: Supplementary Data 4 — Effects of positive and negative perturbations of single chromatin factors on chromatin state identity. [file ncomms10528-s5.zip › Supplementary Data 4/NegativePerturbation/H3.png]

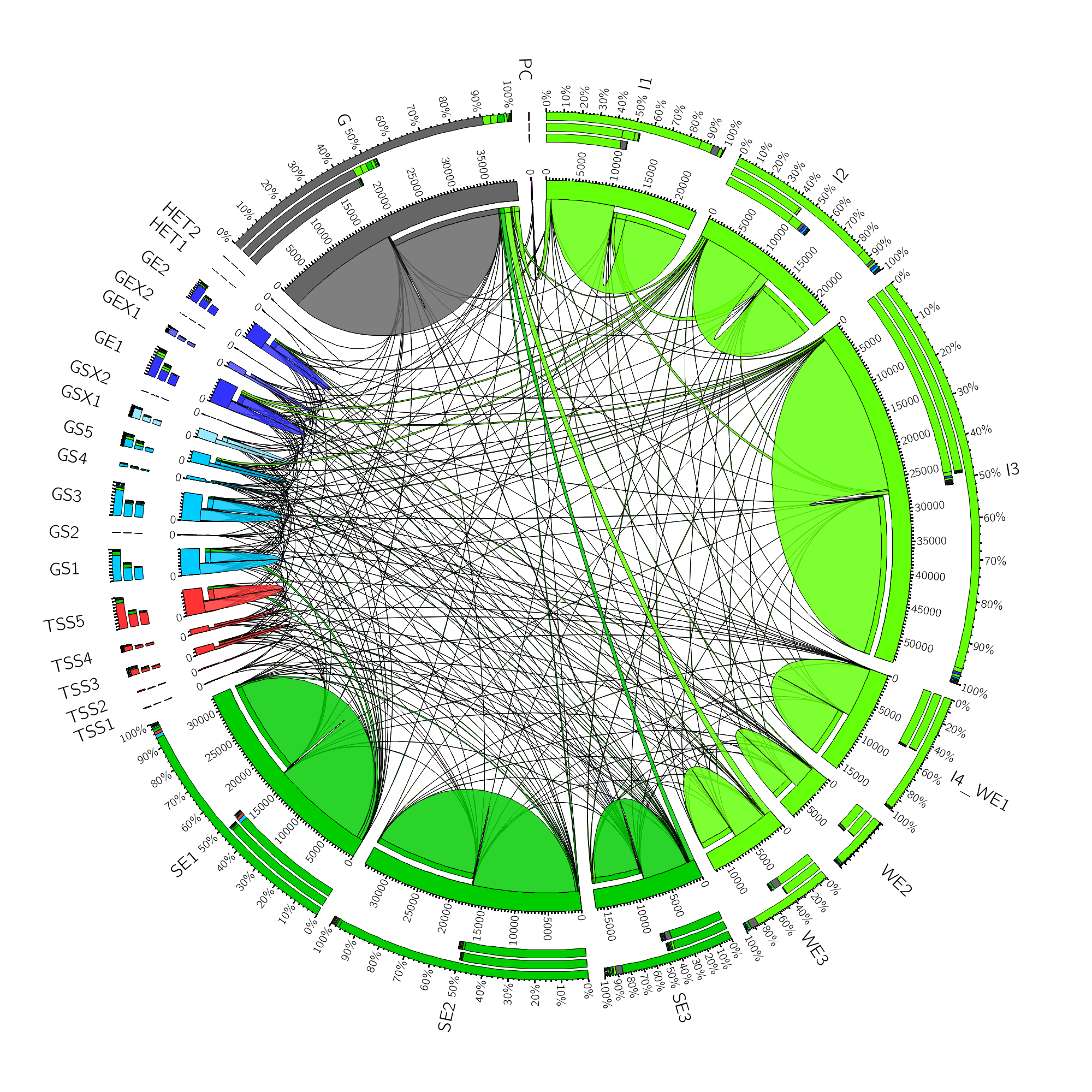

Supplement: Supplementary Data 4 — Effects of positive and negative perturbations of single chromatin factors on chromatin state identity. [file ncomms10528-s5.zip › Supplementary Data 4/NegativePerturbation/H3K18ac.png]

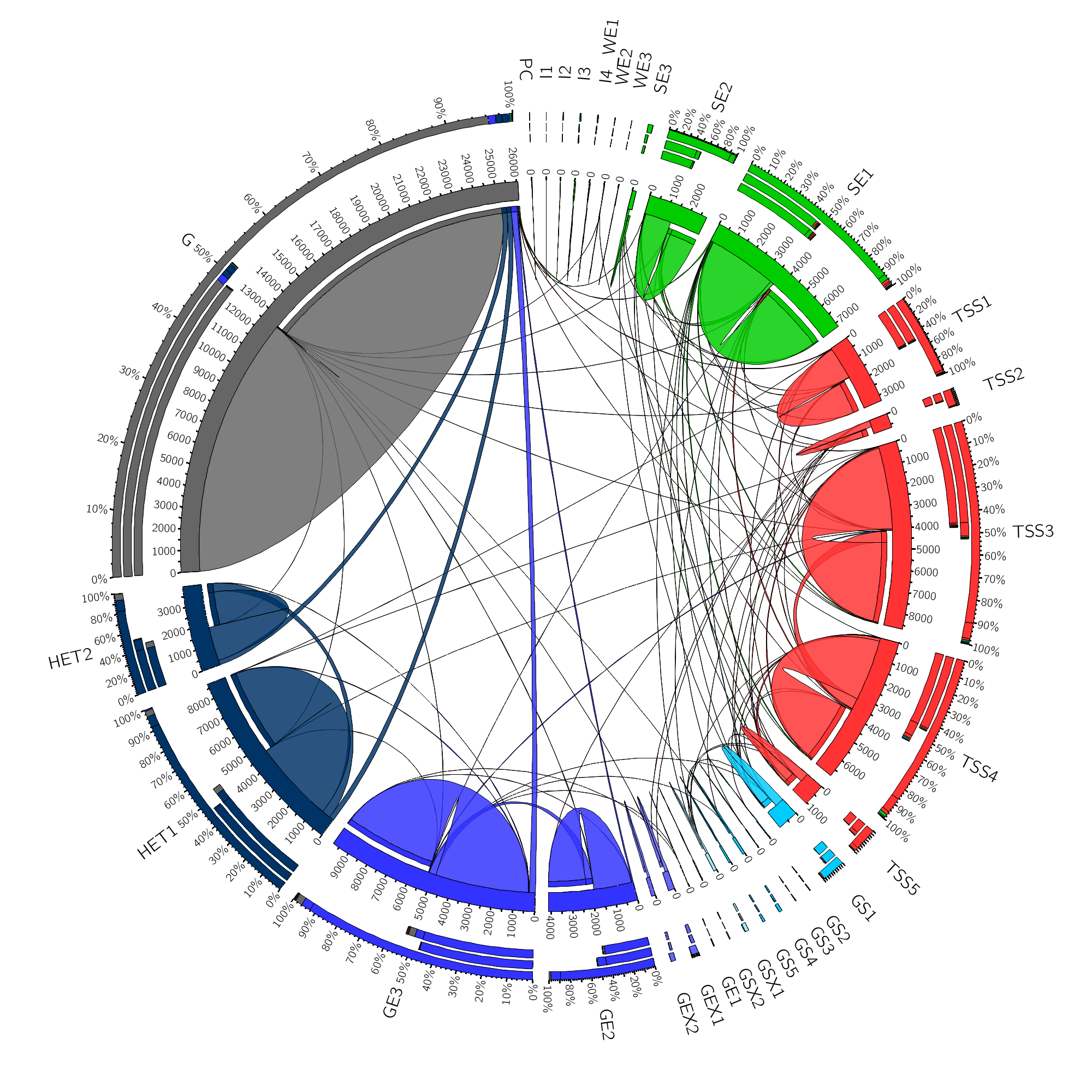

Supplement: Supplementary Data 4 — Effects of positive and negative perturbations of single chromatin factors on chromatin state identity. [file ncomms10528-s5.zip › Supplementary Data 4/NegativePerturbation/H3K23ac.png]

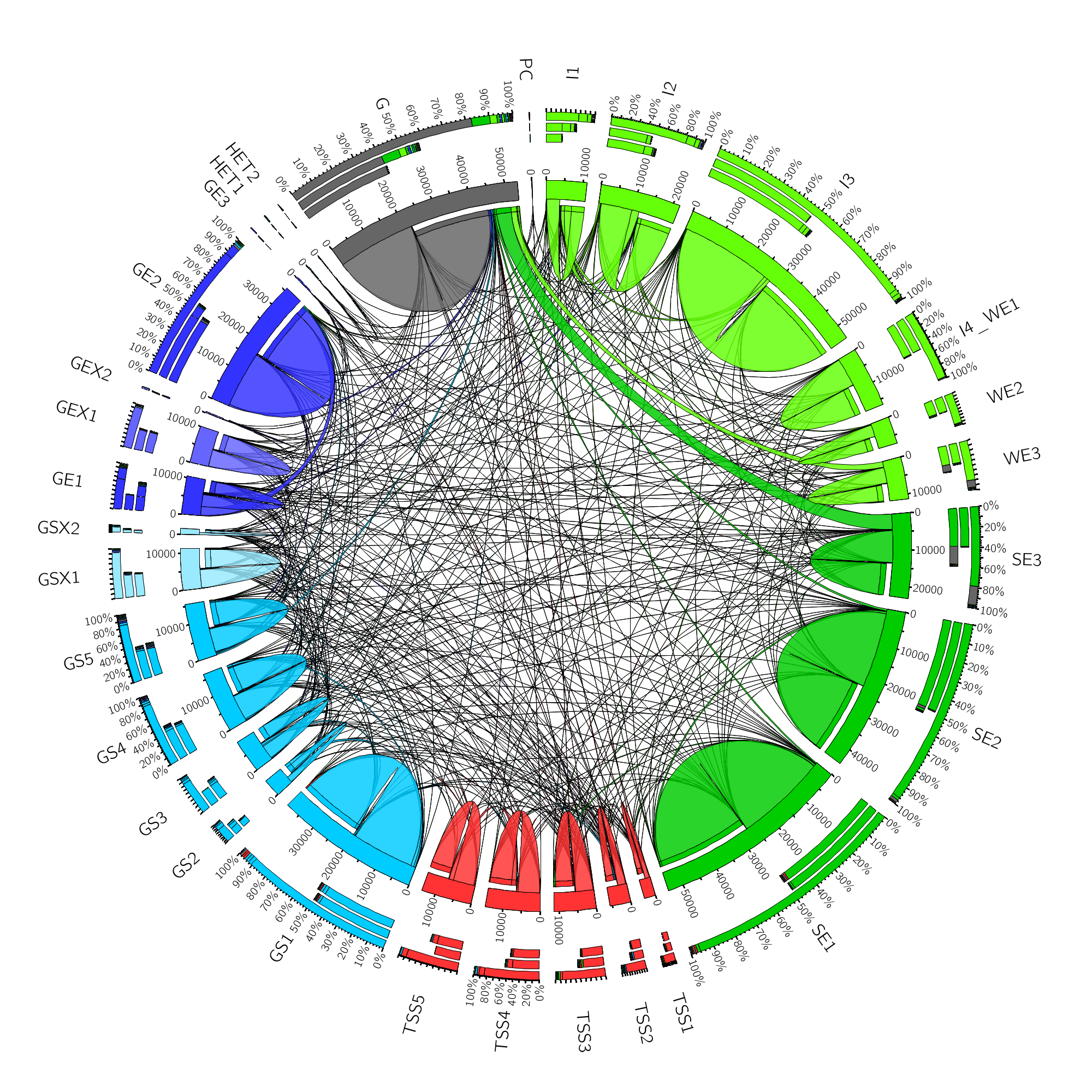

Supplement: Supplementary Data 4 — Effects of positive and negative perturbations of single chromatin factors on chromatin state identity. [file ncomms10528-s5.zip › Supplementary Data 4/NegativePerturbation/H3K27ac.png]

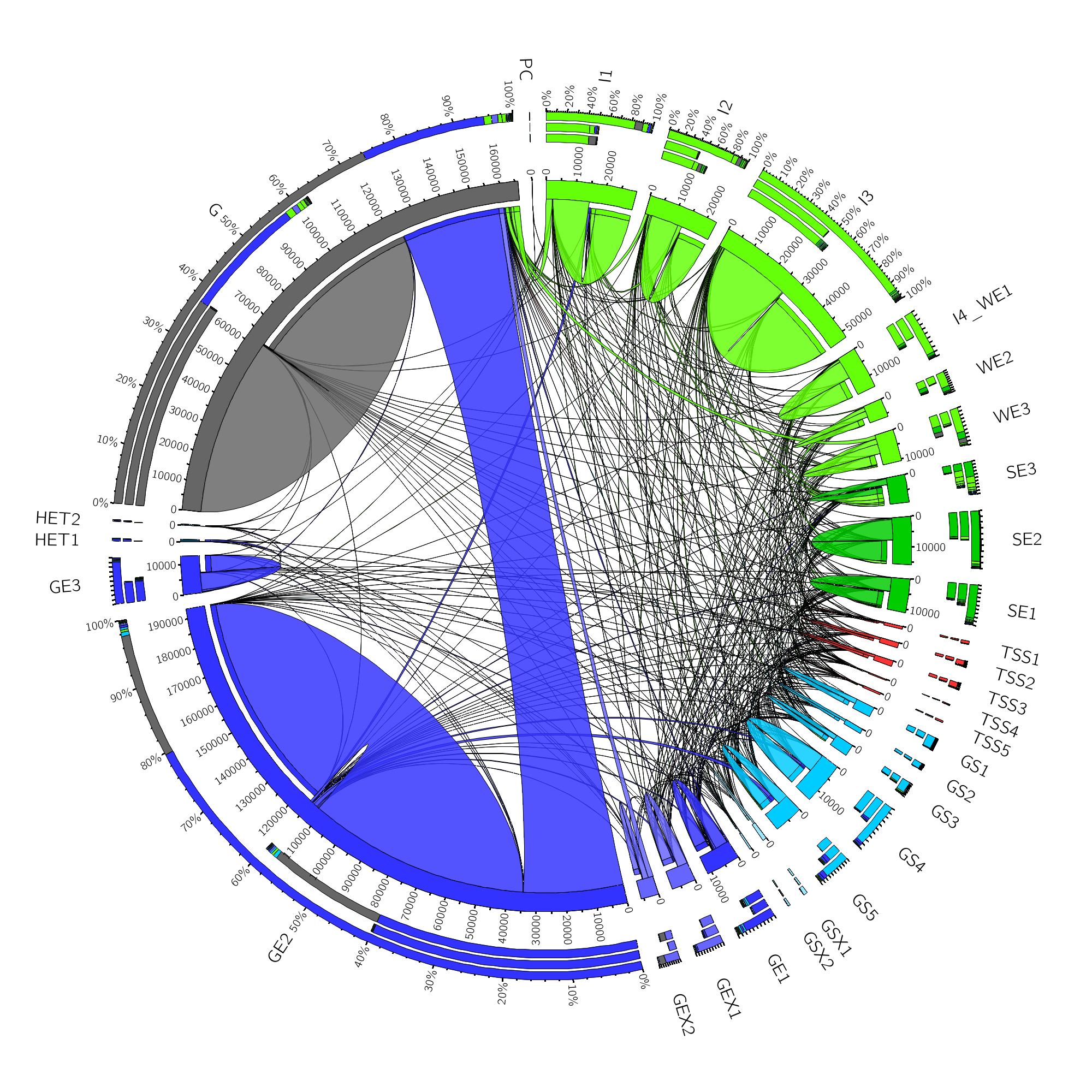

Supplement: Supplementary Data 4 — Effects of positive and negative perturbations of single chromatin factors on chromatin state identity. [file ncomms10528-s5.zip › Supplementary Data 4/NegativePerturbation/H3K27me1.png]

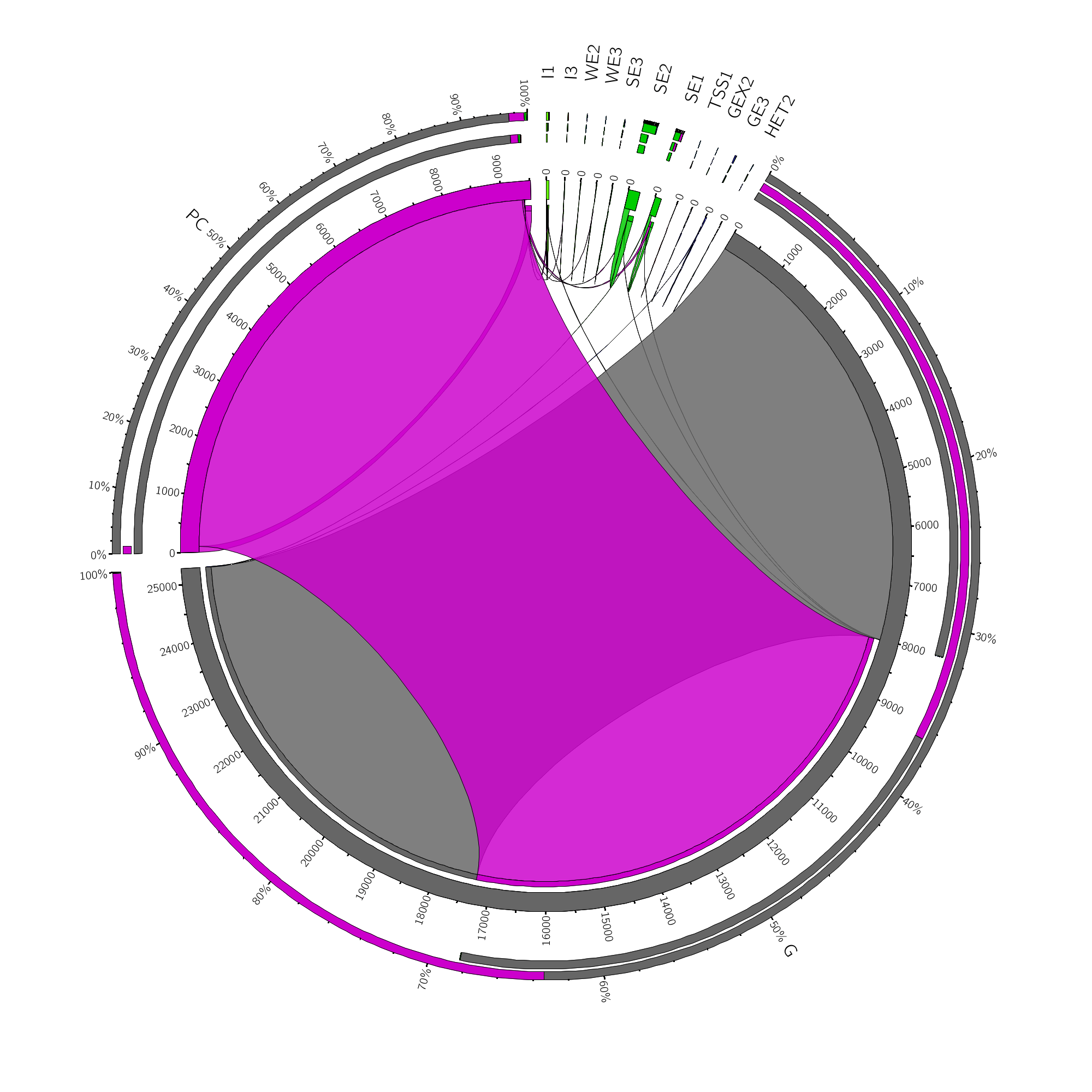

Supplement: Supplementary Data 4 — Effects of positive and negative perturbations of single chromatin factors on chromatin state identity. [file ncomms10528-s5.zip › Supplementary Data 4/NegativePerturbation/H3K27me3.png]

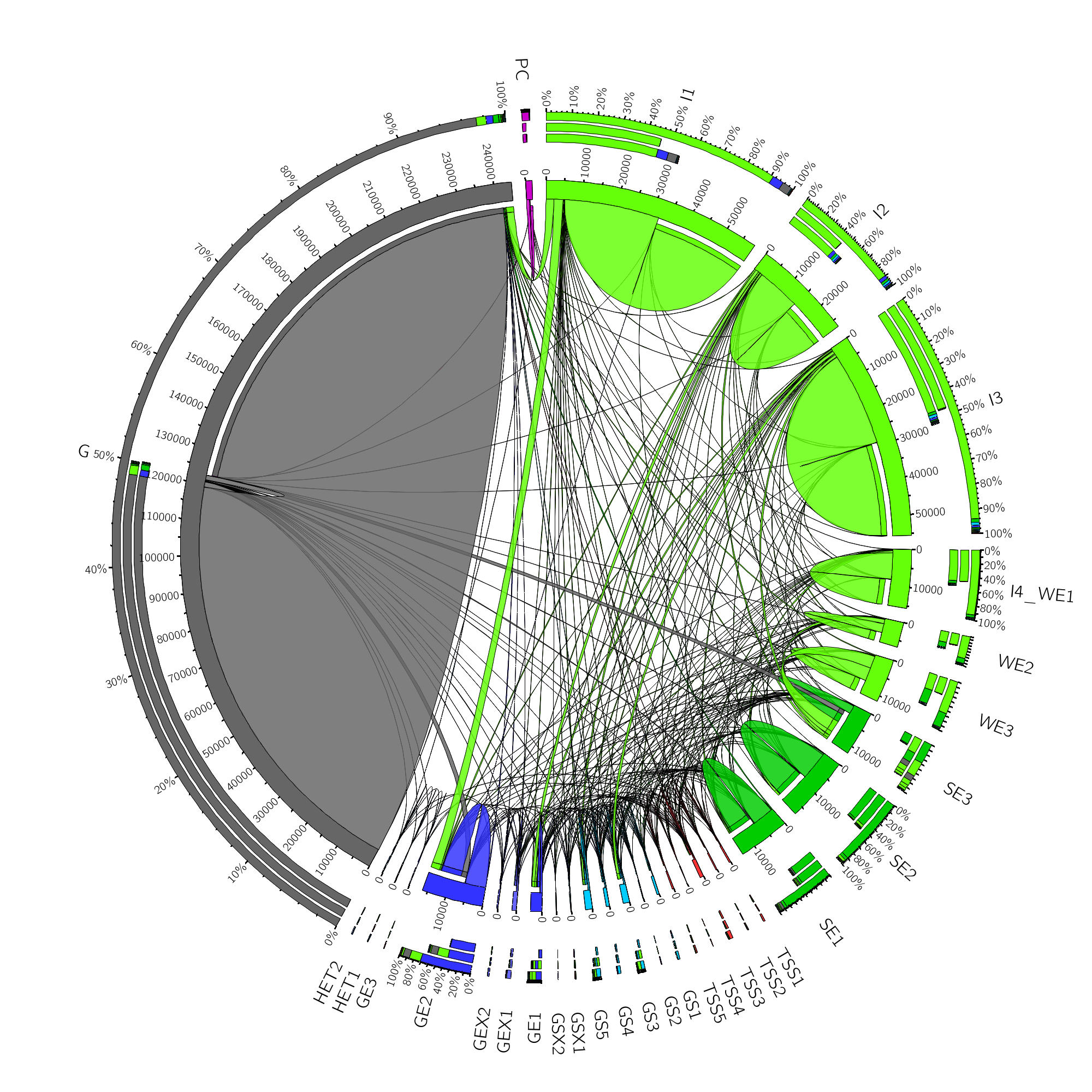

Supplement: Supplementary Data 4 — Effects of positive and negative perturbations of single chromatin factors on chromatin state identity. [file ncomms10528-s5.zip › Supplementary Data 4/NegativePerturbation/H3K36me1.png]

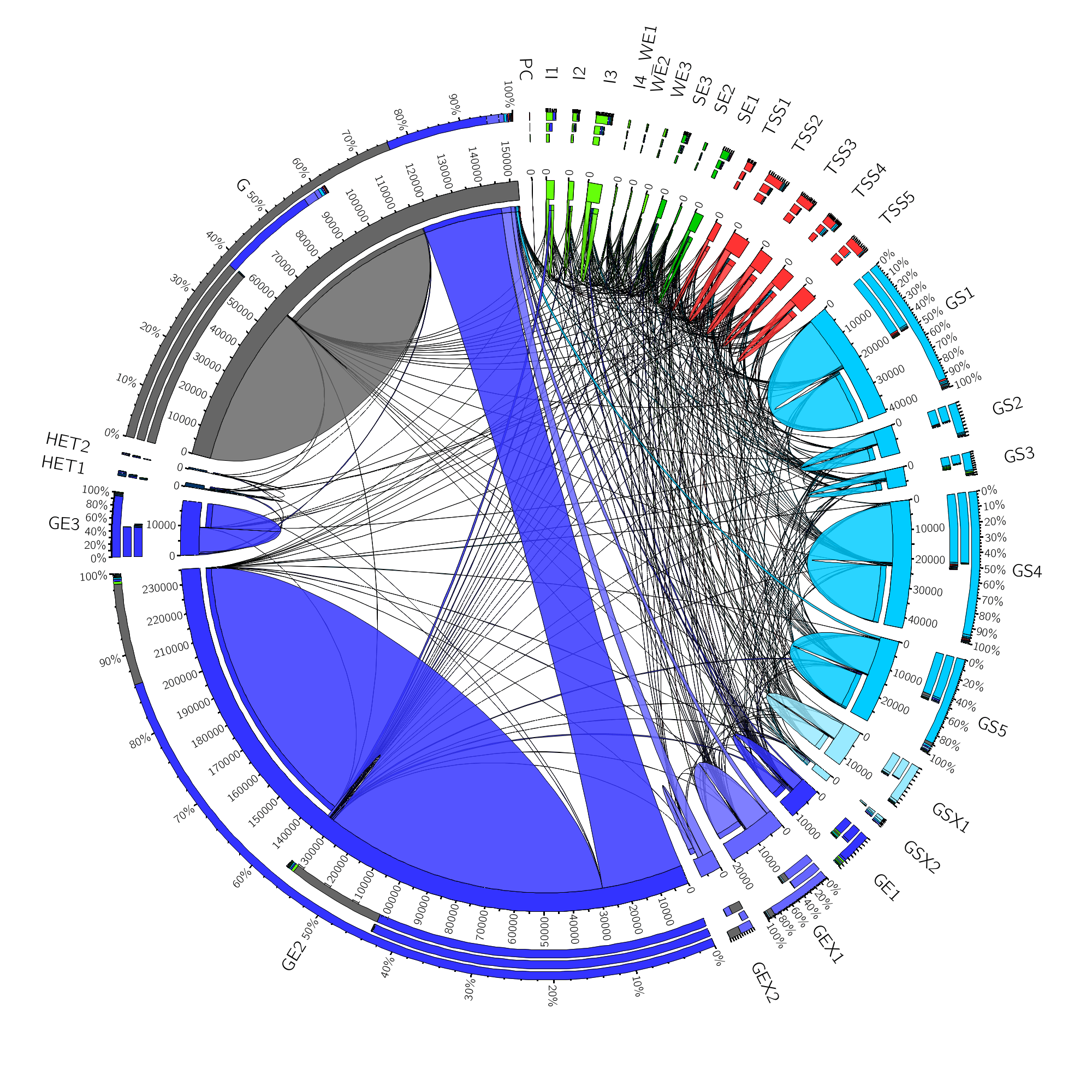

Supplement: Supplementary Data 4 — Effects of positive and negative perturbations of single chromatin factors on chromatin state identity. [file ncomms10528-s5.zip › Supplementary Data 4/NegativePerturbation/H3K36me3.png]

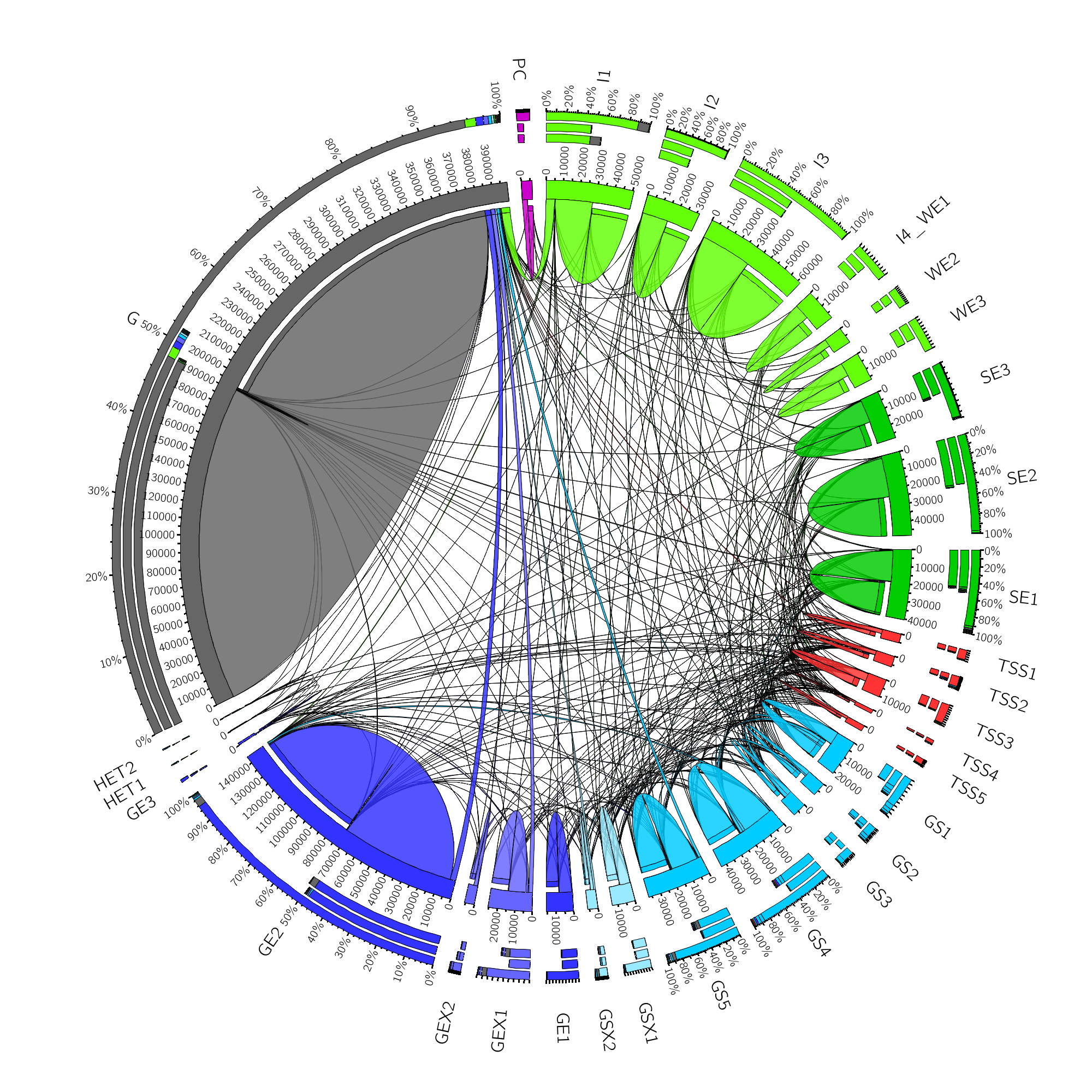

Supplement: Supplementary Data 4 — Effects of positive and negative perturbations of single chromatin factors on chromatin state identity. [file ncomms10528-s5.zip › Supplementary Data 4/NegativePerturbation/H3K4me1.png]

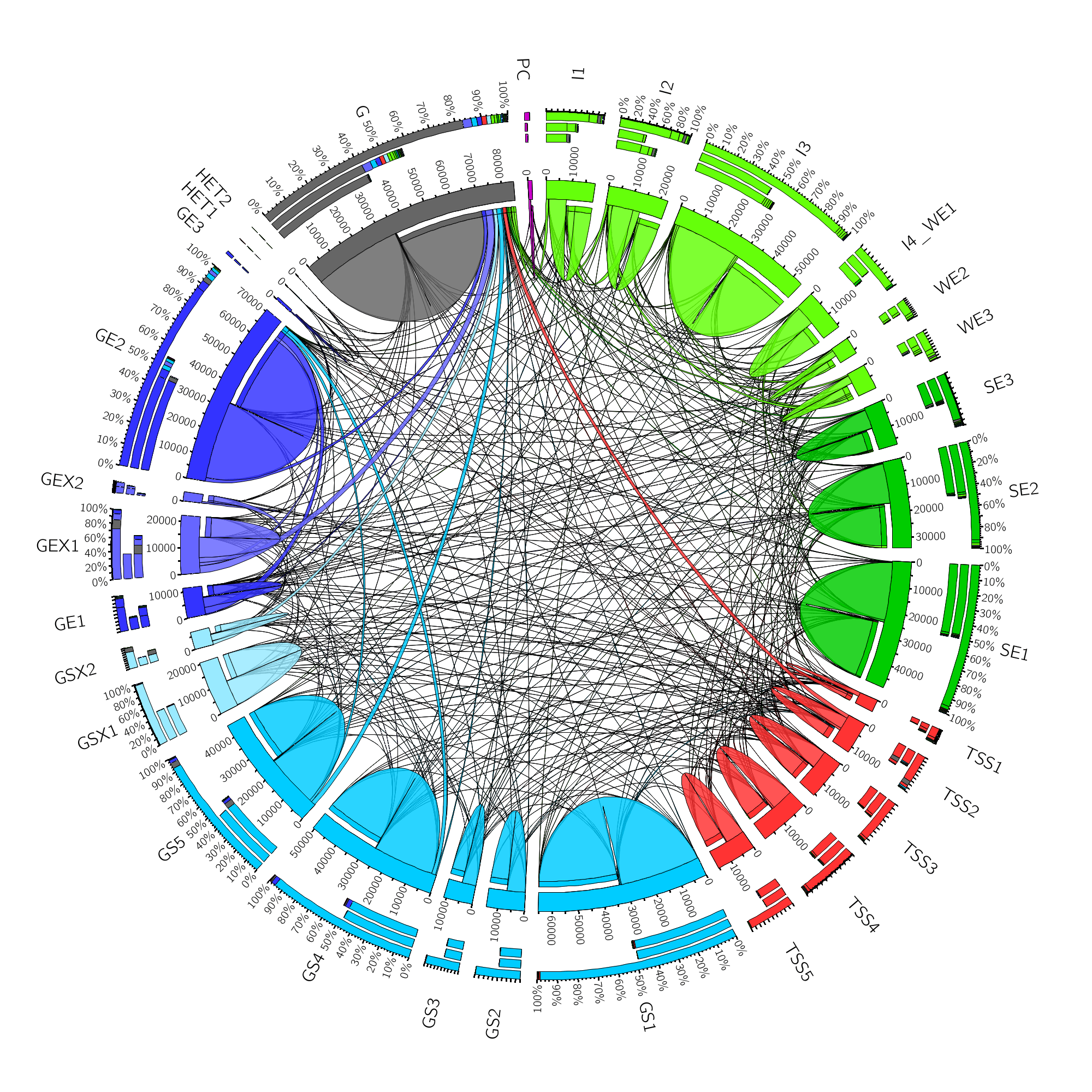

Supplement: Supplementary Data 4 — Effects of positive and negative perturbations of single chromatin factors on chromatin state identity. [file ncomms10528-s5.zip › Supplementary Data 4/NegativePerturbation/H3K4me2.png]

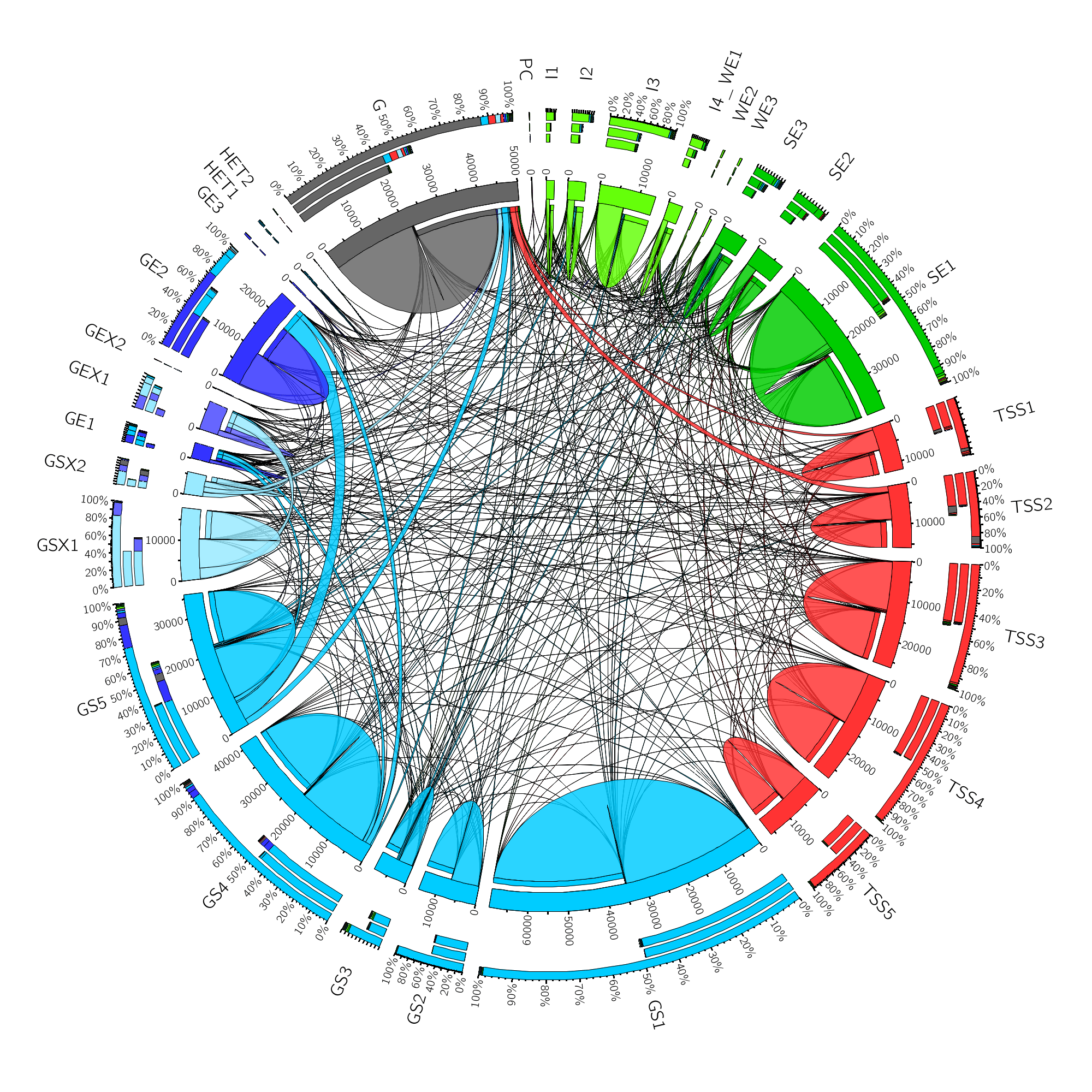

Supplement: Supplementary Data 4 — Effects of positive and negative perturbations of single chromatin factors on chromatin state identity. [file ncomms10528-s5.zip › Supplementary Data 4/NegativePerturbation/H3K4me3.png]

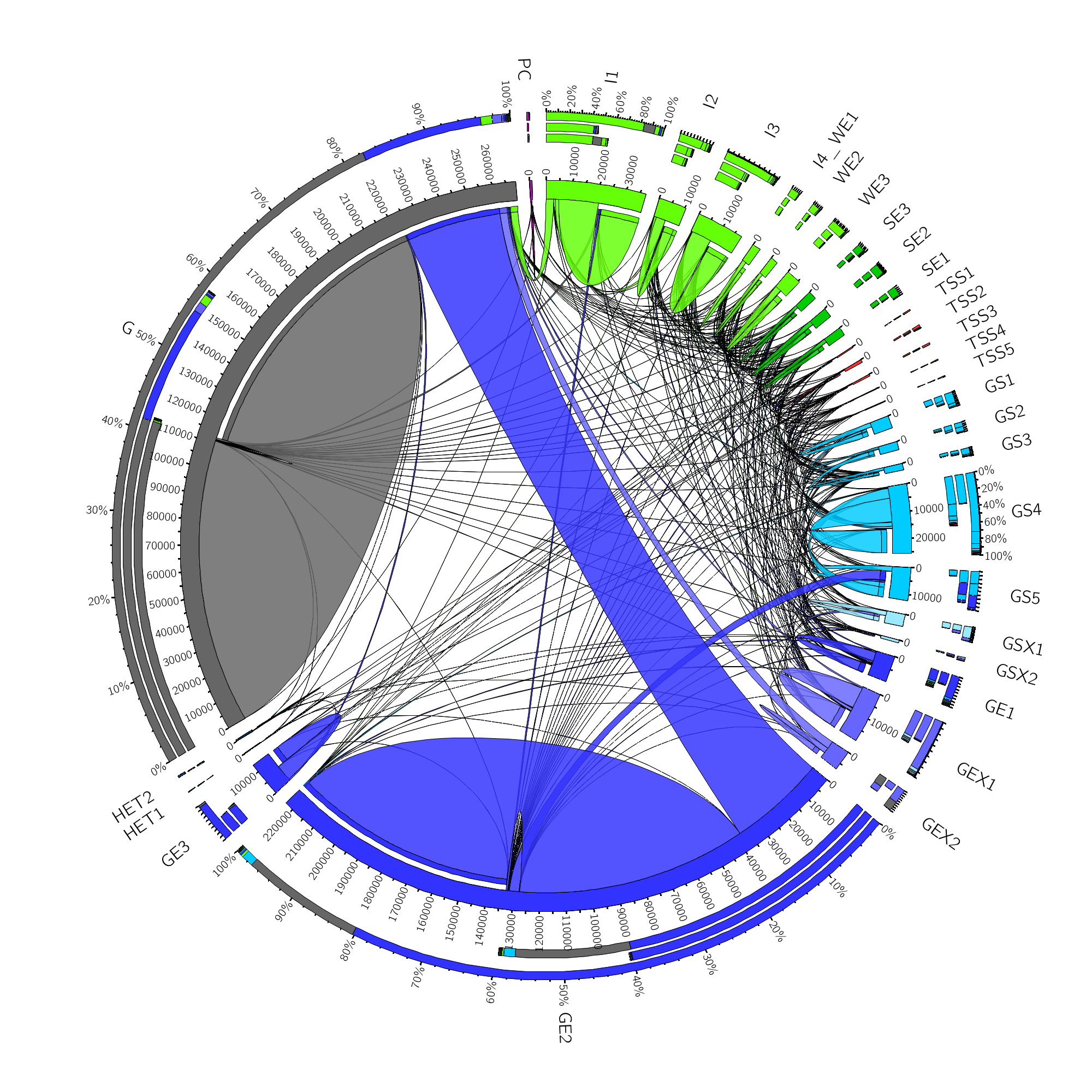

Supplement: Supplementary Data 4 — Effects of positive and negative perturbations of single chromatin factors on chromatin state identity. [file ncomms10528-s5.zip › Supplementary Data 4/NegativePerturbation/H3K79me1.png]

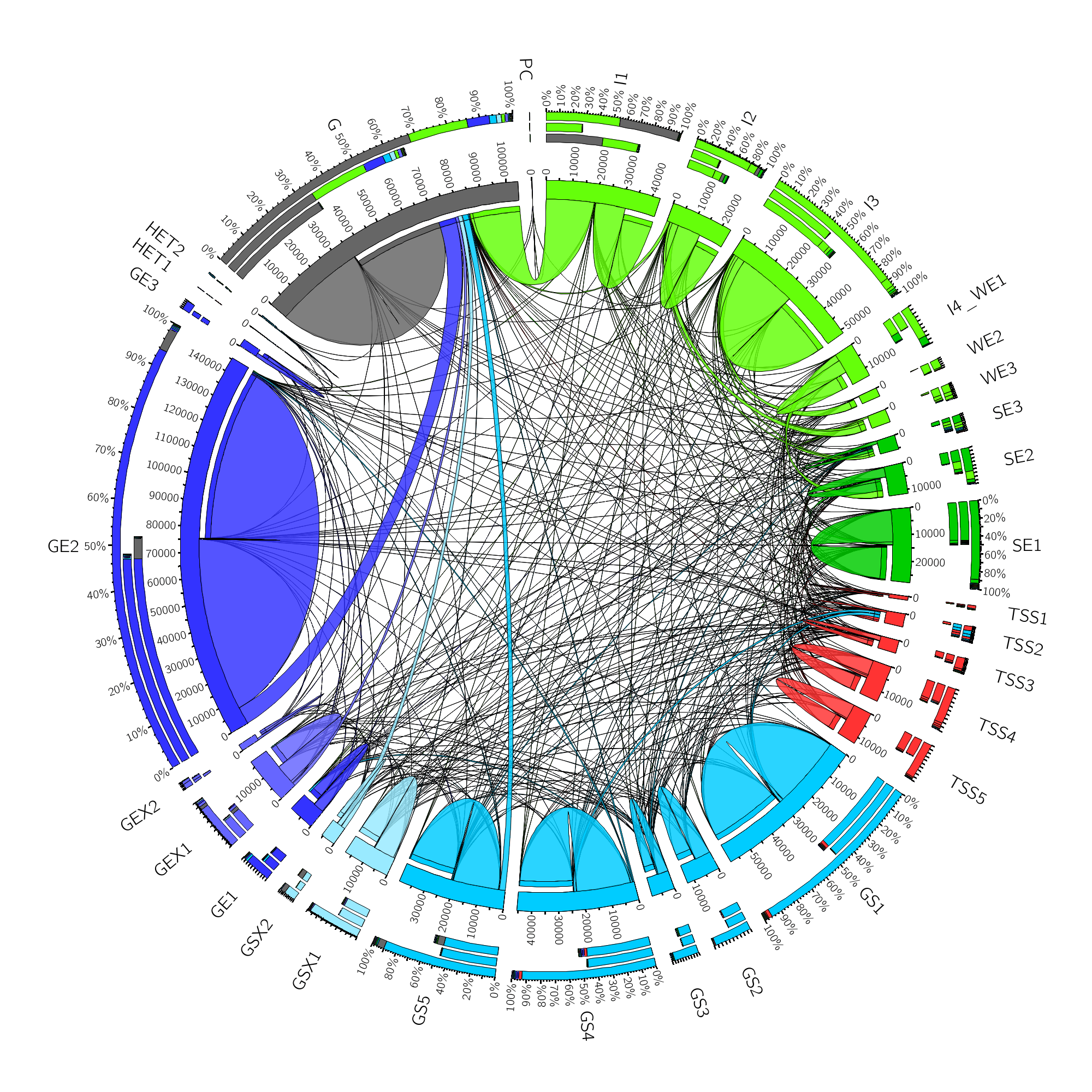

Supplement: Supplementary Data 4 — Effects of positive and negative perturbations of single chromatin factors on chromatin state identity. [file ncomms10528-s5.zip › Supplementary Data 4/NegativePerturbation/H3K79me2.png]

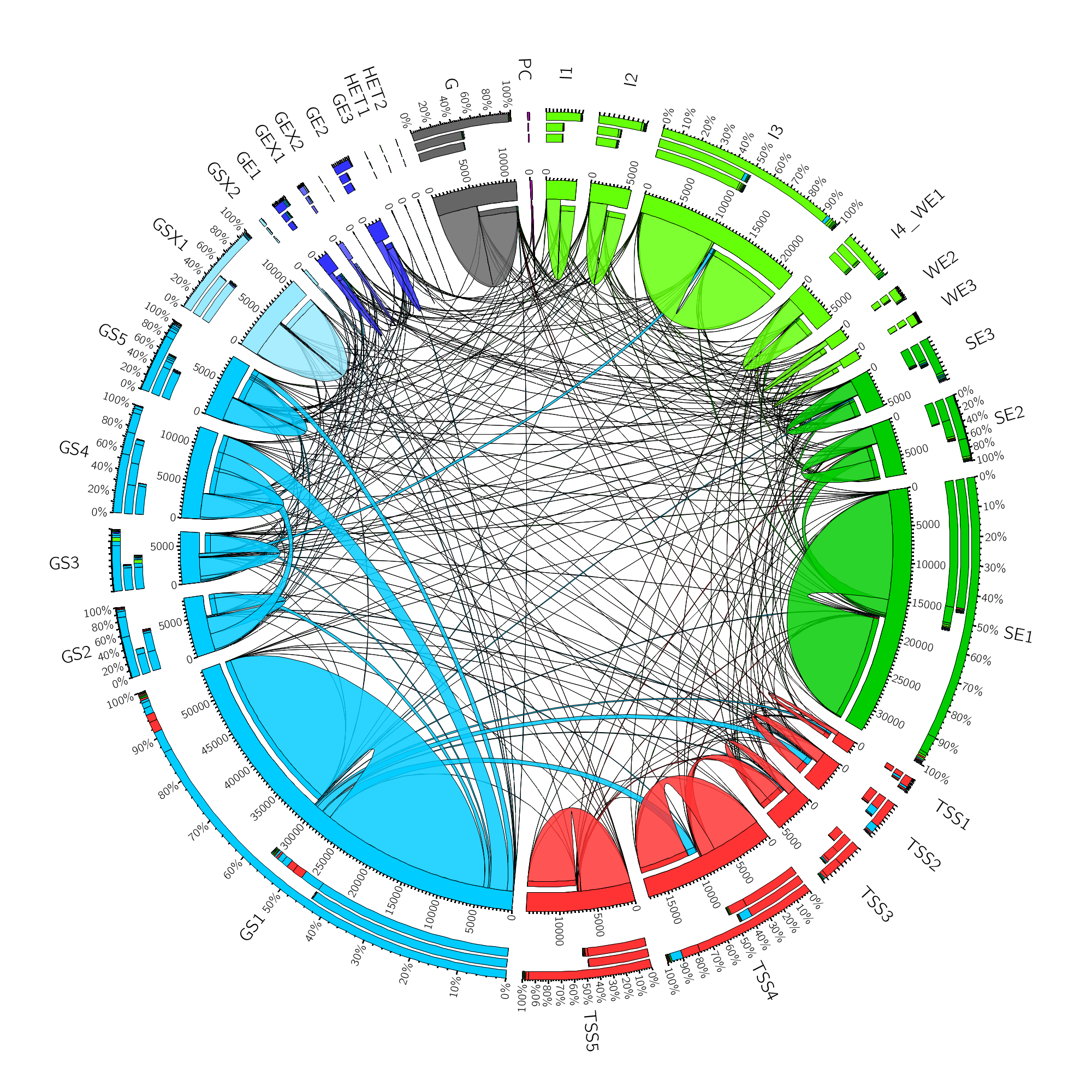

Supplement: Supplementary Data 4 — Effects of positive and negative perturbations of single chromatin factors on chromatin state identity. [file ncomms10528-s5.zip › Supplementary Data 4/NegativePerturbation/H3K9ac.png]

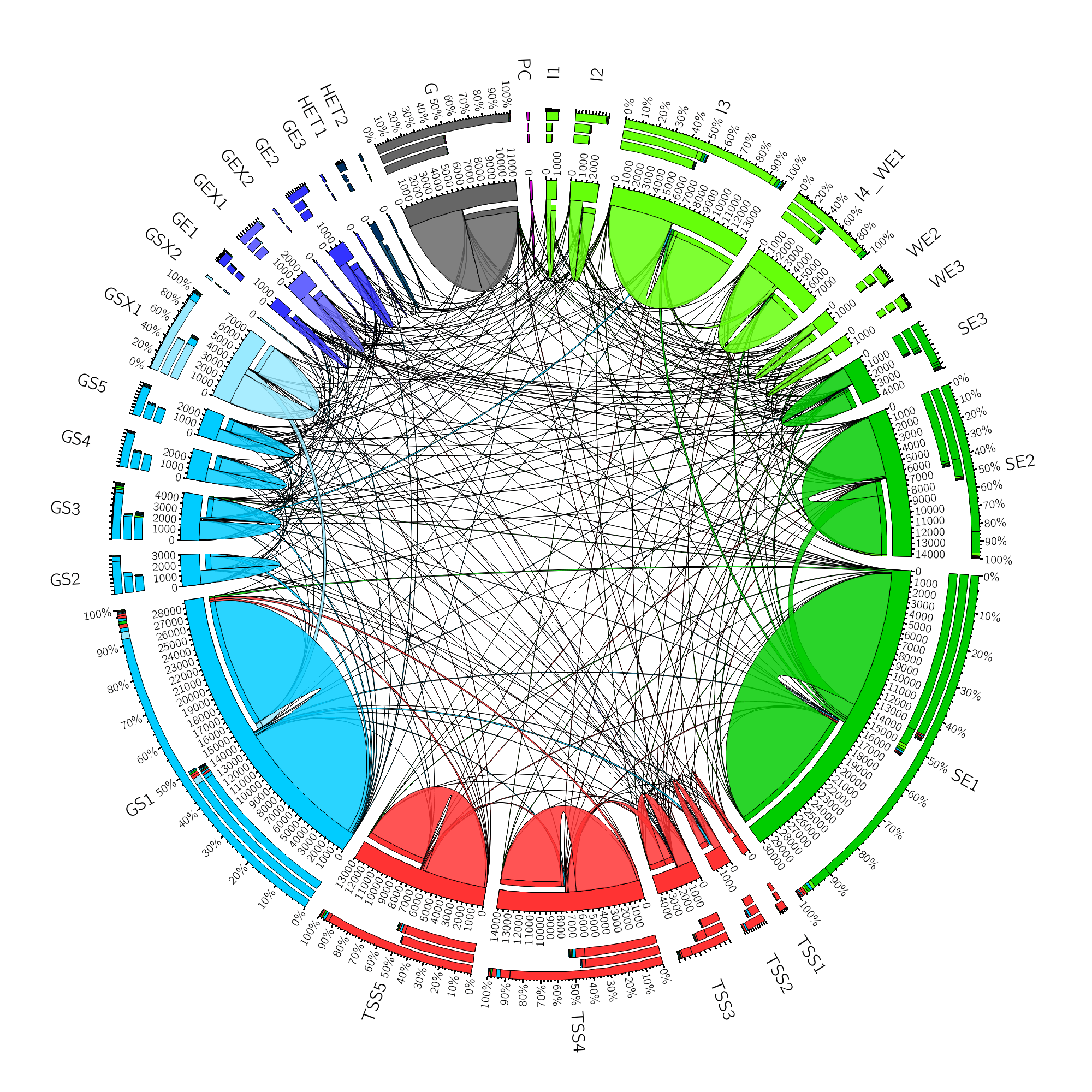

Supplement: Supplementary Data 4 — Effects of positive and negative perturbations of single chromatin factors on chromatin state identity. [file ncomms10528-s5.zip › Supplementary Data 4/NegativePerturbation/H3K9acS10P.png]

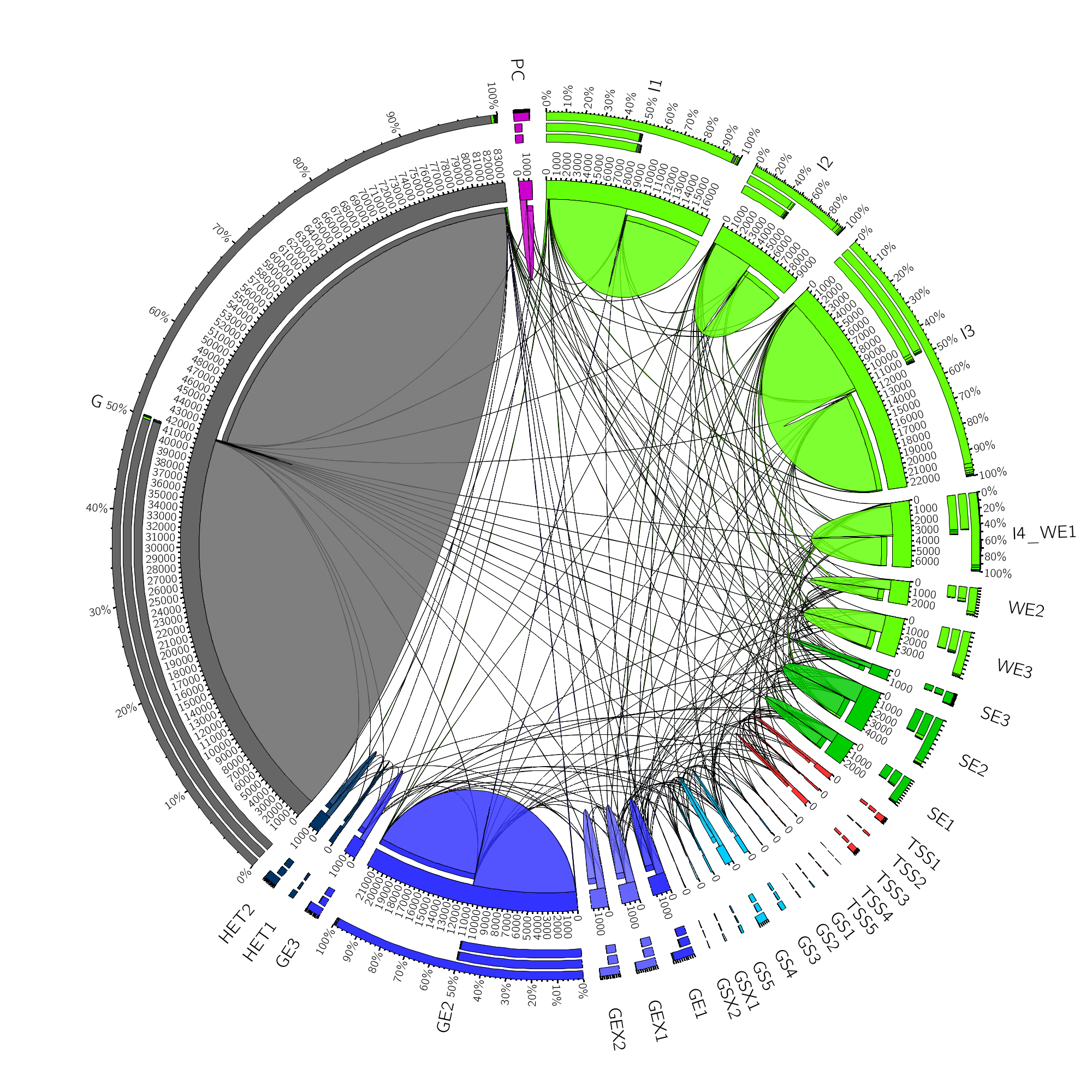

Supplement: Supplementary Data 4 — Effects of positive and negative perturbations of single chromatin factors on chromatin state identity. [file ncomms10528-s5.zip › Supplementary Data 4/NegativePerturbation/H3K9me1.png]

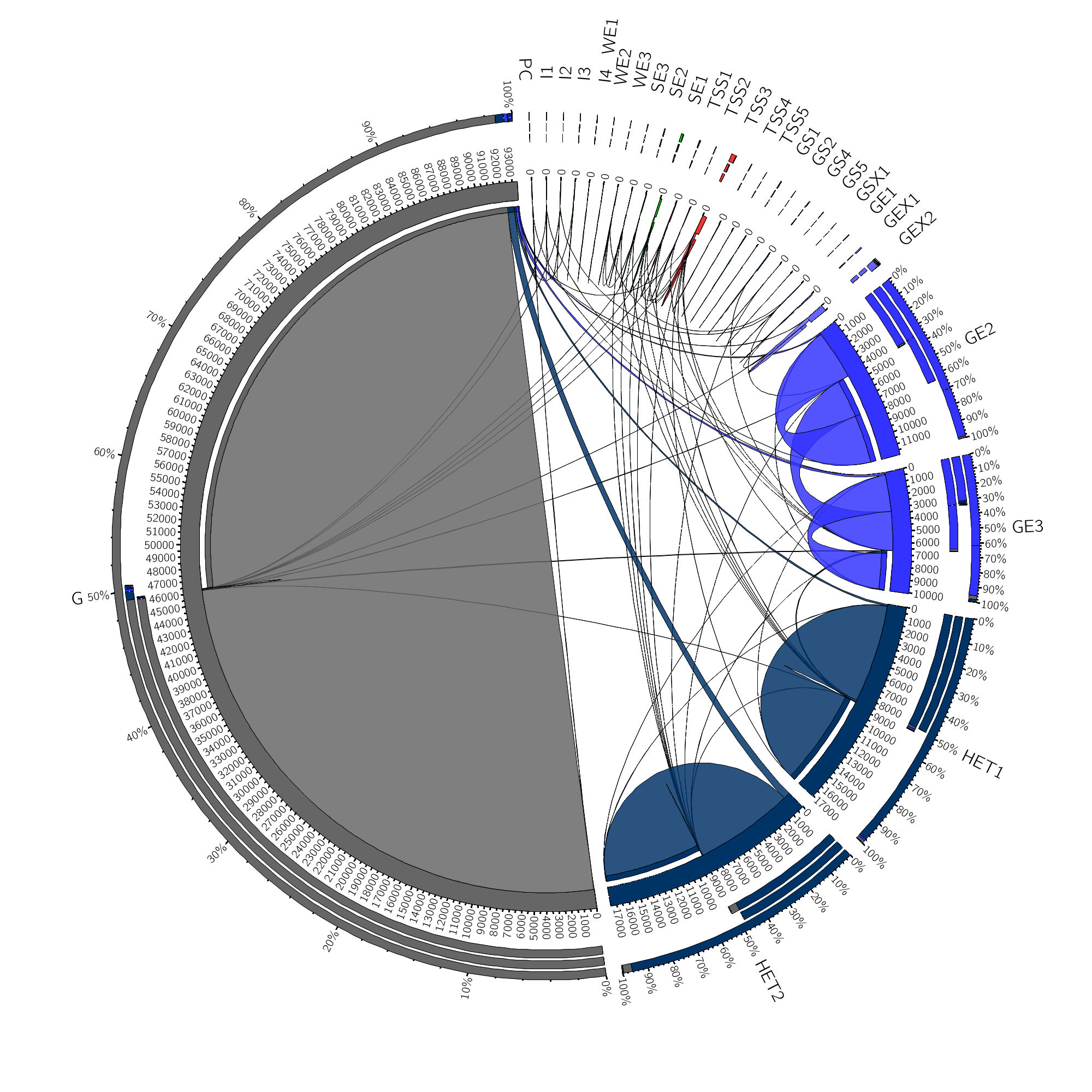

Supplement: Supplementary Data 4 — Effects of positive and negative perturbations of single chromatin factors on chromatin state identity. [file ncomms10528-s5.zip › Supplementary Data 4/NegativePerturbation/H3K9me2.png]

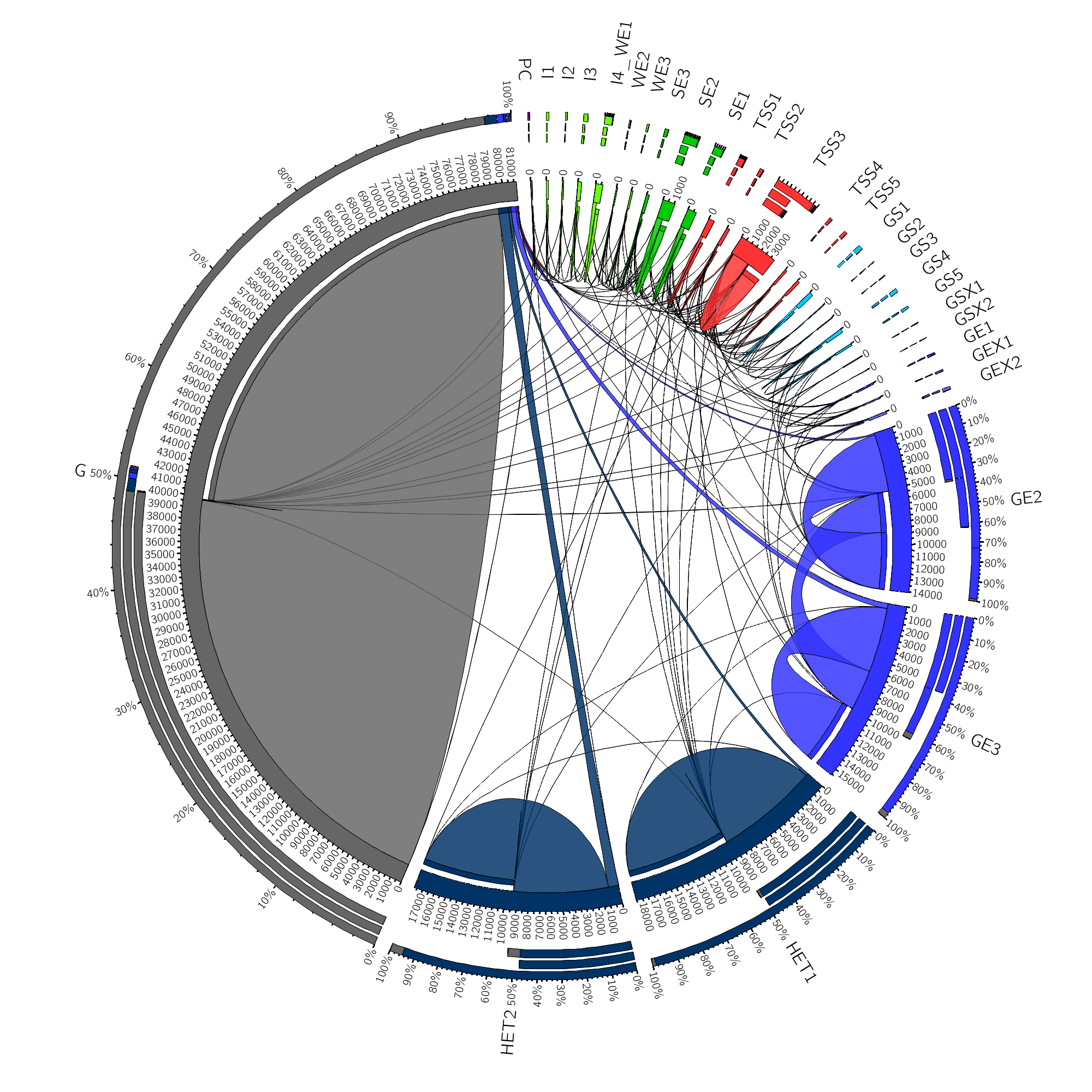

Supplement: Supplementary Data 4 — Effects of positive and negative perturbations of single chromatin factors on chromatin state identity. [file ncomms10528-s5.zip › Supplementary Data 4/NegativePerturbation/H3K9me3.png]

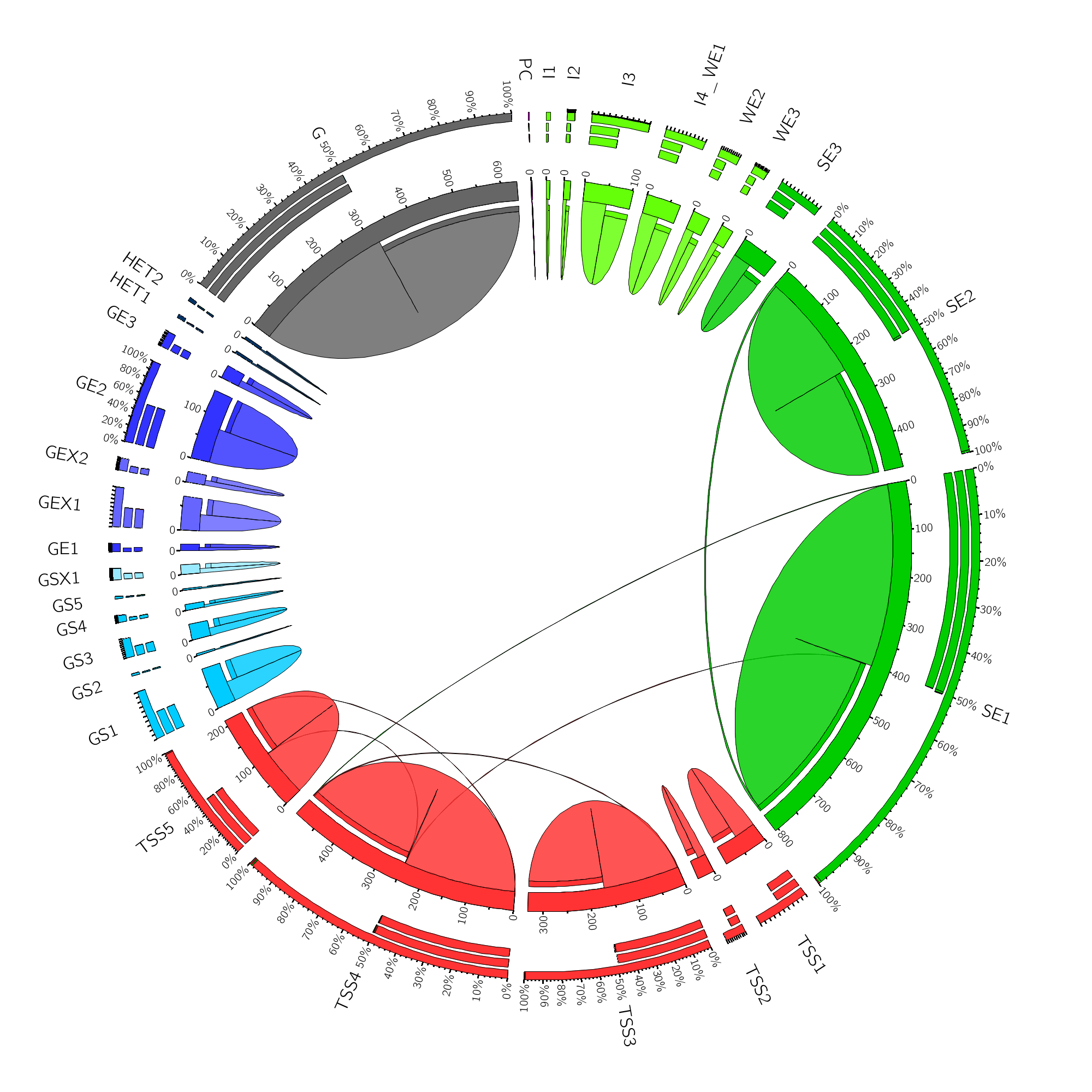

Supplement: Supplementary Data 4 — Effects of positive and negative perturbations of single chromatin factors on chromatin state identity. [file ncomms10528-s5.zip › Supplementary Data 4/NegativePerturbation/H4.png]

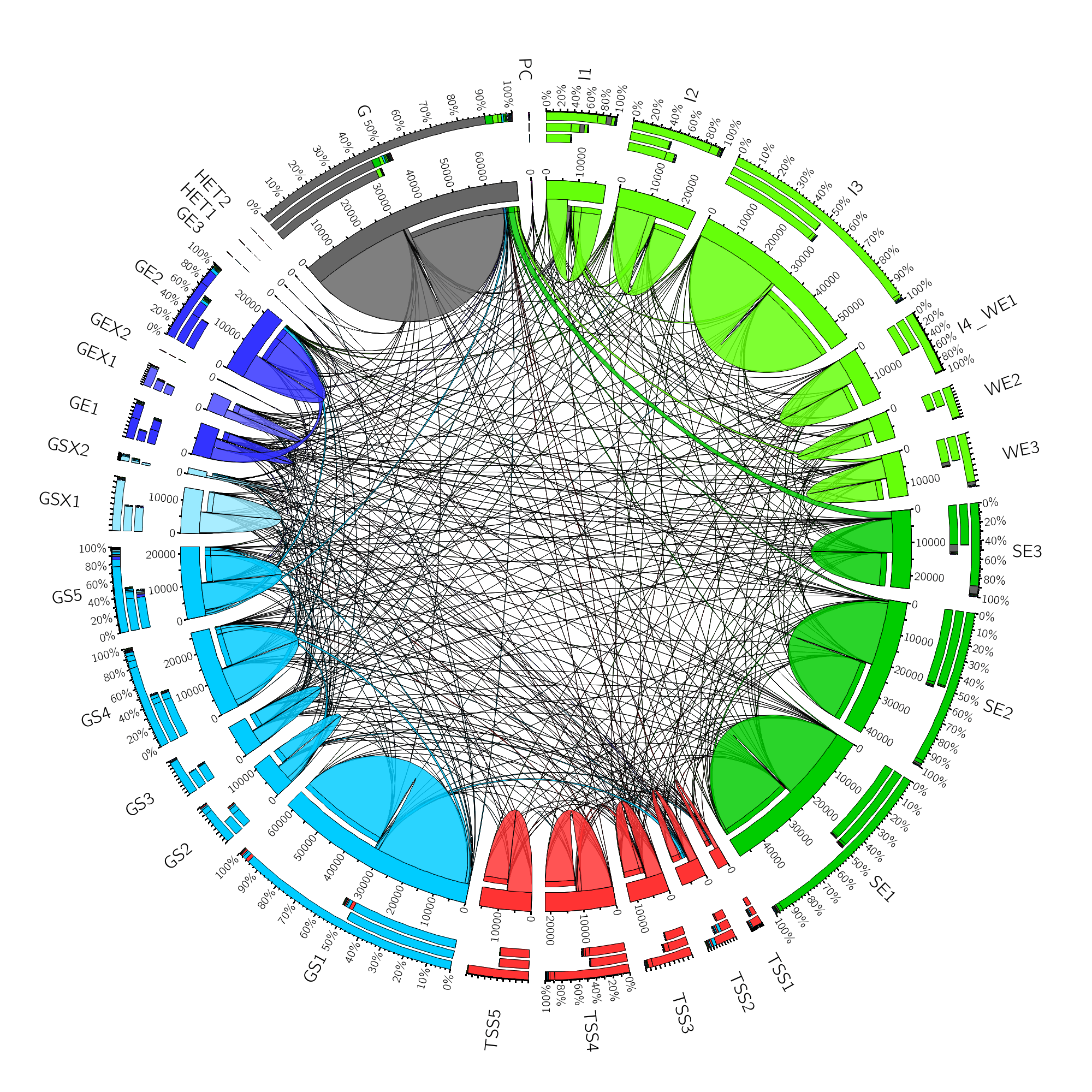

Supplement: Supplementary Data 4 — Effects of positive and negative perturbations of single chromatin factors on chromatin state identity. [file ncomms10528-s5.zip › Supplementary Data 4/NegativePerturbation/H4acTetra.png]

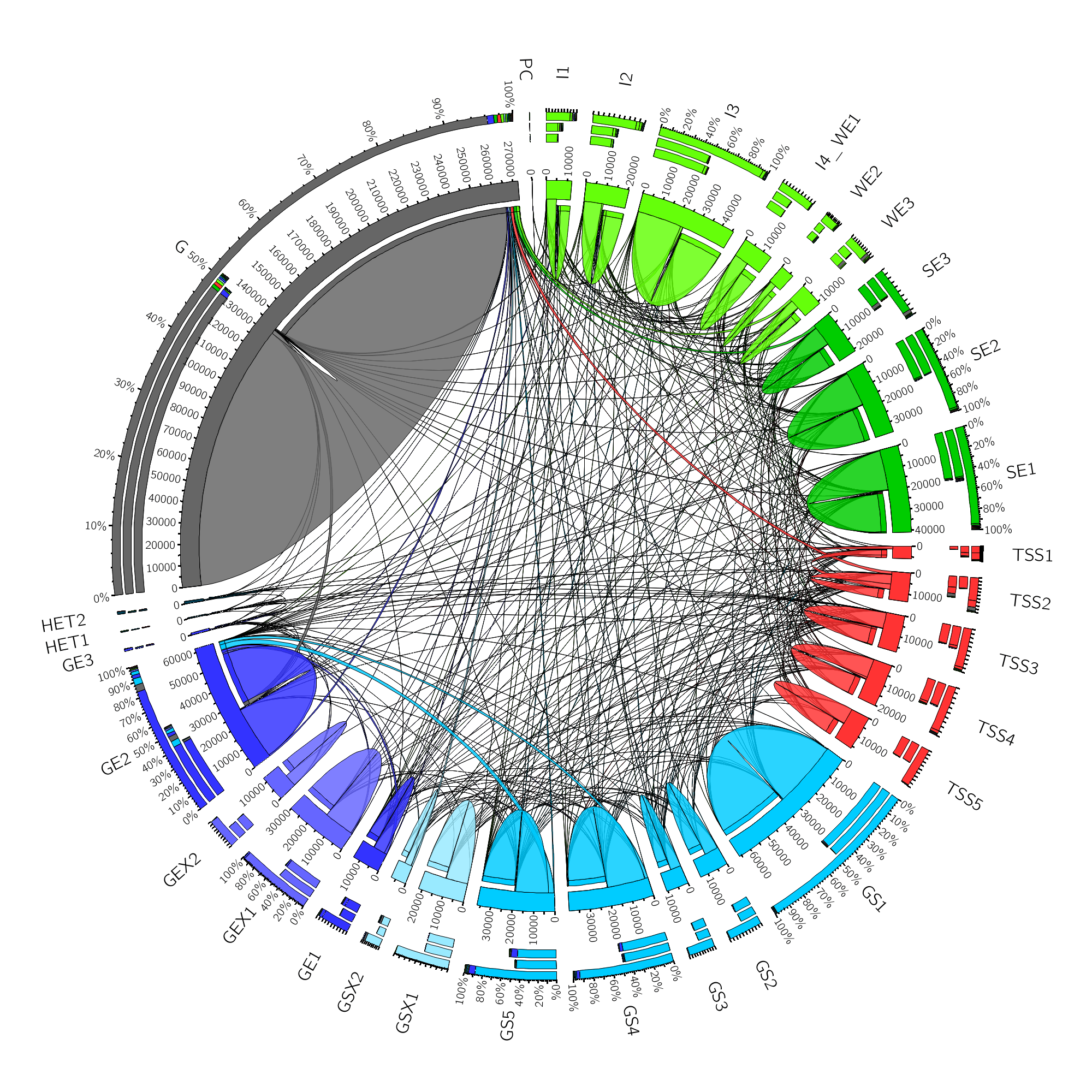

Supplement: Supplementary Data 4 — Effects of positive and negative perturbations of single chromatin factors on chromatin state identity. [file ncomms10528-s5.zip › Supplementary Data 4/NegativePerturbation/H4K16ac.png]

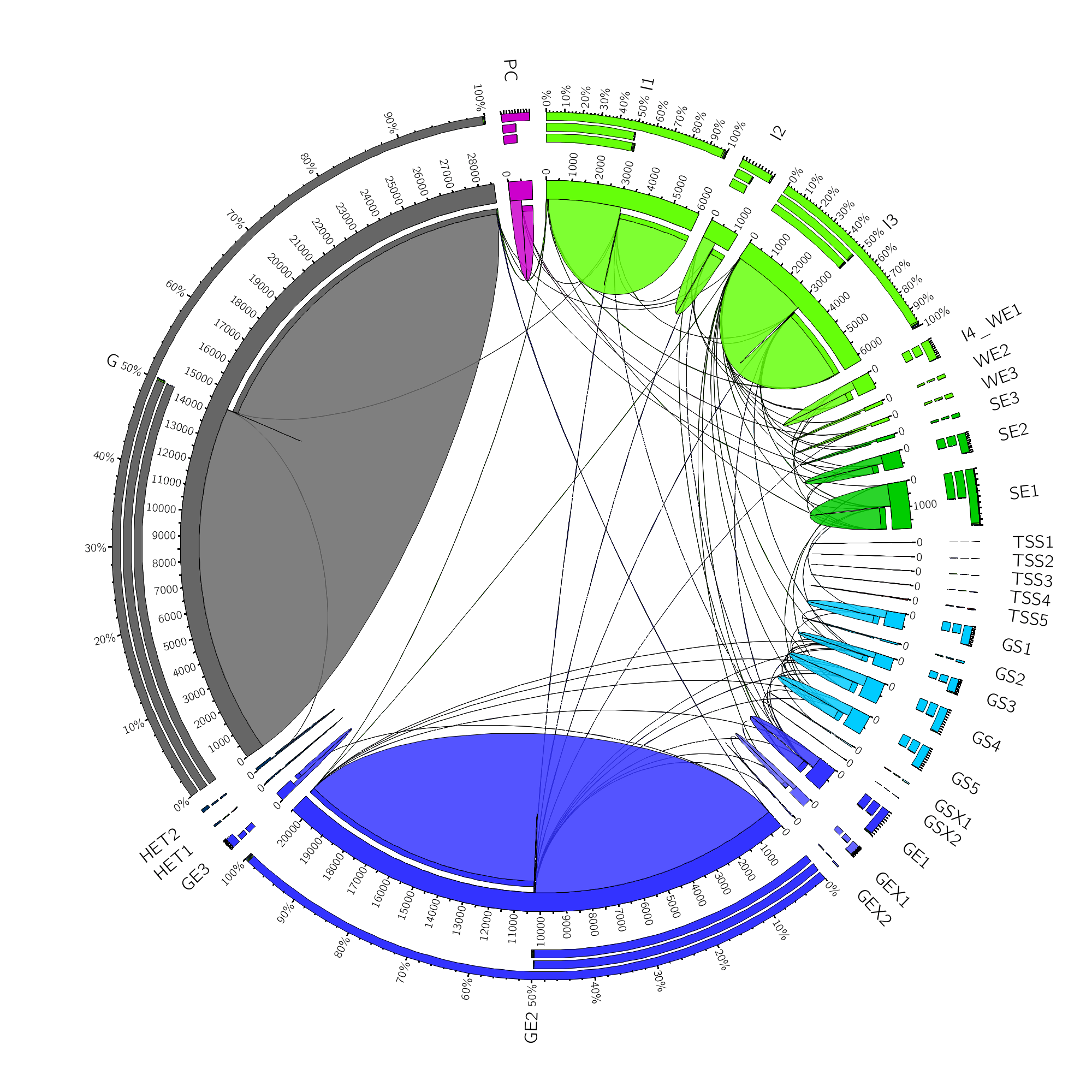

Supplement: Supplementary Data 4 — Effects of positive and negative perturbations of single chromatin factors on chromatin state identity. [file ncomms10528-s5.zip › Supplementary Data 4/NegativePerturbation/H4K20me1.png]

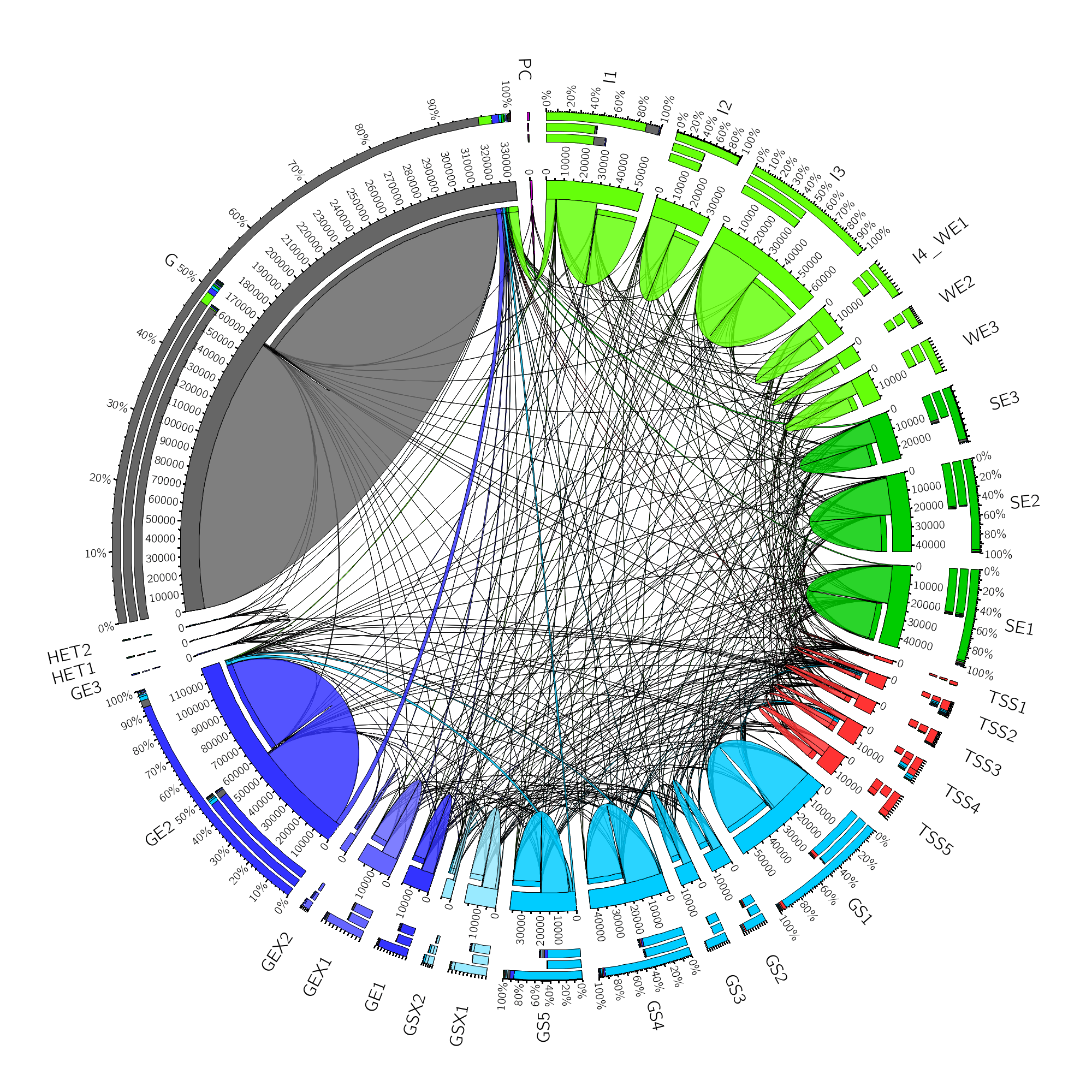

Supplement: Supplementary Data 4 — Effects of positive and negative perturbations of single chromatin factors on chromatin state identity. [file ncomms10528-s5.zip › Supplementary Data 4/NegativePerturbation/H4K5ac.png]

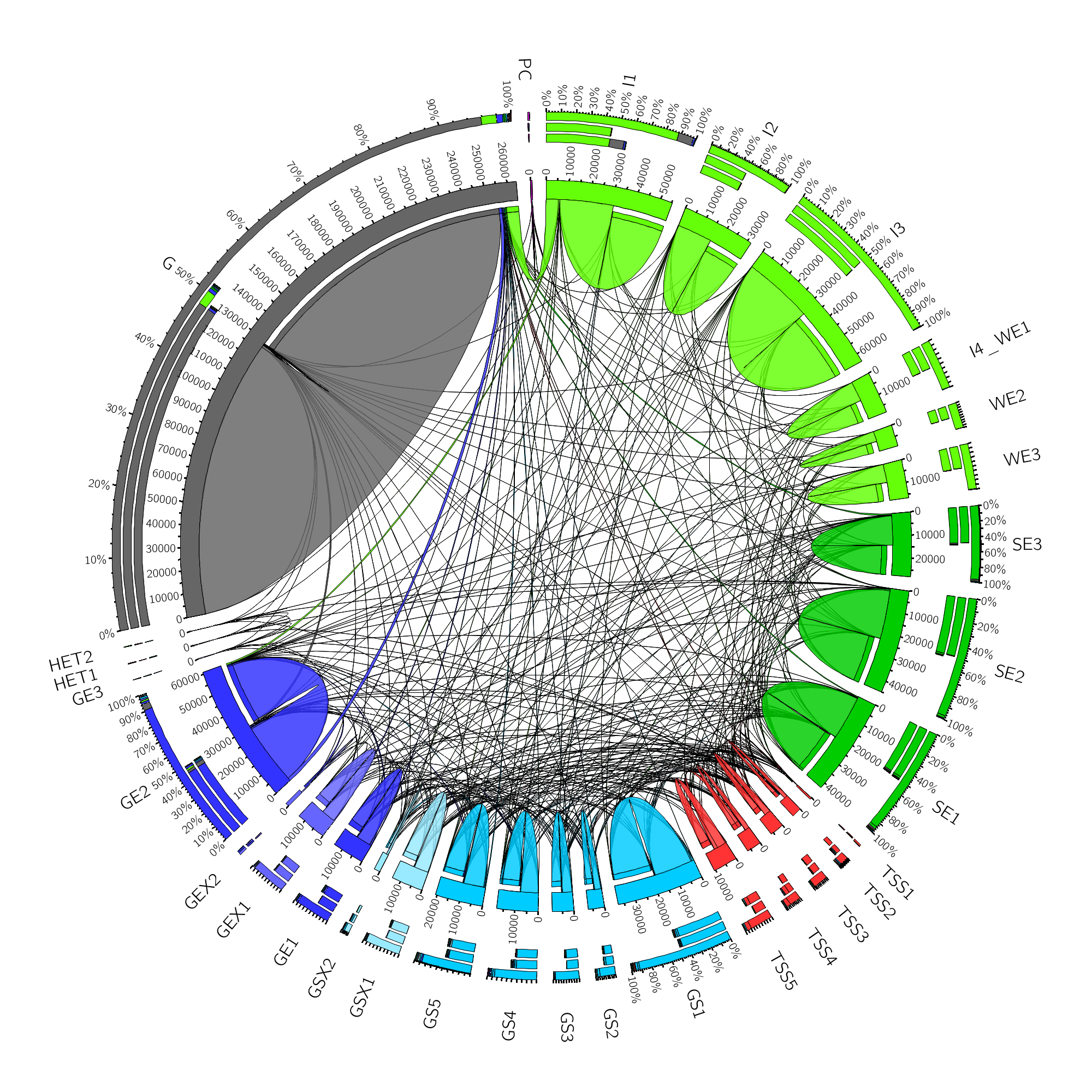

Supplement: Supplementary Data 4 — Effects of positive and negative perturbations of single chromatin factors on chromatin state identity. [file ncomms10528-s5.zip › Supplementary Data 4/NegativePerturbation/H4K8ac.png]

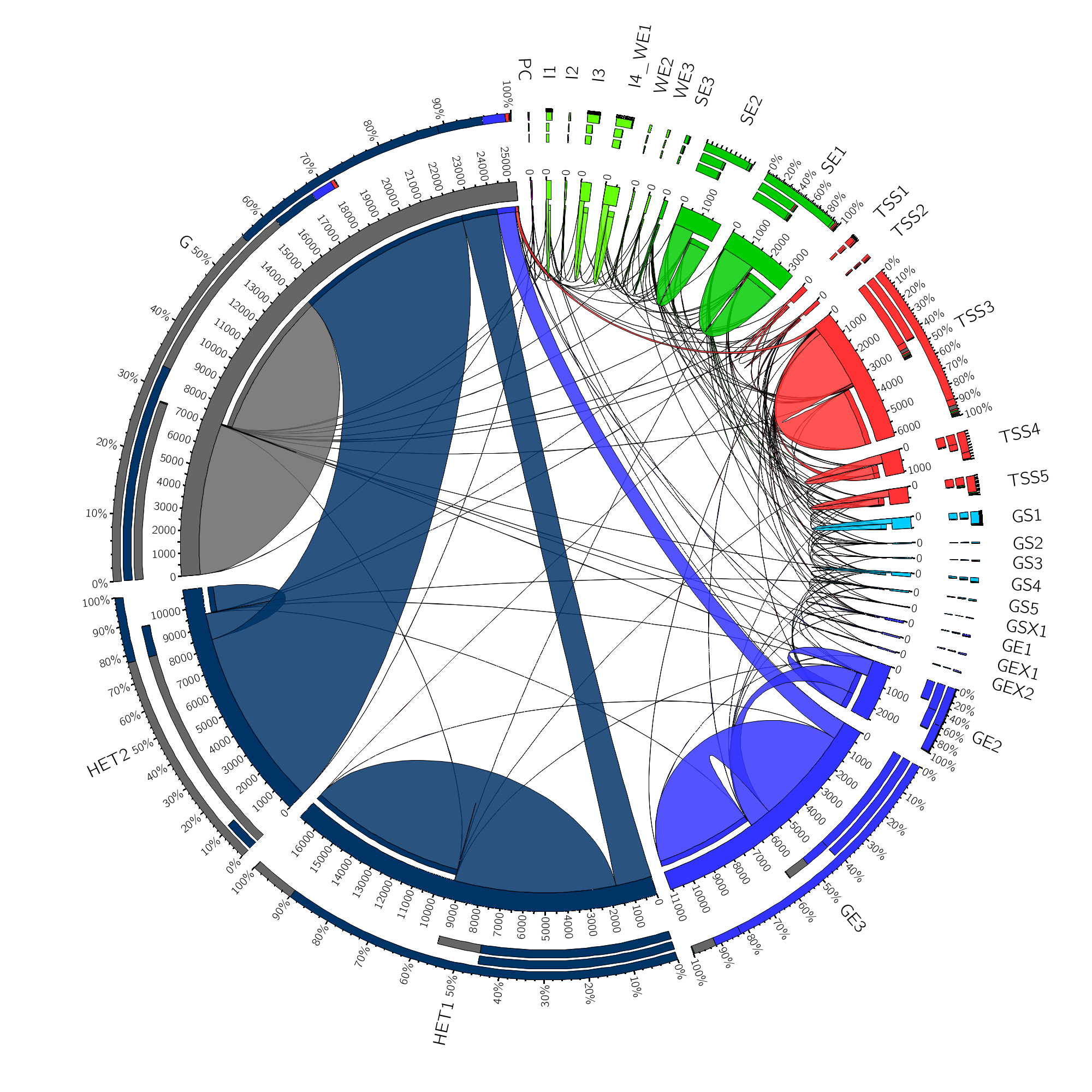

Supplement: Supplementary Data 4 — Effects of positive and negative perturbations of single chromatin factors on chromatin state identity. [file ncomms10528-s5.zip › Supplementary Data 4/NegativePerturbation/HP1a.png]

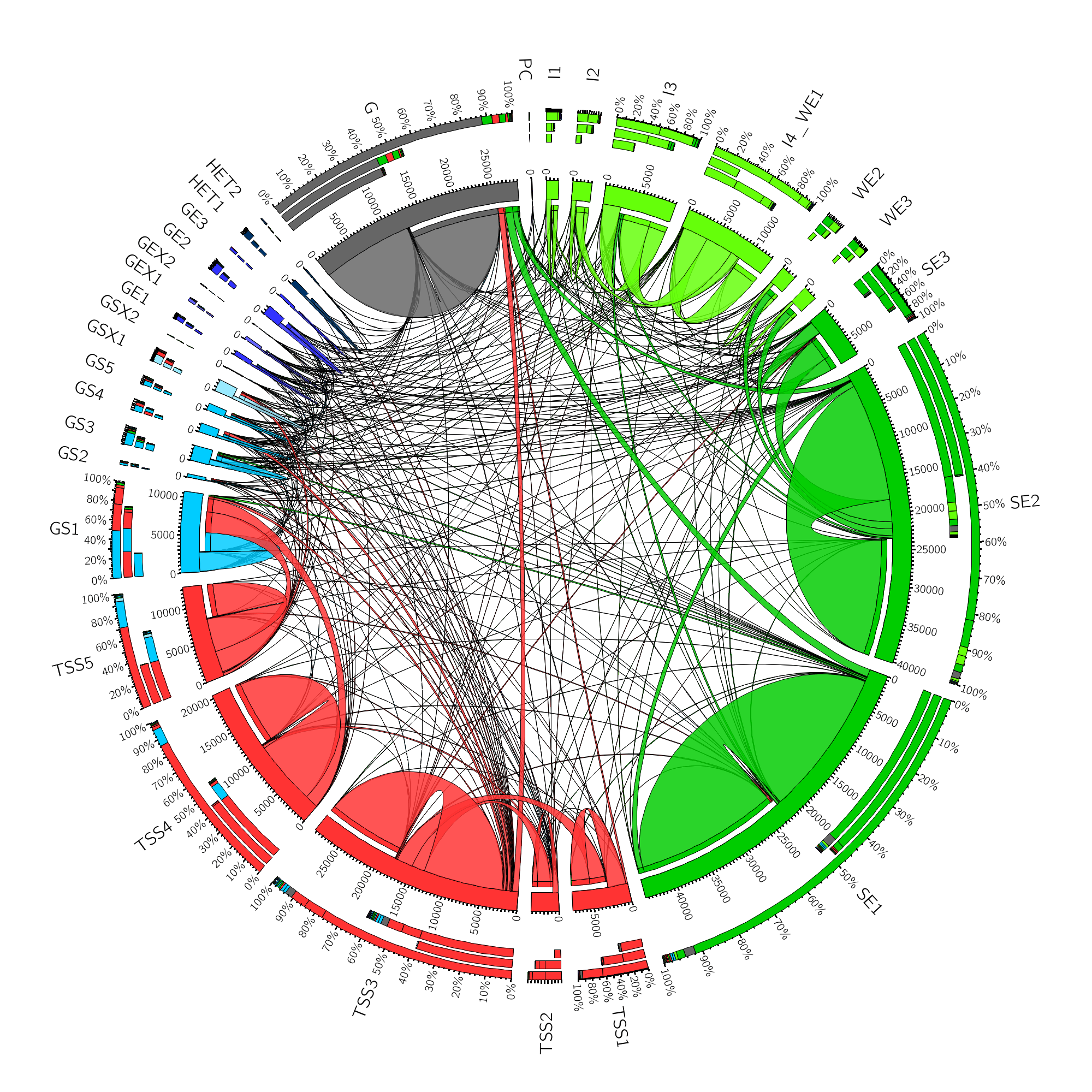

Supplement: Supplementary Data 4 — Effects of positive and negative perturbations of single chromatin factors on chromatin state identity. [file ncomms10528-s5.zip › Supplementary Data 4/NegativePerturbation/HP1b.png]

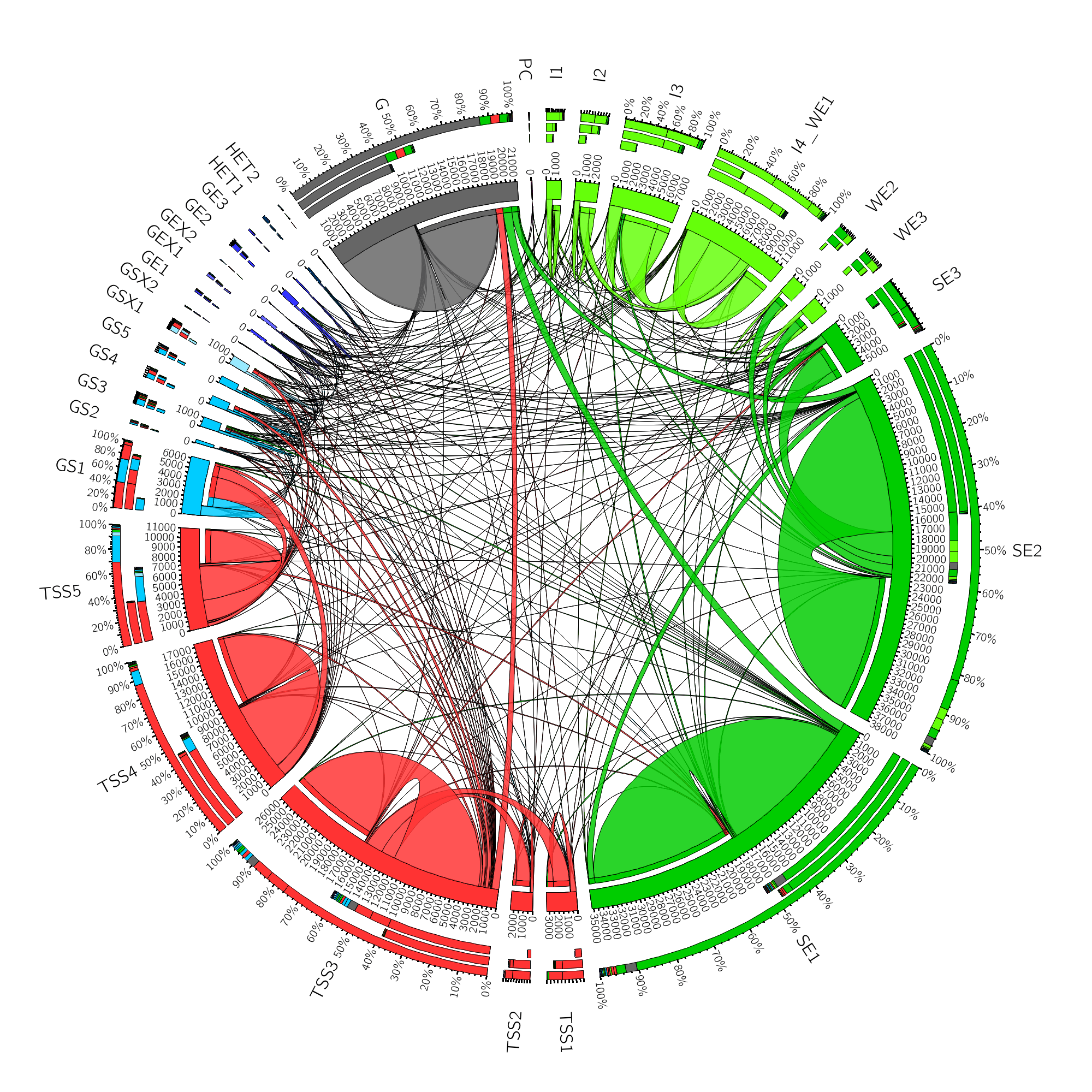

Supplement: Supplementary Data 4 — Effects of positive and negative perturbations of single chromatin factors on chromatin state identity. [file ncomms10528-s5.zip › Supplementary Data 4/NegativePerturbation/HP1c.png]

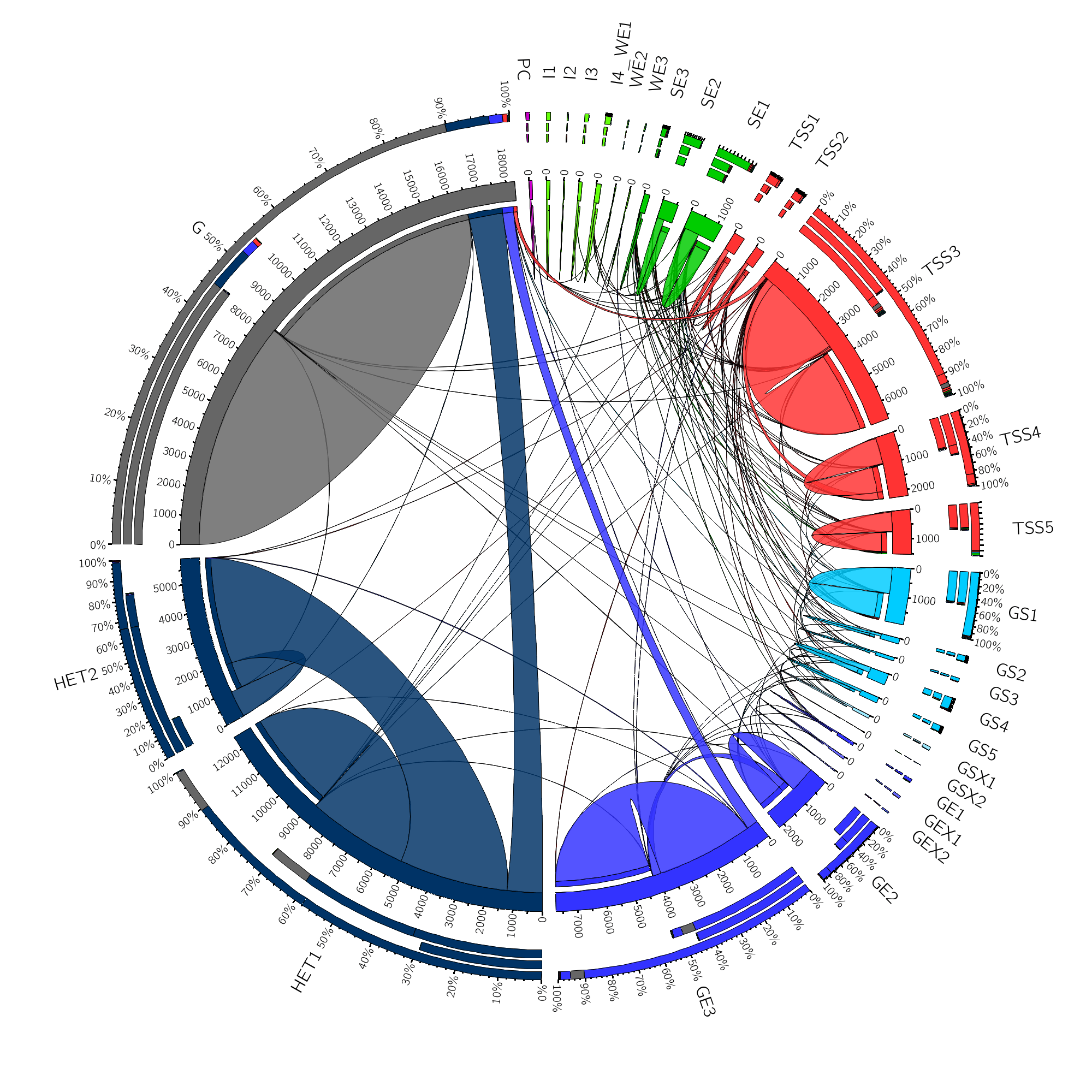

Supplement: Supplementary Data 4 — Effects of positive and negative perturbations of single chromatin factors on chromatin state identity. [file ncomms10528-s5.zip › Supplementary Data 4/NegativePerturbation/HP2.png]

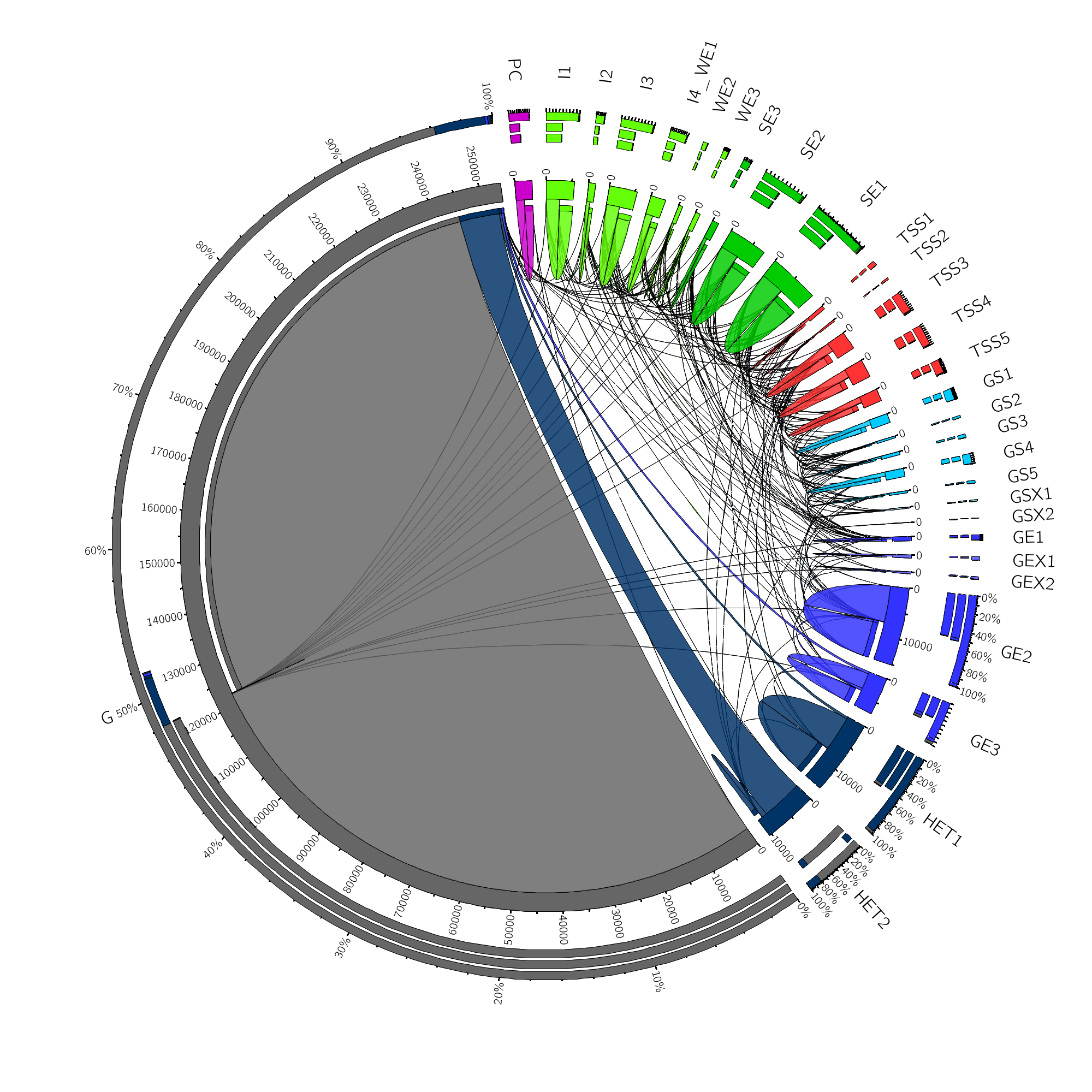

Supplement: Supplementary Data 4 — Effects of positive and negative perturbations of single chromatin factors on chromatin state identity. [file ncomms10528-s5.zip › Supplementary Data 4/NegativePerturbation/HP4.png]

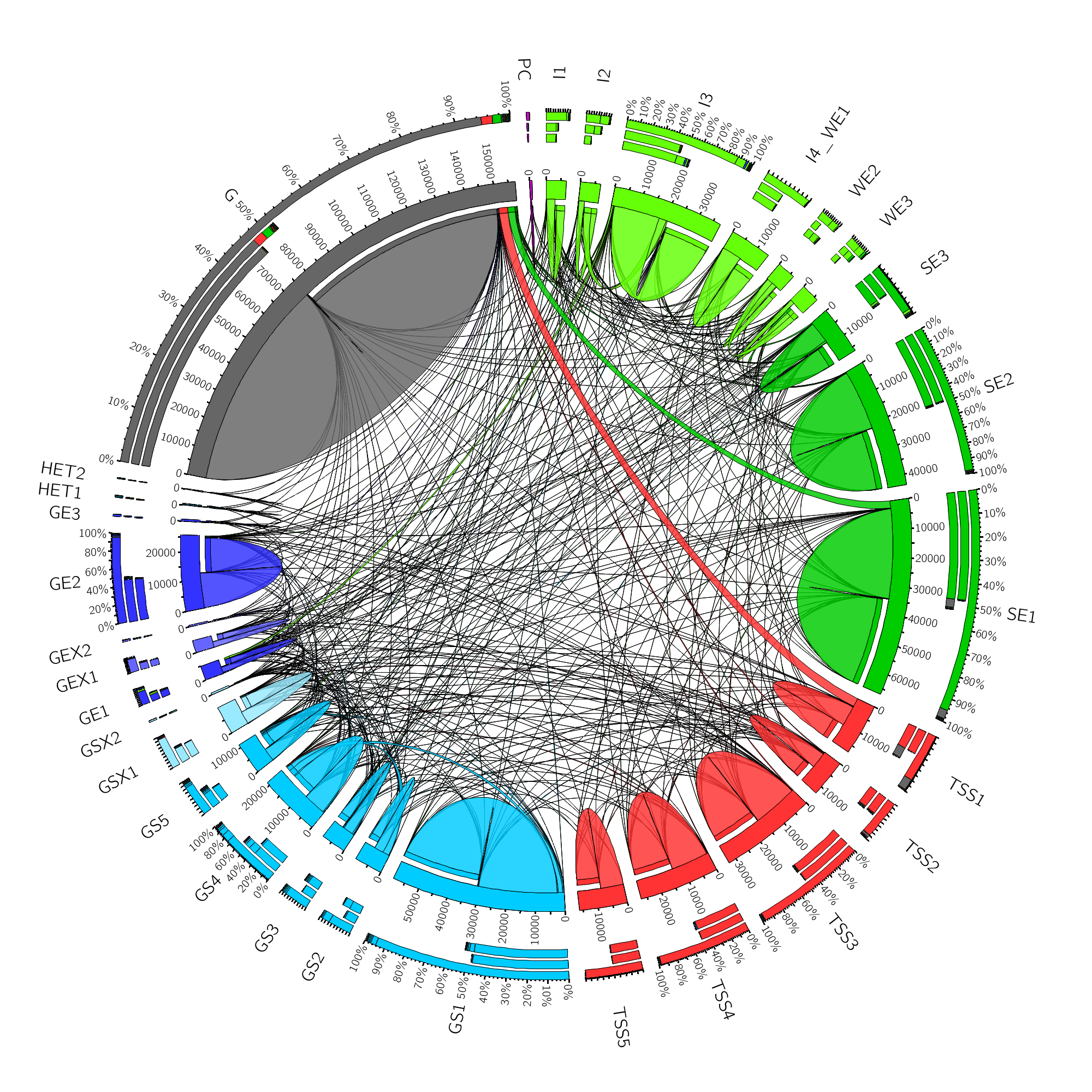

Supplement: Supplementary Data 4 — Effects of positive and negative perturbations of single chromatin factors on chromatin state identity. [file ncomms10528-s5.zip › Supplementary Data 4/NegativePerturbation/ISWI.png]

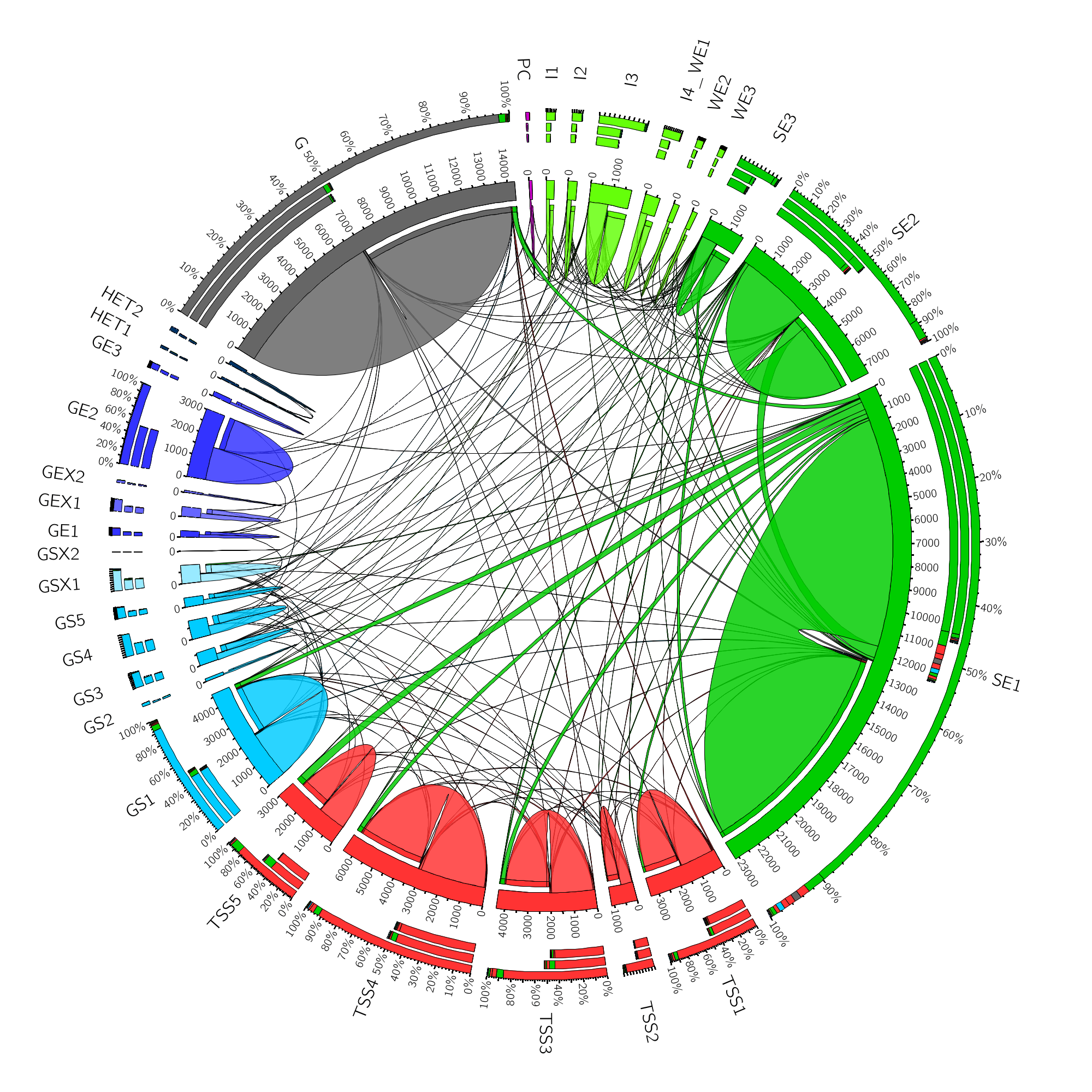

Supplement: Supplementary Data 4 — Effects of positive and negative perturbations of single chromatin factors on chromatin state identity. [file ncomms10528-s5.zip › Supplementary Data 4/NegativePerturbation/JHDM1.png]

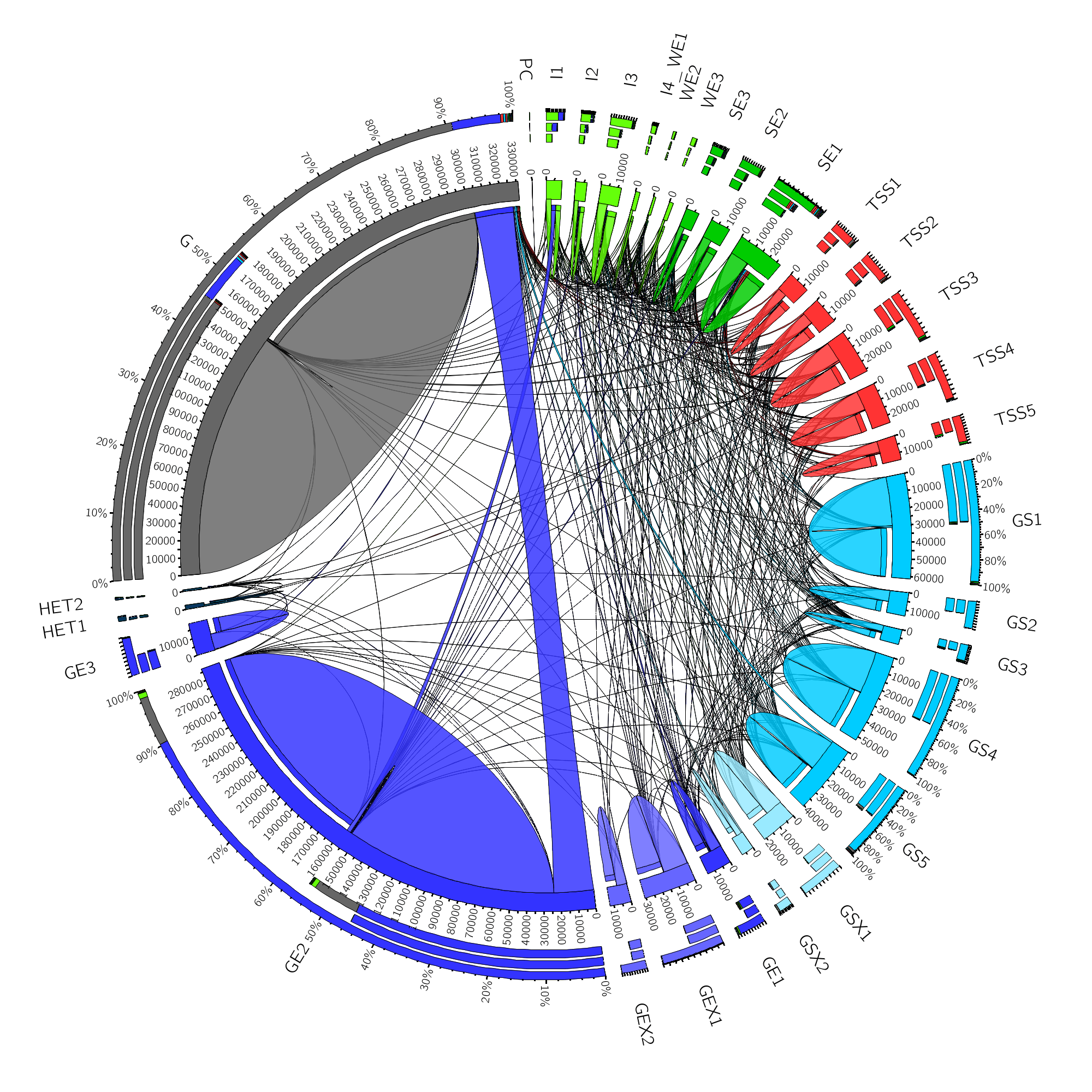

Supplement: Supplementary Data 4 — Effects of positive and negative perturbations of single chromatin factors on chromatin state identity. [file ncomms10528-s5.zip › Supplementary Data 4/NegativePerturbation/JIL1.png]

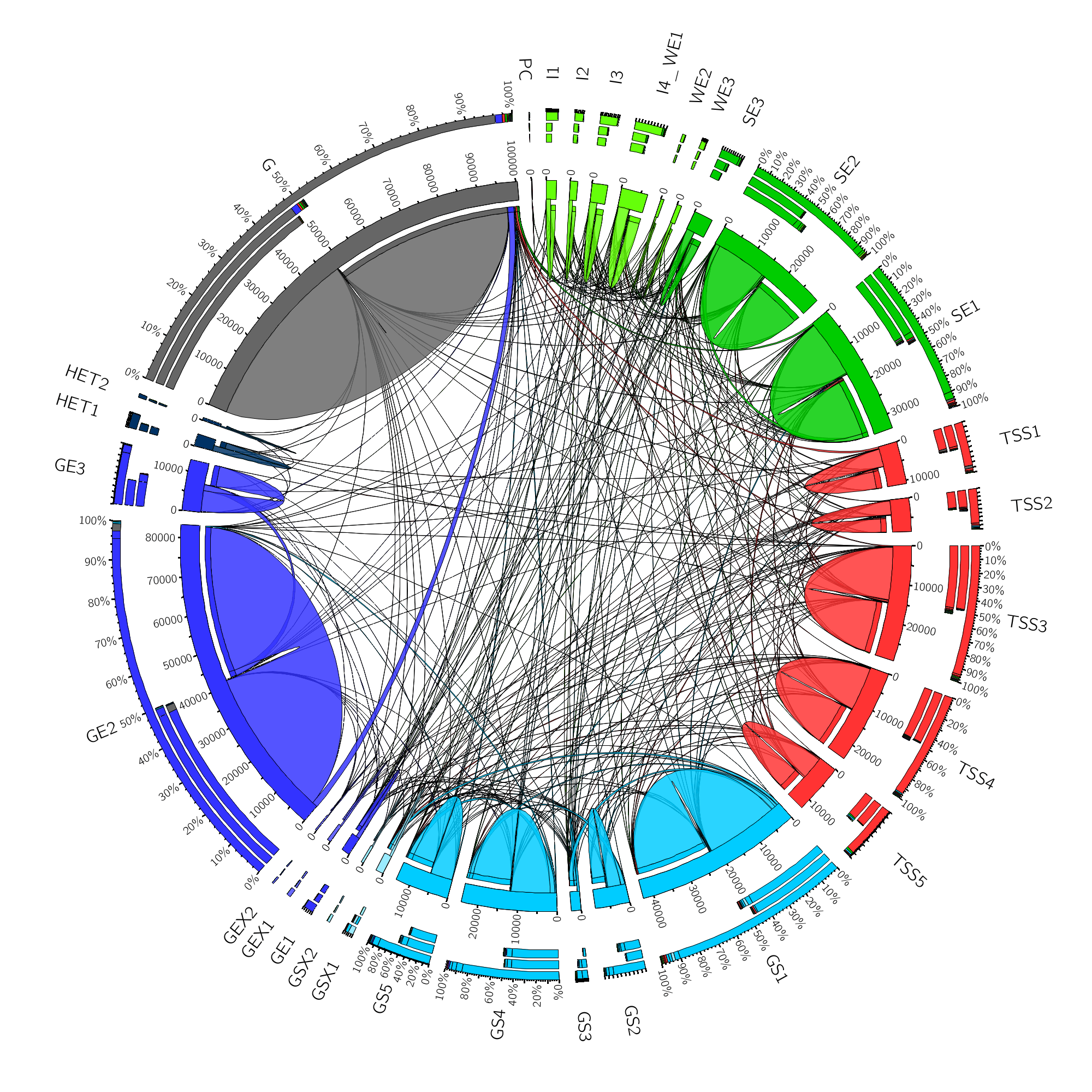

Supplement: Supplementary Data 4 — Effects of positive and negative perturbations of single chromatin factors on chromatin state identity. [file ncomms10528-s5.zip › Supplementary Data 4/NegativePerturbation/JMJD2AKDM4A.png]

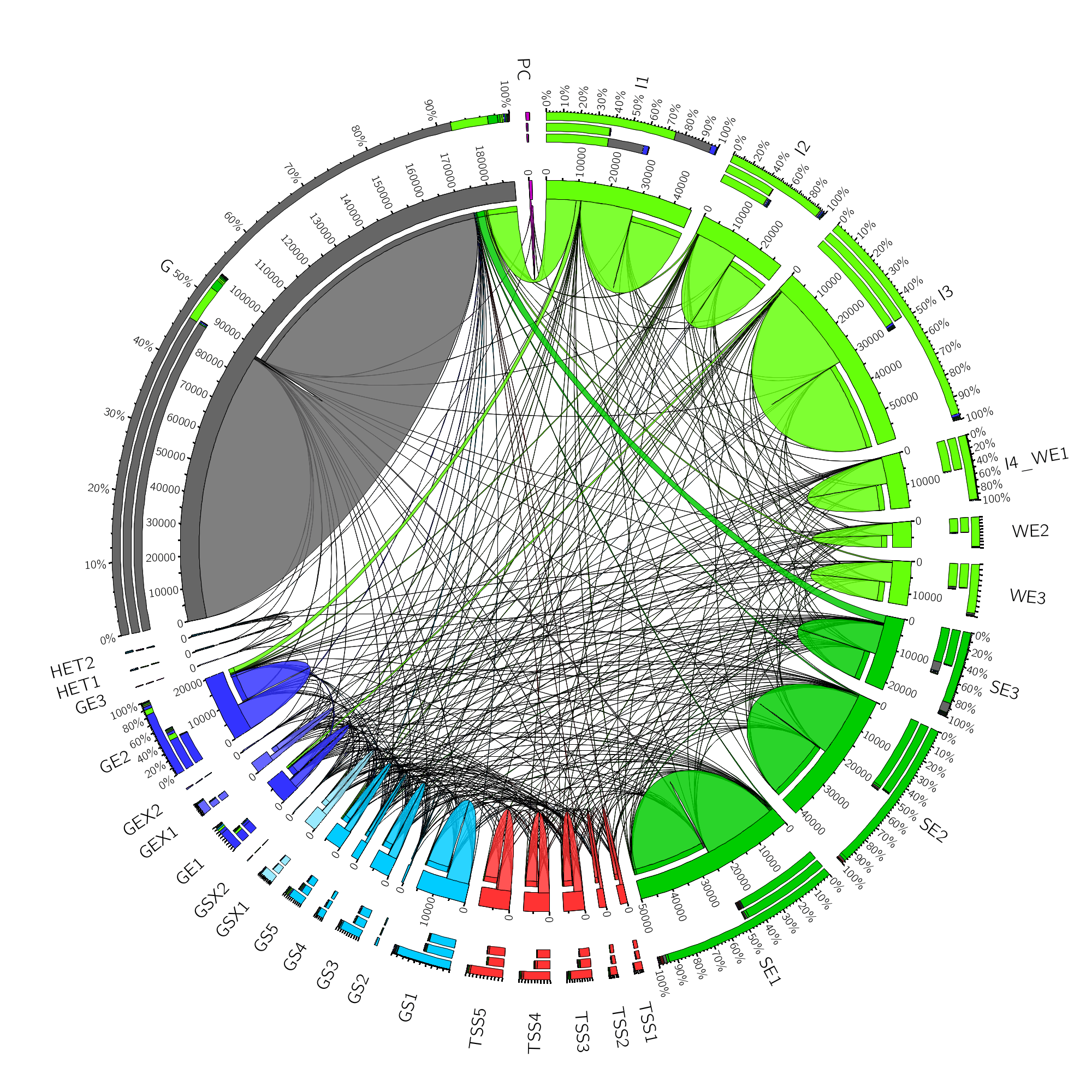

Supplement: Supplementary Data 4 — Effects of positive and negative perturbations of single chromatin factors on chromatin state identity. [file ncomms10528-s5.zip › Supplementary Data 4/NegativePerturbation/LSD1.png]

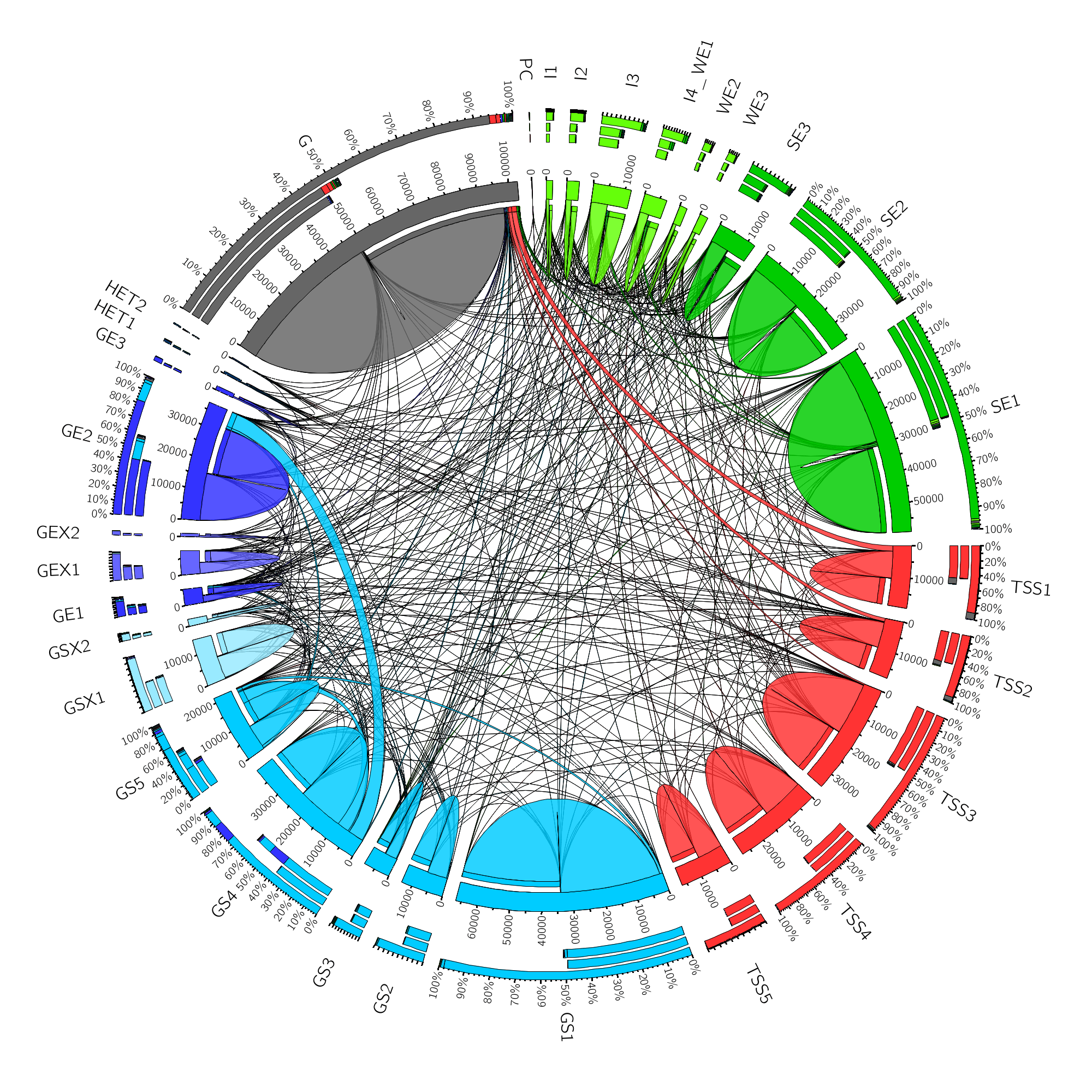

Supplement: Supplementary Data 4 — Effects of positive and negative perturbations of single chromatin factors on chromatin state identity. [file ncomms10528-s5.zip › Supplementary Data 4/NegativePerturbation/MBDR2.png]

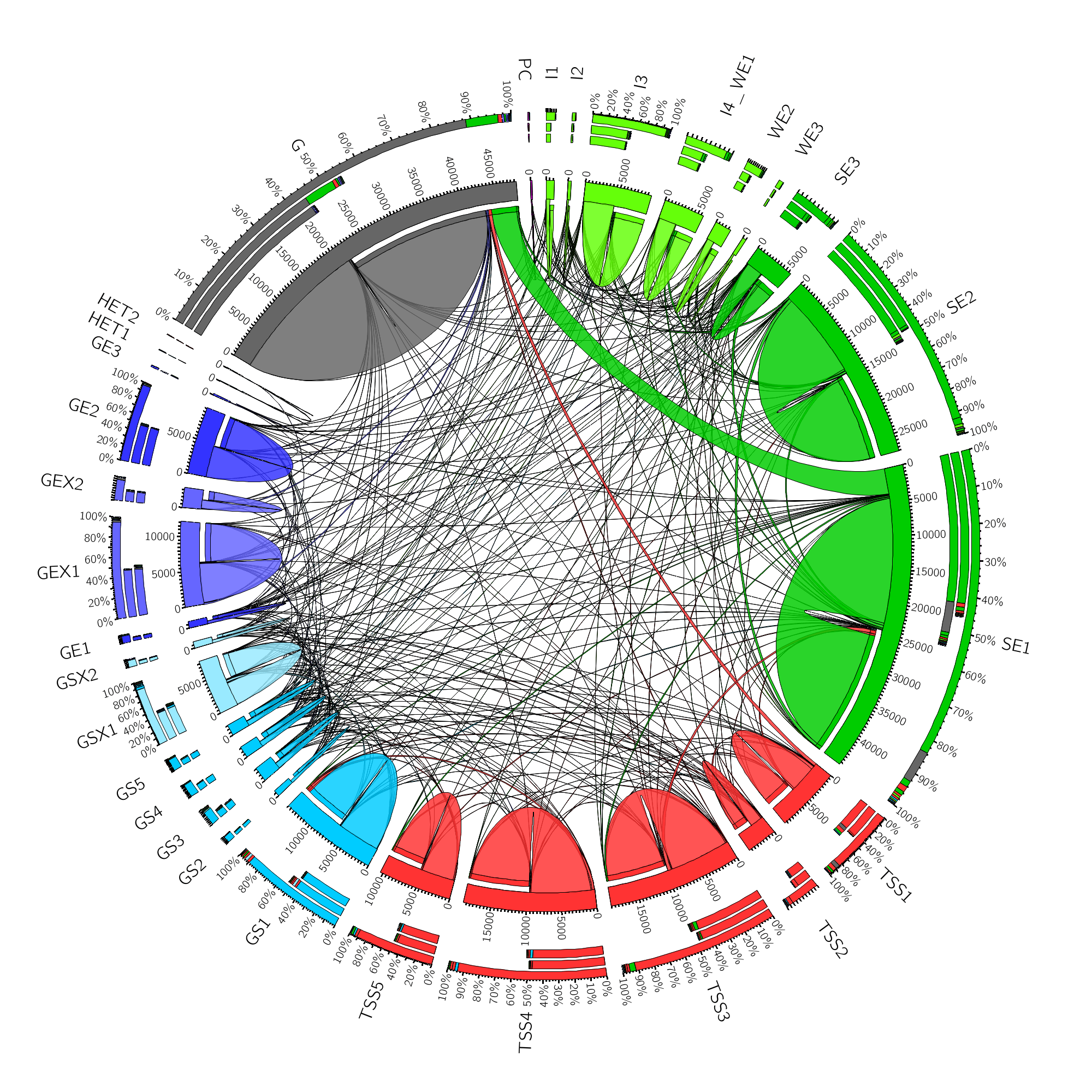

Supplement: Supplementary Data 4 — Effects of positive and negative perturbations of single chromatin factors on chromatin state identity. [file ncomms10528-s5.zip › Supplementary Data 4/NegativePerturbation/MLE.png]

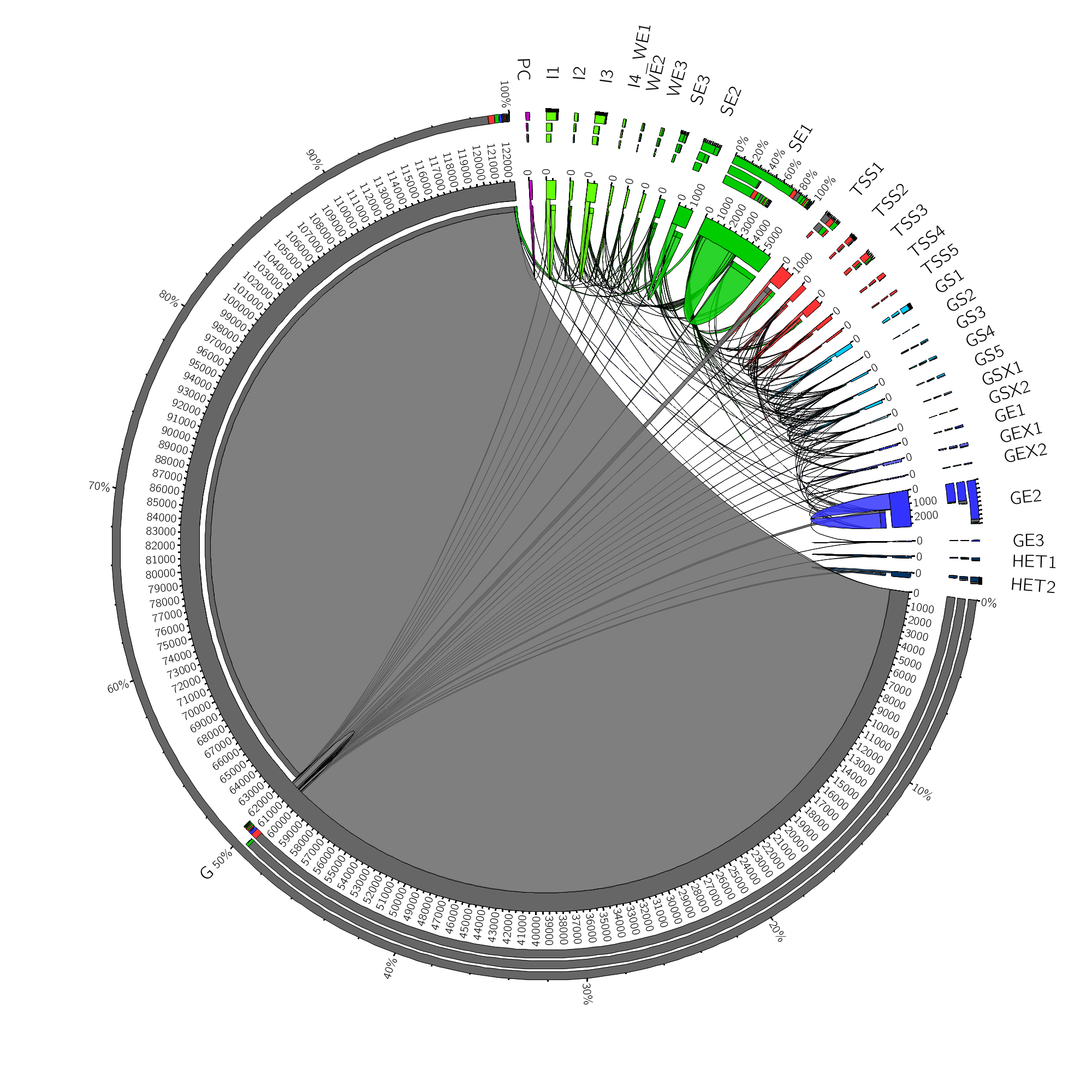

Supplement: Supplementary Data 4 — Effects of positive and negative perturbations of single chromatin factors on chromatin state identity. [file ncomms10528-s5.zip › Supplementary Data 4/NegativePerturbation/modmdg4.png]

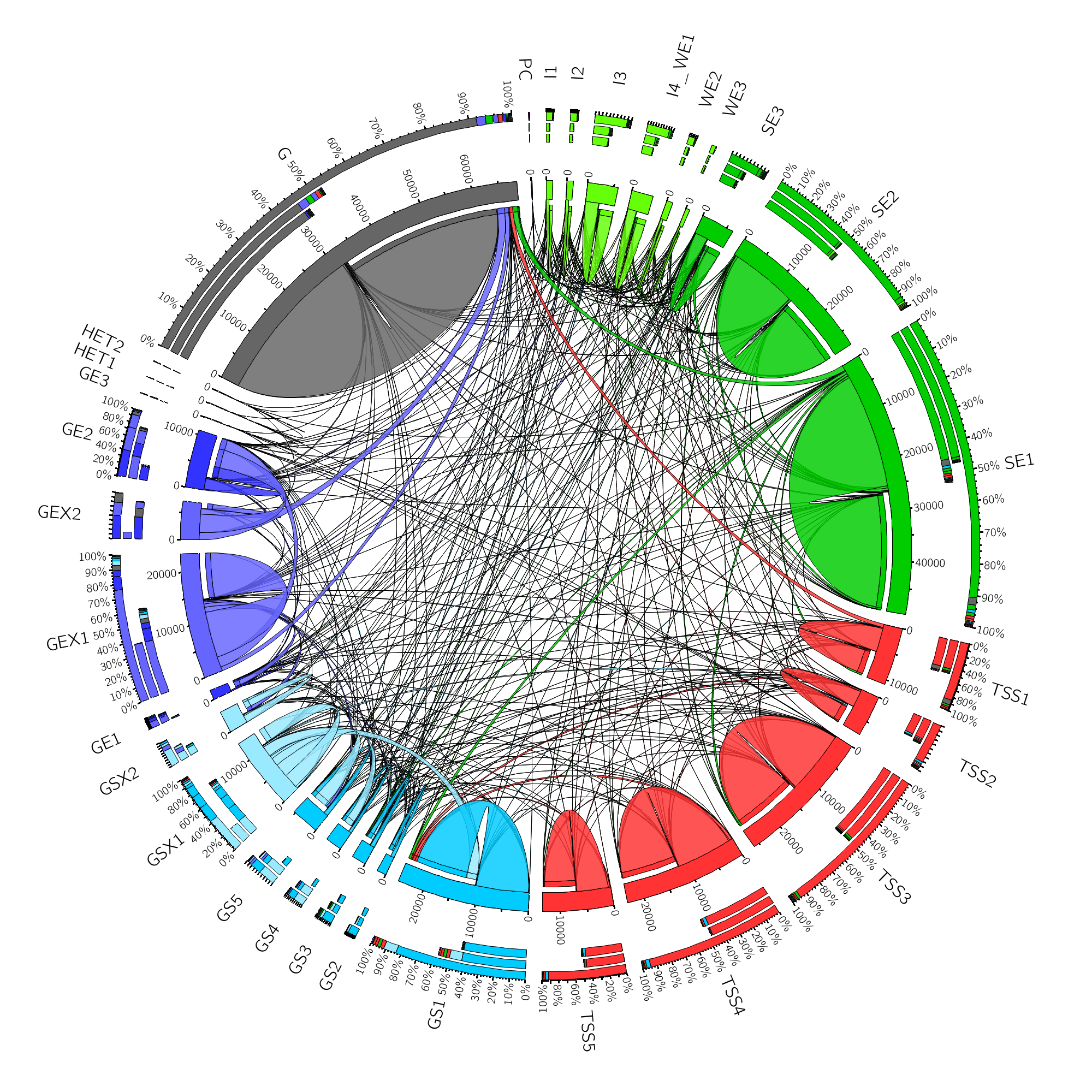

Supplement: Supplementary Data 4 — Effects of positive and negative perturbations of single chromatin factors on chromatin state identity. [file ncomms10528-s5.zip › Supplementary Data 4/NegativePerturbation/MOF.png]

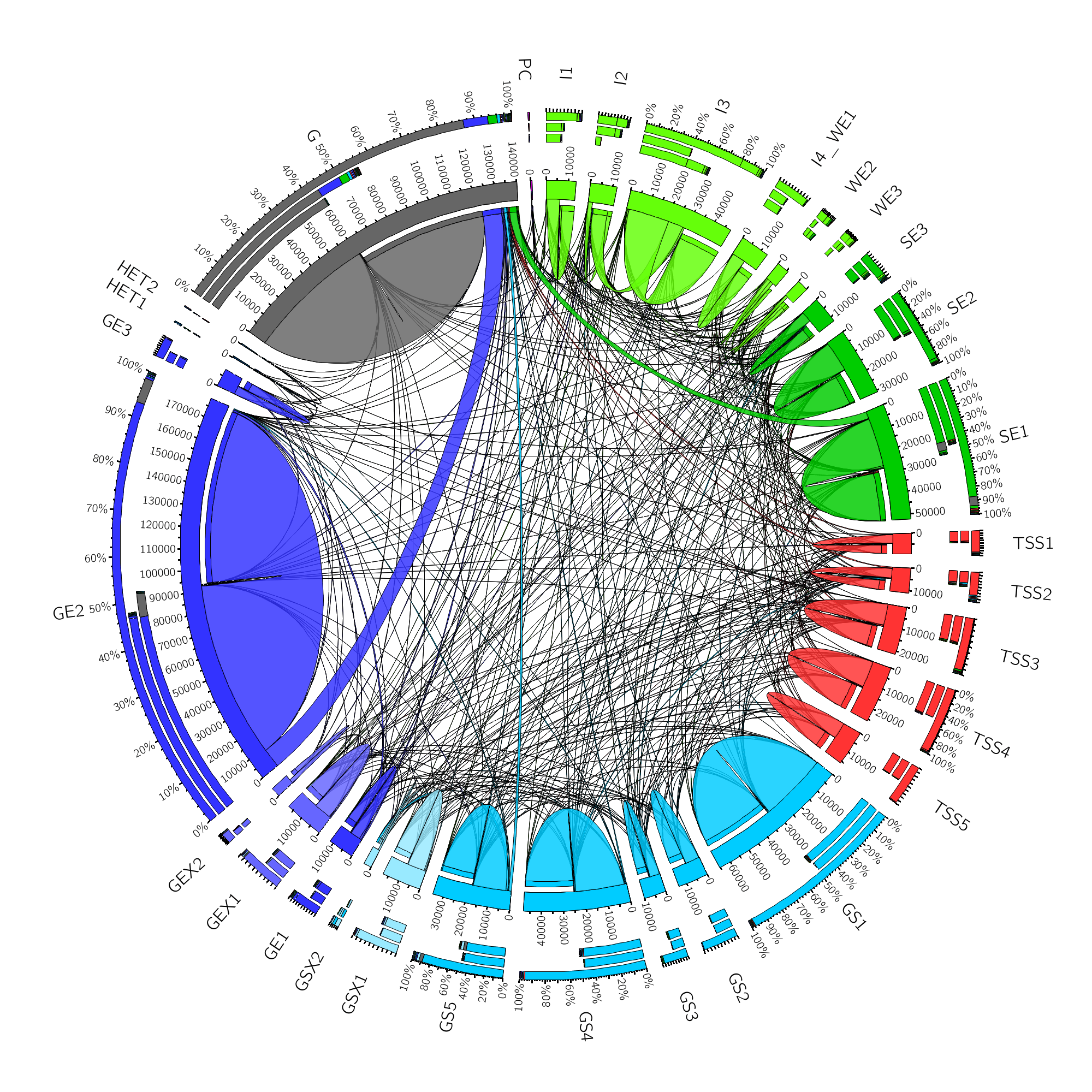

Supplement: Supplementary Data 4 — Effects of positive and negative perturbations of single chromatin factors on chromatin state identity. [file ncomms10528-s5.zip › Supplementary Data 4/NegativePerturbation/MRG15.png]

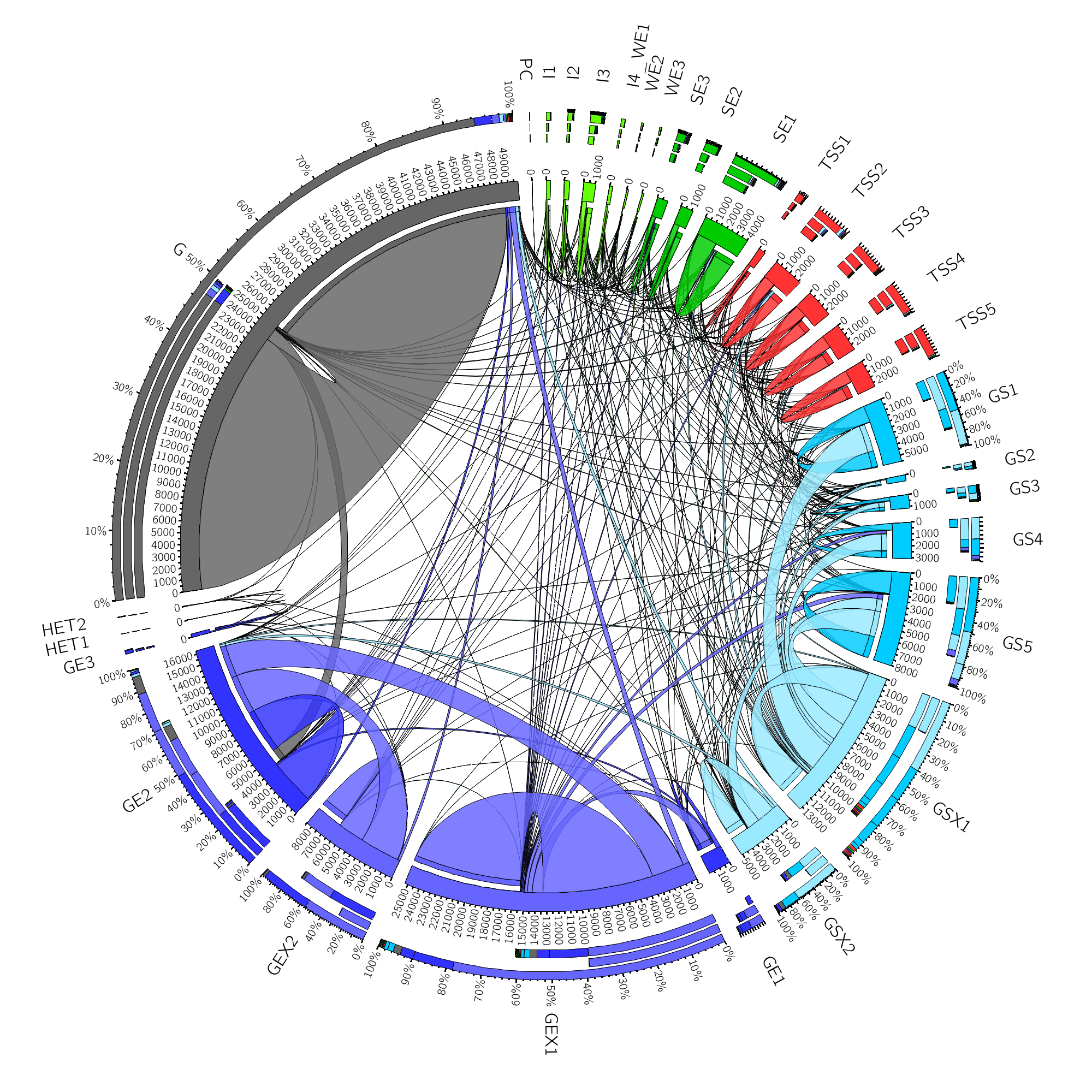

Supplement: Supplementary Data 4 — Effects of positive and negative perturbations of single chromatin factors on chromatin state identity. [file ncomms10528-s5.zip › Supplementary Data 4/NegativePerturbation/MSL1.png]

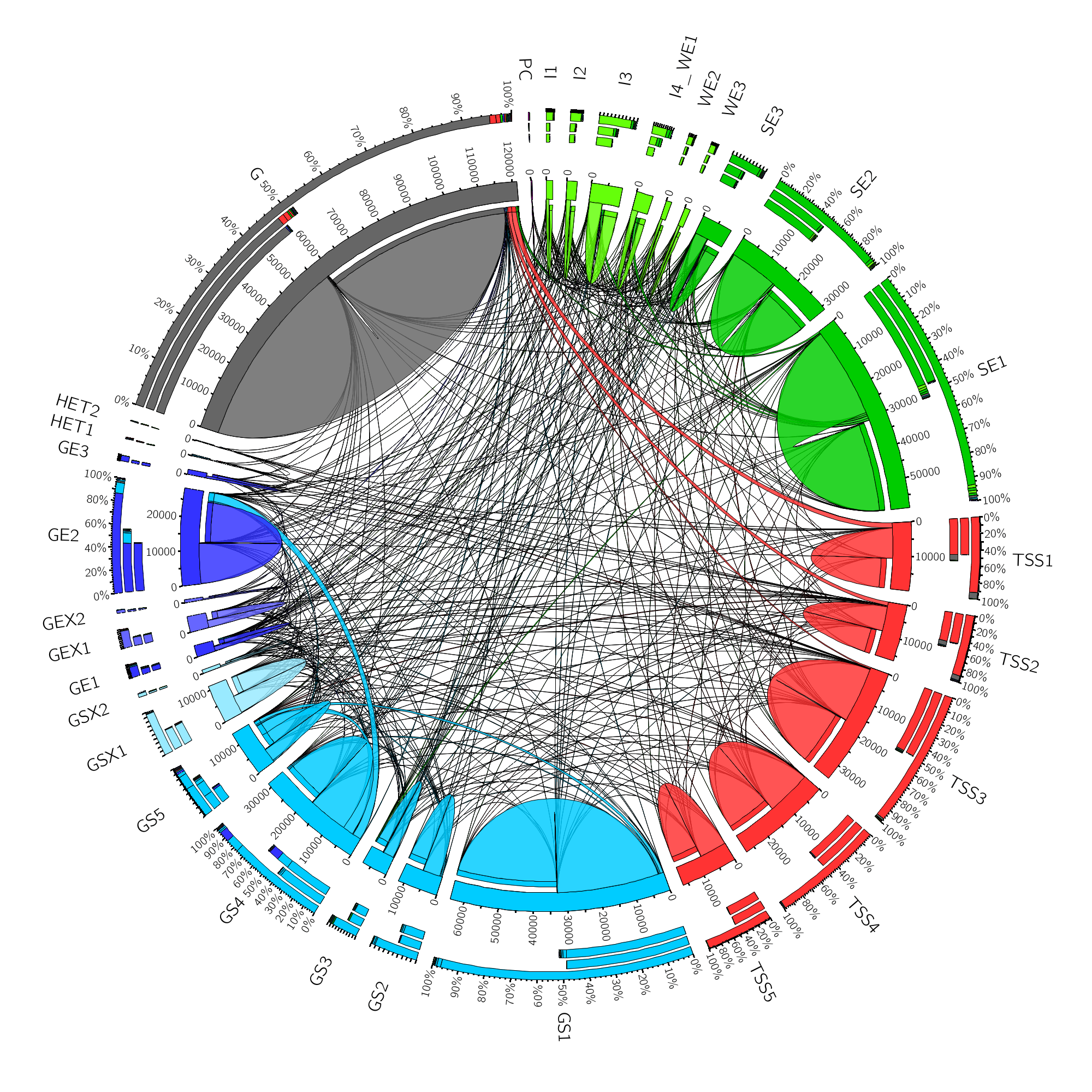

Supplement: Supplementary Data 4 — Effects of positive and negative perturbations of single chromatin factors on chromatin state identity. [file ncomms10528-s5.zip › Supplementary Data 4/NegativePerturbation/NURF301.png]

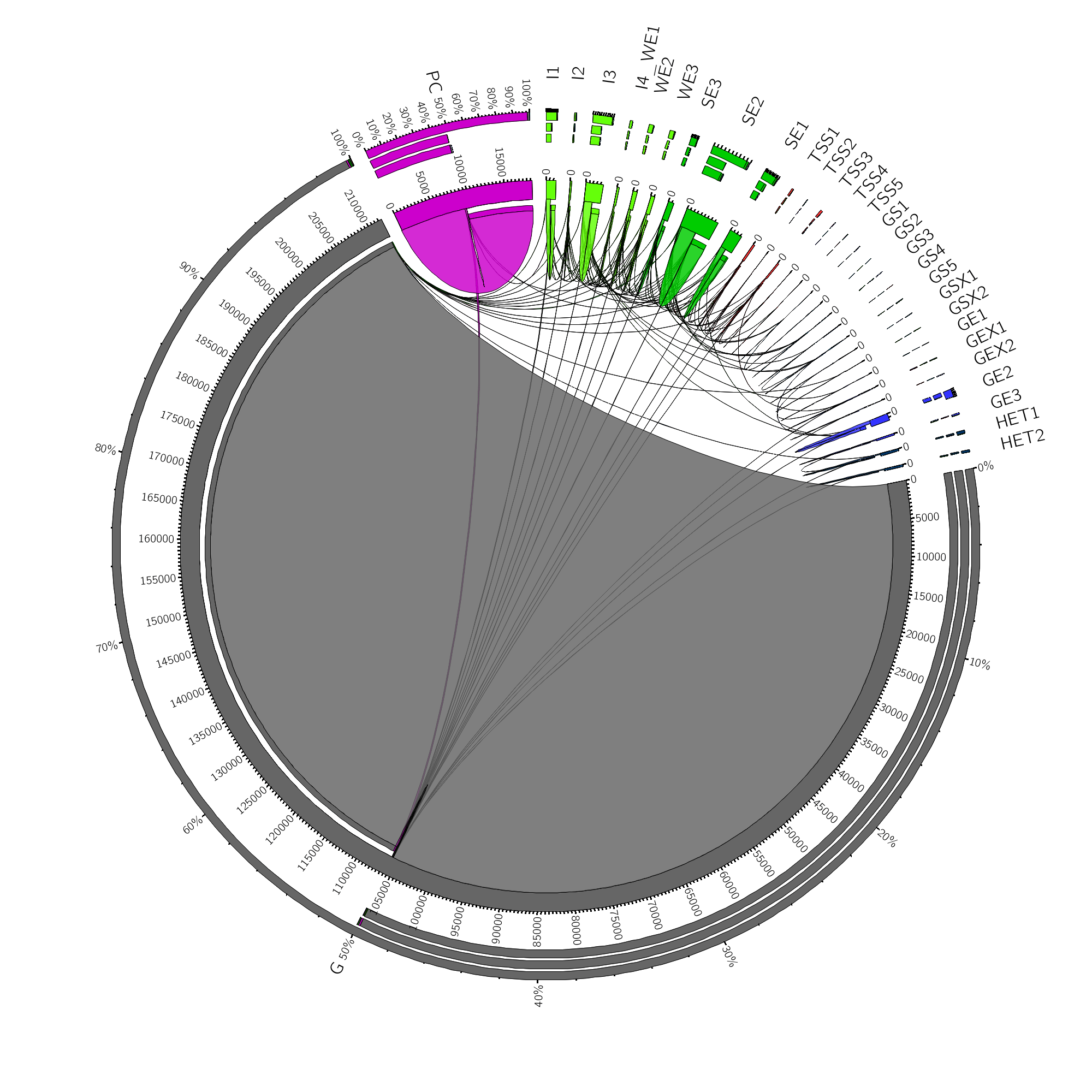

Supplement: Supplementary Data 4 — Effects of positive and negative perturbations of single chromatin factors on chromatin state identity. [file ncomms10528-s5.zip › Supplementary Data 4/NegativePerturbation/Pc.png]

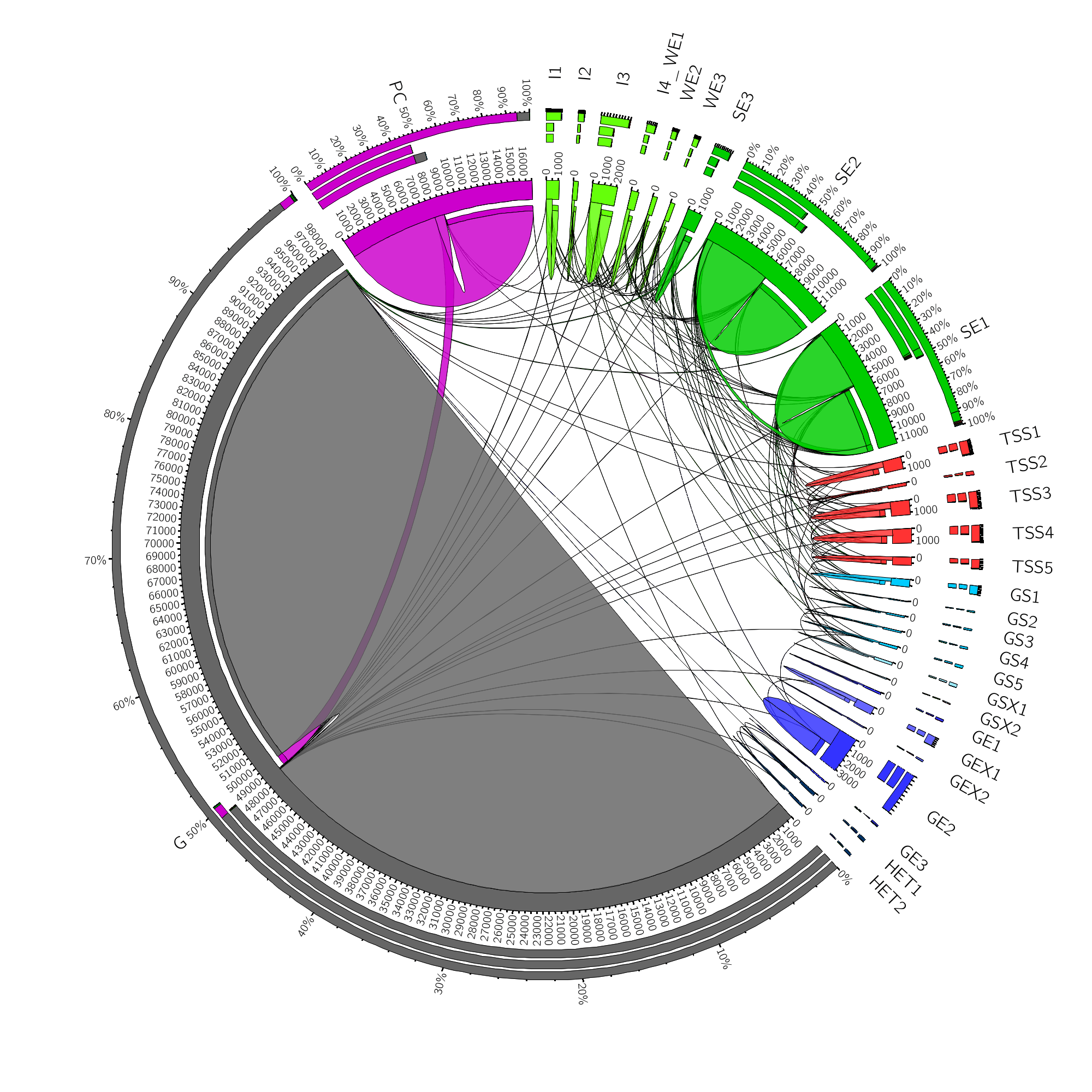

Supplement: Supplementary Data 4 — Effects of positive and negative perturbations of single chromatin factors on chromatin state identity. [file ncomms10528-s5.zip › Supplementary Data 4/NegativePerturbation/PCL.png]

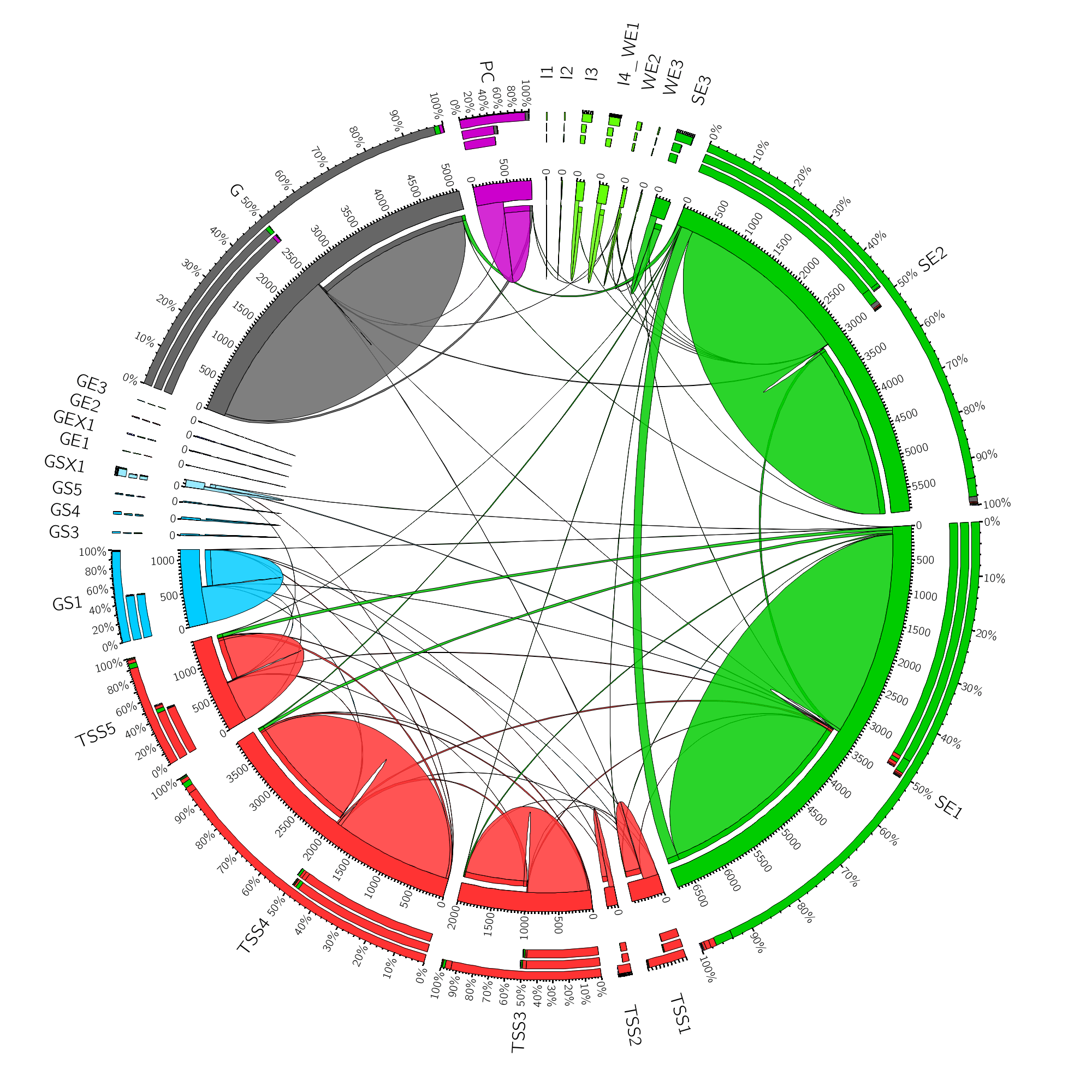

Supplement: Supplementary Data 4 — Effects of positive and negative perturbations of single chromatin factors on chromatin state identity. [file ncomms10528-s5.zip › Supplementary Data 4/NegativePerturbation/Pho.png]

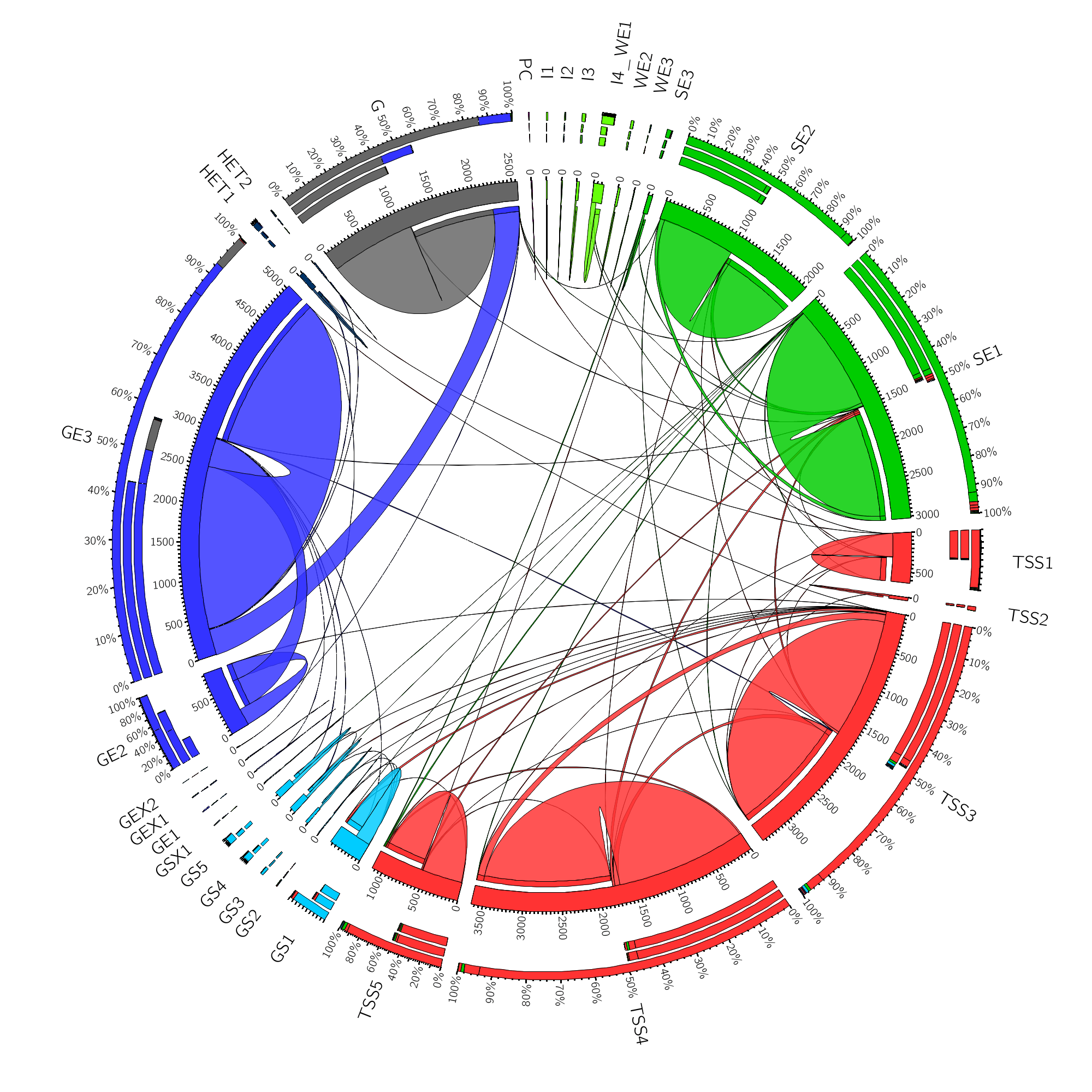

Supplement: Supplementary Data 4 — Effects of positive and negative perturbations of single chromatin factors on chromatin state identity. [file ncomms10528-s5.zip › Supplementary Data 4/NegativePerturbation/POF.png]

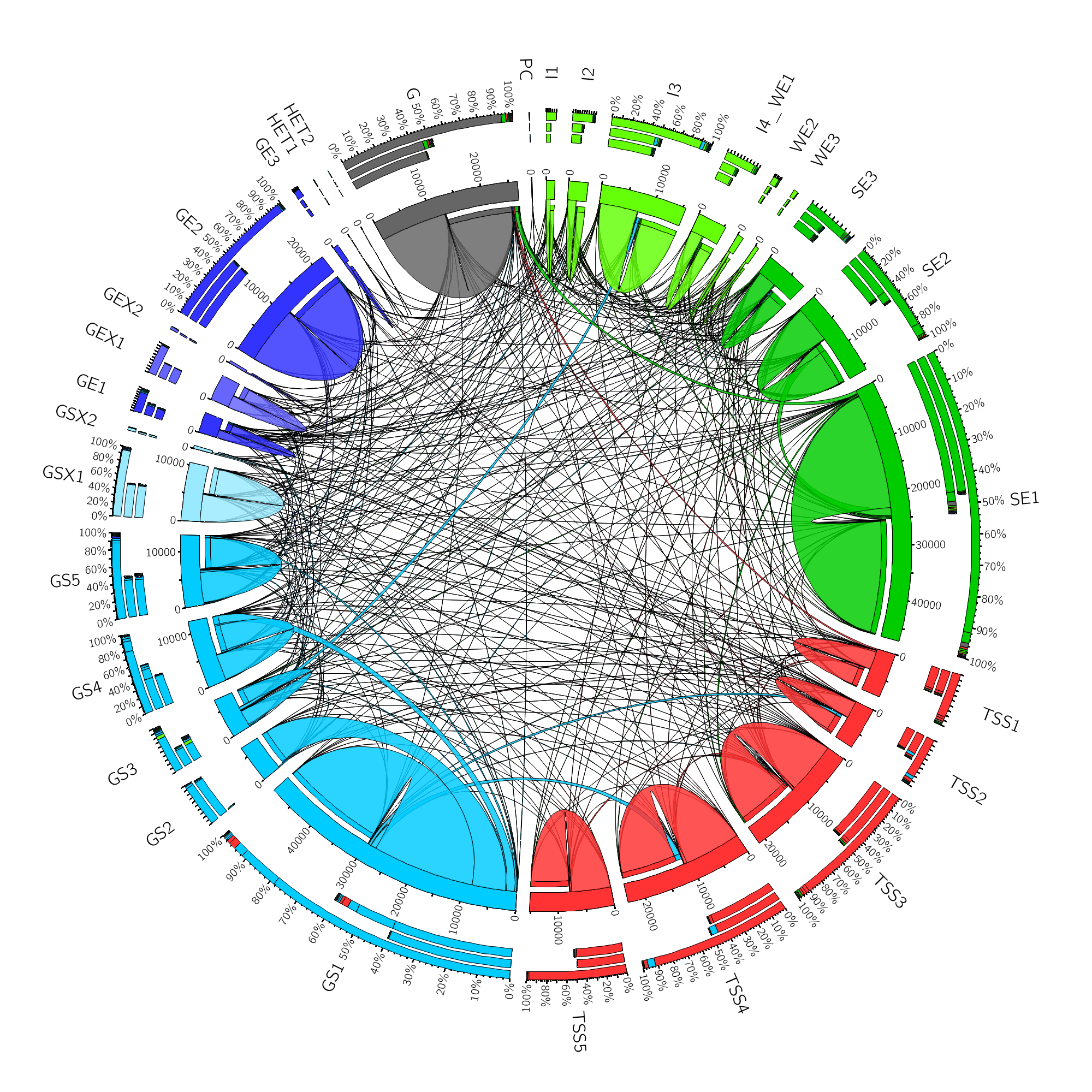

Supplement: Supplementary Data 4 — Effects of positive and negative perturbations of single chromatin factors on chromatin state identity. [file ncomms10528-s5.zip › Supplementary Data 4/NegativePerturbation/pol2.png]

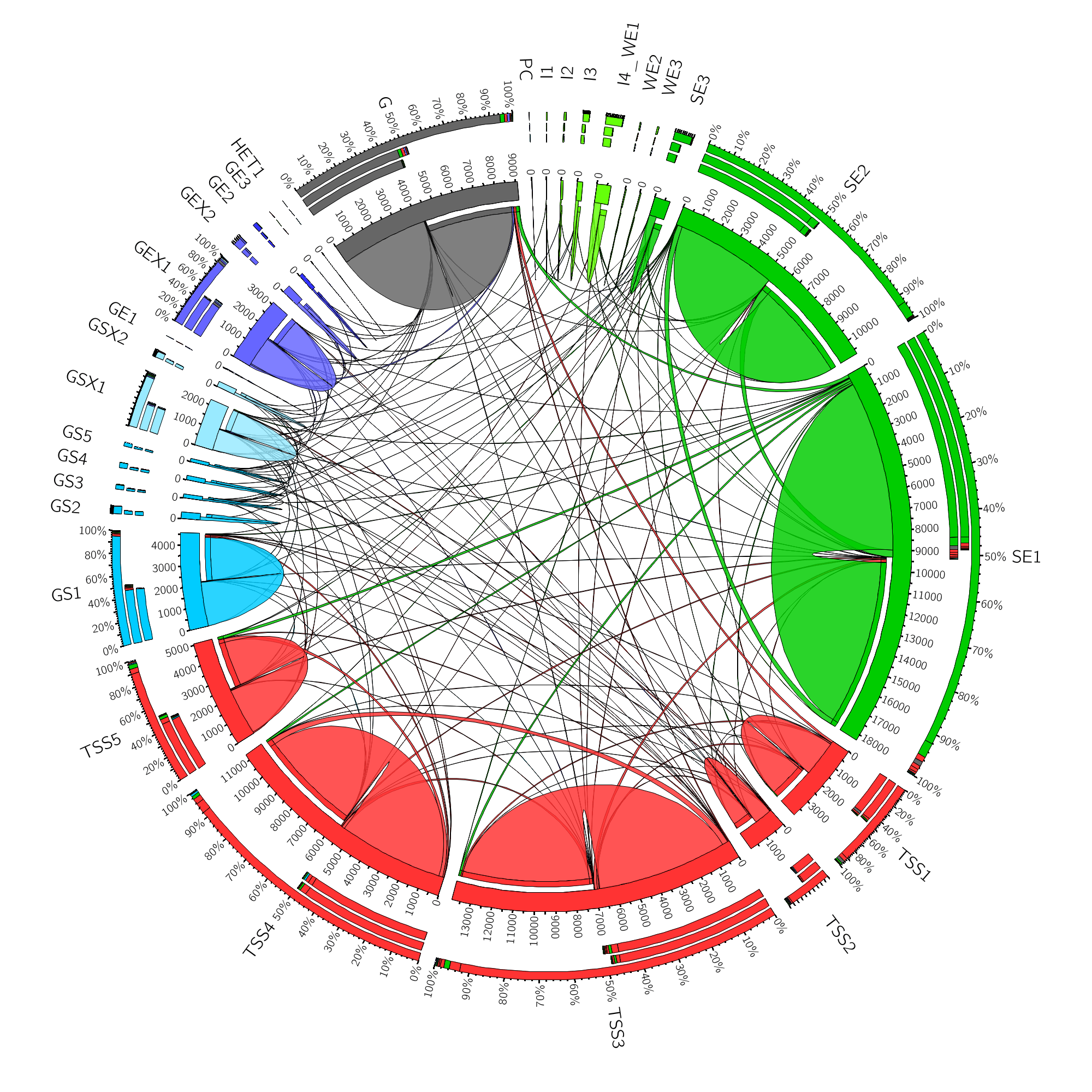

Supplement: Supplementary Data 4 — Effects of positive and negative perturbations of single chromatin factors on chromatin state identity. [file ncomms10528-s5.zip › Supplementary Data 4/NegativePerturbation/PRSet7.png]

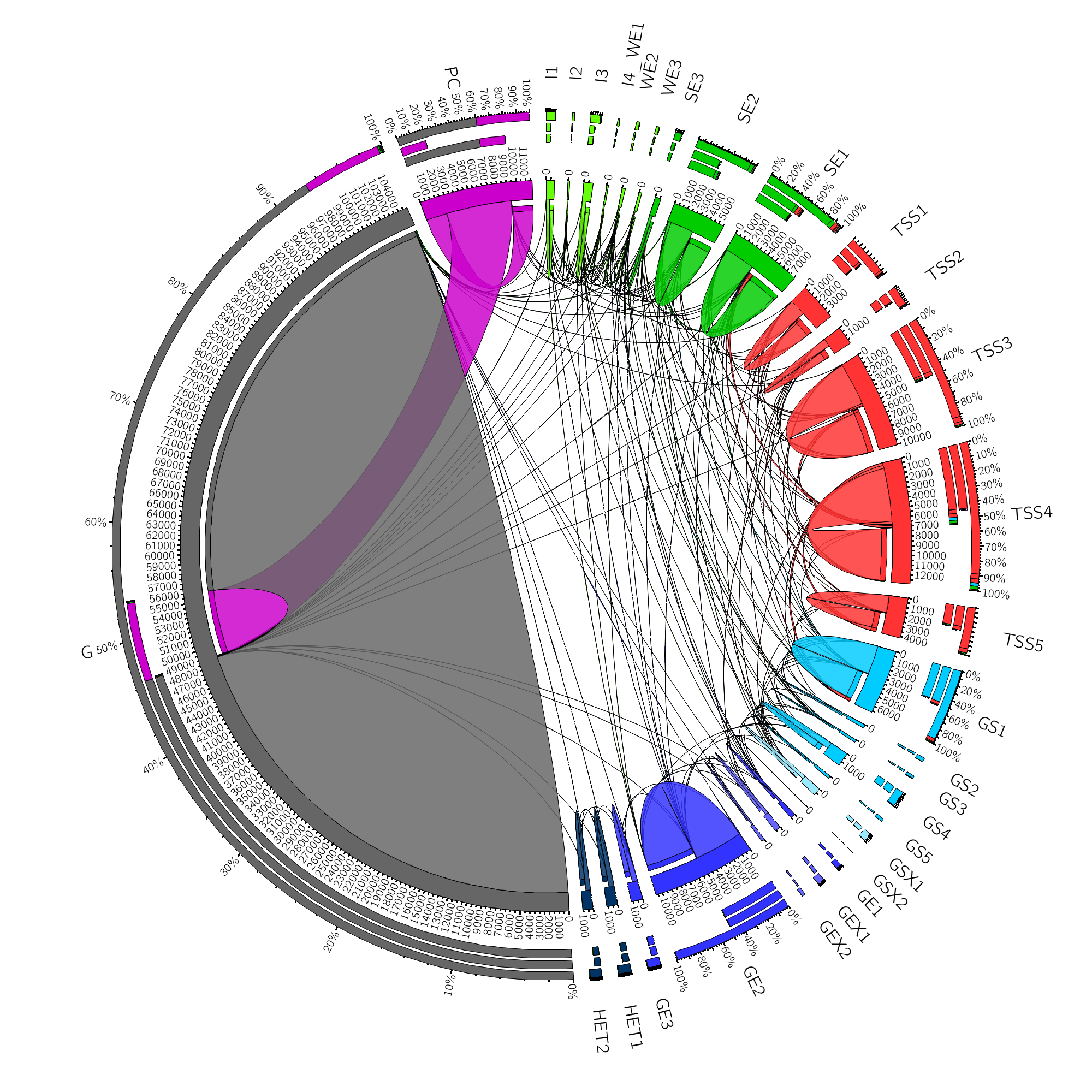

Supplement: Supplementary Data 4 — Effects of positive and negative perturbations of single chromatin factors on chromatin state identity. [file ncomms10528-s5.zip › Supplementary Data 4/NegativePerturbation/Psc.png]

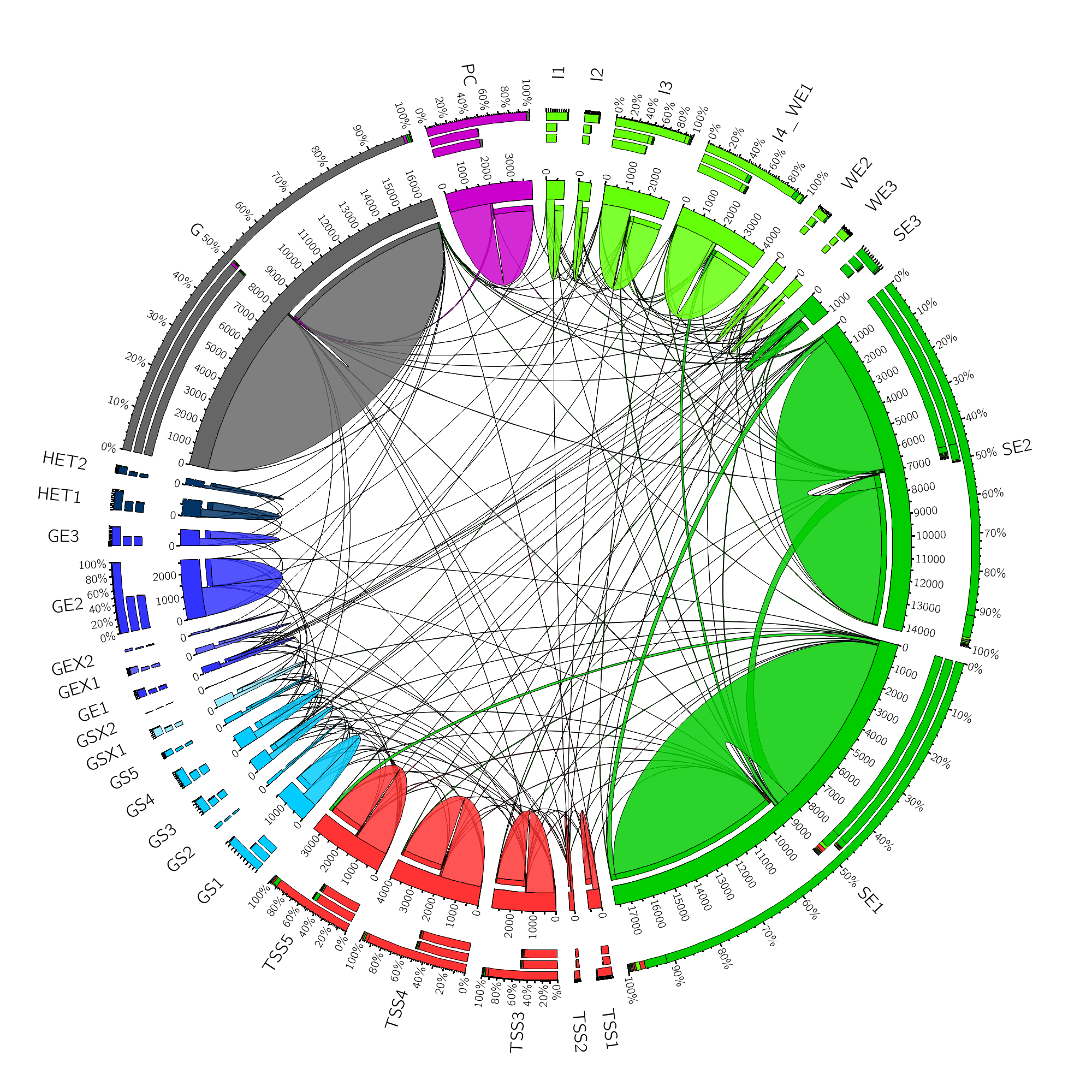

Supplement: Supplementary Data 4 — Effects of positive and negative perturbations of single chromatin factors on chromatin state identity. [file ncomms10528-s5.zip › Supplementary Data 4/NegativePerturbation/Rhino.png]

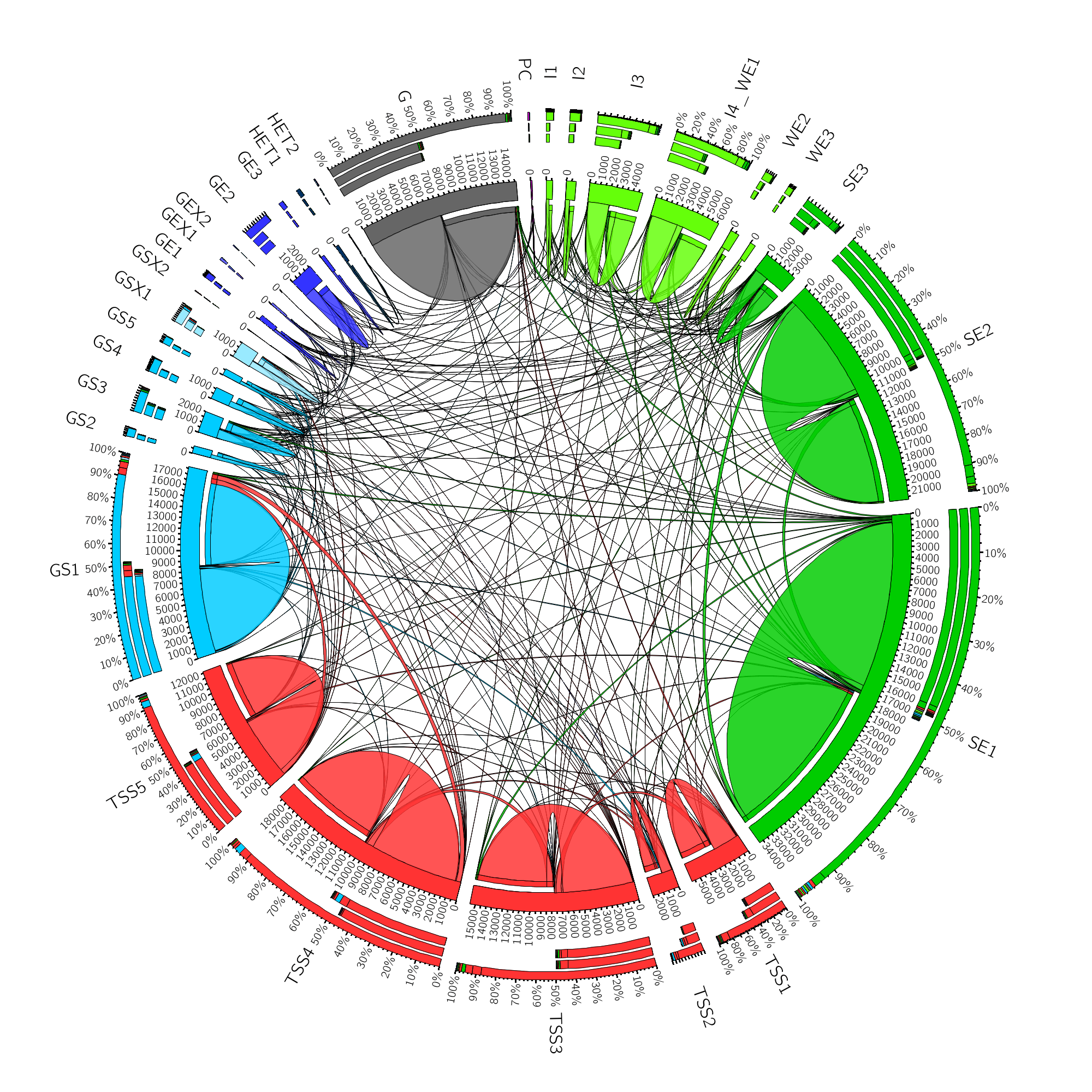

Supplement: Supplementary Data 4 — Effects of positive and negative perturbations of single chromatin factors on chromatin state identity. [file ncomms10528-s5.zip › Supplementary Data 4/NegativePerturbation/RPD3.png]

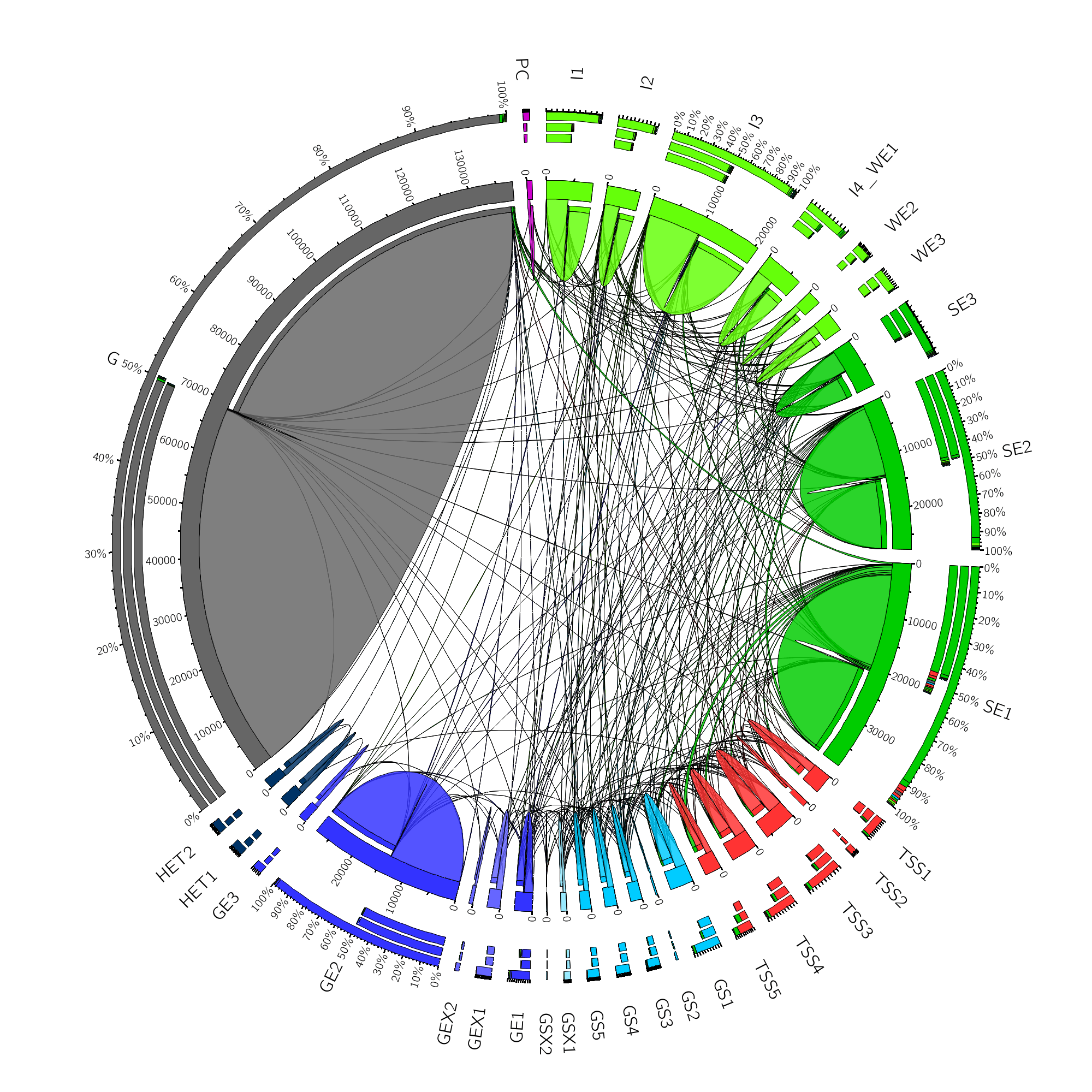

Supplement: Supplementary Data 4 — Effects of positive and negative perturbations of single chromatin factors on chromatin state identity. [file ncomms10528-s5.zip › Supplementary Data 4/NegativePerturbation/Smc3.png]

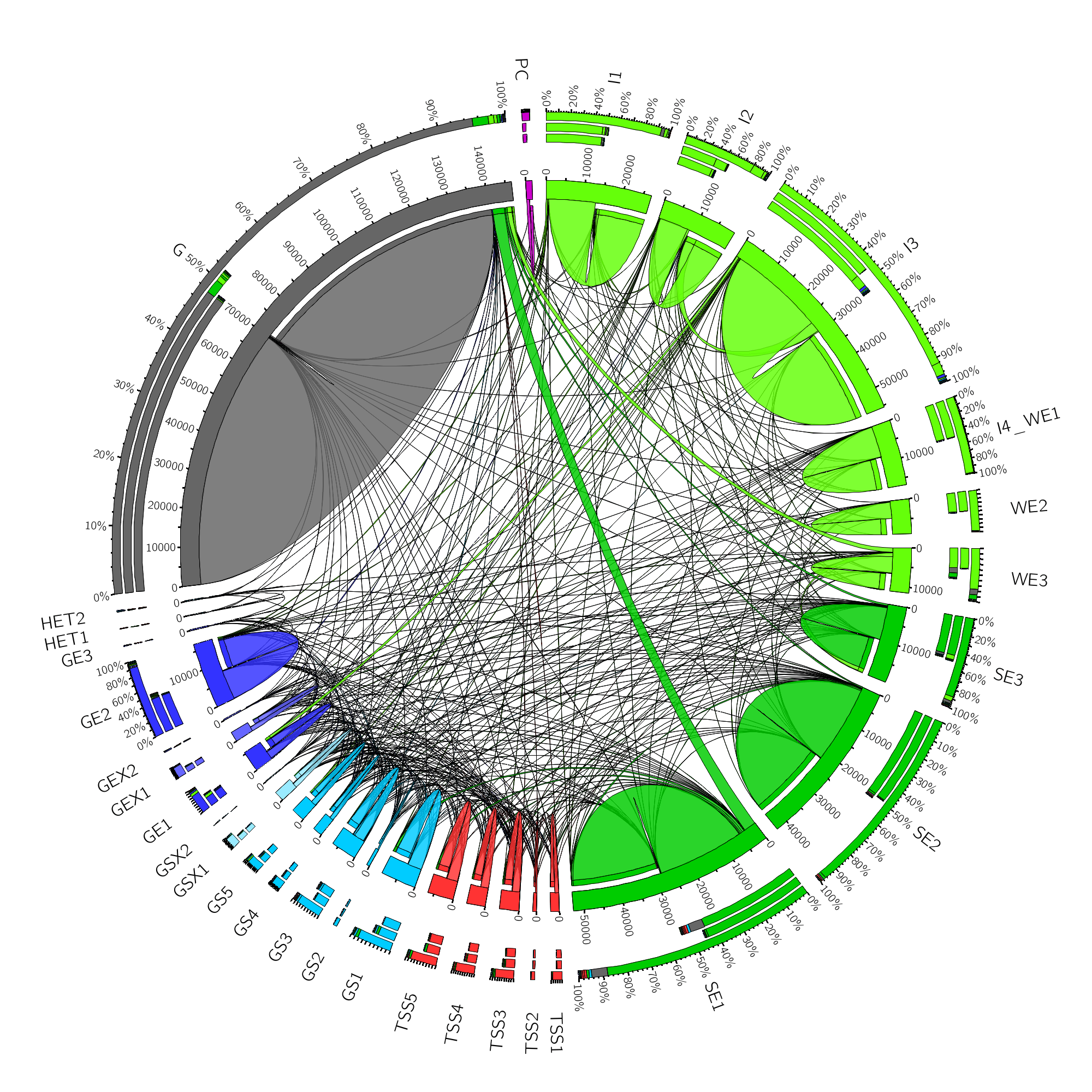

Supplement: Supplementary Data 4 — Effects of positive and negative perturbations of single chromatin factors on chromatin state identity. [file ncomms10528-s5.zip › Supplementary Data 4/NegativePerturbation/SPT16.png]

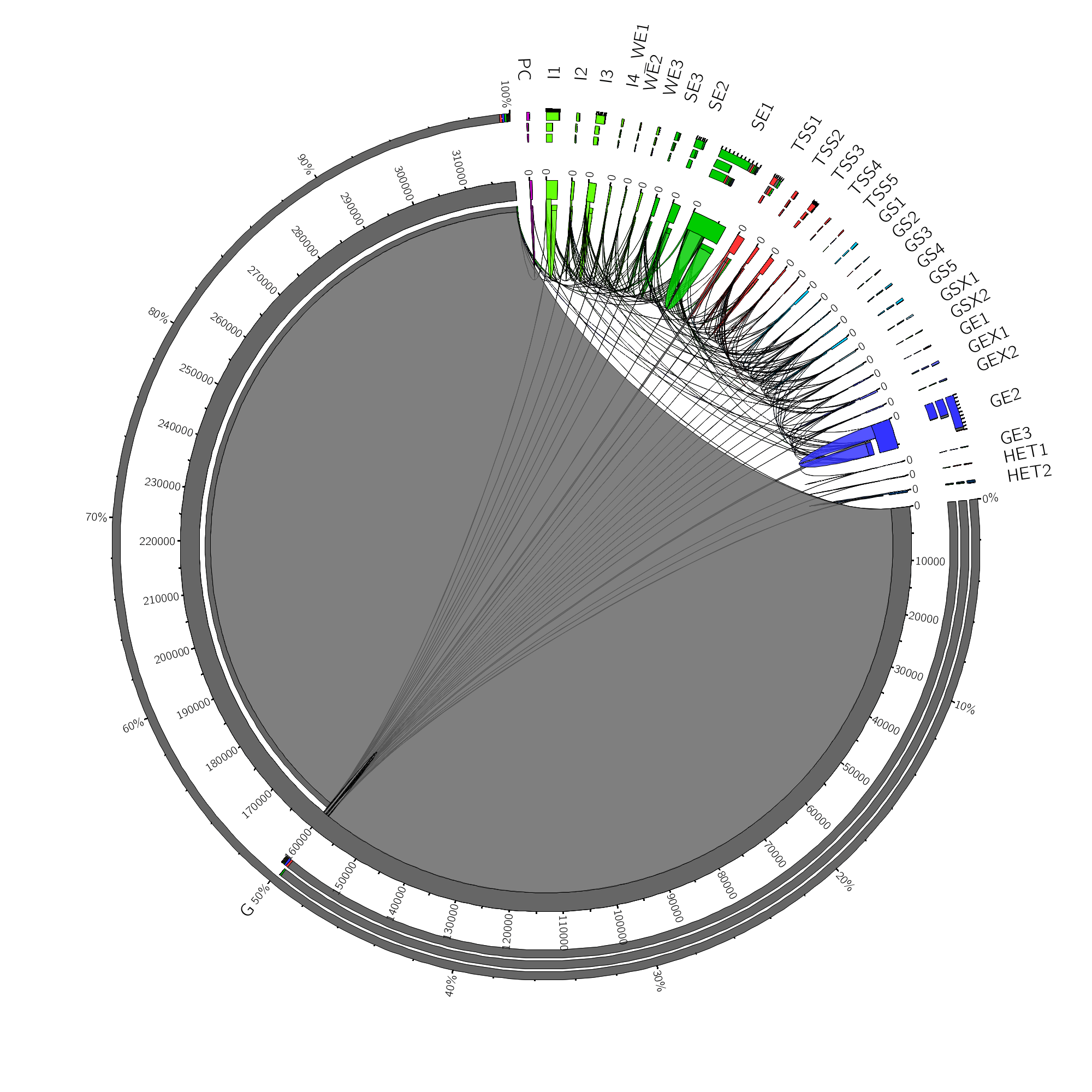

Supplement: Supplementary Data 4 — Effects of positive and negative perturbations of single chromatin factors on chromatin state identity. [file ncomms10528-s5.zip › Supplementary Data 4/NegativePerturbation/SuHw.png]

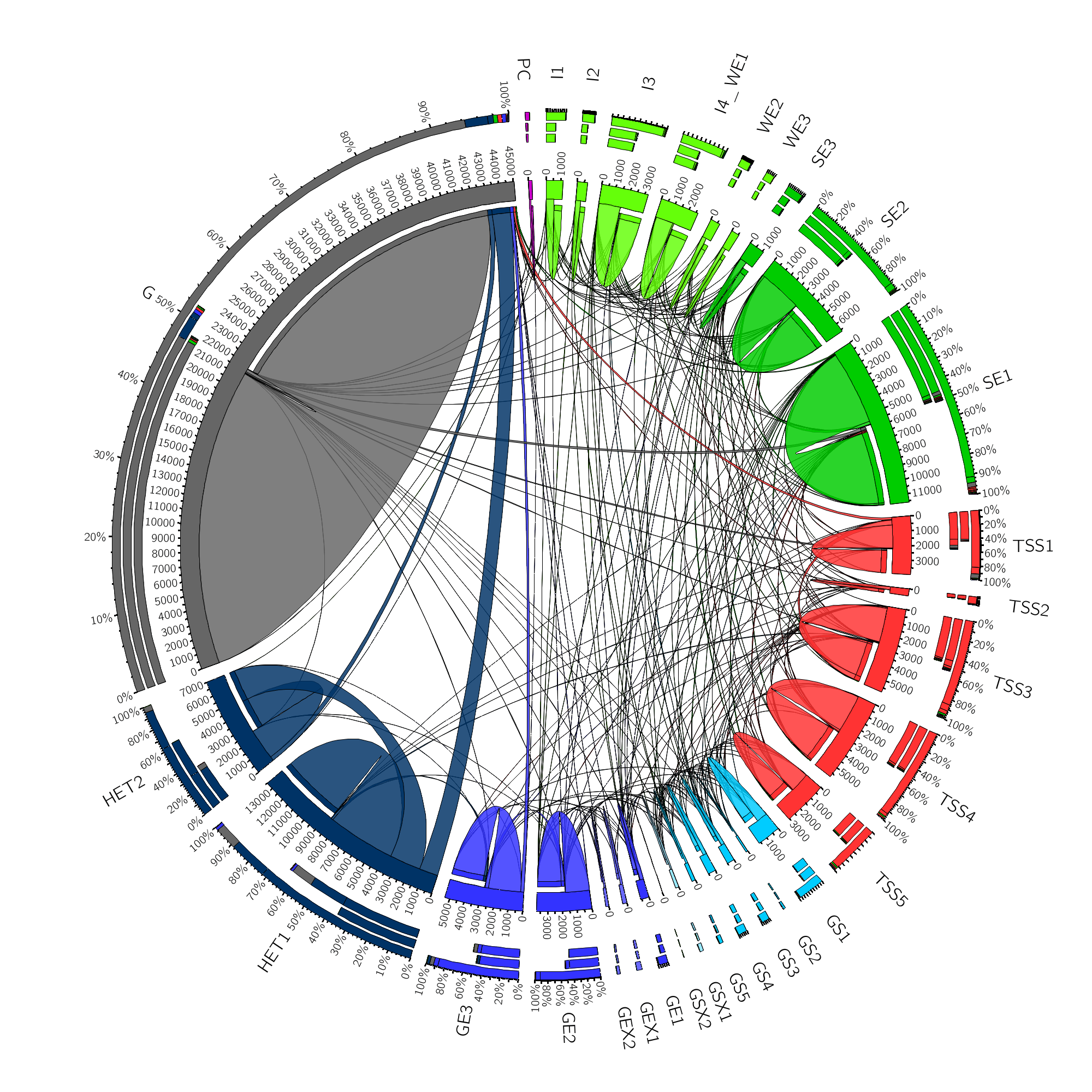

Supplement: Supplementary Data 4 — Effects of positive and negative perturbations of single chromatin factors on chromatin state identity. [file ncomms10528-s5.zip › Supplementary Data 4/NegativePerturbation/Suvar37.png]

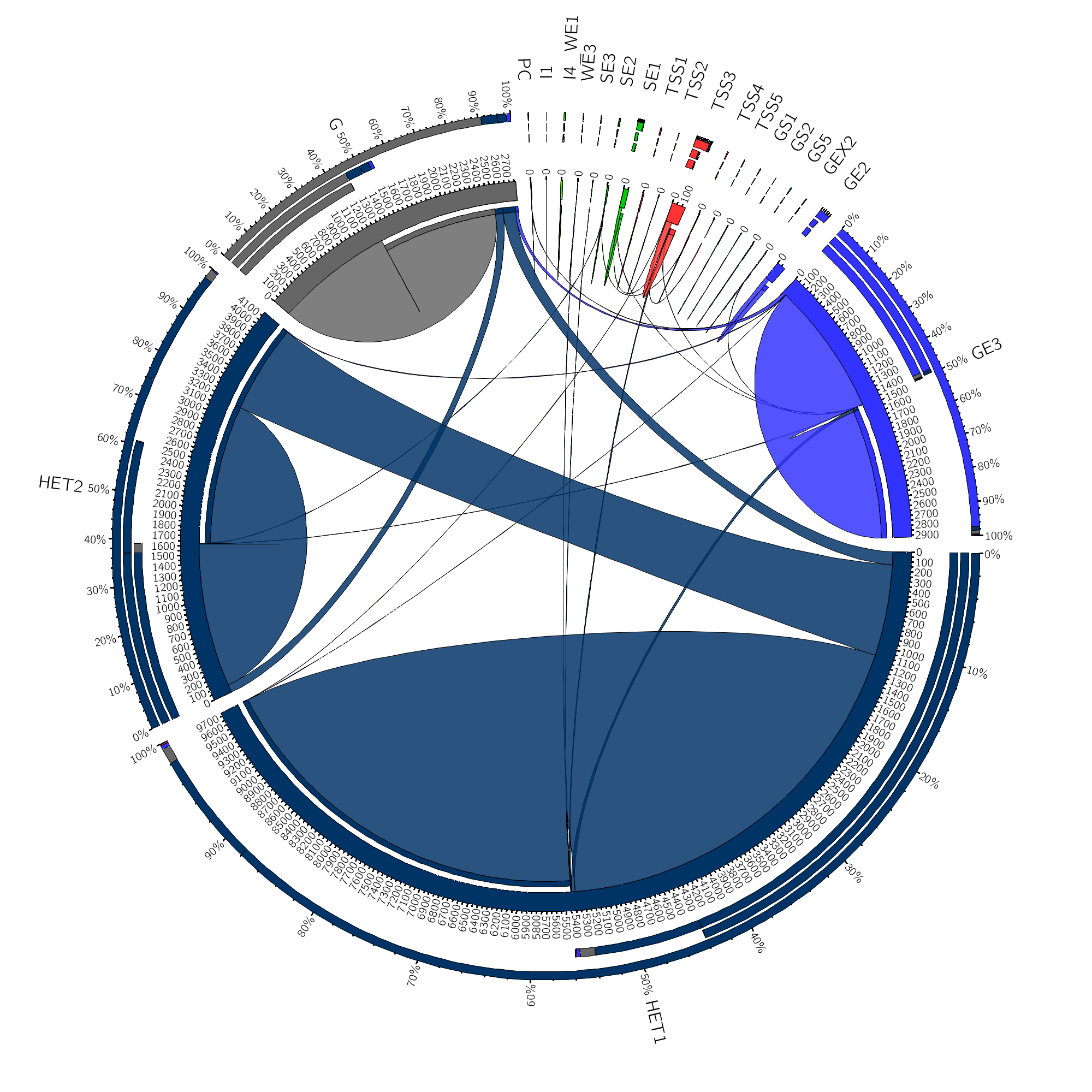

Supplement: Supplementary Data 4 — Effects of positive and negative perturbations of single chromatin factors on chromatin state identity. [file ncomms10528-s5.zip › Supplementary Data 4/NegativePerturbation/Suvar39.png]

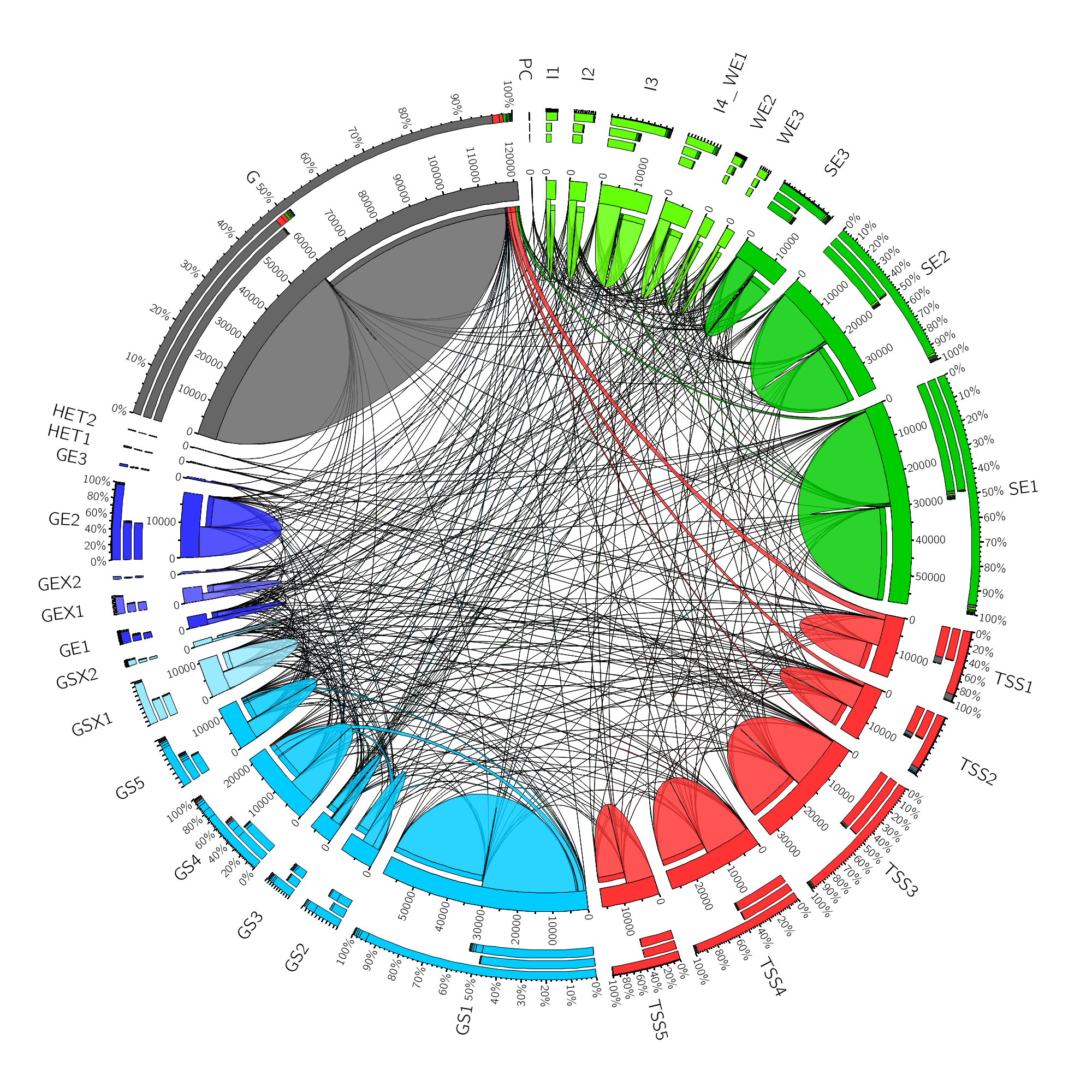

Supplement: Supplementary Data 4 — Effects of positive and negative perturbations of single chromatin factors on chromatin state identity. [file ncomms10528-s5.zip › Supplementary Data 4/NegativePerturbation/WDS.png]

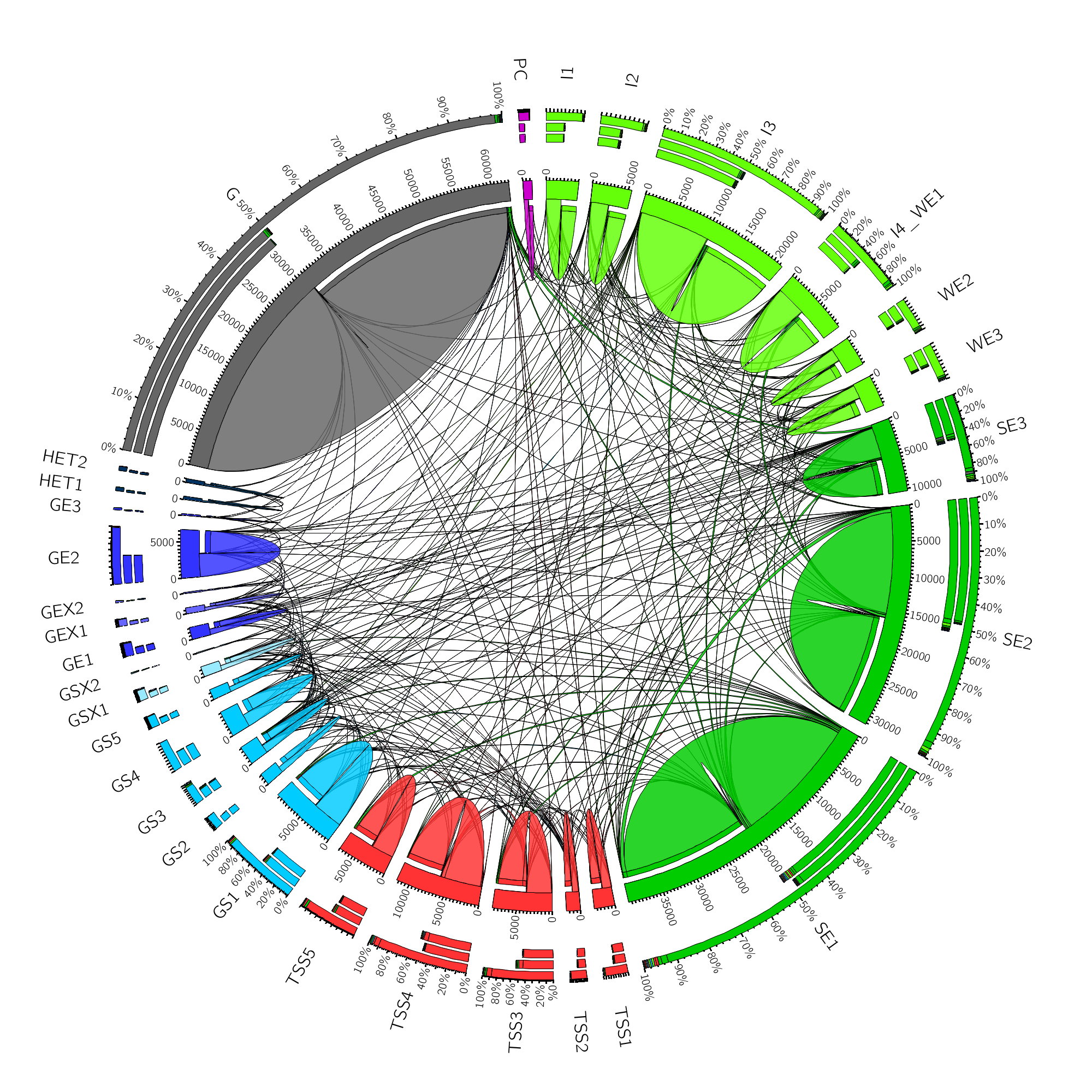

Supplement: Supplementary Data 4 — Effects of positive and negative perturbations of single chromatin factors on chromatin state identity. [file ncomms10528-s5.zip › Supplementary Data 4/NegativePerturbation/ZW5.png]

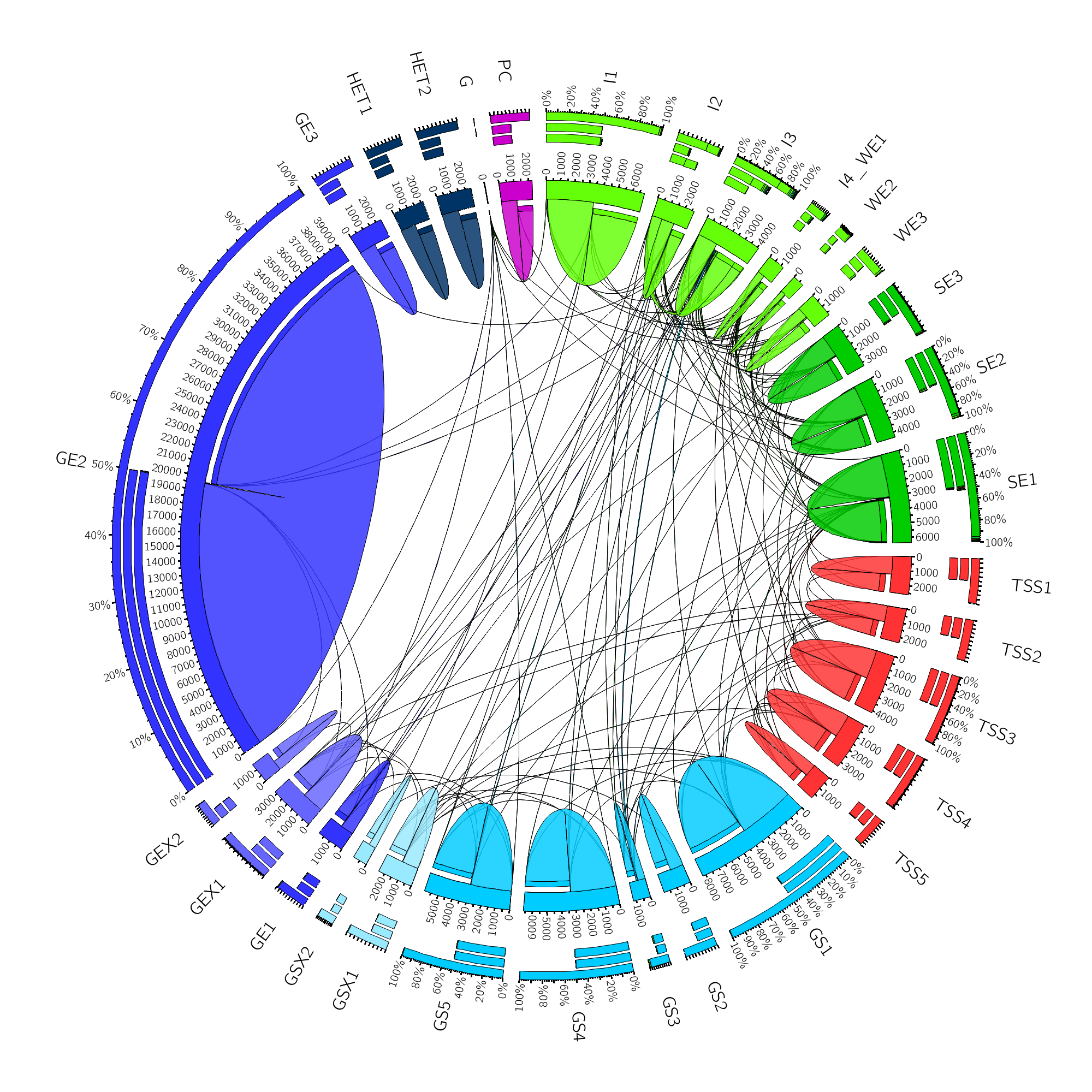

Supplement: Supplementary Data 4 — Effects of positive and negative perturbations of single chromatin factors on chromatin state identity. [file ncomms10528-s5.zip › Supplementary Data 4/PositivePerturbation/ASH1.png]

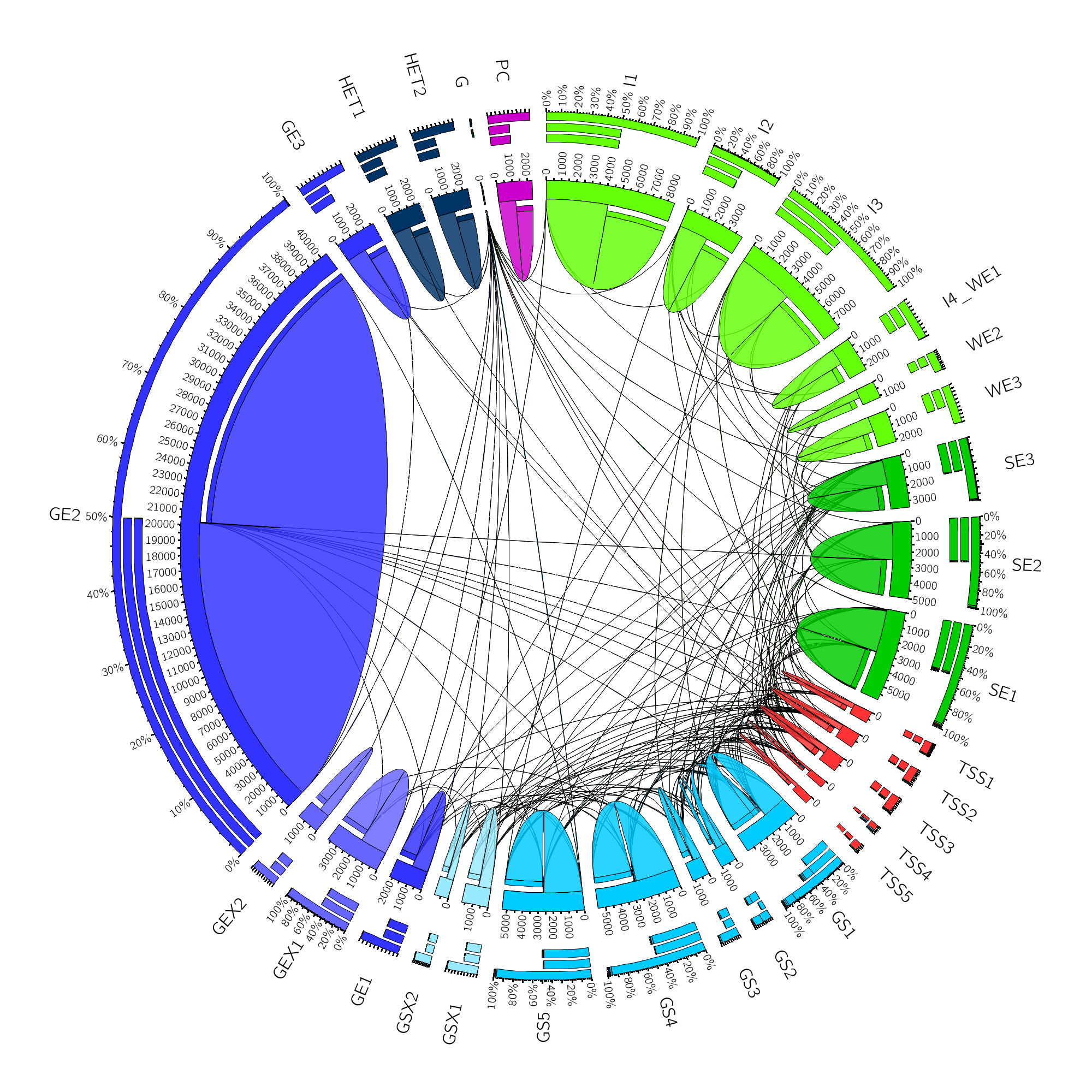

Supplement: Supplementary Data 4 — Effects of positive and negative perturbations of single chromatin factors on chromatin state identity. [file ncomms10528-s5.zip › Supplementary Data 4/PositivePerturbation/BEAF32.png]

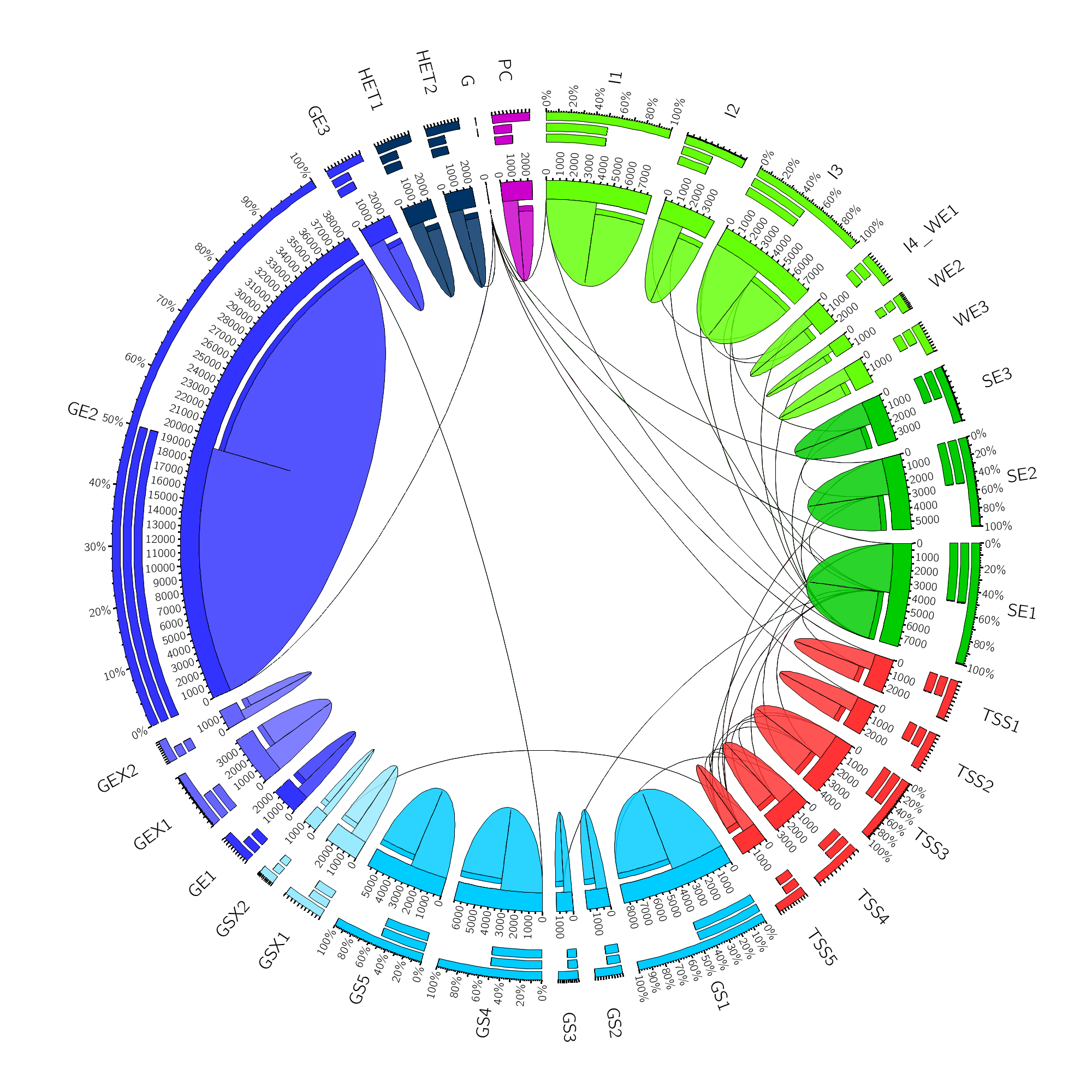

Supplement: Supplementary Data 4 — Effects of positive and negative perturbations of single chromatin factors on chromatin state identity. [file ncomms10528-s5.zip › Supplementary Data 4/PositivePerturbation/CG10630.png]

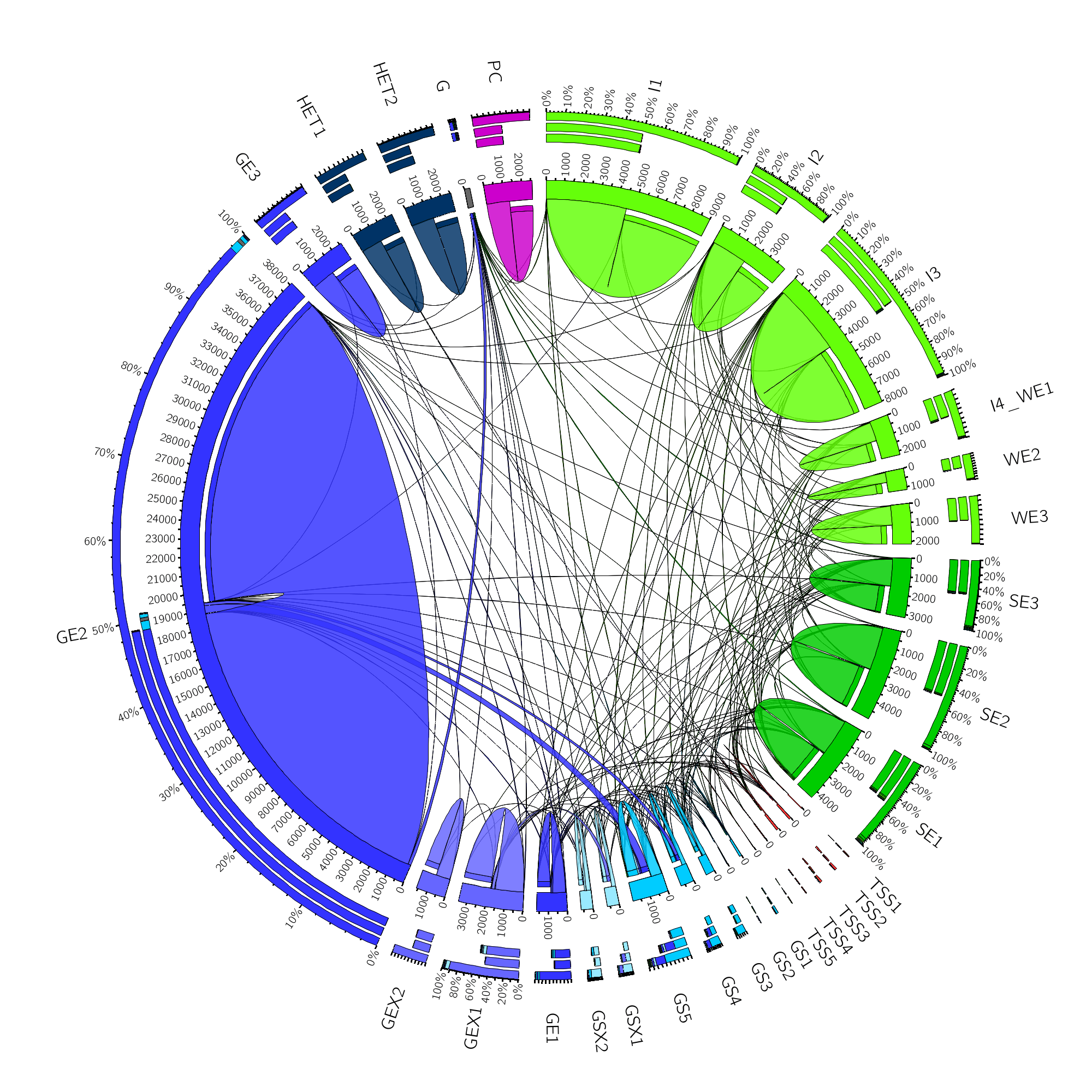

Supplement: Supplementary Data 4 — Effects of positive and negative perturbations of single chromatin factors on chromatin state identity. [file ncomms10528-s5.zip › Supplementary Data 4/PositivePerturbation/Chriz.png]

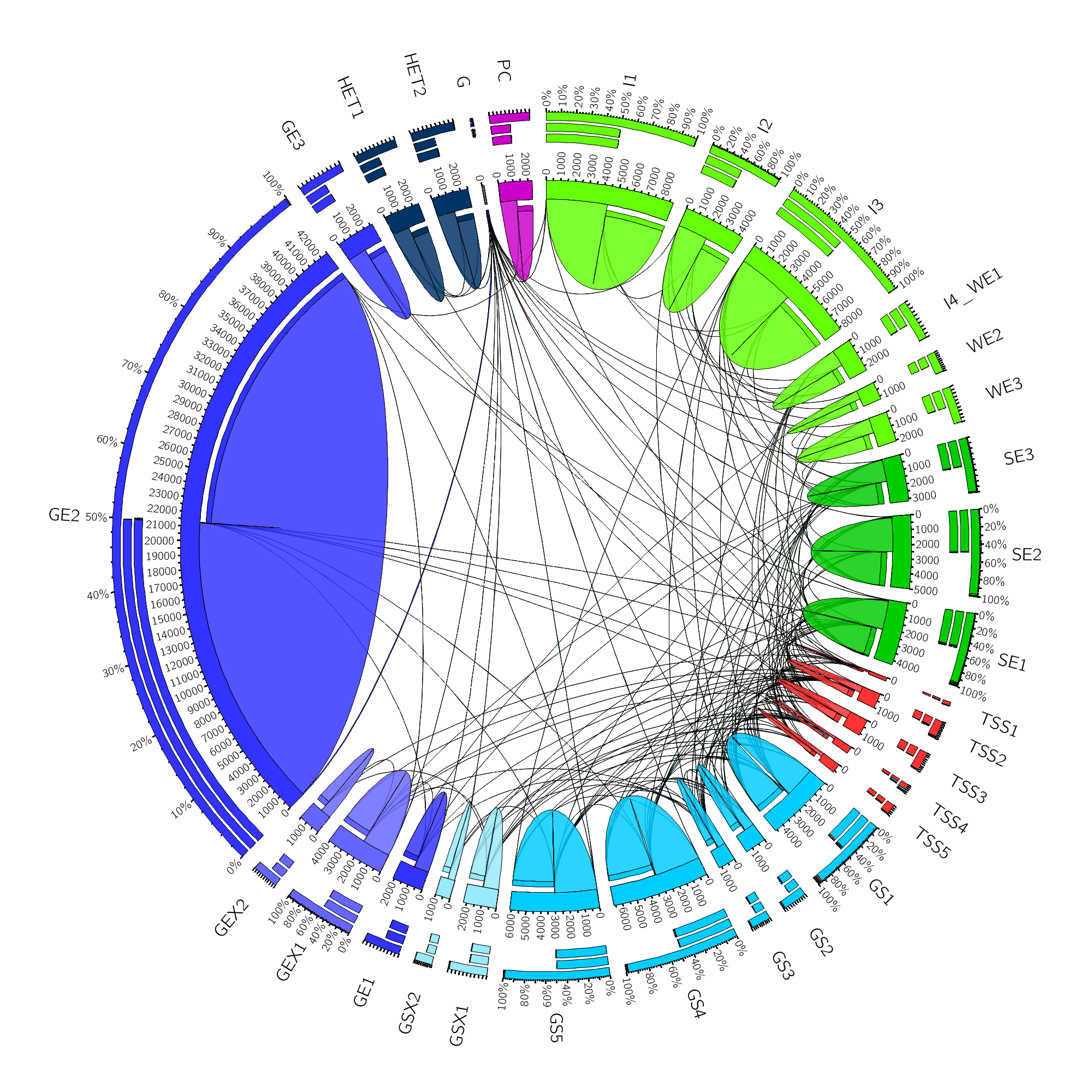

Supplement: Supplementary Data 4 — Effects of positive and negative perturbations of single chromatin factors on chromatin state identity. [file ncomms10528-s5.zip › Supplementary Data 4/PositivePerturbation/CP190.png]

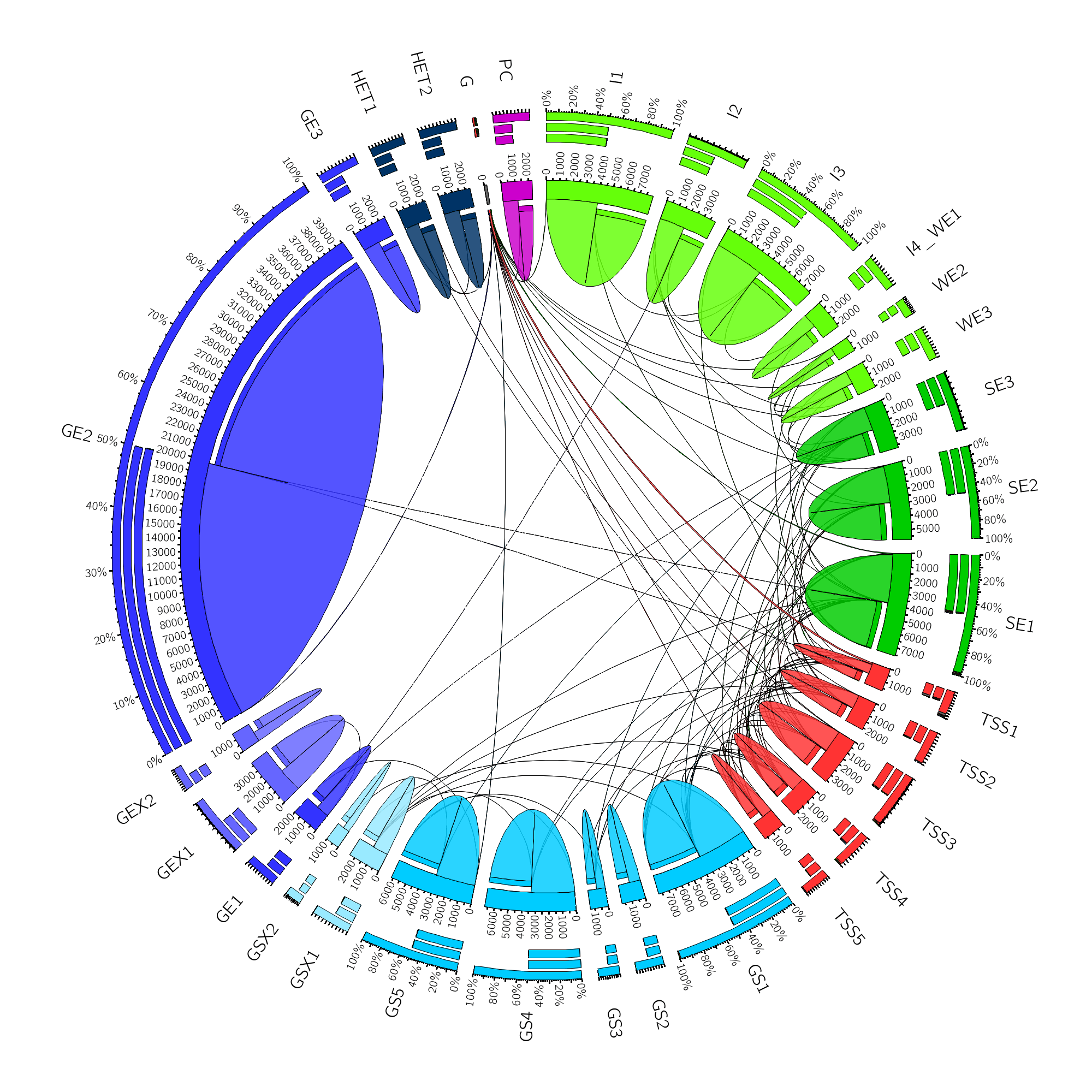

Supplement: Supplementary Data 4 — Effects of positive and negative perturbations of single chromatin factors on chromatin state identity. [file ncomms10528-s5.zip › Supplementary Data 4/PositivePerturbation/CTCF.png]

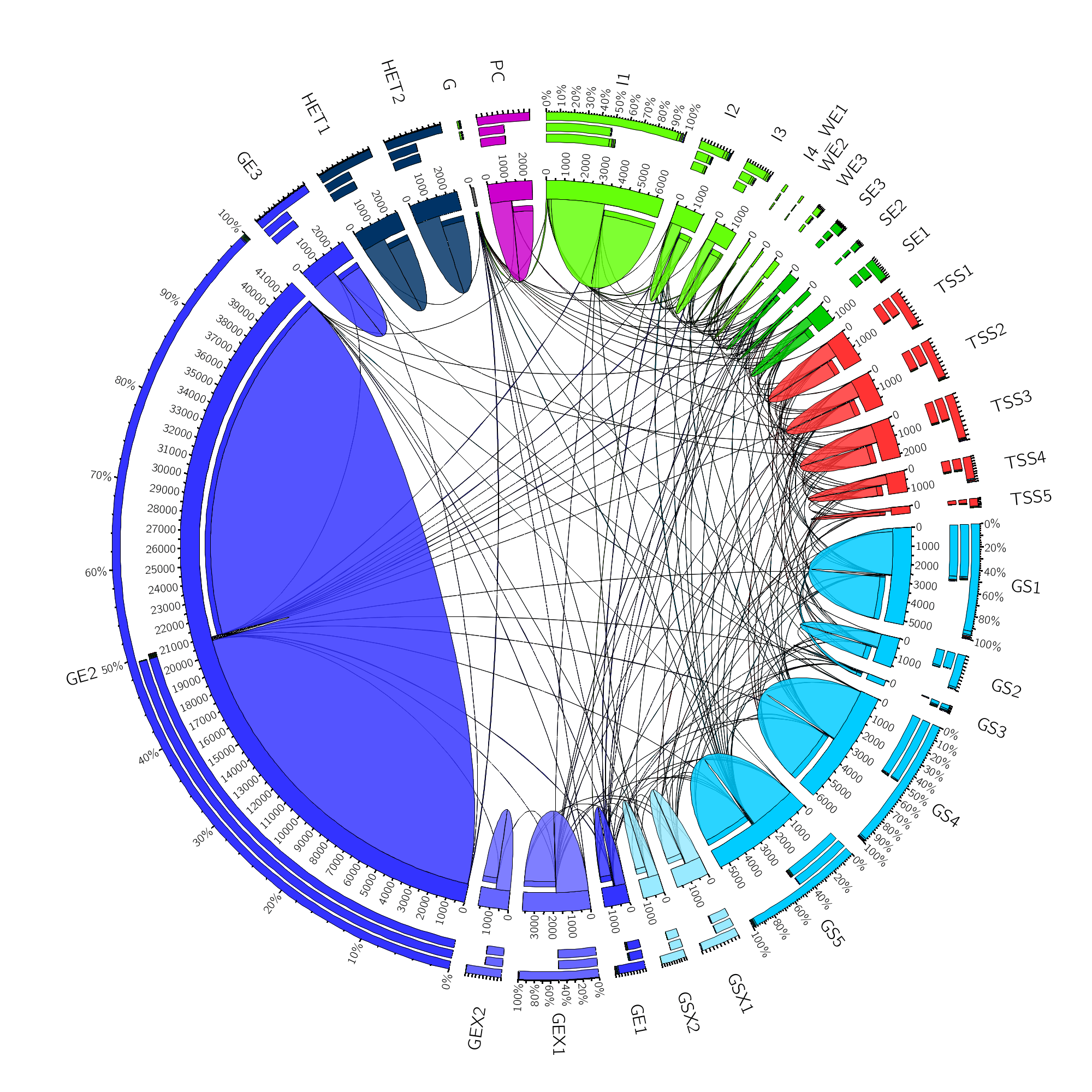

Supplement: Supplementary Data 4 — Effects of positive and negative perturbations of single chromatin factors on chromatin state identity. [file ncomms10528-s5.zip › Supplementary Data 4/PositivePerturbation/dMi2.png]

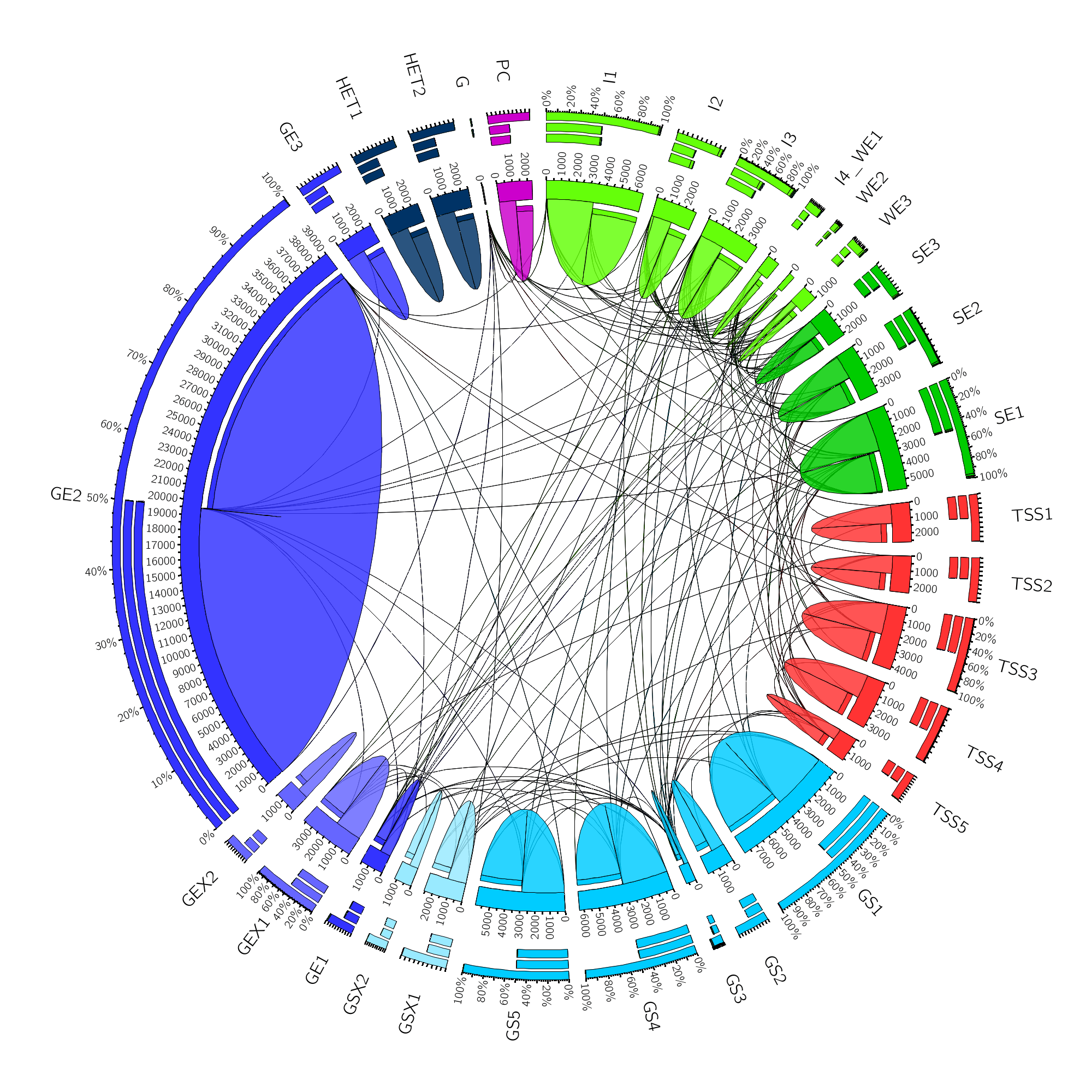

Supplement: Supplementary Data 4 — Effects of positive and negative perturbations of single chromatin factors on chromatin state identity. [file ncomms10528-s5.zip › Supplementary Data 4/PositivePerturbation/dmTopoII.png]

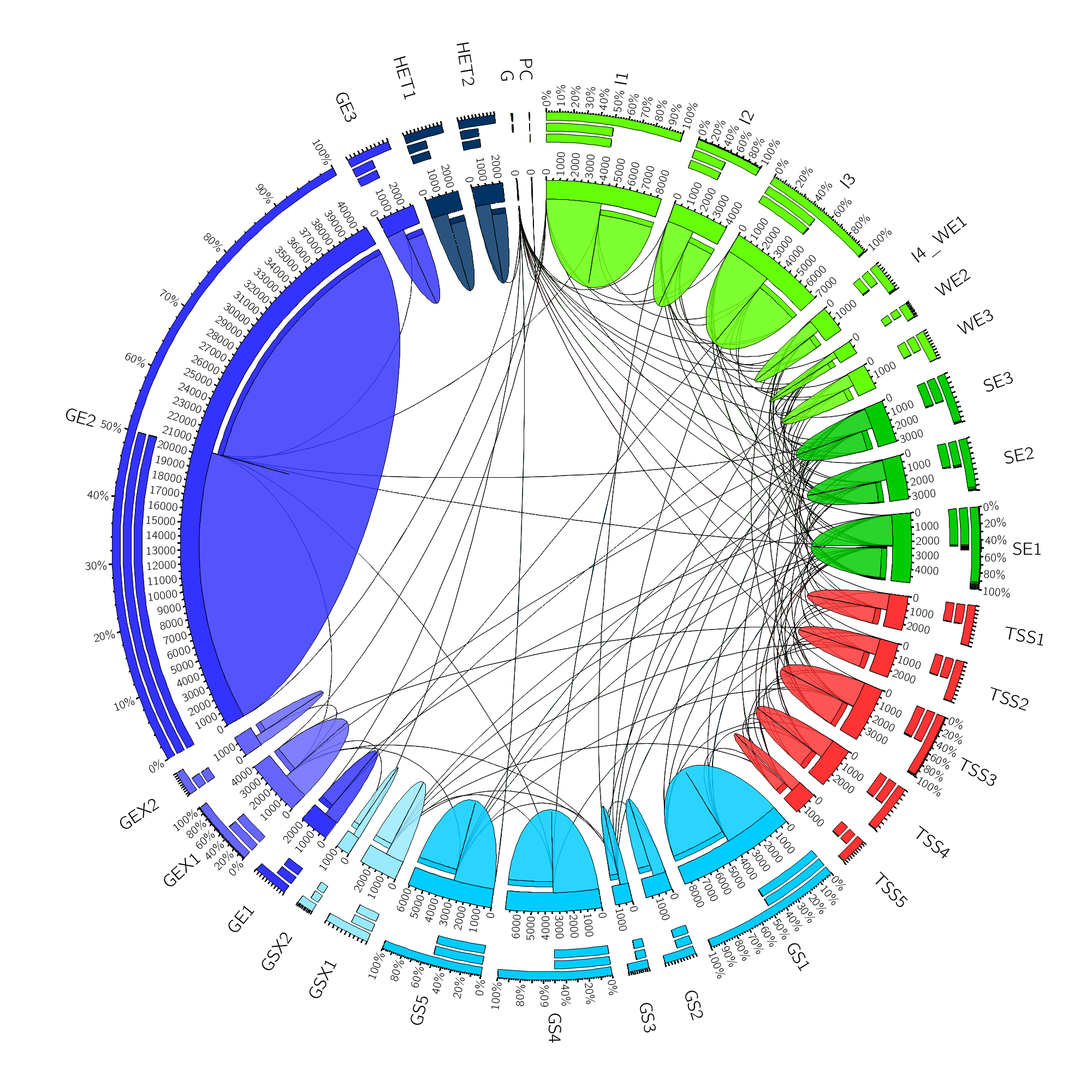

Supplement: Supplementary Data 4 — Effects of positive and negative perturbations of single chromatin factors on chromatin state identity. [file ncomms10528-s5.zip › Supplementary Data 4/PositivePerturbation/dRING.png]

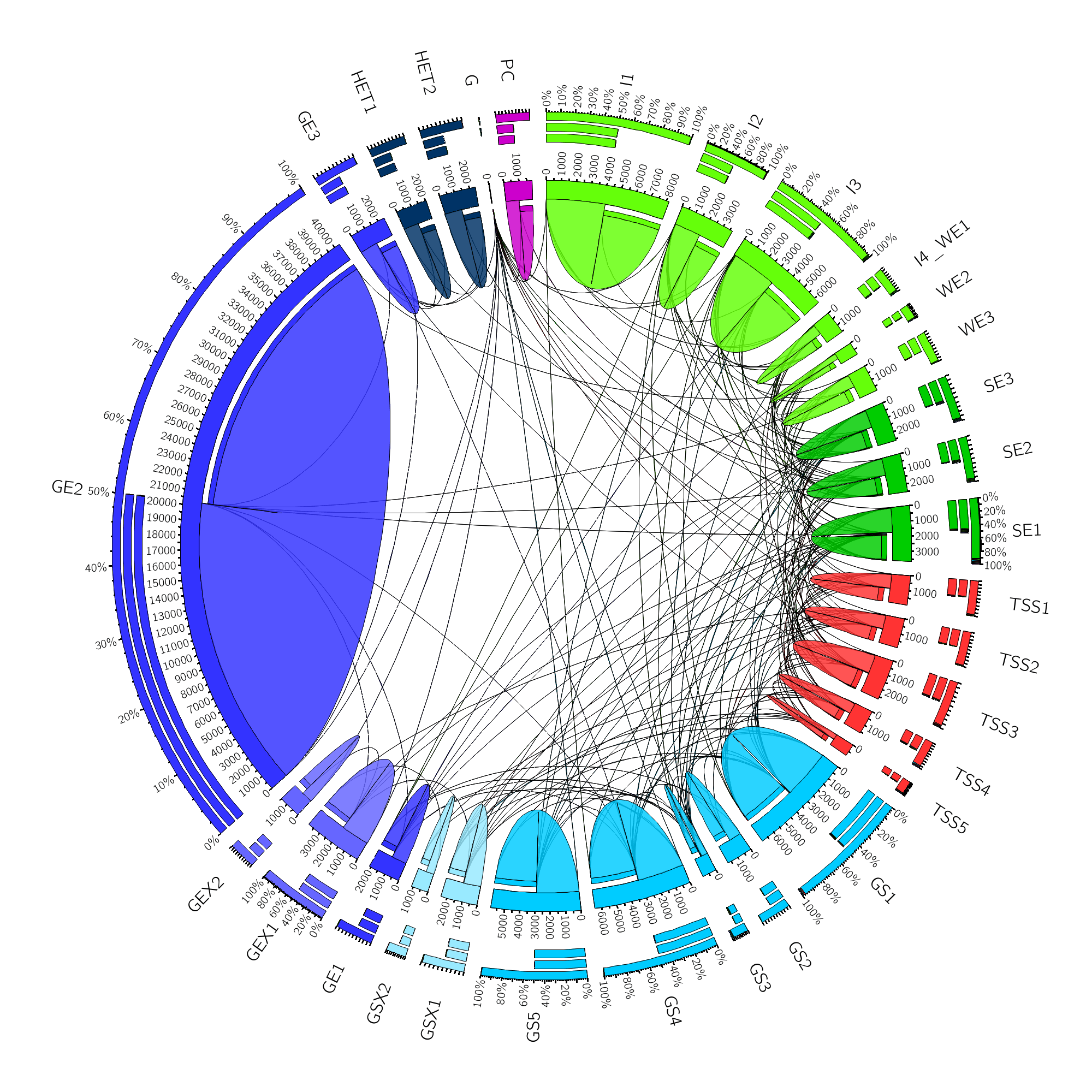

Supplement: Supplementary Data 4 — Effects of positive and negative perturbations of single chromatin factors on chromatin state identity. [file ncomms10528-s5.zip › Supplementary Data 4/PositivePerturbation/dSFMBT.png]

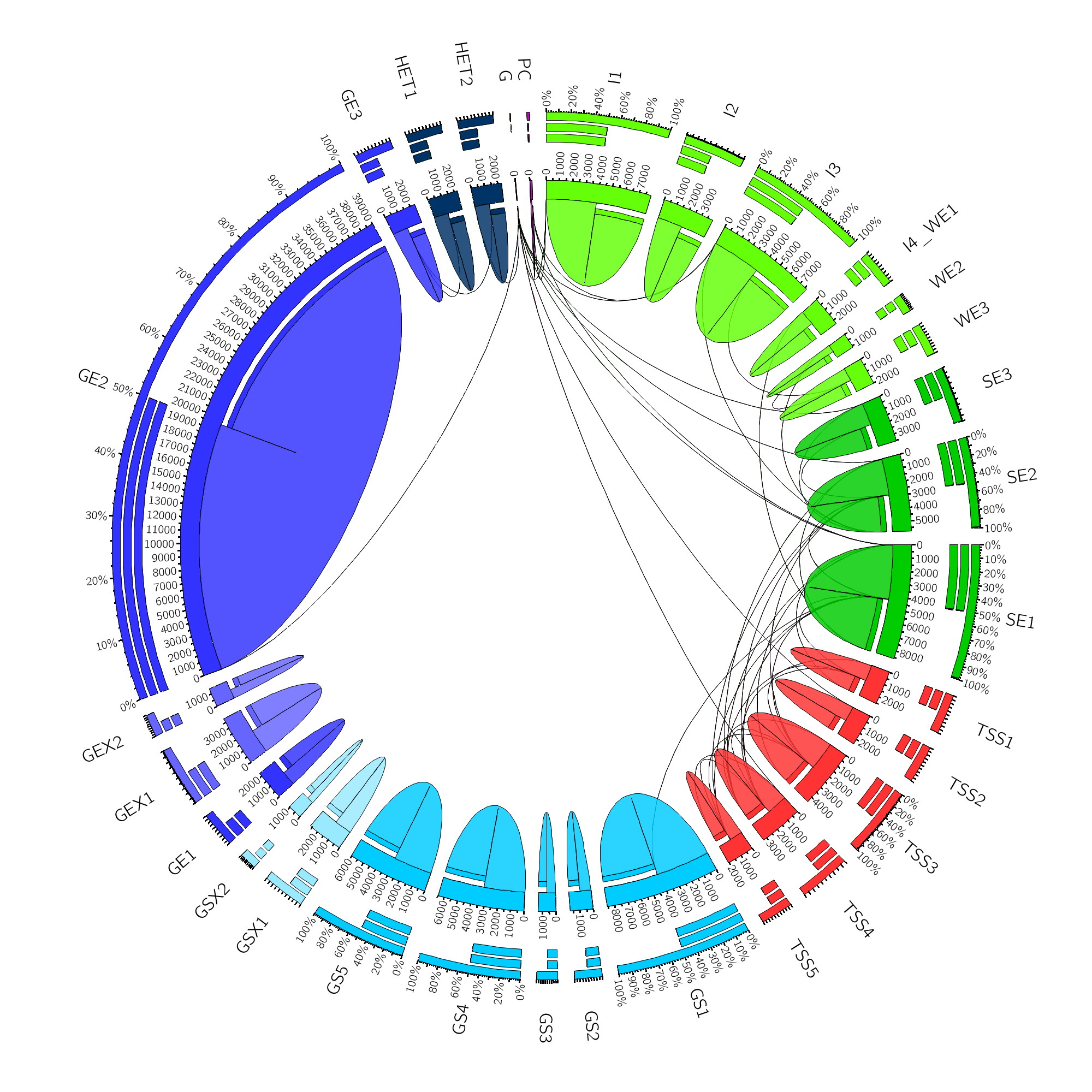

Supplement: Supplementary Data 4 — Effects of positive and negative perturbations of single chromatin factors on chromatin state identity. [file ncomms10528-s5.zip › Supplementary Data 4/PositivePerturbation/Ez.png]

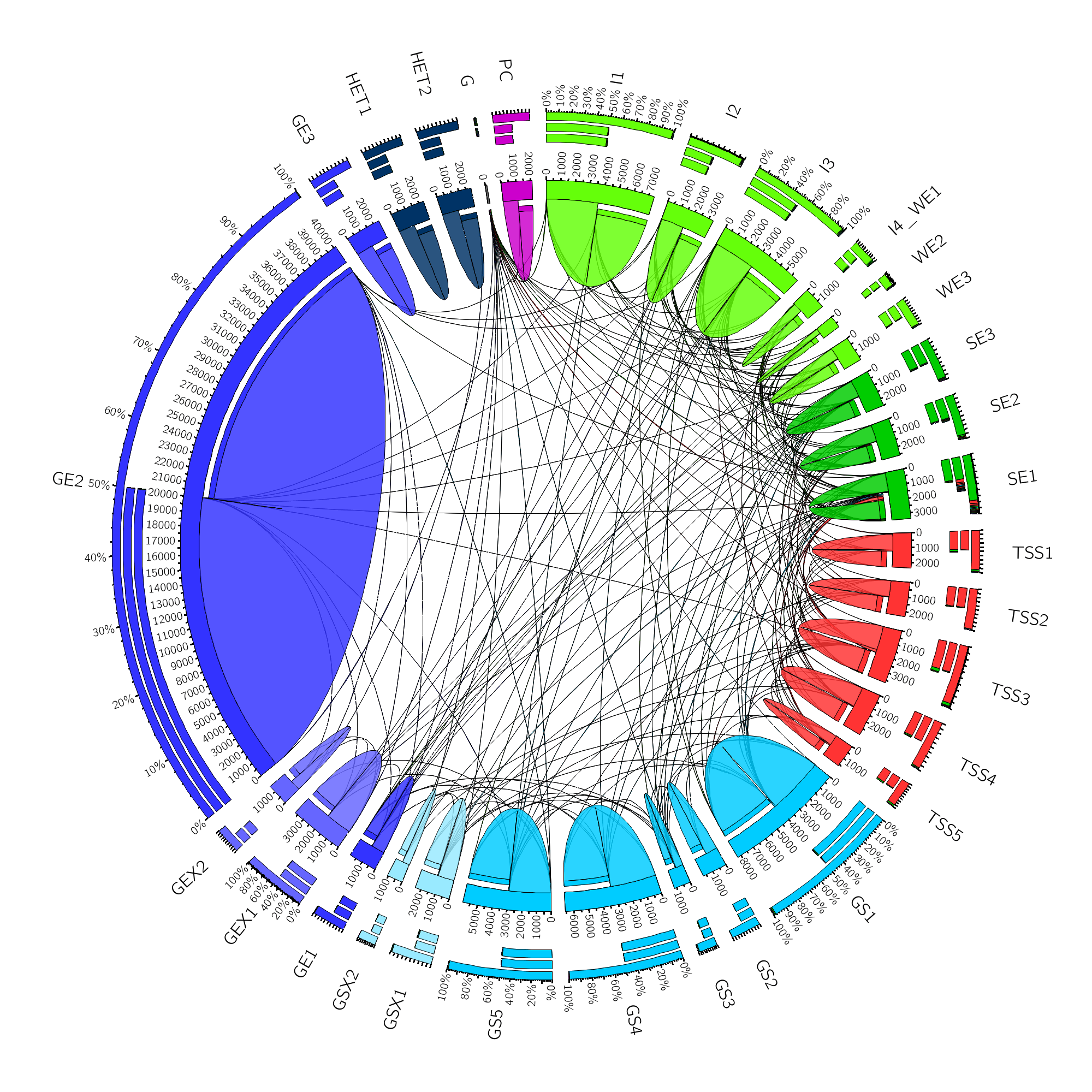

Supplement: Supplementary Data 4 — Effects of positive and negative perturbations of single chromatin factors on chromatin state identity. [file ncomms10528-s5.zip › Supplementary Data 4/PositivePerturbation/GAF.png]

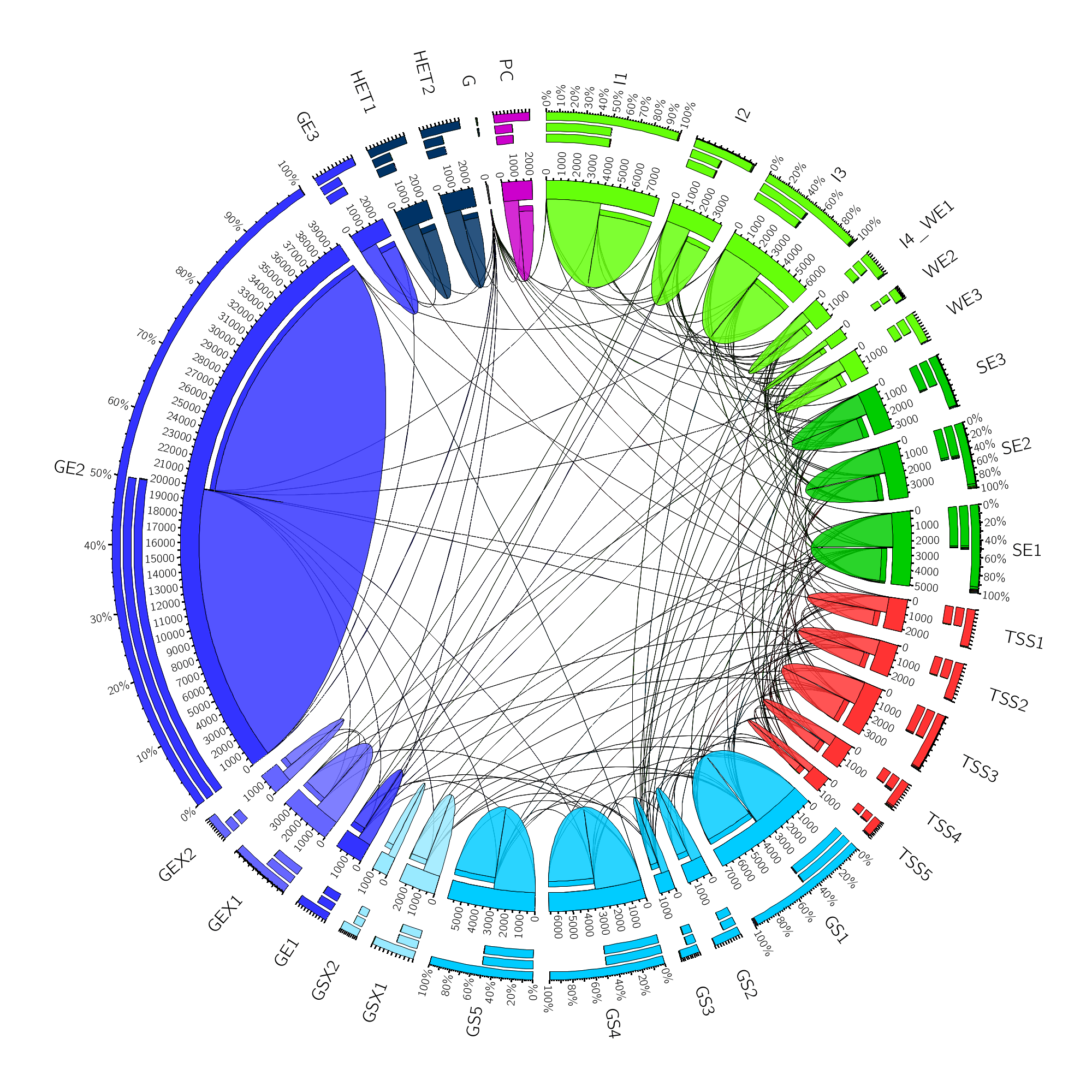

Supplement: Supplementary Data 4 — Effects of positive and negative perturbations of single chromatin factors on chromatin state identity. [file ncomms10528-s5.zip › Supplementary Data 4/PositivePerturbation/H1.png]

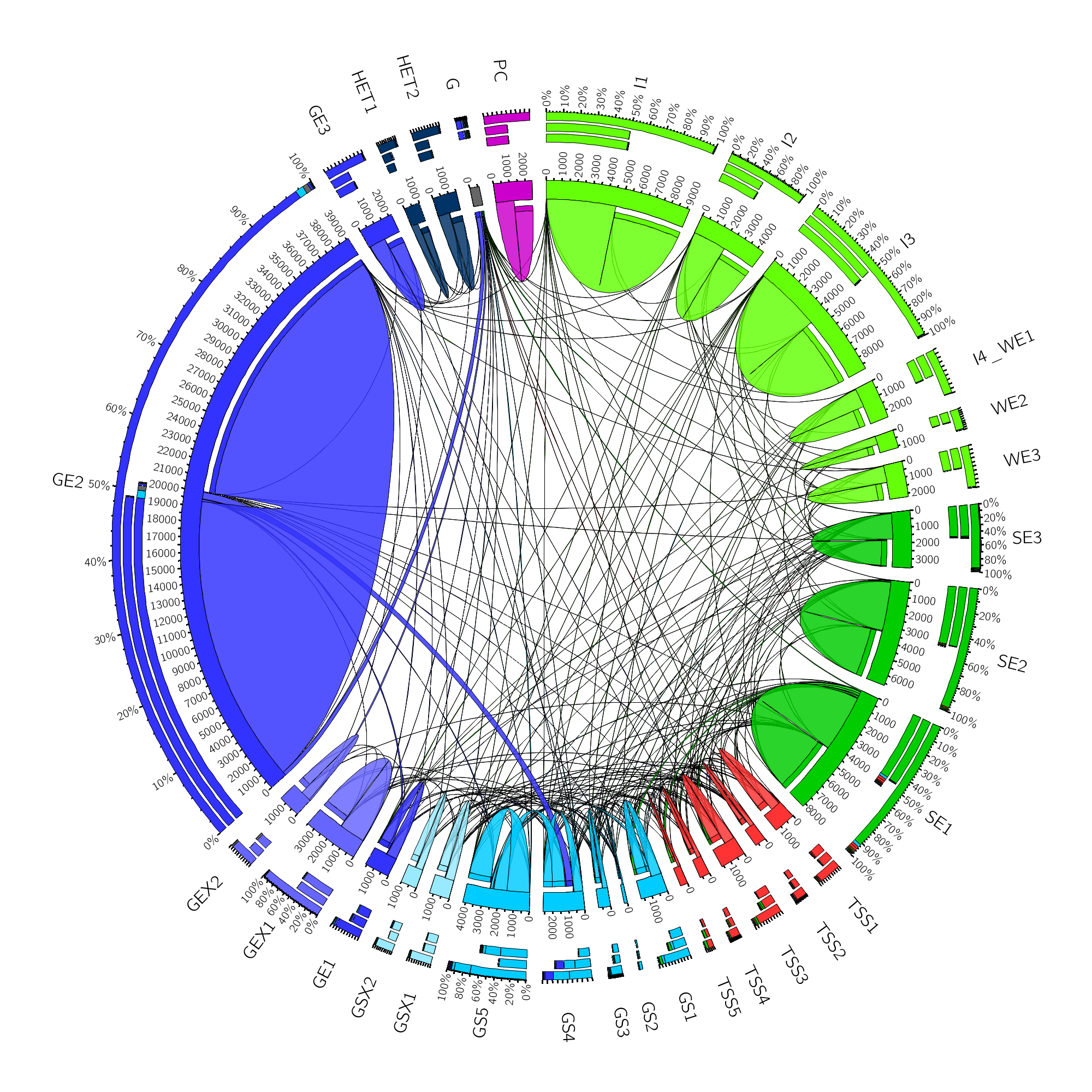

Supplement: Supplementary Data 4 — Effects of positive and negative perturbations of single chromatin factors on chromatin state identity. [file ncomms10528-s5.zip › Supplementary Data 4/PositivePerturbation/H2AV.png]

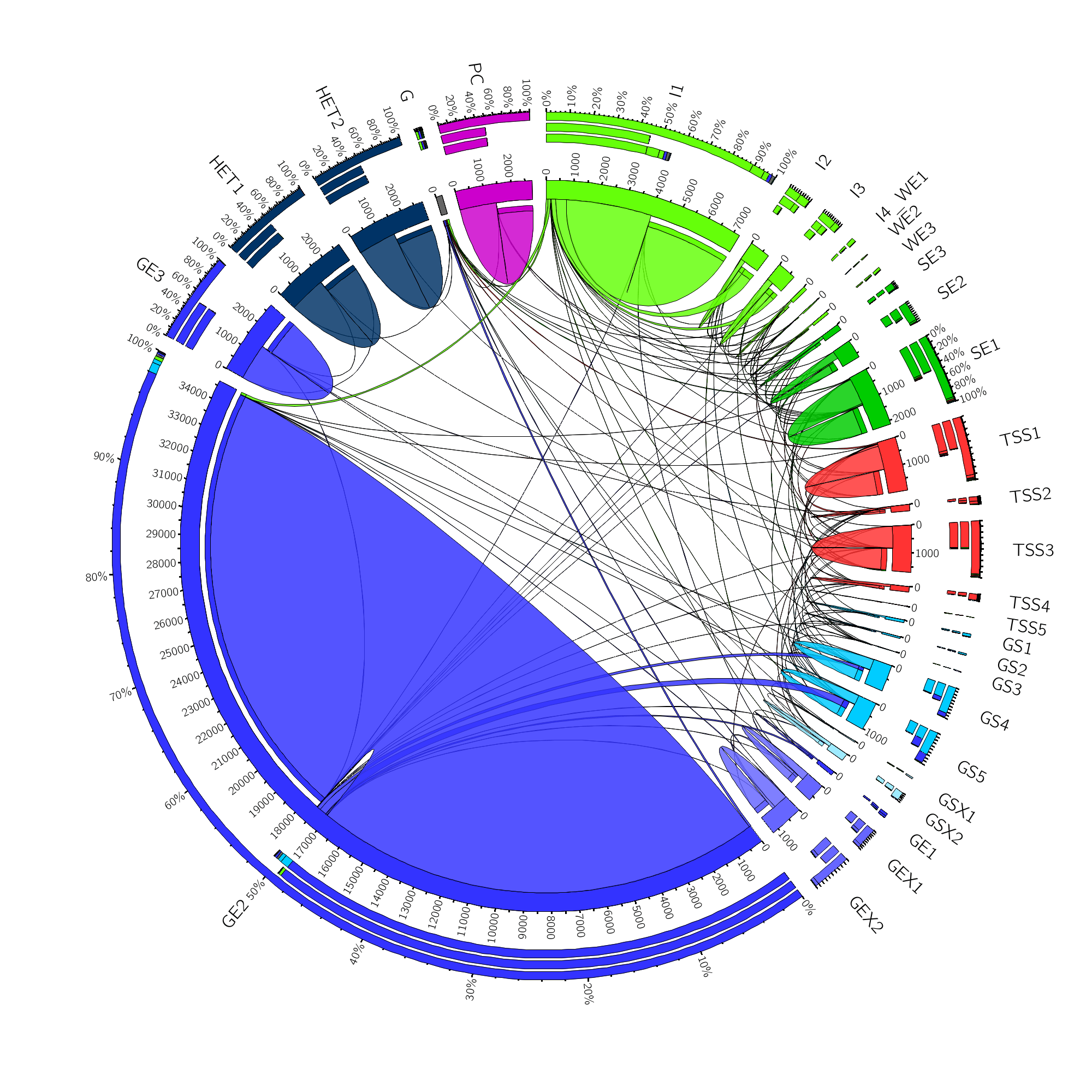

Supplement: Supplementary Data 4 — Effects of positive and negative perturbations of single chromatin factors on chromatin state identity. [file ncomms10528-s5.zip › Supplementary Data 4/PositivePerturbation/H2BK5ac.png]

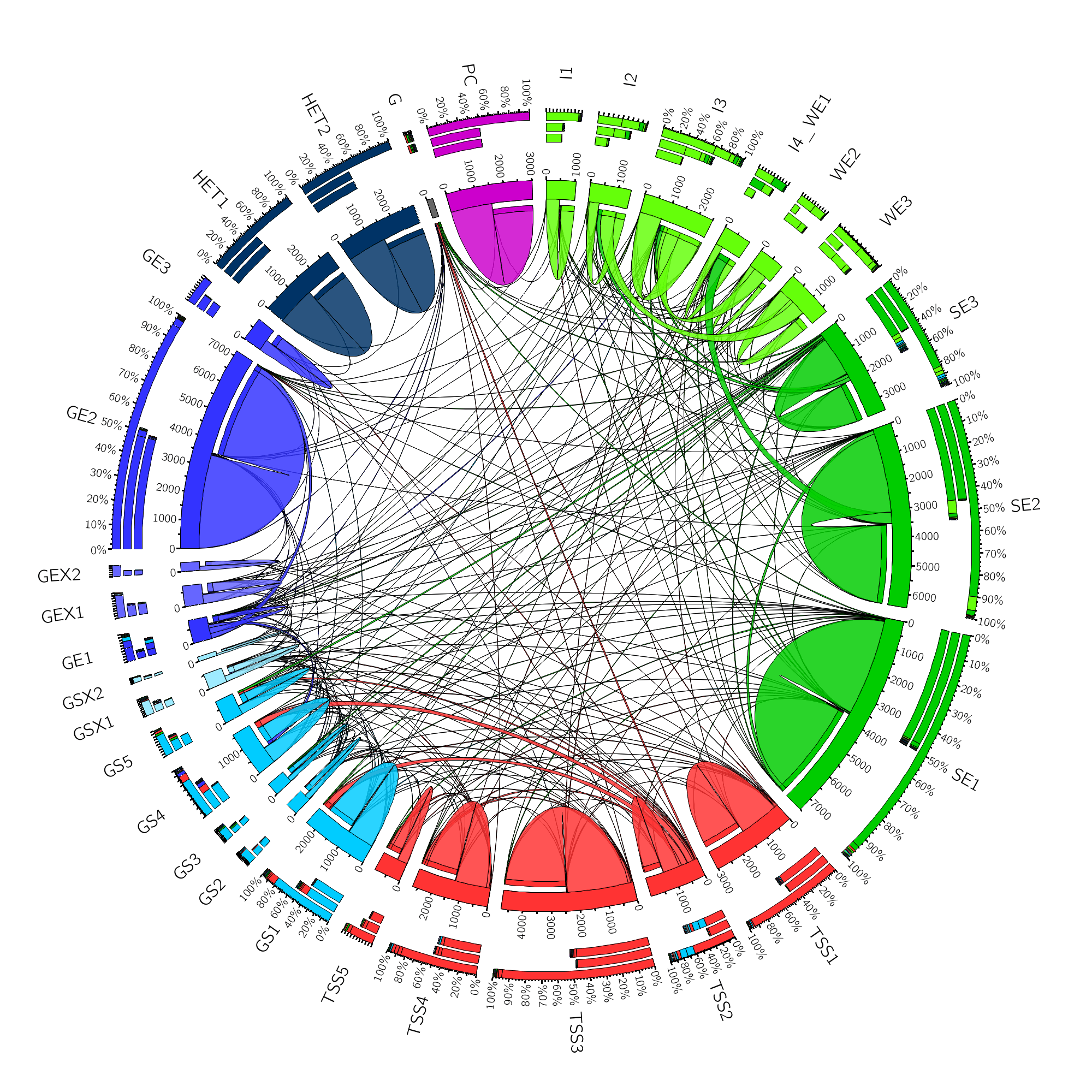

Supplement: Supplementary Data 4 — Effects of positive and negative perturbations of single chromatin factors on chromatin state identity. [file ncomms10528-s5.zip › Supplementary Data 4/PositivePerturbation/H2Bubi.png]

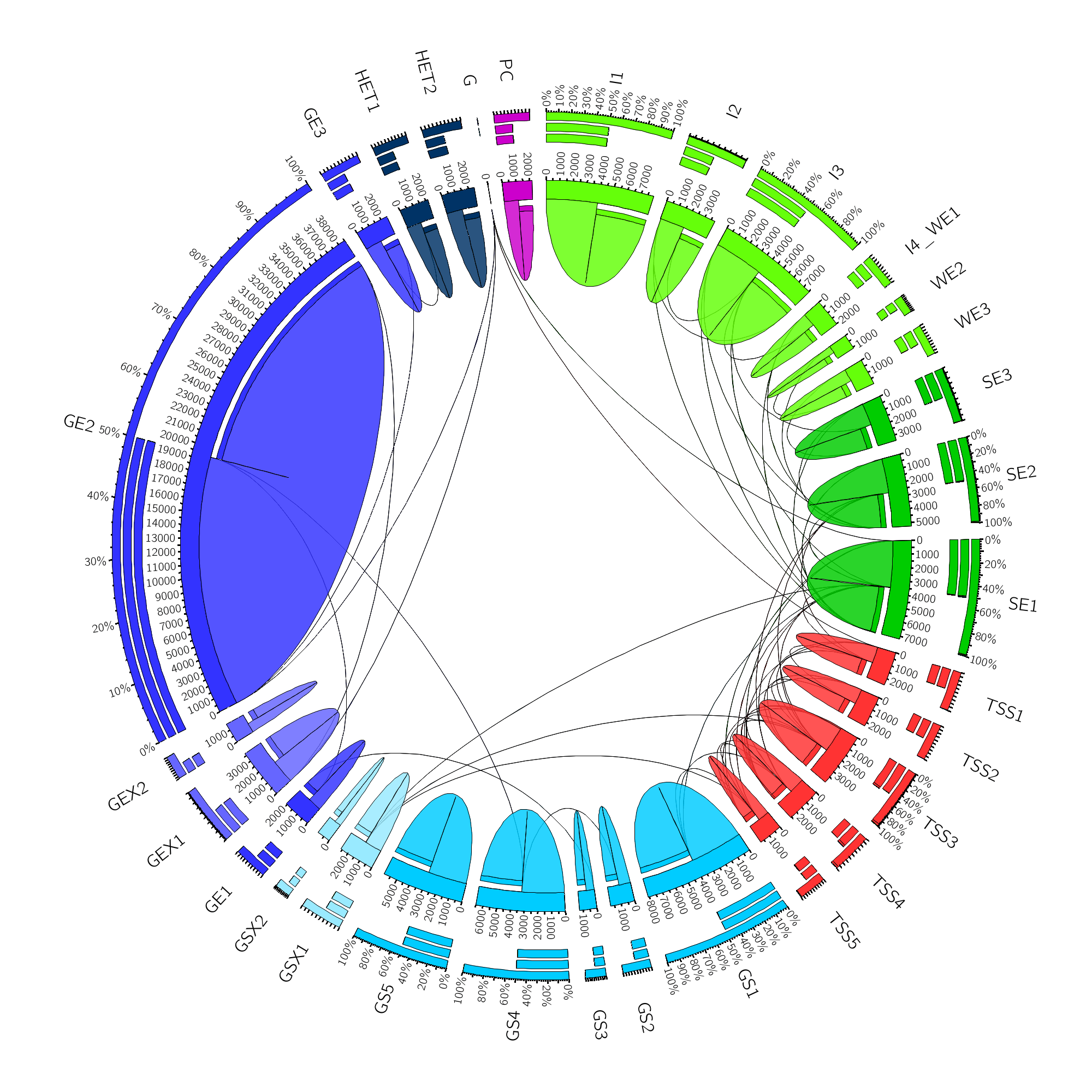

Supplement: Supplementary Data 4 — Effects of positive and negative perturbations of single chromatin factors on chromatin state identity. [file ncomms10528-s5.zip › Supplementary Data 4/PositivePerturbation/H3.png]

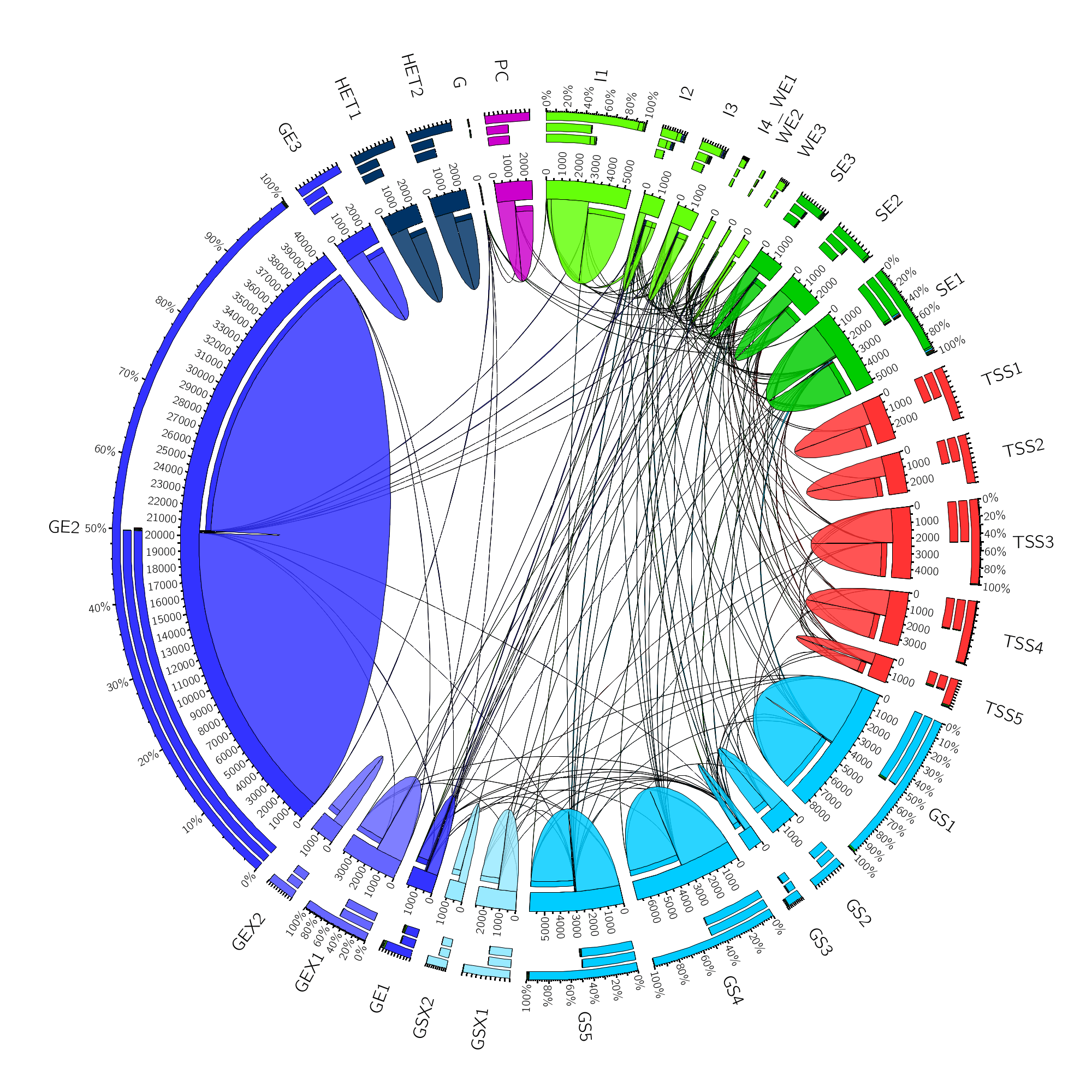

Supplement: Supplementary Data 4 — Effects of positive and negative perturbations of single chromatin factors on chromatin state identity. [file ncomms10528-s5.zip › Supplementary Data 4/PositivePerturbation/H3K18ac.png]

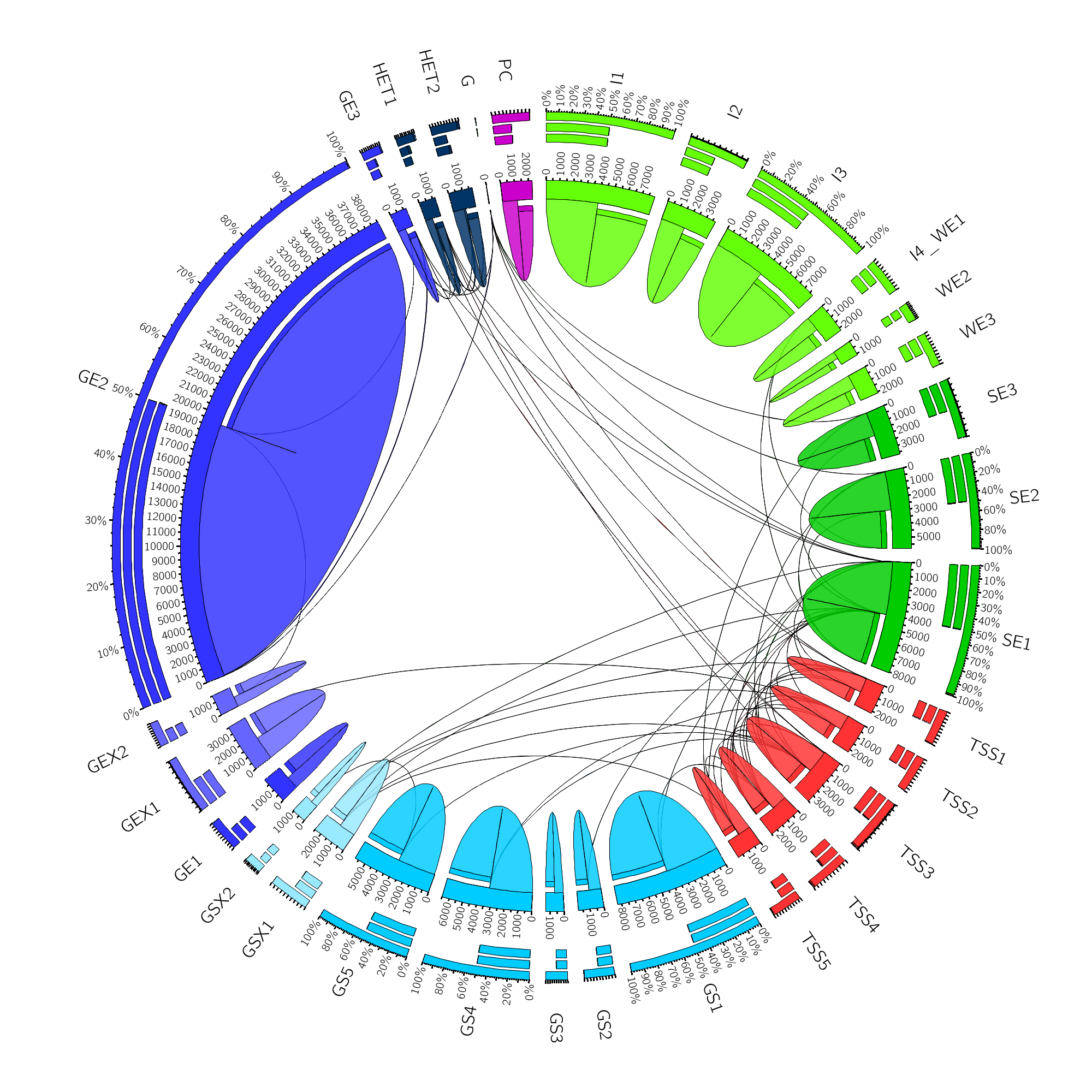

Supplement: Supplementary Data 4 — Effects of positive and negative perturbations of single chromatin factors on chromatin state identity. [file ncomms10528-s5.zip › Supplementary Data 4/PositivePerturbation/H3K23ac.png]

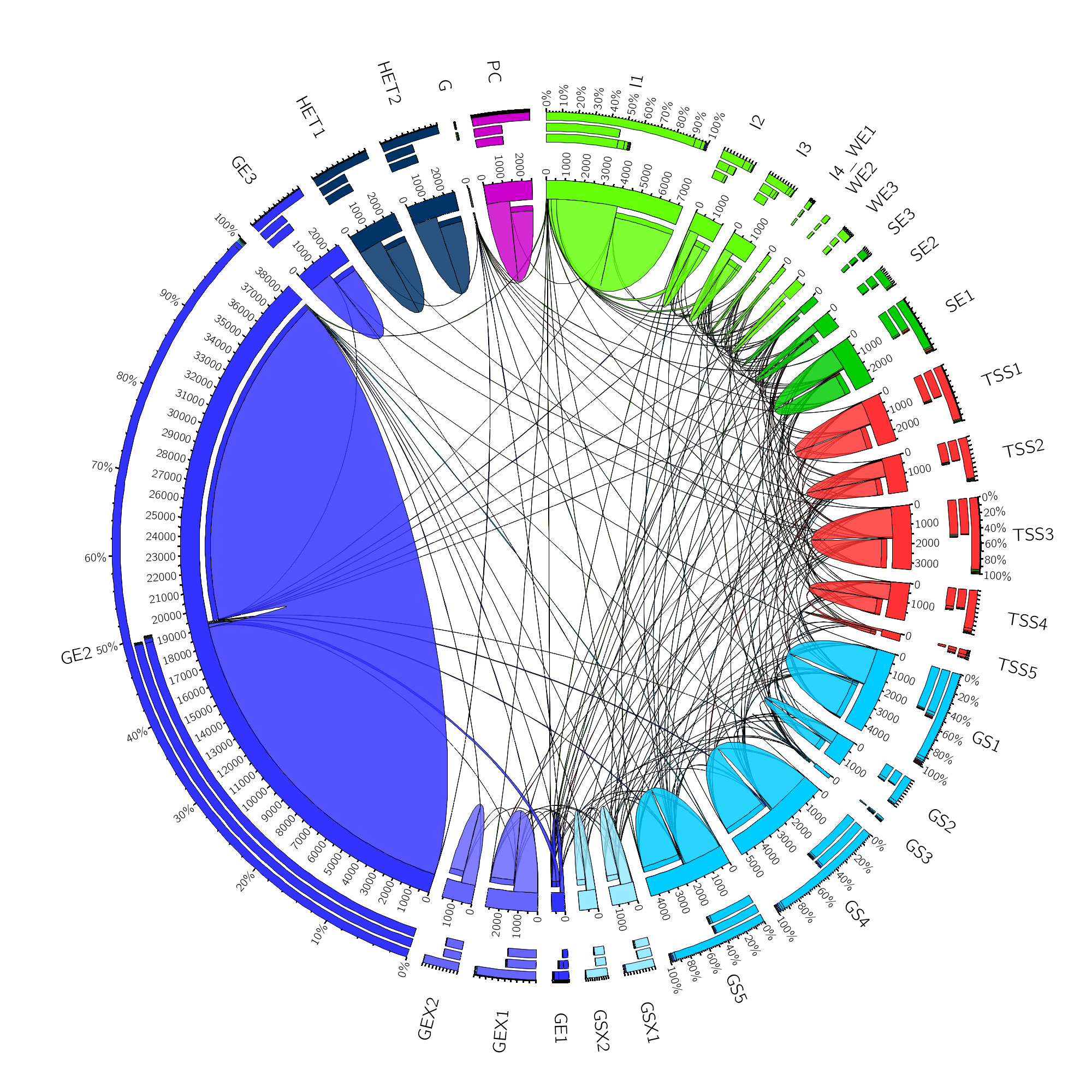

Supplement: Supplementary Data 4 — Effects of positive and negative perturbations of single chromatin factors on chromatin state identity. [file ncomms10528-s5.zip › Supplementary Data 4/PositivePerturbation/H3K27ac.png]

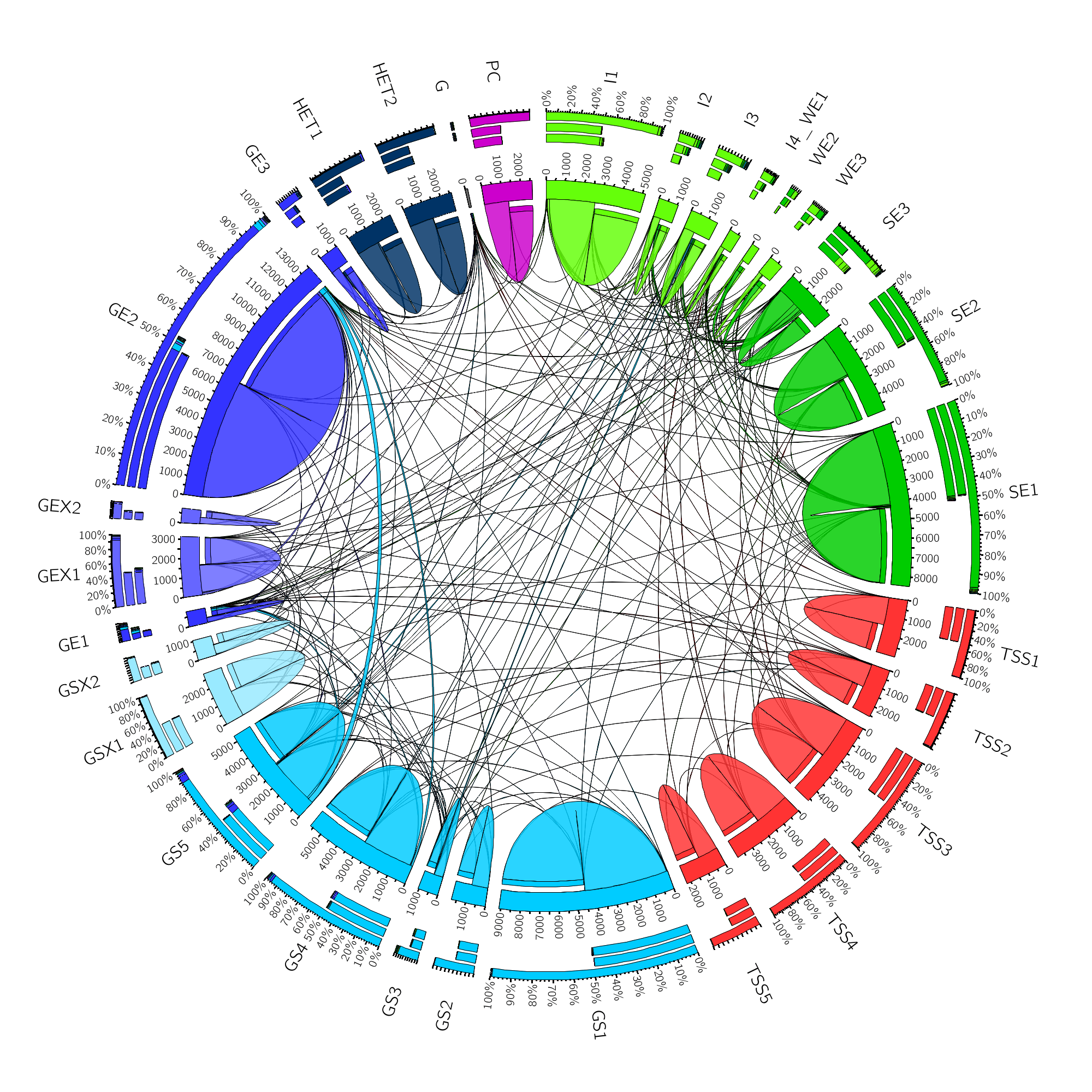

Supplement: Supplementary Data 4 — Effects of positive and negative perturbations of single chromatin factors on chromatin state identity. [file ncomms10528-s5.zip › Supplementary Data 4/PositivePerturbation/H3K27me1.png]

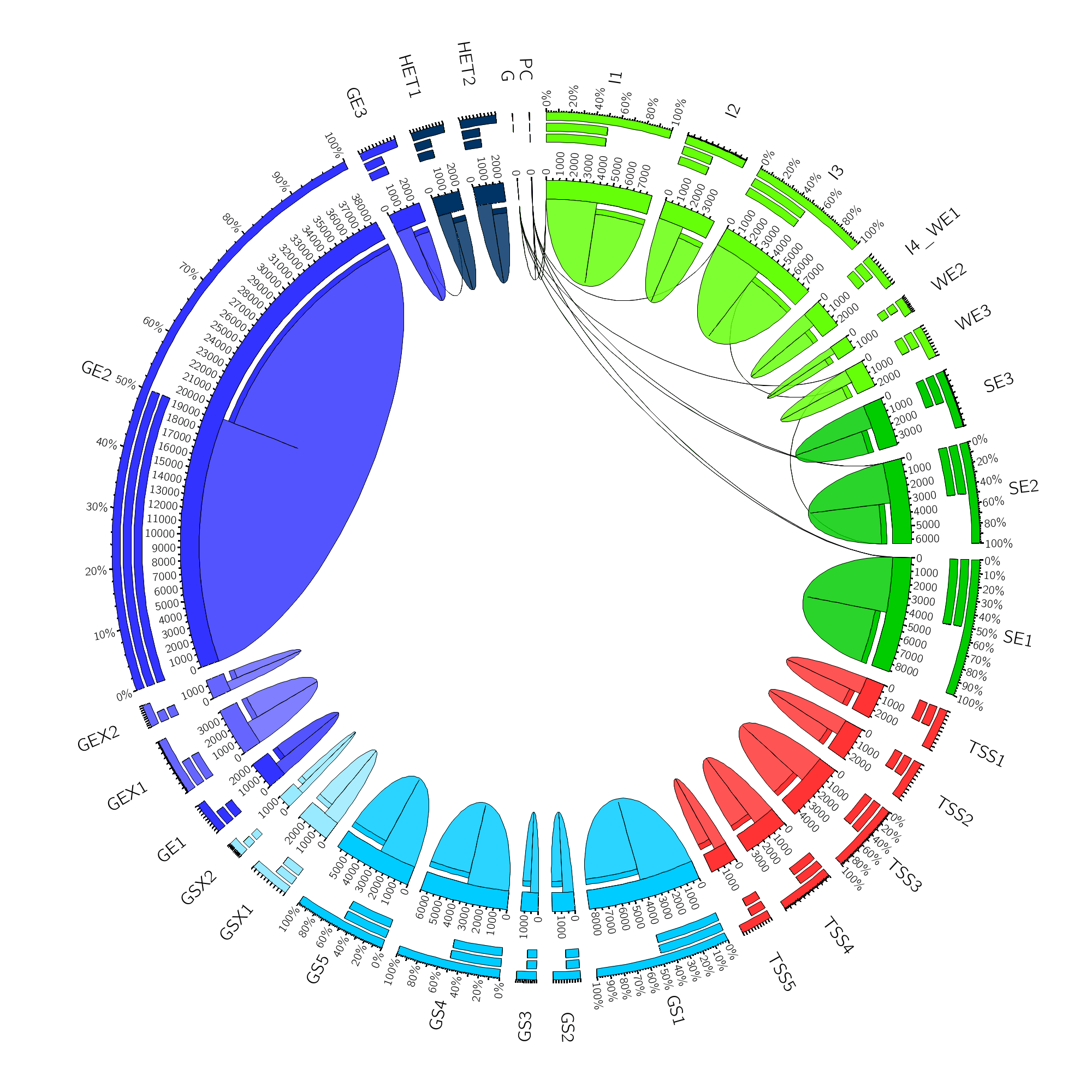

Supplement: Supplementary Data 4 — Effects of positive and negative perturbations of single chromatin factors on chromatin state identity. [file ncomms10528-s5.zip › Supplementary Data 4/PositivePerturbation/H3K27me3.png]

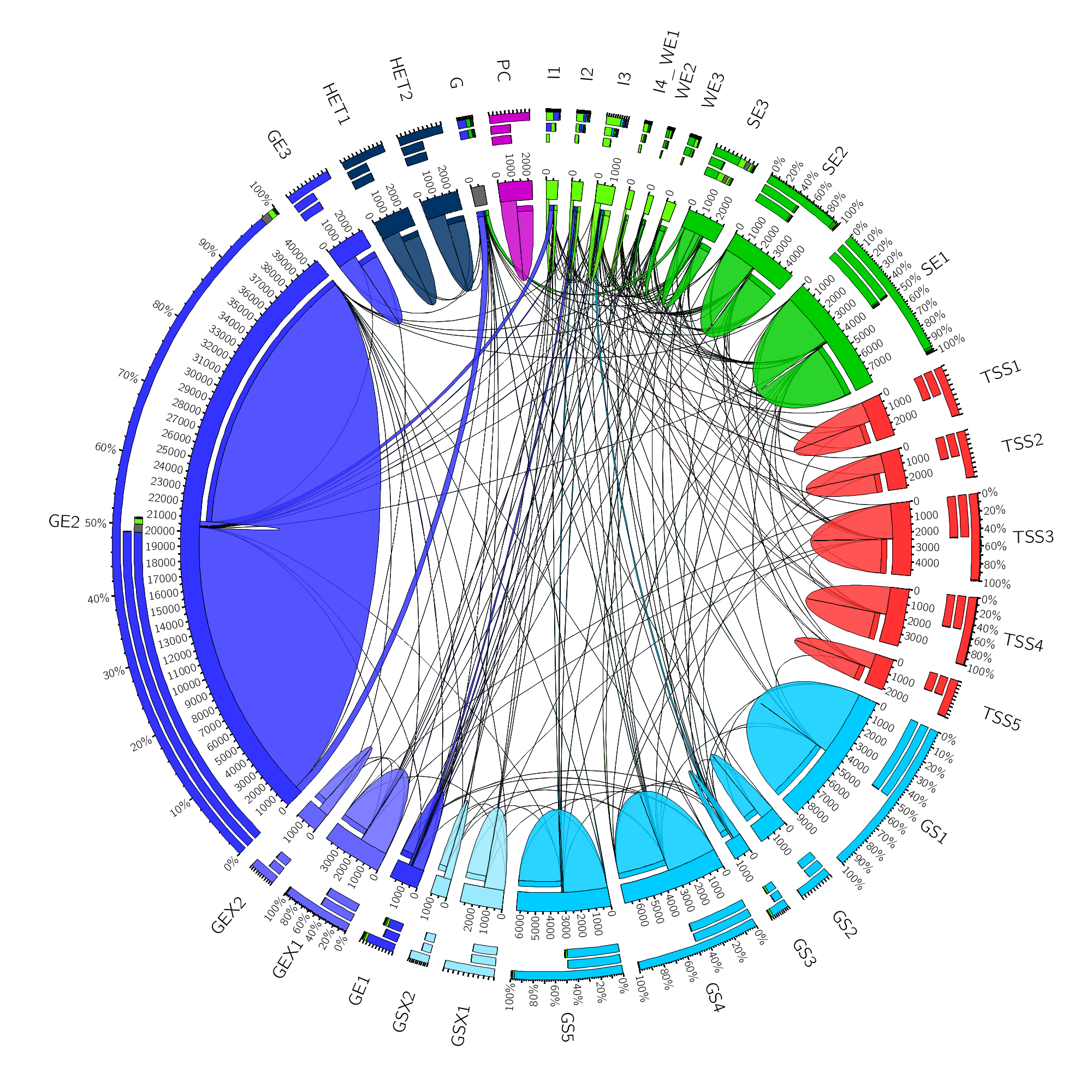

Supplement: Supplementary Data 4 — Effects of positive and negative perturbations of single chromatin factors on chromatin state identity. [file ncomms10528-s5.zip › Supplementary Data 4/PositivePerturbation/H3K36me1.png]

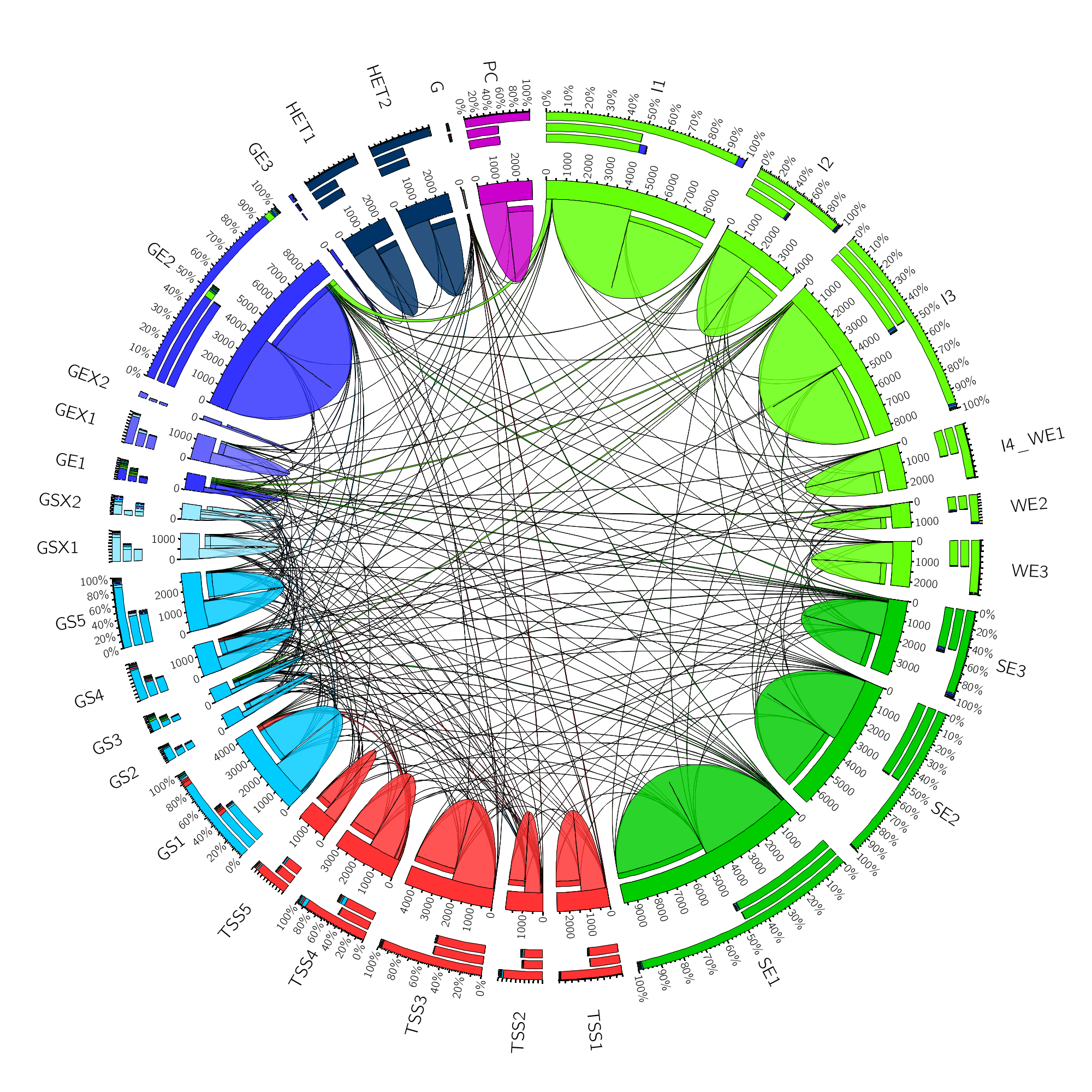

Supplement: Supplementary Data 4 — Effects of positive and negative perturbations of single chromatin factors on chromatin state identity. [file ncomms10528-s5.zip › Supplementary Data 4/PositivePerturbation/H3K36me3.png]

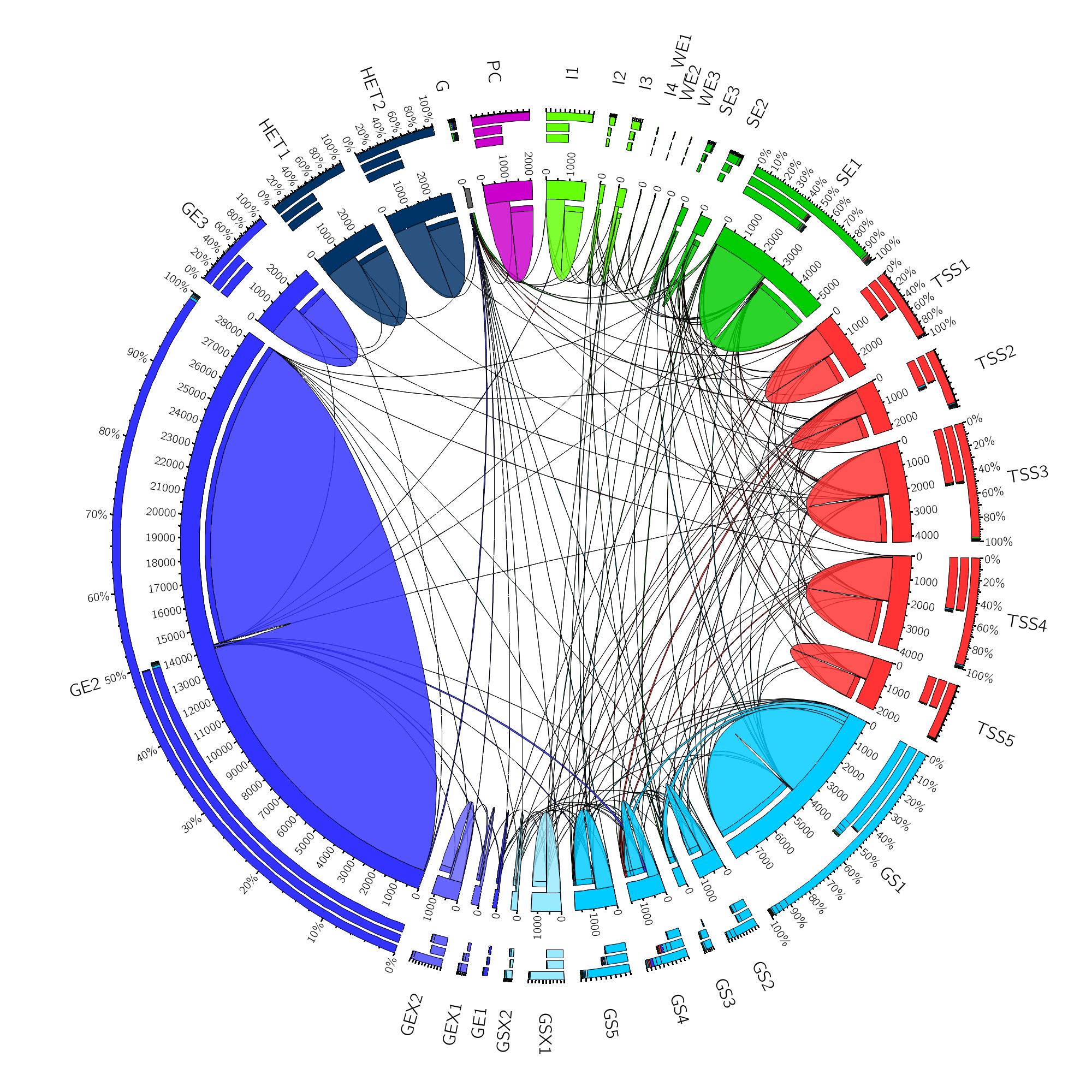

Supplement: Supplementary Data 4 — Effects of positive and negative perturbations of single chromatin factors on chromatin state identity. [file ncomms10528-s5.zip › Supplementary Data 4/PositivePerturbation/H3K4me1.png]

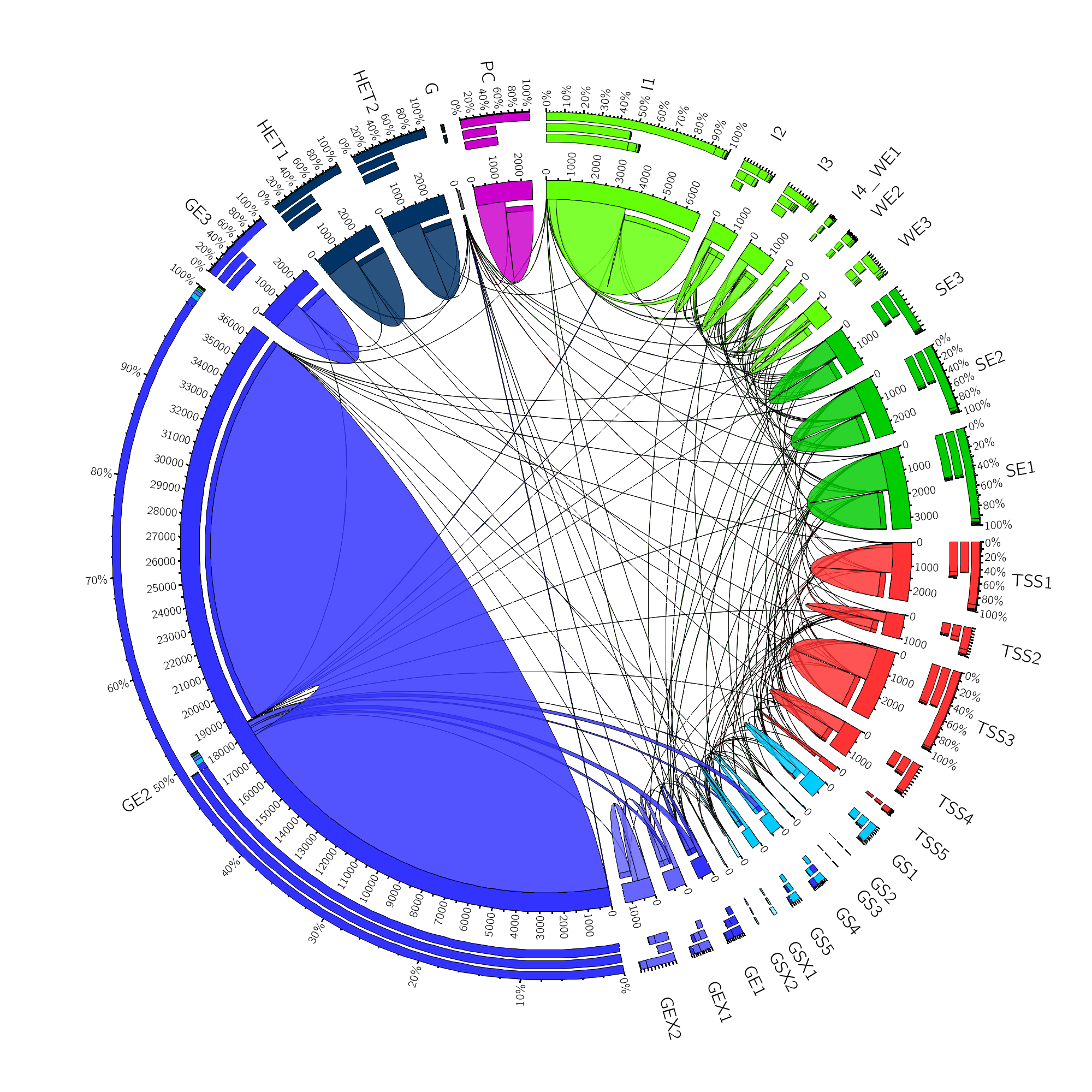

Supplement: Supplementary Data 4 — Effects of positive and negative perturbations of single chromatin factors on chromatin state identity. [file ncomms10528-s5.zip › Supplementary Data 4/PositivePerturbation/H3K4me2.png]

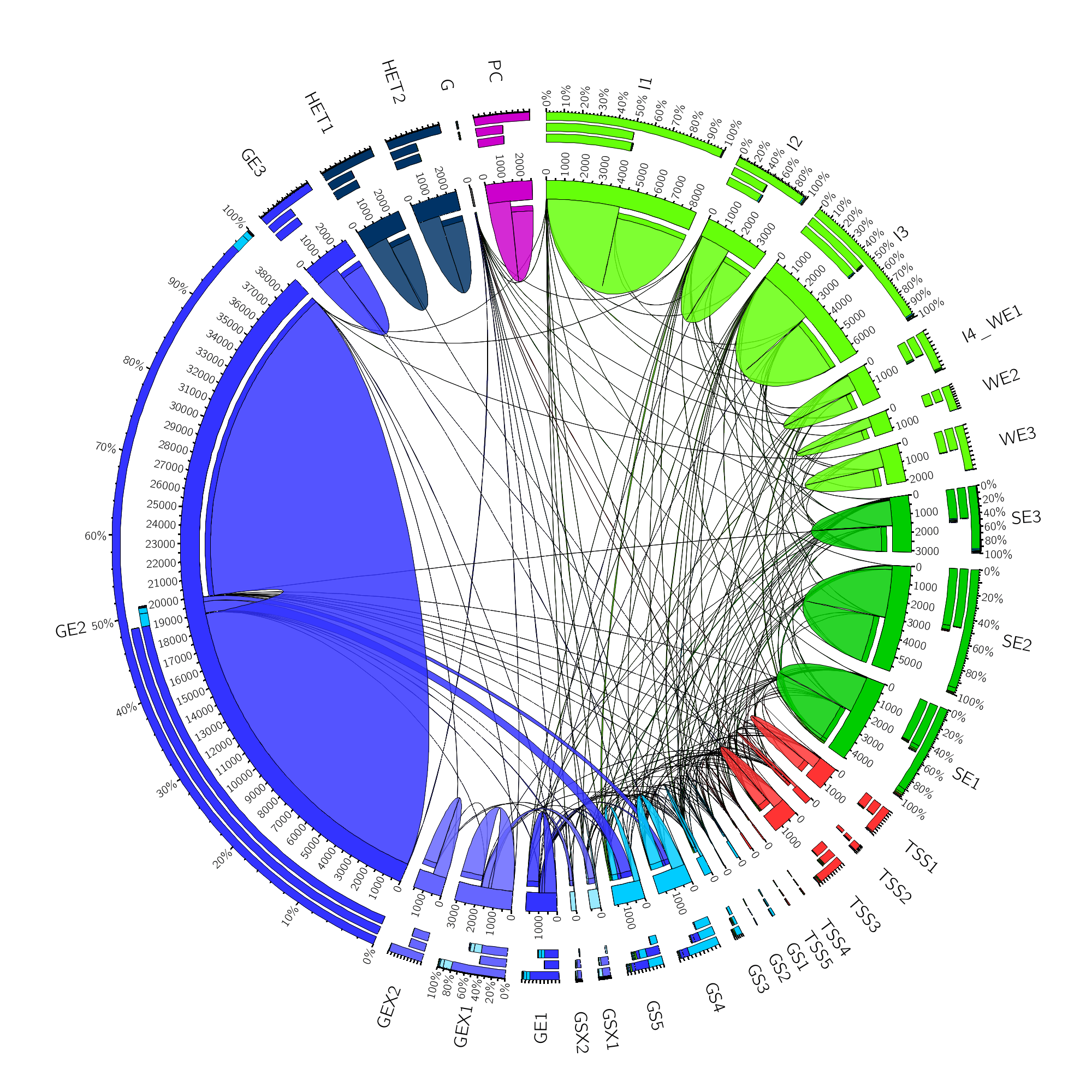

Supplement: Supplementary Data 4 — Effects of positive and negative perturbations of single chromatin factors on chromatin state identity. [file ncomms10528-s5.zip › Supplementary Data 4/PositivePerturbation/H3K4me3.png]
